# Supplementary material for: Factors affecting healthcare workers’ compliance with social and behavioural infection control measures during emerging infectious disease outbreaks: rapid evidence review
Source: BMJ Open. 2021 Aug 14;11(8):e049857. doi: 10.1136/bmjopen-2021-049857 (PMC8370838; doi:10.1136/bmjopen-2021-049857)

Supplementary Table I. Study characteristics

Authors (year)	Country	Disease outbreak	Participants: N, age, gender	Professional role	Protective behaviours examined
Yap et al. (2010)[4]	Singapore	H1N1	331; Mean age 23.2, range 18-61; 7.6% female	Healthcare workers	Questions on preventive practices related to mask wearing, vaccination, avoidance behaviours, personal habits (e.g. washing hands regularly, covering mouth when coughing/sneezing), and medical attention seeking practice
Al-Amri et al. (2019)[12]	Saudi Arabia	MERS	85; 37.6% under 30; 49.4% aged 30-40; 12.9% aged over 40; 38.8% female	General practitioners (62.4%), specialists (27.1%), consultants (10.6%)	'Adherence toward guidelines' assessed through 10 statements; no further information provided
Datta et al. (2011)[13]	India	H1N1	237; 58.23% aged 21-25, 24.05% aged 26-30; 20.25% female	Tertiary care hospital staff: 113 nurses, 41 laboratory technicians, 20 auxilliary nursing midwives, 61 'other' including medical social	Frequent handwashing, use of face mask, use of gloves, closed-front gown, use of disinfectant, 'others'

Brooks SK, *et al. BMJ Open* 2022; 11:e049857. doi: 10.1136/bmjopen-2021-049857

				workers and physiotherapists	
De Perio et al. (2012)[14]	USA	H1N1	88;<br>Median age 30, range 25-53;<br>35% female	Physicians in training: 75 internal medicine residents, 7 cardiology fellows, 6 pulmonary and critical care fellows<br>(only 53 provided sufficient information on protective behaviours)	Respiratory protection: gloves, gowns, surgical masks, fit-tested N95 respirators, powered air-purified respirators, eye protection
Manabe et al. (2012)[15]	Vietnam	Avian influenza	326;<br>Physicians: 39.8% aged 35 or under, 39.8% aged 36-50, 20.4% aged 51 or over, Nurses: 35.6% aged 35 or under, 47.5% aged 36-50, 16.9% aged 51 or over, Lab technicians: 45.5% aged 35 or under, 48.5% aged 36-50, 6.1% aged 51 or over, Other: 43.5% aged	64.7% physicians, 18.1% nurses, 10.1% laboratory technicians, 7.1% other	Wearing PPE to see suspected H5N1 patients

			35 or under, 47.8% aged 36-50, 8.7% aged 51 or over;<br>43.8% female (doctors), 90.0% female (nurses), 84.8% female (lab technicians), 56.5% female (other)		
Nour et al. (2015)[16]	Saudi Arabia	MERS	281;<br>Mean age 30.8, range 21-57;<br>57.7% female	46.3% nurses, 20.6% physicians, 9.6% specialists, 23.5% technicians	Using soap and water to wash hands, covering nose and mouth with a tissue when sneezing or coughing, throwing used tissues away, avoiding touching eyes/nose/mouth, using facemask in crowds, carefully handling suspected patients' belongings, healthy eating/healthy lifestyle, educating patients about disease
Nour et al. (2017)[17]	Saudi Arabia	MERS	188 of the participants in Nour et al. (2015);<br>Age and gender for this sub-group not reported	188 of the participants in Nour et al. (2015);<br>Occupational role for this sub-group not reported	As reported in Nour et al. (2015)

Pratt et al. (2009)[18]	Canada	SARS	536;<br>Mean age 48.4;<br>96.6% female	Hospital nurses; Primary area of practice: 26.2% medicine, surgery; 19.5% maternal newborn, paediatrics; 29.4% emergency, critical care; 24.9% other	Cleaning hands with water and soap or waterless hand rubs after removing disposable gloves, wearing disposable gloves, wearing a disposable outer garment, wearing an N95 mask, wearing protective eyewear
Evirgen et al. (2014)[19]	Turkey	H1N1	22;<br>Mean age 31.95, range 19-48;<br>59.7% female	University hospital staff: 22 professors, 29 researchers, 26 nurses; 35% from surgical departments, 32% from internal medicine departments; 17% from operation room/intensive care unit; 14% from laboratories	Total score for protective behaviours including clearing the air in the environment, washing hands with soap after practice, rubbing hands with antiseptic after practice, using disinfectant at home, H1N1 vaccination, vaccination of children, recommending vaccination to at-risk groups, using medicines supporting the immune system, using alternative medicine drugs, using antivirals for prophylactic purposes, following scientific journals about H1N1 and looking at media statements on H1N1

Kim & Choi (2016)[20]	South Korea	MERS	249;<br>Mean age 21.9, range 20-32;<br>88.4% female	Nursing students in third or fourth year engaged in clinical practice for 8 hours a day at tertiary hospitals with MERS patients	Nine-item scale consisting of five items about reducing the use of public places in daily life, one item about avoiding people with cough, one item about intensive cleaning and disinfection, one item about handwashing and one item about talking with people nearby
Taghrir et al. (2020)[21]	Iran	COVID-19	240;<br>Mean age 23.67;<br>59.2% female	Fifth to seventh year medical students	Preventive behaviours: cancelling social activities, not using public transport, less frequent shopping, reducing the use of closed spaces, cough etiquette, avoiding large gatherings, increased cleaning and disinfecting of surfaces, handwashing, discussing prevention with family and friends
Jeong et al. (2011)[22]	South Korea	H1N1	880;<br>76.3% aged 18-39, 22.4% aged 40-59, 1.4% 60 or over;<br>64.5% female	Hospital employees	The Korea Center of Disease Control and Prevention's recommended behaviours, including washing hands, using a tissue when coughing or sneezing, reducing outings

Chor et al. (2012)[23]	China (Hong Kong), Singapore, UK	H1N1	2100 (1556 Hong Kong, 284 Singapore, 260 UK); Mean age 35 (Hong Kong), 28 (Singapore), 34 (UK); Gender not reported	Doctors, nurses, allied healthcare workers	Washing hands before each patient contact, washing hands after each patient contact, wearing gloves during patient contact, wearing a surgical mask even when not involved in direct patient care, wearing a mask during patient contact
Koh et al. (2009)[24]	Indonesia, Singapore	Avian influenza	333 from Indonesia v 1321 from Singapore plus 51 focus group participants from Indonesia; Quantitative study population: Indonesia: 55.4% aged 40 or under, Singapore: 43.6% aged 40 or under, Qualitative study: Age range 22-56; Quantitative study population: Indonesia: 75.7% female, Singapore: 86.4% female, Qualitative study: 94.1% female	Primary healthcare workers. Quantitative study: Indonesia: 17.6% doctors, 14.6% nurses, 67.8% other. Singapore: 14.6%, 34.2%, 51.2%. 'Other' includes therapists, paramedics, ambulance crew, attendants, pharmacists, social workers, cleaners and administrative staff Qualitative study: 13 physicians, 19 paramedics, 19 nurses	'Adhering to infection control protocols and recommended measures'

Wong et al. (2005)[25]	China (Hong Kong) (n=137), Canada (n=51)	SARS	188;<br>Age not reported;<br>Gender not reported	Family medicine tutors (Hong Kong), family physicians (Canada)	Testing patient temperature as a routine procedure, always wearing mask during consultations, cleaning work surface with antiseptics at least once a day, support staff all wearing masks, wearing gowns for patient encounters, washing hands between every patient, closing clinic, quarantine
Kamate et al. (2020)[26]	International (30.7% from Asia, 25.0% from North/South Americas, 16.3% from Europe, 22.6% from Africa, 5.4% from 'other' (Australia	COVID-19	860;<br>Age reported;<br>Gender not reported	Dental practitioners	Staff sensitised as per World Health Organization guidelines for the prevention of COVID-19, including travel history when recording patient histories, discussing risk and preventive measures related to COVID-19 with patients, showing patients signs and symptoms of COVID-19, taking 'preventive measures' against COVID-19, effect on social life

	and Antarctica)				
Hu et al. (2012)[27]	China	H1N1	650;<br>Median age 28, IQR 25-33;<br>76.3% female	Nurses (421), physicians (229)	Hand hygiene, gloves, gown, mask (surgical and N95 respirator), goggles
Alsahafi & Cheng (2016)[28]	Saudi Arabia	MERS	1216;<br>Age not reported;<br>56.4% female (nurses), 25.5% female (physicians), 23.9% female (other healthcare workers)	Nurses (56.3%), physicians (22%), other healthcare workers (21.7%)	Handwashing after patient contact, wearing of surgical mask during patient contact, wearing of N95 mask during patient contact, training on infectious behaviours, perceived barriers to infection control practices
Alsubaie et al. (2019)[29]	Saudi Arabia	MERS	591;<br>Age not reported;<br>78% female (non-physicians) and 31% female (physicians)	Physicians (55%), non-physicians – including nurses, technicians and respiratory therapists (45%)	Type of preventive behaviours and practices that changed during the outbreak, including hand hygiene and avoidance behaviours such as avoidance of hand-shaking, avoidance of direct contact with people suffering influenza-like illnesses and avoidance of public facilities

Chau et al. (2008)[30]	China (Hong Kong)	SARS	96 (observational study), 109 (interviews); Age not reported; Gender not reported	Healthcare workers and support workers; for interviews: 32 healthcare workers, 30 health care assistants, 47 managers including nursing officers, ward managers, nurse specialists and infection control nurses	Total performance score based on compliance with hand hygiene, mask use, gown use, goggle/face shield precautions, glove use, handling patient care equipment and articles correctly, routine and terminal cleaning, handling linen and laundry, and terminal cleaning of isolation room
Koh et al. (2005)[31]	Singapore	SARS	15,025; Mean age 36.6; 82% female	Healthcare workers from nine major healthcare institutions, incl. doctors, nurses, physiotherapists, attendants, cleaners, pharmacists, managerial staff, clerical staff, service counter staff, other	Hospital's recommended protective practices
May et al. (2010)[32]	USA	H1N1	261; Students: 26% aged under 24, 61% aged 24-29, 10% aged 30-34, 3% 35 and	Medical students (194) and residents (67), at an urban medical teaching centre	Hand sanitiser, handwashing, cough etiquette, disinfection, surgical mask when ill with influenza-like illness, surgical mask in patient care when

			over, Residents: 55% aged 24-29, 36% aged 30-34, 9% 35 and over; Students: 62% female, Residents: 50% female		seeing patients with influenza-like illness, N95 when seeing patients with influenza-like illness, social distancing
Mitchell et al. (2012)[33]	Canada	H1N1	986; Median age 36, range 21-79; 85% female	Nurses (80%), respiratory therapists (14%), physicians (6%)	Use of PPE
Parker & Goldman (2006)[34]	Canada	SARS	116; 27% aged 20-29, 42% aged 30-39, 23% aged 40-49, 8% aged 50 and over; 70% female	Paediatric emergency department staff: 28% physicians, 41% nursing staff, 21% trainees, 10% paramedical staff involved with direct patient care	Wearing N95 mask when examining patients, wearing gown when examining patients, handwashing before and after patient contact, wearing N95 mask at all times in the emergency department, wearing eye protection when examining patients, wearing gloves when examining patients
Shigayeva et al. (2007)[35]	Canada	SARS	795; Median age 41, range 21-67; 74.3% female	46.3% nurses, 13.5% physicians, 14.2% respiratory therapists, 10.3% medical imaging	Use of barrier precautions, practices for removing PPE, infection control training

				technologists, 15.7% other incl. service assistants, physiotherapists, lab technologists, paramedics, housekeeping staff, ward clerks and social workers	
Vinck et al. (2011)[36]	Netherlands	H1N1	166;<br>4% aged 25 or under, 26% aged 26-35, 26% aged 36-45, 32% aged 46-55, 10% aged 56-65;<br>66% female	Public health physicians (46%), public health nurses (51%), health department managers (2%)	Compliance with control measures: applying case definition (i.e. how consistently staff applied the criteria for case definition that was issued to identify suspected patients), amount of consultation with the centralised assessment system for the final classification of patients, use of PPE during patient sampling and home visits, and informing patients and contacts about isolation measures
Alshammari et al. (2018)[37]	Saudi Arabia	MERS	87;<br>Age not reported;<br>Gender not reported	Physicians and nurses	Hand hygiene compliance

Jaeger et al. (2011)[38]	USA	H1N1	63;<br>Median age 35, range 19-74;<br>76% female	70% worked in inpatient settings. 38% clinical practitioner, 10% physician/nurse practitioner/physician assistant, 29% Registered nurse/Registered Dietician, 52% allied health staff (incl. radiology, emergency department and respiratory technicians, phlebotomists, licensed vocational nurses and medical and nursing assistants), 10% support staff (incl. office workers, patient transport and environmental services workers)	PPE use, glove use during index patient exposures, mask or N95 respirator use during index patient encounters

Moore et al. (2005)[39]	Canada	SARS	97;<br>Mean age 43.1, range 26-64;<br>78% female	34% managers, 24% registered nurses, 9% support staff, 8% medical technologists, 6% respiratory therapists, 4% infection control practitioners, 4% physicians, 1% administration staff, 1% pharmacists and 1% physiotherapists	Experience of infection control practices including PPE
Tan et al. (2006)[40]	Singapore	SARS	8;<br>Age not reported;<br>25% female	Family physicians	Participants discussed encounters with suspected SARS patients including their clinical assessment and management of patients in clinics, problems encountered, and use of PPE and other preventive measures
Corley et al. (2010)[41]	Australia	H1N1	32 (questionnaire study), 16 (focus groups)<br>Age not reported;<br>Gender not reported	Questionnaire: 28 nursing staff, 4 medical staff;<br>Focus groups: 4 senior medical staff, 4 senior	How resources were managed during the pandemic

				nursing staff, 8 bedside nursing staff	
Hsu et al. (2011)[42]	Singapore	H1N1	123;<br>Age not reported;<br>Gender not reported	Final-year medical students	Hand hygiene
Chia et al. (2005)[43]	Singapore	SARS	6,198;<br>Mean age 35.8 (doctors), 34.6 (nurses), 38.4 (clerical staff);<br>27.1% female (doctors), 48.8% female (nurses), 77.1% female (clerical staff)	Doctors (14.1%), nurses (4404 – 71%), clerical staff (14.9%)	Perception of risk of infection and preventive measures
Ki et al. (2019)[44]	South Korea	MERS	446;<br>Age not reported;<br>Gender not reported	Medical employees; 228 in the emergency department, 218 in general wards	PPE (face shields or goggles, surgical masks, N95 respirators, gloves, disposable gowns, disposable coveralls, waterproof boots, head cover), hand-washing, hand-rubbing, isolation
Wong et al. (2004)[45]	China (Hong Kong)	SARS	320;<br>Mean age of general practitioners 44.4, mean	General practitioners (137), family medicine tutors (183)	Clinical practices and behaviours: wearing mask during all consultations, wearing goggles, wearing gowns,

			age of family medicine tutors not reported; 17.6% female (general practitioners), 15.3% female (family medicine tutors)		washing hands after seeing patients, insisting patients wear masks, routinely testing patients' temperatures, keeping greater distance from patients, seeing patients at faster pace, avoiding physical examination, requesting more blood tests/chest radiographs, over-prescribing antibiotics, cancelling own appointments, cancelling specialist appointments, difficulties making specialty referrals, closing clinic, reducing working hours, quarantine, requiring staff to wear masks/disposable gloves, cleaning work surfaces with antiseptics, taking staff temperatures, quarantined leave for staff, showering before leaving work, washing hands before entering home, using disinfectant to clean home, staying away from home, wearing a mask at home, sending family away

Yassi et al. (2005)[46]	Canada	SARS	Participants were those reported in Moore et al. (2005)	Participants were those reported in Moore et al. (2005)	Participants discussed barriers to wearing PPE and following infection control procedures
DiGiovanni et al. (2004)[47]	Canada	SARS	195;<br>Age not reported;<br>Gender not reported	76 physicians, 52 nurses, 67 'other' including receptionists, technicians, social workers, research assistants and clinic/office managers	Quarantine
Edeghere et al. (2015)[48]	UK	H1N1	20;<br>Mean age 43.9;<br>80% female	Frontline healthcare workers: 10 clinical attendants, 7 nurses, 3 general practitioners	Use of PPE and infection control measures
Khalid et al. (2016)[49]	Saudi Arabia	MERS	117;<br>Mean age 38.55;<br>76% female	Healthcare workers who worked in high-risk areas. 76% nurses, 14% physicians, 10% respiratory therapists. 65% worked in critical care units, 20% in emergency	Perceived stressors

Brooks SK, *et al. BMJ Open* 2022; 11:e049857. doi: 10.1136/bmjopen-2021-049857

				medicine, 15% in outpatient family medicine	
Locatelli et al. (2012)[50]	USA	H1N1	33;<br>Age not reported;<br>Gender not reported	Infection control key informants at nationally dispersed Veterans Affairs healthcare facilities	Communication about infection control
Rebmann & Wagner (2009)[51]	USA (n=37), UK (n=1, Canada (n=1), Australia (n=1)	H1N1	40;<br>2.5% aged 30-39, 27.5% aged 40-49, 45.0% aged 50-59, 22.5% aged 60-69, 2.5% 70 or over;<br>92% female	Infection preventionists	Issues relating to infection prevention guidance and PPE
Rowlands (2007)[52]	Singapore	SARS	28;<br>Age not reported;<br>75% female	Medical social workers	Experiences of working during the SARS crisis, and the various infection control strategies used at work
Van Dijk et al. (2015)[53]	Netherlands	H1N1	875;<br>General practitioners: 17.5% under 40, 24.5% aged 40-49, 44.9% aged 50-59, 13.2% aged 60+;	General practitioners (372), practice assistants (503)	Experience and acceptance of the national guidelines for infection control

			age for practice assistants not reported<br>General practitioners: 39.3% female; gender of practice assistants not reported		
Kang et al. (2018)[54]	South Korea	MERS	7;<br>Age not reported;<br>Gender not reported	Infection control nurse leaders from major hospitals	Use of PPE equipment
Wong et al. (2012)[55]	China (Hong Kong)	H1N1	10;<br>Mean age 34.9, range 24-49;<br>70% female	4 physicians, 3 nurses, 3 healthcare assistants all working in isolation wards	Concerns relating to care of patients with H1N1
Lam & Hung (2013)[56]	China (Hong Kong)	H1N1	10;<br>20% aged 20-25, 20% aged 26-30, 20% aged 31-35, 30% aged 36-40, 10% over 40;<br>100% female	Emergency nurses: 80% staff nurse, 10% advanced practice nurse, 10% nursing officer	Use of PPE
Nickell et al. (2004)[57]	Canada	SARS	2001;	33.1% allied health care professionals, 31.9% areas	Precautionary measures in the workplace: mask, restricted access (own

			13.6% aged under 30, 25.7% aged 30-39, 30.6% aged 40-49, 30.2% 50 or over; 78.8% female	other than patient care e.g. administration, food services, maintenance or research, 25.6% nurses, 9.3% doctors	hospital), restricted meetings, restricted access (other hospitals), gloves, goggles, 'other'
Kang et al. (2018)[58]	South Korea	MERS	27; Mean age 29.5; 92.6% female	Nurses	Working experiences and challenges faced during the outbreak
Khoo et al. (2005)[59]	Singapore	SARS	51; Age not reported; Female:male ratio was 2:1	19 doctors, 31 nurses/nursing aides, 1 respiratory therapist	Opinions on 3M powered air-purifying respirator and Stryker powered air-purifying respirator
Straus et al. (2004)[60]	Canada	SARS	14; Age not reported; Gender not reported	Physicians from divisions of infectious diseases, general internal medicine, and critical care medicine at university hospitals	Participants discussed barrier precautions such as protective clothing
Tolomiczenko et al. (2005)[61]	Canada	SARS	300; Mean age 42.6, range 20-75; 73.9% female	Registered nurses (25.2%), physicians (20.7%), management (9.5%), services (facilities management, food	Participants discussed patient care and communication about directives

				services, environmental – 3.7%), health professional (occupational therapists, physiotherapists, speech-language pathologists – 17.0%), other (counsellors, technicians, clerks, chaplains – 23.8%)	
Hsu et al. (2006)[62]	Taiwan	SARS	312;<br>4.0% under 30, 32.9% aged 30-39, 48.5% aged 40-49, 14.6% 50 over over;<br>Gender not reported	Public health nurses	Self-protection equipment
Nhan et al. (2012)[63]	Canada	H1N1	129;<br>Age not reported;<br>Gender not reported	68 infectious diseases specialist/medical microbiologists, 61 public health and preventive medicine specialists	Difficulties and frustrations experienced during the pandemic
Rambaldini et al. (2005)[64]	Canada	SARS	17;<br>Age not reported;	Medical house staff allocated to a general	Participants discussed hospitals' communication of directives

			Gender not reported	internal medicine clinical teaching unit, infectious disease unit, infectious diseases consultation service, or intensive care unit	
Robertson et al. (2004)[65]	Canada	SARS	10;<br>Mean age 43.1;<br>60% female	4 nurses, 1 hospital executive, 1 dentist, 1 social worker, 1 occupational therapist, 1 paramedic, 1 physician	Experiences of quarantine and perceptions of infection control guidelines
Goulia et al. (2010)[66]	Greece	H1N1	469;<br>Mean age 38.4;<br>68.4% female.	Nurses (44.6%), medical staff (25.6%), allied staff (12.6%), auxiliary staff (17.3%)	Restriction of social contacts, isolation, intended work avoidance, sense of duty

Supplementary Table II. Summary of themes

Theme	Significant association with protective behaviours	No significant association with protective behaviours	Reported, but not statistically analysed as a potential predictor of protective behaviours
Socio-demographics and personal characteristics			
Age	Kim & Choi (2016)[20]	Al-Amri et al. (2019)[12]<br>Datta et al. (2011)[13]<br>De Perio et al. (2012)[14]<br>Manabe et al. (2012)[15]<br>Nour et al. (2015)[16]<br>Nour et al. (2017)[17]<br>Pratt et al. (2009)[18]	N/A
Gender	Nour et al. (2015)[16]<br>Jeong et al. (2011)[22]<br>Vinck et al. (2011)[36]	Al-Amri et al. (2019)[12]<br>Datta et al. (2011)[13]<br>De Perio et al. (2012)[14]<br>Nour et al. (2017)[17]<br>Evirgen et al. (2014)[19]<br>Kim & Choi (2016)[20]	N/A

		Taghrir et al. (2020)[21]	
Nationality	Al-Amri et al. (2019)[12]	N/A	N/A
Country	Chor et al. (2012)[23]<br>Koh et al. (2009)[24]*<br>Wong et al. (2005)[25]*<br>Kamate et al. (2020)[26]*	Koh et al. (2009)[24]*<br>Wong et al. (2005)[25]*<br>Kamate et al. (2020)[26]*	N/A
Religion	N/A	Kim & Choi (2016)[20]	N/A
Socio-economic status	Jeong et al. (2011)[22]	N/A	N/A
Marital status	N/A	Pratt et al. (2009)[18]<br>Evirgen et al. (2014)[19]	N/A
Level of education	Al-Amri et al. (2019)[12]	Datta et al. (2011)[13]<br>Pratt et al. (2009)[18]<br>Kim & Choi (2016)[20]<br>Taghrir et al. (2020)[21]	N/A
Personal vulnerabilities	N/A	Evirgen et al. (2014)[19]	N/A
Vulnerable people at home	Evirgen et al. (2014)[19]*	Evirgen et al. (2014)[19]*	N/A
Vaccination status	Hu et al. (2012)[27]	N/A	N/A
Occupational role			

Role	Al-Amri et al. (2019)[12]<br>Pratt et al. (2009)[18]<br>Evirgen et al. (2014)[19]<br>Chor et al. (2012)[23]<br>Alsahafi & Cheng (2016)[28]<br>Alsubaie et al. (2019)[29]<br>Chau et al. (2008)[30]<br>Koh et al. (2005)[31]<br>Mitchell et al. (2012)[33]<br>Parker & Goldman (2006)[34]*<br>Shigayeva et al. (2007)[35]<br>Vinck et al. (2011)[36]<br>Chia et al. (2005)[43]	Datta et al. (2011)[13]<br>Nour et al. (2015)[16]<br>Nour et al. (2017)[17]<br>Parker & Goldman (2006)[34]*<br>Alshammari et al. (2018)[37]<br>Jaeger et al. (2011)[38]	May et al. (2010)[32]
Length of experience	Koh et al. (2005)[31]<br>Mitchell et al. (2012)[33]<br>Vinck et al. (2011)[36]	Al-Amri et al. (2019)[12]<br>Nour et al. (2015)[16]<br>Nour et al. (2017)[17]<br>Pratt et al. (2009)[18]<br>Chau et al. (2008)[30]	N/A
Training and knowledge			

Brooks SK, *et al. BMJ Open* 2022; 11:e049857. doi: 10.1136/bmjopen-2021-049857

Receiving training/education	May et al. (2010)[32]*<br>Shigayeva et al. (2007)[35]	Nour et al. (2017)[17]<br>Taghrir et al. (2020)[21]<br>May et al. (2010)[32]*	Alsahafi & Cheng (2016)[28]<br>Moore et al. (2005)[39]<br>Tan et al. (2006)[40]<br>Corley et al. (2010)[41]
Sources of knowledge	Al-Amri et al. (2019) [12]	Kim & Choi (2016)[20]<br>Taghrir et al. (2020)[21]	N/A
Knowledge (of correct practices / outbreak)	Yap et al. (2010)[4]<br>De Perio et al. (2012)[14] *<br>Nour et al. (2015)[16]<br>Kim & Choi (2016)[20]*<br>Jeong et al. (2011)[22]<br>Hu et al. (2012)[27]	Al-Amri et al. (2019)[12]<br>De Perio et al. (2012)[14] *<br>Kim & Choi (2016)[20]*	Alsahafi & Cheng (2016) [28]<br>Hsu et al. (2011)[42]
Work-related factors			
Setting	De Perio et al. (2012)[14]<br>Chau et al. (2008)[30]<br>Mitchell et al. (2012)[33]<br>Shigayeva et al. (2007)[35]<br>Jaeger et al. (2011)[38]<br>Chia et al. (2005)[43]<br>Ki et al. (2019)[44]*<br>Wong et al. (2004)[45]	Evirgen et al. (2014)[19]<br>Taghrir et al. (2020)[21]<br>Ki et al. (2019)[44]*	N/A

Contact with confirmed cases	De Perio et al. (2012)[14]<br>Pratt et al. (2009)[18]*<br>Shigayeva et al. (2007)[35]<br>Chia et al. (2005)[43]<br>Wong et al. (2004)[45]	Pratt et al. (2009)[18]*<br>Evirgen et al. (2014)[19]	N/A
Workload	Pratt et al. (2009)[18]<br>Chor et al. (2012)[23]*<br>Shigayeva et al. (2007)[35]<br>Vinck et al. (2011)[36]	Chor et al. (2012)[23]*	Chau et al. (2008)[30]<br>Moore et al. (2005)[39]<br>Corley et al. (2010)[41]<br>Yassi et al. (2005)[46]
Monitoring of compliance	Hu et al. (2012)[27]+	N/A	Hu et al. (2012)[27]+<br>Hsu et al. (2011)[42]<br>DiGiovanni et al. (2004)[47]
Patient encounters	De Perio et al. (2012)[14]<br>Shigayeva et al. (2007)[35]	N/A	N/A
Personal protective behaviour-related factors			
Availability of resources	Hu et al. (2012)[27]+<br>Mitchell et al. (2012)[33]*	Mitchell et al. (2012)[33]*	De Perio et al. (2012)[14]<br>Hu et al. (2012)[27]+<br>Moore et al. (2005)[39]<br>Tan et al. (2006)[40]

			Corley et al. (2010)[41]<br>Edeghere et al. (2015)[48]<br>Khalid et al. (2016)[49]<br>Locatelli et al. (2012)[50]<br>Rebmann & Wagner (2009)[51]<br>Rowlands (2007)[52]<br>Van Dijk et al. (2015)[53]<br>Hsu et al. (2006)[62]
Perceived difficulty of protective behaviour	N/A	N/A	Van Dijk et al. (2015)[53]<br>Kang et al. (2018)[54]
Logistical issues	N/A	N/A	Rebmann & Wagner (2009)[51]<br>Kang et al. (2018)[54]<br>Wong et al. (2012)[55]
Perceived effectiveness	Yap et al. (2010)[4]<br>Hu et al. (2012)[27]<br>Mitchell et al. (2012)[33]<br>Parker & Goldman (2006)[34]*	Parker & Goldman (2006)[34]*	Yassi et al. (2005)[46]<br>Kang et al. (2018)[54]
Perceived importance	N/A	N/A	Vinck et al. (2011)[36]<br>Hsu et al. (2011)[42]

Brooks SK, *et al. BMJ Open* 2022; 11:e049857. doi: 10.1136/bmjopen-2021-049857

Incentives	N/A	N/A	Jeong et al. (2011)[22]<br>Hsu et al. (2011)[42]<br>Kang et al. (2018)[54]
Convenience	N/A	N/A	De Perio et al. (2012)[14]<br>Hu et al. (2012)[27]<br>Moore et al. (2005)[39]<br>Tan et al. (2006)[40]<br>Corley et al. (2010)[41]<br>Hsu et al. (2011)[42]<br>Kang et al. (2018)[54]<br>Lam & Hung (2013)[56]<br>Nickell et al. (2004)[57]
Comfort	Mitchell et al. (2012)[33]*	Mitchell et al. (2012)[33]*	Moore et al. (2005)[39]<br>Tan et al. (2006)[40]<br>Corley et al. (2010)[41]<br>Yassi et al. (2005)[46]<br>Khalid et al. (2016)[49]<br>Rowlands (2007)[52]<br>Lam & Hung (2013)[56]<br>Nickell et al. (2004)[57]<br>Kang et al. (2018)[58]

			Khoo et al. (2005)[59]<br>Straus et al. (2004)[60]
Impact on patient care	Hu et al. (2012)[27]+	N/A	Hu et al. (2012)[27]+<br>Tan et al. (2006)[40]<br>Corley et al. (2010)[41]<br>Rowlands (2007)[52]<br>Lam & Hung (2013)[56]<br>Nickell et al. (2004)[57]<br>Khoo et al. (2005)[59]<br>Straus et al. (2004)[60]<br>Tolomiczenko et al. (2005)[61]
Guidance			
Issues with availability or understanding	N/A	N/A	Alsahafi & Cheng (2016)[28]<br>Chau et al. (2008)[30]<br>Moore et al. (2005)[39]<br>Tan et al. (2006)[40]<br>Corley et al. (2010)[41]<br>Yassi et al. (2005)[46]<br>Locatelli et al. (2012)[50]<br>Rebmann & Wagner (2009)[51]<br>Van Dijk et al. (2015)[53]

			Kang et al. (2018)[54]<br>Wong et al. (2012)[55]<br>Lam & Hung (2013)[56]<br>Kang et al. (2018)[58]<br>Tolomiczenko et al. (2005)[61]<br>Hsu et al. (2006)[62]<br>Nhan et al. (2012)[63]<br>Rambaldini et al. (2005)[64]<br>Robertson et al. (2004)[65]
Distress and risk perception			
Distress	Chia et al. (2005)[43]<br>Wong et al. (2004)[45]	N/A	DiGiovanni et al. (2004)[47]
Risk perception	Kim & Choi (2016)[20]<br>Taghrir et al. (2020)[21]<br>Jeong et al. (2011)[22]<br>Chor et al. (2012)[23]*<br>Parker & Goldman (2006)[34]<br>Goulia et al. (2010)[66]	Chor et al. (2012)[23]*	Moore et al. (2005)[39]<br>DiGiovanni et al. (2004)[47]<br>Rebmann & Wagner (2009)[51]
Attitudes and behaviours of others			

Non-compliance of others	N/A	N/A	Hu et al. (2012)[27]<br>Chau et al. (2008)[30]<br>Moore et al. (2005)[39]<br>Hsu et al. (2011)[42]<br>Yassi et al. (2005)[46]<br>DiGiovanni et al. (2004)[47]<br>Locatelli et al. (2012)[50]
Attitudes of family members	N/A	N/A	Moore et al. (2005)[39]<br>Yassi et al. (2005)[46]
Other potential predictors of compliance			
Complacency	N/A	N/A	Tan et al. (2006)[40]
Commitment	N/A	N/A	Alsahafi & Cheng (2016)[28]<br>Moore et al. (2005)[39]
Previous experience with pandemic	N/A	N/A	Moore et al. (2005)[39]
Passing of time	N/A	N/A	Rebmann & Wagner (2009)[51]
Forgetting	Hu et al. (2012)[27]	N/A	N/A
Death of a doctor	N/A	N/A	Tan et al. (2006)[40]

Effort-reward imbalance	Pratt et al. (2009)[18]	N/A	N/A
Consulting with experts	N/A	Manabe et al. (2012)[15]	N/A
Lack of staff	N/A	N/A	Moore et al. (2005)[39]<br>Corley et al. (2010)[41]
PPE fit-testing procedures	N/A	N/A	Moore et al. (2005)[39]
Professionalism	N/A	N/A	Yassi et al. (2005)[46]
Use of public transport	N/A	Evirgen et al. (2014)[19]	N/A
Loss of income	N/A	N/A	DiGiovanni et al. (2004)[47]
Overcrowding	N/A	N/A	Alsahafi & Cheng (2016)[28]

*Appears in both 'significant association' and 'no significant association' column due to both significant and non-significant results being reported for different groups of participants.
+Contains both statistically analysed data and non-statistically analysed (e.g. qualitative data, descriptive survey results).

Supplementary Table III. Summary of evidence

Theme	Evidence
Socio-demographics and personal characteristics	
Age	Al-Amri et al. (2019)[12]: Compliance was not significantly associated with age (p=0.785).
	Datta et al. (2011)[13]: Protective behaviours were not significantly associated with age.
	De Perio et al. (2012)[14]: Compliance was not significantly associated with age.
	Manabe et al. (2012)[15]: For physicians only (other roles not included in analysis), age was not significantly associated with wearing PPE to see suspected avian influenza patients.
	Nour et al. (2015)[16]: There was no significant difference in median protective practice scores between age groups (under 30s v 30 and over) (p=0.582).
	Nour et al. (2017)[17]: There was no significant difference in median protective practice scores between age groups (under 30s v 30 and over).
	Pratt et al. (2009)[18]: Age was not significantly associated with compliance.
	Kim & Choi (2016)[20]: Age was significantly positively correlated with preventive behaviours (p<0.05); older staff were more likely to comply.
Gender	Al-Amri et al. (2019)[12]:Compliance was not significantly associated with gender (p=0.241).
	Datta et al. (2011)[13]: Protective behaviours were not significantly associated with gender.
	DePerio et al. (2012)[14]: Compliance was not significantly associated with gender.

	Nour et al. (2015)[16]: Female staff were significantly more likely to comply with preventive behaviours (p<0.001).
	Nour et al. (2017)[17]: There was no significant difference in median protective practice scores between genders.
	Evirgen et al. (2014)[19]: Behaviour was not significantly associated with gender.
	Kim & Choi (2016)[20]: Gender was not significantly associated with preventive behaviours.
	Taghrir et al. (2020)[21]: There was no significant association between preventive behaviours and gender.
	Jeong et al. (2011)[22]: Female gender was a significant predictor of preventive behaviours.
	Vinck et al. (2011)[36]: Female staff reported higher compliance than males (p=0.002) and greater use of PPE (p=0.037).
Nationality	Al-Amri et al. (2019)[12]:Compliance was significantly associated with nationality (p=0.047), with Saudi staff showing higher compliance than non-Saudi staff in the same city.
Country	Chor et al. (2012)[23]: Hong Kong reported the highest compliance with all infection control practices, except for wearing a mask during patient contact, which was second to Singapore (70.4% v 87.7%). Wearing masks in clinical areas while not involved with patient contact was lowest for the UK (25.3% v 96.4% for Hong Kong and 82.3% for Singapore), as was use of masks (either surgical or N95) during physical contact with patients (62% for the UK v 70.4% for Hong Kong and 87.7% for Singapore).
	Koh et al. (2009)[24]: Healthcare workers in Singapore were significantly more likely than those in Indonesia to screen patients and clinic visitors for fever (92.9% v 80.8%, p<0.001), limit number of visitors to the clinic (88.7% v 46.0%, p<0.001), post prominent infection control notices to patients/visitors (97.4% v 80.3%, p<0.001), do temperature checks for staff (91.5% v 61.1%, p<0.001); wear gowns (91.1% v 61.5%, p<0.001); wear paper masks (43.2% v 11.7%, p<0.001); wear surgical masks (28.6% v 12.6%, p<0.001); wear N95 masks (94.0% v 82.4%, p=0.004); and carry out regular handwashing (98.6% v 87.0%, p<0.001). However, there were no significant differences between countries in wearing gloves (94.2% v 93.3%, p=0.502); wearing goggles (77.6% v 67.8%, p=0.140); using powered air purifier

	respirators (86.1% v 85.4%, p=0.550); using alcohol hand rub (95.6% v 90.8%, p=0.183); or using a special room to isolate bird flu patients (97.9% v 97.5%, p=0.254).
	Wong et al. (2005)[25]: Physicians in Hong Kong were less likely than those in Canada to quarantine themselves after contact with a probable or suspected SARS case (19.4% v 77.1%, p<0.01) or give quarantine leave to their staff (59.7% v 95%, p<0.01). However, physicians in Hong Kong were more likely than those in Canada to test patient temperature as a routine procedure (68.1% v 47.1%, p<0.01), wear a mask during consultations (97.7% v 52.9%, p<0.01) and have their support staff wear masks (97.8% v 68.6%, p<0.01). No significant differences between countries were found for wearing gowns for every patient encounter or washing hands between every encounter.
	Kamate et al. (2020)[26]: There were no significant differences between countries in terms of discussing risk of COVID-19 with patients; discussing preventive measures with patients; showing patients the signs and symptoms of COVIF-19; or taking preventive measures themselves. However, there was a significant difference in whether they included travel history while recording patients' histories (99.0% in Asia v 97.2% in Americas v 94.3% in Europe v 94.3% in Africa v 89.4% in Australia/Antarctica, p=0.025).
Religion	Kim & Choi (2016)[20]: Being religious or not was not significantly associated with preventive behaviours.
Socio-economic status	Jeong et al. (2011)[22]: 'High or middle economic state' significantly predicted preventive behaviours.
Marital status	Pratt et al. (2009)[18]: Marital status was not significantly associated with compliance.
	Evirgen et al. (2014)[19]: Behaviour was not significantly associated with marital status.

Level of education	Al-Amri et al. (2019)[12]: Compliance was significantly associated with qualifications (p=0.037) with more highly-qualified staff being more likely to comply. Percentage of poor, good and excellent preventive practices, by qualification: MBBS: 30.6%, 69.4%, 0.0%; Diploma: 46.7%, 46.7%, 6.7%; Masters: 43.8%, 43.8%, 12.5%; Doctorate/fellowship: 66.7%, 22.2%, 11.1%.
	Datta et al. (2011)[13]: Protective behaviours were not significantly associated with level of education.
	Pratt et al. (2009)[18]: Highest level of education was not significantly associated with compliance.
	Kim & Choi (2016)[20]: Level of education (junior/senior grade) was not significantly associated with compliance.
	Taghrir et al. (2020)[21]: There was no significant association between preventive behaviours and education level.
Personal vulnerabilities	Evirgen et al. (2014)[19]: Behaviour was not significantly associated with being pregnant or having a chronic disease.
Vulnerable people at home	Evirgen et al. (2014)[19]: Behaviour was not significantly associated with having a pregnant spouse, elderly person, or school-aged child at home. However, staff with babies at home were more likely to comply with protective behaviours (p<0.005).
Vaccination status	Hu et al. (2012)[27]: Vaccination for H1N1 influenza was an independent predictor of high compliance to PPE (p<0.001).
Occupational role	
Role	Al-Amri et al. (2019)[12]: Practice-related adherence to guidelines differed significantly according to position (p=0.035), with specialists having the highest percentage of excellent practice.
	Datta et al. (2011)[13]: Protective behaviours were not significantly associated with occupation.
	Nour et al. (2015)[16]: There was no significant difference in median protective practice scores between occupational groups (p=0.433).

	Nour et al. (2017)[17]: There was no significant difference in median protective practice scores between occupational groups (nurses v physicians v specialists v technicians).
	Pratt et al. (2009)[18]: Job status (full-time, part-time or casual) was reported to be significantly associated with compliance although the authors do not state which group were more likely to comply. Medical/surgical unit as a primary area of practice (compared to maternal/newborn or paediatrics, emergency or critical care, or 'other' roles) was also reported to be significantly associated with compliance but again the authors do not state in which way.
	Evirgen et al. (2014)[19]: Professors had the highest average score for positive behaviours.
	Chor et al. (2012)[23]: In Hong Kong and Singapore, employment type was the strongest predictor of adoption of infection control practices, with nurses showing significantly higher compliance with most measures compared to doctors in both countries, and allied healthcare workers also reporting significantly higher compliance with most measures compared to doctors in Hong Kong. Administrative staff in Hong Kong reported compliance similar to that of other healthcare workers, whereas administrative staff in the UK reported significantly lower compliance than doctors.
	Alsahafi & Cheng (2016)[28]: There appeared to be significant differences between physicians, nurses and other healthcare staff in terms of handwashing after patient contact (p=0.04), wearing of surgical mask during patient contact (p<0.001), and wearing of N95 mask during patient contact (p=0.009). It is not clear from the data presented which groups are significantly more likely to comply, but perhaps nurses are slightly more likely to comply than physicians who are slightly more likely to comply than other healthcare staff.
	Alsubaie et al. (2019)[29]: Non-physicians were significantly more likely than physicians to report improvement (that is, improvement since pre-outbreak) in hand hygiene compliance at the hospital (90% v 80%, p<0.002), improvement in compliance with universal precautions at the hospital (85.5% v 69%, p=0.001), increase in avoidance behaviour towards people with flu-like symptoms (83% v 72%, p<0.009), decrease in using public facilities (66% v 28.7%,

	p=0.001), decrease in hand-shaking (60% v 27.7%, p<0.001), and decrease in social visits e.g. to friends (60% v 21.7%, p<0.001).
	Chau et al. (2008)[30]: Nurses performed better in a number of isolation precautions (including hand-washing, hand rub use, glove use, handling of linen and laundry, and handling of patient care equipment) than other roles. Doctors performed better when it came to wearing masks and putting on gowns.
	Koh et al. (2005)[31]: All other occupational groups, including nurses (OR 0.49; 95% CI 0.39-0.61) reported greater difficulty adhering to preventive measures compared with doctors.
	May et al. (2010)[32]: Medical students appeared more likely than residents to use hand sanitiser both at work and home, wash hands both at work and home, and practice cough etiquette at work and home, while residents appeared more likely to use disinfectant and use surgical masks and N95 masks when seeing patients with influenza-like illness – however, no statistical analysis is presented.
	Mitchell et al. (2012)[33]: Compared with physicians, nurses and respiratory therapists were significantly more compliant with wearing N95 respirators and gloves for aerosol-generating medical procedures and with wearing protective eyewear and gloves for patient care not involving these procedures. Nurses and respiratory therapists were also significantly more compliant with performing a user seal check after donning an N95 respirator than physicians (35% and 40% v 19%, p=0.023).
	Parker & Goldman (2006)[34]: Physicians reported more handwashing before and after all patient contacts compared with nurses and trainees (p<0.05); however, there were no significant differences between in occupational groups in compliance with wearing an N95 mask when examining patients, wearing a gown when examining patients, wearing an N95 mask at all times in the emergency department, wearing eye protection when examining patients, or wearing gloves when examining patients.

	Shigayeva et al. (2007)[35]: Clinically trained health professional status was reported to be a significant predictor of consistent adherence (statistics not reported). Safe removal of PPE was significantly associated with being a nurse (OR 1.3, 95% CI, 1.1-1.6).
	Vinck et al. (2011)[36]: Public health physicians followed the case definition less strictly than public health nurses (p=0.000).
	Alshammari et al. (2018)[37]: No significant difference in hand hygiene compliance between physicians and nurses.
	Jaeger et al. (2011)[38]: Use of PPE stratified by healthcare role did not yield significant differences.
	Chia et al. (2005)[43]: The risk of using a less effective level of respiratory protection was lowest among doctors, followed by nurses and clerical staff.
Length of experience	Al-Amri et al. (2019)[12]:Compliance was not significantly associated with length of experience in primary care (p=0.265).
	Nour et al. (2015)[16]: There was no significant association between median protective practice scores and years of experience (p=0.674).
	Nour et al. (2017)[17]: There was no significant association between median protective practice scores and years of experience.
	Pratt et al. (2009)[18]: Years of nursing experience were not significantly associated with compliance.
	Chau et al. (2008)[30]: Compliance was not significantly associated with years of experience.
	Koh et al. (2005)[31]: The longer the work experience, the less likely staff were to experience difficulty in adhering to preventive measures (OR 1.21; 95% CI 1.13-1.29).

	Mitchell et al. (2012)[33]: Staff who had worked in health care for more than eleven years were significantly more compliant with wearing protective eyewear during aerosol-generating medical procedures than those who had worked for eleven years or less (69% v 54%, p<0.001), and also with wearing gowns (82% v 69%, p<0.001), gloves (92% v 85%, p=0.006) and N95 respirators (75% v 60%, p<0.001). For patient care not involving aerosol-generating medical procedures, staff with more than eleven years of experience were again significantly more likely to comply with wearing protective eyewear (38% v 31%, p=0.04), gowns (82% v 69%, p<0.001), N95 respirators (43% v 31%, p=0.003) and surgical masks (18% v 11%, p<0.016).
	Vinck et al. (2011)[36]: Compliance was higher in those who had been working in the public health service for one to ten years than those who had worked for eleven years or longer (p=0.034).
Training and knowledge	
Receiving training/education	Nour et al. (2017)[17]: After the training intervention in the study, there was a non-significant increase in preventive practices (p=0.168) – but compliance was good pre-intervention too.
	Taghrir et al. (2020)[21]: There was no significant association between preventive behaviours and having received education on COVID-19.
	Alsahafi & Cheng (2016)[28]: 22.8% of participants reported having received training about dealing with infectious disease outbreaks, 37.1% training in infection control policies and procedures, 54.4% training in hand hygiene and 45.6% training in N95 mask-wearing techniques. A high proportion of participants believed that insufficient training in infection control procedures contributed to poor compliance and the risk of infection (92.4% of nurses, 91% of physicians and 90.9% of other healthcare staff).

	May et al. (2010)[32]: There was a significant association between level of training and use of currently recommended CDC guidelines for patients with influenza-like illness, for fellows only (p=0.003); however, the number of fellows within the sample was small (n=5).
	Shigayeva et al. (2007)[35]: Recent infection control training was a significant predictor of consistent adherence to recommended behaviours (interactive training: OR 2.7, 95% CI, 1.7-4.4; passive training: OR 1.7, 95% CI, 1.0-3.0).
	Moore et al. (2005)[39]: Many participants reported having received poor prior training in infection control and therefore having to use new procedures and equipment without training or experience.
	Tan et al. (2006)[40]: Participants reported that little information on the disease or methods of control were available from previous education and training, which made them feel helpless.
	Corley et al. (2010)[41]: Participants believed that an infection control course with yearly refreshers would be useful and help improve compliance.
Sources of knowledge	Al-Amri et al. (2019)[12]: Those who attended CME activities had significantly higher levels of excellent practice than those who did not attend a CME activity (11.1% vs 1.5%, p=0.002). Participants who had textbooks as a source of knowledge had the highest percent of excellent practice (p<0.001).
	Kim & Choi (2016)[20]: Source of knowledge of MERS (television, or other) was not significantly associated with preventive behaviours.
	Taghrir et al. (2020)[21]: There was no significant association between preventive behaviours and source of information (WHO, CDC, or UpToDate v national guidelines v both v others).
Knowledge (of correct practices / outbreak)	Yap et al. (2010)[4]: There was a significant positive correlation between knowledge about H1N1/preventive behaviours, and practice of preventive behaviours (p<0.01).
	Al-Amri et al. (2019)[12]: Practice-related adherence to guidelines was not significantly associated with knowledge.

	De Perio et al. (2012)[14]: Knowledge of correct PPE was significantly associated with compliance at one hospital (p=0.047) but not the other three hospitals in the study.
	Nour et al. (2015)[16]: There was a significant positive association between knowledge and practice (p<0.001).
	Kim & Choi (2016)[20]: Having received MERS education was not significantly associated with preventive behaviours (p=0.209). However, in a correlational analysis, preventive behaviours were significantly correlated with MERS-related knowledge (p<0.01).
	Jeong et al. (2011)[22]: A score for 'cues to action' (consisting of consulting news or articles about the pandemic, being interested in methods for health promotion, and wishing to be educated on effective ways of preventing infection) was significantly associated with compliance (OR 2.63, CI 1.81-3.81).
	Hu et al. (2012)[27]: Self-reported adequate knowledge of self-protection and patient protection was significantly correlated with the perception of further improvement of PPE compliance (p<0.001).
	Alsahafi & Cheng (2016)[28]: A high proportion of participants believed that lack of knowledge about the mode of transmission contributed to poor compliance (94.2% of nurses, 90% of physicians and 90.2% of other healthcare staff).
	Hsu et al. (2011)[42]: 1.9% of participants believed lack of education about correct practices prevented hand hygiene compliance. 6.5% of participants believed improving undergraduate education efforts was the most important strategy for improving hand hygiene compliance. 7.3% of participants believed incorporating hand hygiene into examinations and the student internship marking scheme was the most important strategy.
Work-related factors	
Setting	De Perio et al. (2012)[14]: Staff who worked on an intensive care unit rotation during the study period were more likely to be in the 'high adherence' group (OR, 3.43; 95% CI, 1.02-11.56). Staff who worked on an inpatient ward rotation were less likely to be classified as high adherence (OR, 0.22, 95% CI, 0.06-0.80).

	Evirgen et al. (2014)[19]: Behaviour was not significantly associated with department at work or the department where the participant spends the most time.
	Taghrir et al. (2020)[21]: There was no significant association between preventive behaviours and ward worked on.
	Hu et al. (2012)[27]: Compliance rates significantly differed between institutions, from 0% to 88.1%, although all institutions were adult intensive care units.
	Chau et al. (2008)[30]: Those working in rehabilitation hospitals were significantly more likely to comply with protective behaviours overall, and use hand rub, than participants working in acute hospitals.
	Mitchell et al. (2012)[33]: Staff working in an intensive care unit or designated influenza ward were more compliant with wearing an N95 respirator for aerosol-generating medical procedures than those working in an emergency department (78% and 67% v 47%, p<0.001).
	Shigayeva et al. (2007)[35]: Working in a SARS unit was significantly associated with consistent adherence to recommended guidelines (OR 4.0, 95% CI, 1.8-8.9), as was working in an intensive care unit (OR 4.3, 95% CI, 2.0-9.0). Employment in a teaching hospital (OR 7.6, 95% CI, 4.8-11.9), SARS unit (OR 3.4, 95% CI, 1.3-9.4) or an intensive care unit (OR 1.9, 95% CI, 0.7-5.0) were associated with safer removal of PPE.
	Jaeger et al. (2011)[38]: 68% of staff in outpatient settings reported no PPE use with index patients, compared to 9% of staff in inpatient setting (p<0.01). Mask or N95 respirator use during index patient contact was more common in inpatient (41%) than outpatient settings (5%) (p<0.01). Glove use was more common among inpatient (57%) than outpatient (21%) settings. Always donning gloves, gown and either mask or N95 respirator was more common in inpatient (18%) than outpatient (5%) (p<0.01).
	Chia et al. (2005)[43]: Using administrative staff as the reference group, individuals working in surgical, medical and intensive care units were more likely to wear the appropriate respiratory protection. However, there was no significant

	difference for those individuals in the other areas (A&E, radiographic services, laboratory services, and others) compared to administrative personnel.
	Ki et al. (2019)[44]: Staff working in the emergency department were significantly more likely than staff in general wards to wear surgical masks (93.0% v 1.8%, p<0.001); however, there were no significant differences in hand-washing or wearing gloves.
	Wong et al. (2004)[45]: Staff working in high-infection districts were significantly more likely to wear gowns (63.0% v 42.0%, p<0.05), wash their hands before going home (82.6% v 63.0%, p<0.05) and use disinfectants to clean surfaces regularly (87.0% v 65.4%, p<0.01); however, they were less likely to quarantine themselves (6.5% v 27.5%, p<0.01) or grant leave to staff until confirmation of diagnosis (41.3% v 71.3%, p<0.01). Staff in public clinics were significantly more likely to wear gowns during consultations than staff in private clinics (81.3% v 45.3%, p<0.05); however, staff in private clinics were more likely to quarantine themselves for a 10-day period after SARS contact than their public sector colleagues (58.1% v 31.3%, p<0.05). Staff in the public sector were more likely to stay away from home.
Contact with confirmed cases	De Perio et al. (2012)[14]: Staff who were present at an aerosol-generating procedure on a patient with pH1N1 (OR 5.20, 95% CI, 1.54-17.55) were more likely to be in the high adherence group.
	Pratt et al. (2009)[18]: Nurses' experience of working directly with confirmed SARS cases was a significant predictor of compliance (p<0.001), with nurses who worked with SARS patients more likely to comply than those who did not. However, this was no longer significant after combining the impact of effort-reward imbalance and burnout.
	Evirgen et al. (2014)[19]: Behaviour was not significantly associated with awareness of suspected or confirmed H1N1 infection in their hospital or their department, or having contact with H1N1 cases.
	Shigayeva et al. (2007)[35]: Recognition of the patient as a SARS case was a significant predictor of consistent adherence to recommended barrier precautions (OR 2.5, 95% CI, 1.5-4.5).

	Chia et al. (2005)[43]: Staff who regularly came into contact with SARS patients were more likely to wear appropriate respiratory protection compared to those who did not (OR 0.673, 95% CI, 0.587-0.771).
	Wong et al. (2004)[45]: SARS-exposed staff were significantly more likely to insist on patients wearing masks (65.1% v 48.1%, p<0.05) and more likely to ensure their staff wore gloves and masks all the time (33.7% v 11.5%, p<0.01 for gloves, 100% v 92.3%, p<0.01 for masks). However, they were also less likely to quarantine themselves until confirmation of diagnosis (10.8% v 33.3%, p<0.01).
Workload	Pratt et al. (2009)[18]: Hours worked per week were reportedly significantly associated with compliance; however, the authors do not give details about whether more or less hours are associated with greater or lower compliance.
	Chor et al. (2012)[23]: There was a significant decrease in compliance with handwashing before patient contact with the increase in number of weekly patient contacts only in Hong Kong; no significant difference was noted in Singapore or the UK.
	Chau et al. (2008)[30]: Workload was a common barrier to compliance identified by participants.
	Shigayeva et al. (2007)[35]: Work on shifts requiring more frequent patient room entry was associated with lower compliance (OR for 6 or more entries per shift: 0.5, 95% CI, 0.32-0.86). The reported rate of consistent adherence was lower for staff members who reported entering a patient's room more than 5 times per shift: consistent adherence was reported for 84.1% of procedures involving staff members who reported entering the room 1-5 times, compared with 75.5% of procedures involving staff members who reported entering the room 6 or more times (p<0.001).
	Vinck et al. (2011)[36]: The compliance to measures to properly identify suspected cases of respondents who regularly worked overtime was reduced compared with those who did not (p=0.024). Conversely, working overtime was associated with increased compliance with informing patients that they were not supposed to leave their home while ill (p=0.048) and providing patients with the information leaflet (p=0.002). Additionally, compliance with control

	measures was not influenced by the number of inhabitants within the catchment area of the public health service or by the number of cases for which consultation within the centralised assessment system of each public health service with the Preparedness and Response Unit was carried out (objective workload).
	Moore et al. (2005)[39]: Fatigue was cited as a major cause of failing to follow the proper guidelines, with exhaustion leading to 'sloppy practice'.
	Corley et al. (2010)[41]: Isolation procedures and preventive behaviours created extra workload, frustration and confusion - educating and monitoring external staff and patient's relatives regarding PPE was an extra duty for already overstretched staff and was time-consuming and frustrating.
	Yassi et al. (2005)[46]: The increased time constraints and increased workload were felt to be important barriers to worker adherence to recommendations.
Monitoring of compliance	Hu et al. (2012)[27]: Significantly more nurses than physicians (92.1% v 86.4%, p=0.020) reported being reprimanded by their supervisor for non-compliance. Perceived reprimand by supervisor for non-compliance was a significant predictor of compliance (p=0.035).
	Hsu et al. (2011)[42]: 4.1% of participants believed better policing by infection control staff was the most important strategy for improving hand hygiene, and 3.3% thought that improving public awareness (patients monitoring doctors) was the most important.
	DiGiovanni et al. (2004)[47]: Public health authorities' monitoring of compliance played an important role in establishing the credibility of quarantine for 75% of physicians, 81% of nurses, and 85% of other healthcare workers: however, 58% of physicians, 37% of nurses and 40% of other staff rated the monitoring of their own compliance as bad.

Patient encounters	De Perio et al. (2012)[14]: 55% of those who reported not using the recommended PPE (n=40) stated it was because they did not know the patient had H1N1 or an influenza-like illness; 20% stated they did not think PPE was needed for the particular activity they were doing; 18% stated it was because the only entered the patients' room for a brief time; 13% stated it was because they did not touch the patient and 10% stated it was because they did not come within six feet of the patient.
	Shigayeva et al. (2007)[35]: Lower rates of consistent adherence were found among staff providing care for patients with higher Acute Physiology and Chronic Health Evaluation (APACHE) II scores – i.e. more severe illness (OR for score APACHE II of 20 or greater: 0.4, 95% CI, 0.28-0.68). Reported rates of consistent adherence were higher when healthcare workers were themselves performing procedures (adjusted OR: 1.7, 95% CI, 1.4-2.2) or assisting with them (adjusted OR: 1.5, 95% CI, 1.1-2.1), compared with procedures that they observed (81% v 79.1% v 74.2%, p<0.001).
Personal protective behaviour-related factors	
Availability of resources	De Perio et al. (2012)[14]: 18% of those who reported not using the recommended PPE (n=40) stated it was because their facility had run out of PPE, while 10% reported that their facility did not provide PPE.
	Hu et al. (2012)[27]: 63.2% reported that appropriate PPE was readily available. Availability of PPE in the ICU was significantly associated with compliance.
	Mitchell et al. (2012)[33]: Staff were significantly more compliant with PPE use when eyewear and gloves were readily available at the point of care; however, PPE availability did not increase compliance with the use of N95 respirators, surgical masks, or gowns.
	Moore et al. (2005)[39]: Participants reported supply problems with face shields and goggles.

	Tan et al. (2006)[40]: Many participants reported that they had not been properly mask-fitted due to lack of proper testing equipment in their clinic. Many were not provided with gowns. Several participants reported lack of goggles and visual shields in their clinics.
	Corley et al. (2010)[41]: Participants believed there were not enough PPE supplies and that guidelines changed depending on supplies running out – for example, surgical masks and plastic aprons became acceptable due to the lack of other PPE. One staff member reported wearing an incorrectly sized mask as their correct size was not in stock.
	Edeghere et al. (2015)[48]: Only 20% of participants experienced barriers in applying infection control measures; the main reported barrier was the occasional unavailability of recommended PPE, most often eye protection.
	Khalid et al. (2016)[49]: 60% perceived inadequate protective measures available to them and cited this as a stressor.
	Locatelli et al. (2012)[50]: Participants reported that stocks of N95 masks ran low so they started using surgical masks, which made it difficult to enforce the use of N95s.
	Rebmann & Wagner (2009)[51]: Many participants reported a lack of resources. Less than half of those whose facility had requested supplies from external agencies actually received them, whereas three participants whose facilities had not requested supplies did receive them. Many reported that supplies were back-ordered and they could not receive them in a timely manner. Theft of supplies was also an issue, with masks and respirators presumably being used or taken by visitors. 26.5% said their facility ran out of respiratory protection. Many reported staff in their facilities were wearing the wrong sizes as they were unable to get the correct sizes.
	Rowlands (2007)[52]: Participants reported that there were not enough masks for staff.
	Van Dijk et al. (2015)[53]: 73.5% believed they had sufficient personal protective materials; 26.5% believed they were insufficient.
	Hsu et al. (2006)[62]: 44% expressed dissatisfaction with shortage of protective equipment such as N95 masks and gloves.

Perceived difficulty of protective behaviour	Van Dijk et al. (2015)[53]: 35.0% believed recommended personal protective measures were feasible.
	Kang et al. (2018)[54]: Participants reported difficulties with the complexity of using several PPE items together.
Logistical issues	Rebmann & Wagner (2009)[51]: Participants reported logistical issues with needing to fit-test large numbers of staff in a short time.
	Kang et al. (2018)[54]: Limited hospital layouts disrupted space management for PPE use.
	Wong et al. (2012)[55]: Barriers to PPE use included inadequate shower facilities for staff, the small size of the changing room, and the physical layout of the two-bed isolation room which was poor for infectious case management.
Perceived effectiveness	Yap et al. (2010)[4]: There was a significant correlation between attitudes (an 'attitude' score was based on responses to questions on attitudes towards the use of PPE, vaccination, Tamiflu, help-seeking and risk perception) (r=0.24, p<0.01); there was no such correlation for cases or close contacts who answered the same questions.
	Hu et al. (2012)[27]: Positive attitude towards PPE use (i.e. belief that it provided adequate protection) significantly predicted compliance (p=0.001).
	Mitchell et al. (2012)[33]: Staff who felt better protected against influenza when they wore an N95 respirator were significantly more compliant with N95 respirator use than those who did not (p<0.001 for aerosol-generating medical procedures and p=0.04 for patient care not involving these procedures).
	Parker & Goldman (2006)[34]: Staff considering the combination of all infection control measures to be highly effective in protecting patients from SARS reported increased use of a mask when examining patients compared with staff considering all measures less effective (p=0.05). However, handwashing, glove use, gown use, eye protection use, and wearing a mask at all times in the emergency department did not significantly differ between the two groups.

	Yassi et al. (2005)[46]: Beliefs in the effectiveness of infection control guidelines, as modified by past experiences, were identified as having important influence on worker adherence to procedures.
	Kang et al. (2018)[54]: Participants expressed doubts about the quality and effectiveness of PPE.
Perceived importance	Vinck et al. (2011)[36]: Compliance with the recommendation to consult the Preparedness and Response Unit for centralised assessment of patients was fairly low, with the main reasons for non-compliance being finding it unnecessary (38.3%) or time-consuming (22.7%).
	Hsu et al. (2011)[42]: 20.1% believed that failure to recognise the importance of hand hygiene prevented compliance
Incentives	Jeong et al. (2011)[22]: Perceived benefits of the preventive behaviours significantly predicted higher compliance (OR 3.18, CI 2.15-4.68).
	Hsu et al. (2011)[42]: 19.9% of participants believed that providing positive incentives for hand hygiene was the single most important factor in improving compliance.
	Kang et al. (2018)[54]: Participants reported a lack of incentives to comply with preventive behaviours.
Convenience	De Perio et al. (2012)[14]: 30% of those who reported not using the recommended PPE (n=40) stated it was because the PPE was not available near their patients' room; 10% felt too busy to wear PPE; and 10% stated it was inconvenient to use.
	Hu et al. (2012)[27]: Half of participants reported that PPE use was inconvenient.
	Moore et al. (2005)[39]: Some participants reported that PPE was time-consuming due to having to constantly gown, de-gown, and re-gown.
	Tan et al. (2006)[40]: Participants reported that gowns were inconvenient during toilet breaks.

	Corley et al. (2010)[41]: Application and removal of PPE was reported to be time-consuming.
	Hsu et al. (2011)[42]: 60.4% believed lack of time prevented hand hygiene compliance.
	Kang et al. (2018)[54]: PPE use was inconvenient in the presence of hospital visitors.
	Lam & Hung (2013)[56]: Participants reported that gowning and de-gowning was very time-consuming.
	Nickell et al. (2004)[57]: 41.7% of participants felt precautionary measures affected their ability to do their job.
Comfort	Mitchell et al. (2012)[33]: Staff who reported always or often feeling comfortable wearing protective eyewear were significantly more likely to wear it ($p<0.001$). Similarly, staff who reported always or often feeling comfortable wearing an N95 respirator were significantly more compliant with N95 use. Less than 19% of staff reported always or often feeling short of breath, claustrophobic or dizzy when wearing protective eyewear or N95 respirators; no significant differences in compliance were observed between these staff and staff who reported never or rarely feeling short of breath, claustrophobic or dizzy.
	Moore et al. (2005)[39]: Some participants reported that PPE was uncomfortable.
	Tan et al. (2006)[40]: Participants reported physical discomfort from prolonged use of N95 masks, including difficulty breathing, headache, and development of allergic facial rash; they reported moments of complacency where they removed masks intermittently in order to allow normal breathing.
	Corley et al. (2010)[41]: Wearing PPE for extended periods was difficult as it was uncomfortable and could cause dehydration and injuries such as skin peeling.
	Yassi et al. (2005)[46]: Participants identified the need to address the increased amount of worker fatigue that existed when staff worked with full PPE, noting that the discomfort associated with wearing PPE was an important barriers to worker adherence to recommendations.

	Khalid et al. (2016)[49]: Participants reported that having to wear PPE was stressful.
	Rowlands (2007)[52]: Participants reported that masks were warm and uncomfortable, often leading them to develop headaches or skin rashes, and made it hard to breathe.
	Kang et al. (2018)[54]: Many participants reported having ill-fitted PPE.
	Lam & Hung (2013)[56]: Participants reported that PPE was uncomfortable.
	Nickell et al. (2004)[57]: 85.4% of participants found masks bothersome; of these, 92.9% stated they caused physical discomfort and 13.0% said they gave them a sense of isolation.
	Kang et al. (2018)[58]: Participants reported finding it stressful that their masks did not fit properly and emphasised the discomfort of PPE: they found it hard to breathe or see clearly while wearing it, which made their work harder, and it made them sweaty and dizzy when they wore it for too long.
	Khoo et al. (2005)[59]: Participant opinions on 3M powered air-purifying respirator: 2.3% found it uncomfortable, 11.6% reported moderate discomfort, 32.6% found it tolerable, 30.2% reported slight discomfort, and 23.3% found it very comfortable. 2.3% reported unacceptable visual impairment. 2.3% felt it was suffocating, 4.6% found breathing uncomfortable, 7.0% reported it was comfortable but moderately hard to breathe, 60.5% found it comfortable with minimal difficulty breathing, and 25.6% reported no breathing discomfort. Compared to Stryker powered air-purifying respirator: 4.5% found it uncomfortable, 4.5% reported moderate discomfort, 13.7% found it tolerable, 31.8% reported slight discomfort, and 45.5% found it very comfortable. 4.5% reported unacceptable visual impairment. 0% felt suffocated.
	Straus et al. (2004)[60]: Participants reported that masks hurt, made them sweat and gave them headaches.

Impact on patient care	Hu et al. (2012)[27]: 21.2% believed that PPE use would interfere with patient care (no significant difference between nurses and physicians). The perception that PPE use would interfere with patient care was significantly associated with poor compliance (p=0.002).
	Tan et al. (2006)[40]: Staff reported that masks muffled their speech and they had to raise their voices to communicate with patients. They also reported that patient reactions to PPE ranged from amusement to apprehension and anxiety; some saw their doctor wearing PPE and assumed they had been exposed to SARS and could infect them and their families.
	Corley et al. (2010)[41]: Wearing PPE was reported to make communication difficult.
	Rowlands (2007)[52]: Participants reported that masks impacted the patient counselling process as they compromised their communication skills and made their speech muffled. They also believed that psychiatric patients with paranoia were sensitive to masks.
	Lam & Hung (2013)[56]: Participants reported that PPE affected their communication with patients, making them have to speak very loudly in order to be heard.
	Nickell et al. (2004)[57]: 85.4% of participants found masks bothersome; of these, 47.0% stated that they caused difficulties communicating.
	Khoo et al. (2005)[59]: Some difficulties regarding communication. Opinions on the 3M powered air-purifying respirator: 0% felt they had to shout but 25.6% felt they had to raise their voice significantly, 37.2% had to raise voice moderately, 32.6% raised voice normally, and 4.6% could speak normally. 2.3% said it caused unacceptable hearing impairment, 11.6% significant, 27.9% moderate, 41.9% mild and 16.3% hearing impairment. Compared to the Stryker powered air-purifying respirator: 0% had to shout, 27.3% raised voice significantly, 31.8% raised voice moderately, 27.3% raised voice mildly, and 13.7% could speak normally. 0% reported unacceptable hearing impairment. About two-thirds of

	respondents agreed (22%) or strongly agreed (42%) that they looked frightening to their patients whenever they used the PAPR.
	Straus et al. (2004)[60]: Participants reported that wearing protective equipment made assessing and communicating with patients a challenge, as they could not establish any of the usual non-verbal cues with patients. They were concerned that patients may have been seen less frequently than usual because of the time required to get into protective clothing.
	Tolomiczenko et al. (2005)[61]: Nurses, more than other occupational groups, reported that infection control measures had an adverse impact on patient care because they were less 'visible' to their patients when gowned, masked and gloved.
Guidance	
Issues with availability or understanding	Alsahafi & Cheng (2016)[28]: Almost two thirds of participants were unaware of guidelines or protocols for the care of infected patients. A high proportion believed that compliance was negatively affected by the lack of policies and procedures (87.3% of nurses, 85% of doctors, 89.4% of other healthcare staff).
	Chau et al. (2008)[30]: Issues with communication and understanding were cited as barriers to compliance. Participants expressed mixed views on the guidelines; some described them as easy to follow and facilitating increased alertness, while others found them difficult to follow and too long (and suggested they be given summaries instead, to get a basic idea of the principles). Other suggestions included tailored information; more programmes in Chinese with a more interactive approach to learning; making courses more practical; and educating the public and patients too.
	Moore et al. (2005)[39]: Participants reported a lack of consistency with safety instructions and frequently changing directives. New levels of enforcement by regulatory agencies interfered with rational infection control practice and infection control policies developed elsewhere had little perceived relevance to their own workplace. Basic infection control policies and procedures were frequently perceived as not well-developed. A major problem was perceived to be

Brooks SK, *et al. BMJ Open* 2022; 11:e049857. doi: 10.1136/bmjopen-2021-049857

	issues with communicating the guidelines: participants reported it was difficult to disseminate information simultaneously to all staff; often new information was sent by email, which was not checked before going to work.
	Tan et al. (2006)[40]: Some participants reported not wearing gowns or gloves due to inadequate dissemination of information/instructions from health authorities. However, a SARS seminar and hotline were organised which were perceived to be an effective channel of communication to address concerns and queries on PPE.
	Corley et al. (2010)[41]: Participants perceived a lack of firm recommendations/guidelines regarding what specific PPE was required, which created confusion and made them feel unprotected. They also remarked that current guidelines changed too frequently to keep up. Participants also perceived a lack of clear guidelines and conflicting advice about what to do when a patient is deemed non-infectious.
	Yassi et al. (2005)[46]: To improve adherence to infection control guidelines, participants felt that better enforcement of infection control guidelines was needed but did not want such enforcement to rely on nurses "policing" other professionals.
	Locatelli et al. (2012)[50]: Participants reported many barriers to effective communication about infection control, including information overload, often contradictory information from different sources, and difficulty integrating information from various sources and working out what was a priority. 10 participants reported not having a pre-defined communication plan, or discovered flaws with their plan. Some reported that everyone had to develop their own guidelines, with no clear guidance or resources on how to do so provided. This resulted in confusion about the use of PPE.
	Rebmann & Wagner (2009)[51]: Participants reported a lack of guidance for alternate care sites and non-acute care settings such as physician offices, ambulatory care clinics, long-term care and nursing homes, as well as a general lack of infection prevention information and guidance related to occupational health issues. Rapidly-changing practice

	recommendations were often contradictory which made it difficult to maintain staff compliance and trust, and to stay abreast of the latest recommendations. There was a perceived lack of consistency between sources, with CDC, state health departments, and city health departments all saying different things. Participants reported gaps in communication between departments, or between hospitals and physician offices. Many did not know which type of mask or respirator to use when caring for H1N1 patients, and reported conflicting guidelines related to isolation, masks, and respirator use. 34.3% reported their facility had changed PPE use guidelines and 34.3% reported isolation guidelines had been changed midway through the H1N1 response.
	Van Dijk et al. (2015)[53]: 71.8% believed information provision was well-timed, 65.1% believed information provision was complete, 59.9% believed infection prevention policy changes were well-communicated, 59.2% believed information was explicitly formulated, and 68.5% felt they were clear on when to take protection measures.
	Kang et al. (2018)[54]: Participants described confusion from non-standardized and constantly evolving protocols, making it difficult to develop appropriate PPE protocols and training.
	Wong et al. (2012)[55]: Participants reported difficulties with guidelines as they changed so often.
	Lam & Hung (2013)[56]: Half the participants were satisfied that departmental communication about guidelines was effective, but others had problems with excessive guidelines and rapidly-changing regulations.
	Kang et al. (2018)[58]: Participants cited frequently changing guidelines as a stressor.
	Tolomiczenko et al. (2005)[61]: Emergency room personnel and intensive care unit personnel, more than other groups, reported poor communication about SARS and false ideas about safety precautions, adding that they were not kept up-to-date about what to wear and that personnel at the hospital entrance had no idea what the rules of the day were regarding what should be worn and when.

	Hsu et al. (2006)[62]: 37% participants reported poor communication between agencies and 30% reported a lack of standard operating procedures.
	Nhan et al. (2012)[63]: Participants reported that communication of guidelines was a barrier to compliance: guidelines were inconsistent between different pandemic management levels and advisory committees, often lacked clarity, were from an overwhelming number of different sources, frequently changed, and were slow to be communicated. Guidelines were also perceived as too rigid to accommodate particular regional or local situations.
	Rambaldini et al. (2005)[64]: Participants reported variability in the information made available at different hospitals, the perceived inconsistency of interhospital and interdepartmental interpretation of public health directives, and the lack of communication between institutions. Some reported frustration at receiving information filtered down from other sources, rather than first-hand. Residents at other hospitals, however, reported receiving regular updates from program directors, chief medical residents and infection control specialists in a variety of forms including websites, email and direct communication via personal conversations or group meetings. The most important information was deemed to be changes to infection control protocols, the status of SARS at their hospital and within the community, and the clinical condition of affected health care colleagues.
	Robertson et al. (2004)[65]: Participants reported a lack of clear guidelines about how to minimise infection at home and in quarantine and uncertainty about effective risk control, leading them to fear contaminating their family members. Unclear guidelines meant that staff developed their own thoughts about which behaviours were necessary and which were not.
Distress and risk perception	

Distress	Chia et al. (2005)[43]: Staff with IES scores of 20 or greater were more likely to wear more effective respiratory protection than those with scores of 19 or less.
	Wong et al. (2004)[45]: Staff who were highly anxious/frightened of dealing with SARS were significantly more likely to insist on patients wearing masks (68.1% v 48.5%, p<0.05), to ensure their staff wore gloves at work (32.4% v 17.9%, p<0.05), and to wash their hands before entering their home (79.4% v 61.2%, p<0.05).
	DiGiovanni et al. (2004)[47]: 5% reported such high stress they were tempted to break quarantine.
Risk perception	Kim & Choi (2016)[20]: Risk perception was significantly positively correlated with preventive behaviours (p<0.01).
	Taghrir et al. (2020)[21]: There was a significant negative correlation between preventive behaviours and fear (fear of becoming infected and thinking they are more likely to become infected than others) – as preventive behaviours increased, risk perception declined.
	Jeong et al. (2011)[22]: Perceived susceptibility to the pandemic significantly predicted preventive behaviours.
	Chor et al. (2012)[23]: In Hong Kong, compliance with infection control practices (wearing gloves, changing gloves and washing hands before each patient contact) was associated with the perceived seriousness of the likely impact of pandemic H1N1 on their life; however, compliance with these practices did not correlate with the perceived disease severity among staff in Singapore or the UK.
	Parker & Goldman (2006)[34]: Considering SARS to be a high, rather than low, public health threat was significantly associated with increased compliance with hand-washing, wearing a mask at all times in the emergency department, and wearing gloves when examining patients (p<0.05).
	Moore et al. (2005)[39]: Some participants reported that nobody ‘cut corners’ as fear of infection was very real.

	DiGiovanni et al. (2004)[47]: Staff who directly cared for SARS patients feared becoming infected and passing along infection to their families, so they not only complied with quarantine but also restricted social contact more strictly and for longer than required, with many sending their families away or living alone in their basements; 94% reported the most important reason for complying was to reduce the risk of transmission to others.
	Rebmann & Wagner (2009)[51]: Participants believed that healthcare workers' perceptions of H1N1 severity led to poor compliance with PPE; for example, one participant reported that staff chose not to wear PPE with the idea that they wanted to get mild flu so they wouldn't get a more virulent form of the disease in the autumn.
	Goulia et al. (2010)[66]: Degree of worry about H1N1 was significantly associated with restricting social contacts outside of work because the work environment was considered dangerous ($p<0.0005$).
Attitudes and behaviours of others	
Non-compliance of others	Hu et al. (2012)[27]: 21% reported their colleagues often forgot to use PPE during patient care.
	Chau et al. (2008)[30]: A common barrier to compliance identified by participants was poor preventive practices by colleagues, patients and visitors.
	Moore et al. (2005)[39]: Basic infection control policies and procedures were frequently perceived as not being enforced; participants identified various deficiencies including tracking who received training, lack of consistent policies for quarantining people, reuse of masks, and lack of consistency in deciding which patients require negative pressure rooms.
	Hsu et al. (2011)[42]: 56.1% of participants believed that senior clinicians leading by example was the most important strategy for improving hand hygiene compliance, and 10.1% believed that peer example (i.e. senior staff not practicing

	hand hygiene) was a reason for poor compliance. 2.4% of participants believed support and emphasis of guidelines by top administrative staff was the most important strategy for improving hand hygiene compliance.
	Yassi et al. (2005)[46]: The peer environment, especially the compliance of other occupational groups (including physicians), and the feedback from peers were identified as factors that could exert positive or negative influence on individual worker actions.
	DiGiovanni et al. (2004)[47]: As hospitals began to experience critical staff shortages, many modified the recommended quarantine guidelines to allow their staff to return.
	Locatelli et al. (2012)[50]: Participants reported resistance from administration staff about putting up signs with precautions, because they thought it might cause panic.
Attitudes of family members	Moore et al. (2005)[39]: Participants reported that a facilitator of compliance was the attitude of family members, who wanted them to comply with recommended measures as they were scared of getting sick and were often angry or upset that their healthcare worker family member was continuing to work.
	Yassi et al. (2005)[46]: Attitudes of family members, in particular the fear that family members expressed toward contracting SARS, influenced workers' behaviour.
Other potential predictors of compliance	
Complacency	Tan et al. (2006)[40]: Participants reported not wearing gowns due to complacency.
Commitment	Alsahafi & Cheng (2016)[28]: 96.2% of nurses, 93.6% of physicians and 98.4% of other healthcare staff believed that healthcare workers' lack of commitment to policies and procedures reduced compliance.

	Moore et al. (2005)[39]: Participants reported a lack of commitment to occupational health and safety by workers and management.
Previous experience with pandemic	Moore et al. (2005)[39]: Some participants reported that past exposures to disease led to decreased compliance when experience demonstrated that protective behaviours are not needed 100% of the time.
Passing of time	Rebmann & Wagner (2009)[51]: Participants reported that compliance lowered with time, as it was hard to keep vigilant in the middle of outbreak.
Forgetting	Hu et al. (2012)[27]: Approximately 21% reported they forgot to change PPE between patients. Forgetting to use or change PPE was significantly associated with poor compliance ($p<0.001$).
Death of a doctor	Tan et al. (2006)[40]: Compliance improved after the death of a doctor friend.
Effort-reward imbalance	Pratt et al. (2009)[18]: Effort-reward imbalance ratio was significantly predictive of decreased compliance ($p<0.05$).
Consulting with experts	Manabe et al. (2012)[15]: Consulting with experts with experience of caring for H5N1 patients did not significantly predict compliance with recommended behaviours ($p=0.776$).
Lack of staff	Moore et al. (2005)[39]: Participants felt there were not enough infection control practitioners, or they were not visible enough.
	Corley et al. (2010)[41]: Participants reported that not having enough staff (or enough experienced staff working at once) was a barrier to compliance.
PPE fit-testing procedures	Moore et al. (2005)[39]: Participants reported the fit-testing process for PPE was not standardised and some felt it was unnecessary.
Professionalism	Yassi et al. (2005)[46]: Professionalism was identified as having an important influence on adherence to procedures.

Use of public transport	Evirgen et al. (2014)[19]: Behaviour was not significantly associated with using public transportation to get to work.
Loss of income	DiGiovanni et al. (2004)[47]: Fear of loss of income was an obstacle to complying with quarantine, particularly among those unconvinced quarantine was necessary – 60% of doctors, 76% of nurses and 70% of other healthcare workers said they would need fairly detailed information about when, how, and how much compensation they would receive as encouragement to comply with quarantine.
Overcrowding	Alsahafi & Cheng (2016)[28]: Overcrowding in the emergency department was cited as a reason for poor compliance (97.2% of nurses, 96.6% of physicians, 93.2% of other healthcare staff)

Appendix I. Search strategy

Search 1: Compliance-related terms
compliance OR comply OR adhere* OR attitude* OR behaviour* OR behavior*

Search 2: Infection control-related terms
hygien* OR handwashing OR hand-washing OR infection control OR mask* OR PPE OR protective equipment OR social distanc*

Search 3: Healthcare worker-related terms
healthcare worker* OR healthcare staff OR healthcare personnel OR "medical personnel" OR "hospital personnel" OR frontline OR medical staff OR medical worker* OR doctor* OR dentist* OR midwife* OR midwives OR surgeon* OR nurse* OR gp* OR general practitioner* OR physician* OR health care worker* OR health care staff OR hospital employee* OR medical employee* OR healthcare employee* OR health care employee* OR hospital worker* OR hospital staff

Search 4: Emerging infectious disease-related terms
SARS OR severe acute respiratory syndrome OR MERS OR middle east respiratory syndrome OR H1N1 OR swine flu OR H5N1 OR avian influenza OR bird flu OR pandemic* OR coronavirus OR COVID-19 OR n-COV OR SARS-COV-2

Searches 1, 2, 3 and 4 were combined with AND.

Brooks SK, *et al. BMJ Open* 2022; 11:e049857. doi: 10.1136/bmjopen-2021-049857

Appendix II. Flow diagram of screening process

PRISMA 2009 Flow Diagram

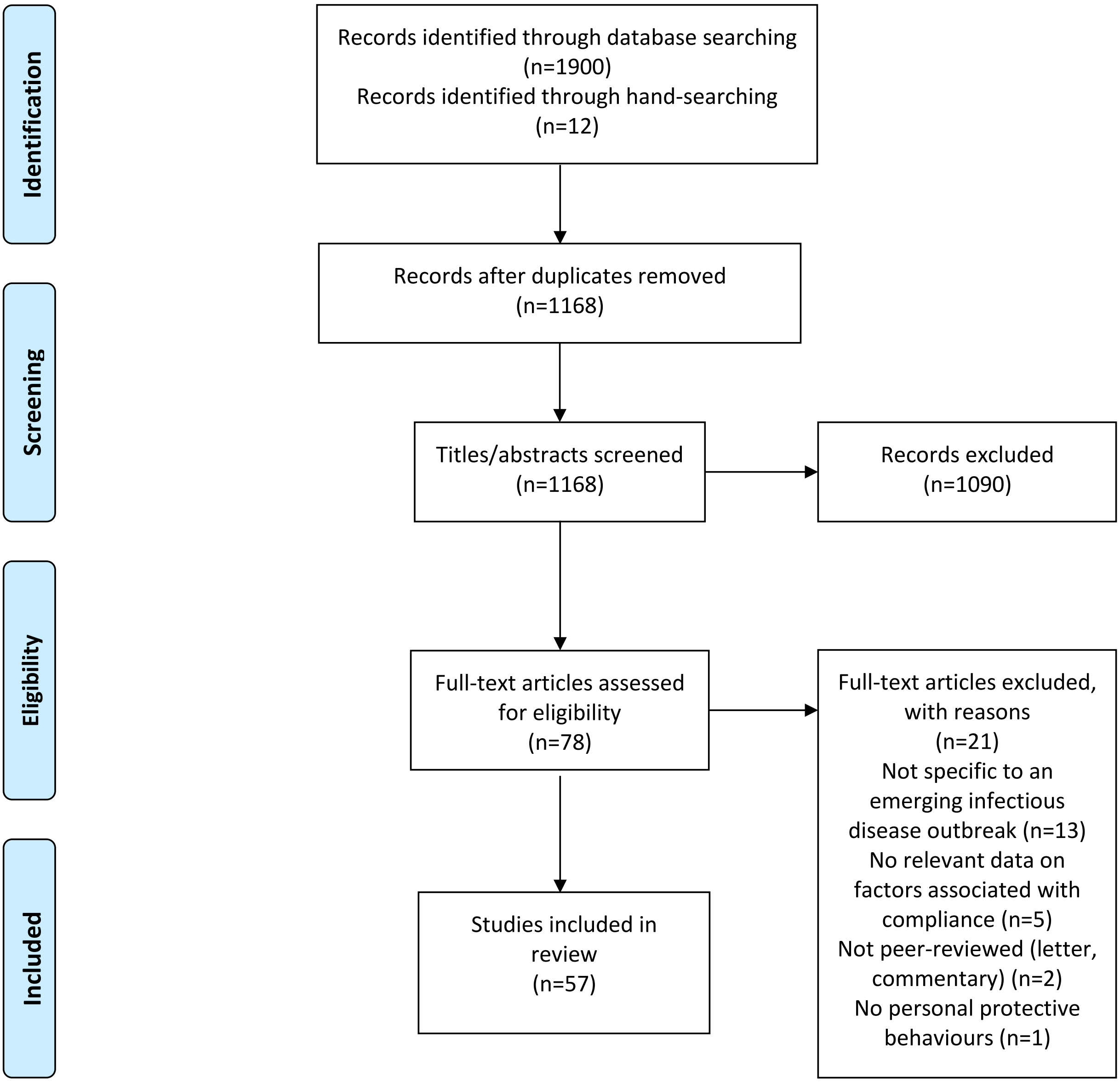

Supplement: Supplementary data [file bmjopen-2021-049857supp001.pdf]
