# Supplementary material for: Factors affecting healthcare workers’ compliance with social and behavioural infection control measures during emerging infectious disease outbreaks: rapid evidence review
Source: BMJ Open. 2021 Aug 14;11(8):e049857. doi: 10.1136/bmjopen-2021-049857 (PMC8370838; doi:10.1136/bmjopen-2021-049857)
Supplement: Author's manuscript [file bmjopen-2021-049857.draft_revisions.pdf]

BMJ Open

BMJ Open is committed to open peer review. As part of this commitment we make the peer review history of every article we publish publicly available.

When an article is published we post the peer reviewers' comments and the authors' responses online. We also post the versions of the paper that were used during peer review. These are the versions that the peer review comments apply to.

The versions of the paper that follow are the versions that were submitted during the peer review process. They are not the versions of record or the final published versions. They should not be cited or distributed as the published version of this manuscript.

BMJ Open is an open access journal and the full, final, typeset and author-corrected version of record of the manuscript is available on our site with no access controls, subscription charges or pay-per-view fees (http://bmjopen.bmj.com).

If you have any questions on BMJ Open's open peer review process please email info.bmjopen@bmj.com

BMJ Open

Factors affecting healthcare workers' compliance with social and behavioural infection control measures during emerging infectious disease outbreaks: Rapid evidence review.

Journal:	*BMJ Open*
Manuscript ID	bmjopen-2021-049857
Article Type:	Original research
Date Submitted by the Author:	08-Feb-2021

Complete List of Authors:	BROOKS, SAMANTHA; King's College London, Psychological Medicine<br>Greenberg , N; King's College London, Academic Department of Military Mental Health<br>Wessely, Simon; King's College London, KCMHR<br>Rubin, GJ; King's College London, Psychological Medicine

Keywords:	COVID-19, Public health < INFECTIOUS DISEASES, Infection control < INFECTIOUS DISEASES

SCHOLARONE™
Manuscripts

I, the Submitting Author has the right to grant and does grant on behalf of all authors of the Work (as defined in the below author licence), an exclusive licence and/or a non-exclusive licence for contributions from authors who are: i) UK Crown employees; ii) where BMJ has agreed a CC-BY licence shall apply, and/or iii) in accordance with the terms applicable for US Federal Government officers or employees acting as part of their official duties; on a worldwide, perpetual, irrevocable, royalty-free basis to BMJ Publishing Group Ltd ("BMJ") its licensees and where the relevant Journal is co-owned by BMJ to the co-owners of the Journal, to publish the Work in this journal and any other BMJ products and to exploit all rights, as set out in our licence.

The Submitting Author accepts and understands that any supply made under these terms is made by BMJ to the Submitting Author unless you are acting as an employee on behalf of your employer or a postgraduate student of an affiliated institution which is paying any applicable article publishing charge ("APC") for Open Access articles. Where the Submitting Author wishes to make the Work available on an Open Access basis (and intends to pay the relevant APC), the terms of reuse of such Open Access shall be governed by a Creative Commons licence – details of these licences and which Creative Commons licence will apply to this Work are set out in our licence referred to above.

Other than as permitted in any relevant BMJ Author's Self Archiving Policies, I confirm this Work has not been accepted for publication elsewhere, is not being considered for publication elsewhere and does not duplicate material already published. I confirm all authors consent to publication of this Work and authorise the granting of this licence.

For peer review only

Factors affecting healthcare workers' compliance with social and behavioural infection control measures during emerging infectious disease outbreaks: Rapid evidence review.

Dr Samantha K. Brooks PhD[1], Professor Neil Greenberg FRCPsych[2], Professor Sir Simon Wessely FMedSci[3], Dr G. James Rubin PhD[4]

[1]King's College London, Department of Psychological Medicine, Cutcombe Road, London SE5 9RJ, United Kingdom

[2]King's College London, Department of Psychological Medicine, Cutcombe Road, London SE5 9RJ, United Kingdom

[3]King's College London, Department of Psychological Medicine, Cutcombe Road, London SE5 9RJ, United Kingdom

[4]King's College London, Department of Psychological Medicine, Cutcombe Road, London SE5 9RJ, United Kingdom

Corresponding author: Dr S.K. Brooks, King's College London, Department of Psychological Medicine, Cutcombe Road, London SE5 9RJ, United Kingdom. Email: samantha.k.brooks@kcl.ac.uk Telephone: 020 7848 0432

Keywords Compliance, COVID-19, healthcare workers, infection control, public health

Word count 4,281

For peer review only

Abstract

Objective The 2019-2020 outbreak of novel coronavirus has raised concerns about nosocomial transmission. This review's aim was to explore the existing literature on emerging infectious disease outbreaks to identify factors associated with compliance with infection control measures among healthcare staff.

Methods A rapid evidence review for primary studies relevant to healthcare workers' compliance with infection control measures.

Results Fifty-six papers were reviewed. Staff working in emergency or intensive care settings or with contact with confirmed cases appeared more likely to comply with recommendations. There was some evidence anxiety and concern about the risk of infection were more associated with compliance, and that monitoring from superiors could improve compliance. Observed non-compliance of colleagues could hinder compliance. Staff identified many barriers to compliance related to personal protective equipment, including availability; perceived difficulty and effectiveness; inconvenience; discomfort; and a negative impact on patient care. There were many issues regarding the communication and ease of understanding of infection control guidance.

Conclusion We recommend provision of training and education tailored for different occupational roles within the healthcare setting; managerial staff 'leading by example'; ensuring adequate resources for infection control; and timely provision of practical evidence-based infection control guidelines.

For peer review only - http://bmjopen.bmj.com/site/about/guidelines.xhtml

Strengths and limitations of this study

- This review synthesises existing literature on healthcare workers' compliance with infection control measures during infectious disease outbreaks, providing an overview of what has already been established and identifying gaps in the literature.
- The search strategy was broad, and reference lists of included papers were hand-searched for any additional relevant papers which may have been missed.
- The search was limited to English-language papers due to the rapid nature of the review.
- Included papers did not undergo formal quality appraisal, again due to the rapid nature of the review.

For peer review only

Introduction

Recent years have seen frequent outbreaks of emerging infectious diseases.[1] Examples include severe acute respiratory syndrome (SARS) in 2003, avian influenza (H5N1) in 2004, swine flu (H1N1) in 2009, Middle East respiratory syndrome (MERS) in 2015 and most recently coronavirus SARS-CoV-2 originating in 2019. Previous outbreaks saw high levels of nosocomial transmission (hospital-acquired infections).[2]

A major cause of nosocomial transmission is poor compliance with personal protective behaviours among healthcare staff.[3 4] Early reports from the COVID-19 frontline have noted poor compliance of healthcare workers with recommended behaviours: in one hospital in China, many had their masks hung from one ear or pulled downwards, while more than half had inadequate hand hygiene.[5]

Compliance with infection control behaviours can be difficult. Previous literature has reported on difficulties in the general population with adhering to protective behaviours such as facemask-wearing,[6] using hand sanitising gel[7] and quarantine[8] during infectious disease outbreaks. The main facilitators of compliance in the general population appear to be perceived susceptibility, perceived severity of being afflicted and perceived benefits of

For peer review only - http://bmjopen.bmj.com/site/about/guidelines.xhtml

compliance as well as accurate knowledge about the disease and the recommended behaviours, while major barriers include discomfort, embarrassment and practical issues.[6 8] A review[9] of healthcare workers' perceptions of barriers and facilitators to compliance with guidelines during respiratory outbreaks suggested that protective practices are influenced by understanding of guidelines, support received from managers, communication about guidelines, sufficient resources, perceived value of following guidance, comfort of personal protective equipment (PPE), perceived impact of PPE on patients, and workplace culture. However, this review focused only on qualitative literature, the majority of which related to tuberculosis.

For peer review only

We systematically reviewed existing literature on compliance with social and behavioural protective behaviours among staff involved in healthcare, specifically during outbreaks of emerging infectious diseases and encompassing quantitative and qualitative research.

Methods

This rapid evidence review was carried out according to World Health Organization guidelines:[10] the basic principles of a systematic literature review were followed, with certain aspects simplified in order to produce evidence rapidly at a time when urgent evidence synthesis is required. Searching of grey literature and quality appraisal of included studies were not carried out.

Search strategy

The search strategy consisted of four search strings (adherence terms; protective behaviour terms; emerging infectious disease terms; and healthcare worker terms). The full search strategy can be seen in Appendix I. Five databases were searched from date of inception to May 4th, 2020: MEDLINE®, PsycINFO®, Embase, Global Health, and Web of Science.

Selection criteria

To be included, studies had to: i) contain primary data; ii) be published in peer-reviewed journals in English; iii) include participants who worked in healthcare; iv) include data on factors predicting adherence to social or behavioural infection control practices during emerging infectious disease pandemics. For the latter criterion, quantitative data needed to report statistics on factors associated with compliance, while the qualitative component of the review considered studies reporting on participants' beliefs about facilitators and barriers to compliance as well as any reported difficulties in complying with protective behaviours.

Screening

One author (SKB) ran the searches on all databases on May 4th, 2020. Resulting citations were downloaded to EndNote© version X9 (Thomson Reuters, New York, USA). The same author evaluated titles for relevance, then used the inclusion criteria to screen abstracts and then full texts of remaining citations and exclude any which were irrelevant. Reference lists of all remaining papers were hand-searched for additional relevant studies.

Data extraction and synthesis

SKB extracted the following data from papers: authors, publication year, country of study, design, participants (including *n,* demographic information and profession), disease outbreak, protective behaviours measured, measures used, and key results. We used thematic analysis[11] to synthesise the data and group results into themes.

For peer review only - http://bmjopen.bmj.com/site/about/guidelines.xhtml

Patient and public involvement

No patients were involved in this review study.

Results

The initial search strategy yielded 1900 papers, of which 744 duplicates were removed and 1090 were excluded based on title or abstract. An additional twelve papers were found via hand-searching the reference lists of included papers. After full-text screening, 56 papers remained for inclusion. A PRISMA flow diagram of the process can be seen in Appendix II.

Countries represented in the literature included Canada (n=13), Saudi Arabia (n=7), Singapore (n=7), China (n=5), South Korea (n=5), USA (n=4), Netherlands (n=2), Australia (n=1), Greece (n=1), India (n=1), Iran (n=1), Taiwan (n=1), Turkey (n=1), UK (n=1) and Vietnam (n=1). A further five papers included participants from multiple countries. Papers discussed H1N1 (n=21), SARS (n=20), MERS (n=11), avian influenza (n=2) and COVID-19 (n=2). The participants represented in the literature were from a wide range of roles and departments in the healthcare profession, and a wide range of protective behaviours were considered. A full overview of study characteristics is presented in Supplementary Table I.

Eight main themes were identified. These were socio-demographics and personal characteristics; occupational role; training and knowledge; work-related factors; personal protective behaviour-related factors; guidance; distress and risk perception; and attitudes and behaviours of others. A number of other potential predictors of compliance were considered but only appeared in one paper each; these are presented in Supplementary Tables I and III but not covered in the text. Supplementary Table II provides a summary of themes and sub-themes and identifies for each theme which papers showed a significant association with protective behaviours, which papers found no significant association, and which papers supplemented these findings by reporting on the theme but without statistical analysis (e.g. qualitative papers and papers with descriptive statistics only). Supplementary Table III summarises the evidence extracted from the literature for each theme.

Socio-demographics and personal characteristics

For peer review only - http://bmjopen.bmj.com/site/about/guidelines.xhtml

Overall, there appeared to be no significant association between age[12-18] or gender[12-14 17 19-21] and protective behaviour. Only one study[20] found that older age was significantly associated with protective behaviours, while two found that female staff were significantly more likely to comply with protective behaviours.[16 22]

One study examined nationality as a predictor[12] and found that Saudi staff were significantly more likely to comply with protective behaviours than non-Saudi staff working in the same city. Mixed findings were reported in the studies comparing behaviours across countries. Staff in Hong Kong and Singapore were more likely to comply than UK staff[23] whereas Koh et al.[24] and Wong et al.[25] found that staff in Singapore vs Indonesia or Hong Kong vs Canada respectively were more likely to comply with some recommendations but not others (see Supplementary Table III for details). A worldwide study[26] found no significant differences between countries in terms of taking protective measures.

There was no evidence of association between compliance and religion[20] or marital status.[18 19] One paper found that staff in 'high or middle' socioeconomic status were more likely to comply than those of lower socioeconomic status.[22] Four studies found that level of education was not associated with compliance[13 18 20 21] while one found a significant association between compliance and qualification[12] (see Table III for details). One study found that H1N1 influenza vaccination was significantly associated with high compliance.[27] Having a chronic illness, being pregnant, or having a pregnant spouse, elderly person or school-aged child at home were not associated with compliance, but staff with babies at home were more likely to comply with protective behaviours.[19]

Occupational role

Many studies which examined role as a predictor of compliance found a significant relationship;[12 19 23 28-36] however, due to the variety of different roles compared across studies, it was not possible to identify an exact pattern (see Supplementary Table III for details). Five studies found no significant association between role and compliance.[13 16 17 37 38]

Length of time in role was not significantly associated with compliance in five studies[12 16-18 30] while two studies suggested that longer experience of working in healthcare was associated with greater compliance[31 33] and one suggested that less than ten years' experience was associated with significantly higher compliance than more experience.[36]

For peer review only - http://bmjopen.bmj.com/site/about/guidelines.xhtml

Training and knowledge

There was mixed evidence on the effectiveness of outbreak-specific training and education: Taghrir et al.[21] found no significant association between protective behaviours and having received education; Nour et al.[17] found a non-significant increase in protective practices post-training; and Shigayeva et al.[35] found that recent infection control training was a significant predictor of compliance with recommended behaviours. Jeong et al.[22] found that staff who sought information about the outbreak and infection control were more likely to comply with recommended behaviours. Qualitative evidence suggested that staff felt their prior training and education were not useful in dealing with the rapidly-changing nature of emerging infectious disease outbreaks.[39 40] Participants themselves believed that inadequate training was a barrier to compliance[28] and that infection control training with annual refresher courses would benefit them.[41]

Sources of knowledge about the outbreak and protective behaviours were not associated with protective behaviours in two studies[20 21] while knowledge from textbooks and attending Continuing Medical Education activities were significantly associated with higher levels of protective practice in one study.[12] Receiving outbreak-specific training was not significantly associated with compliance, but higher outbreak-related knowledge did result in significantly higher compliance.[20] Another study examining knowledge of the outbreak itself[28] found that the majority of participants believed lack of knowledge about mode of transmission contributed to poor compliance. Knowledge of current recommendations was associated with compliance in three studies;[4 16 27] associated with compliance in one hospital but not three other hospitals in another study;[14] and was not associated with compliance in one further study.[12] Hsu et al.[42] found that a minority of participants believed that lack of education explained lack of compliance.

Work-related factors

Compliance appeared to be higher in higher acuity settings such as emergency, intensive care or inpatient departments.[14 35 38 43 44] Wong et al.[45] found that staff in high-infection districts were more likely to wear gowns, wash hands and use disinfectants but less likely to comply with quarantine measures. Two studies found no significant association between setting and compliance.[19 21]

For peer review only - http://bmjopen.bmj.com/site/about/guidelines.xhtml

Having contact with confirmed cases was associated with higher compliance in three studies[14 35 43] whereas one study[19] found no association. A further study[18] found higher compliance in staff who worked directly with confirmed cases. Wong et al.[45] found that SARS-exposed staff were more likely than non-SARS-exposed staff to comply with mask guidance, but less likely to quarantine themselves.

There was some evidence that high workload may be a barrier to compliance with recommended personal protective behaviours,[23 30 35 36 39 41 46] although one study also suggested that higher workload (in terms of working overtime) was associated with increased compliance in terms of giving patients appropriate infection control advice.[36]

Two studies suggested that monitoring of compliance by superiors or public health authorities encouraged compliance,[27 47] while a small minority of participants in Hsu et al.'s[42] study reported that better policing by infection control staff was the most important strategy for improving compliance.

Two studies suggested compliance was associated with characteristics of the patient encounter. Many of De Perio et al.'s[14] participants reported not using recommended PPE if they did not know the patient had H1N1 or an influenza-like illness, if they did not think it was needed for the particular activity they were doing, if they only entered the patient's room for a brief time, if they did not touch the patient or if they did not come within six feet of the patient. Meanwhile, Shigayeva et al.'s[35] participants were less likely to comply with recommended behaviours when providing care for patients with more severe illness (which the authors suggest may be due to the time required to don barrier equipment leading staff to put patient safety above self-protection) and if they were only observing procedures, rather than performing or assisting with them.

Personal protective behaviour-related factors

Many studies, although mostly without statistical analysis, reported issues in performing personal protective behaviours, most notably due to lack of availability of appropriate PPE, perceived difficulty of protective behaviours, logistic issues, perceived effectiveness, perceived importance, convenience, comfort, and the impact on patient care.

For peer review only - http://bmjopen.bmj.com/site/about/guidelines.xhtml

Participants in several studies raised concerns about lack of PPE,[14 39-42 48-53] mostly due to insufficient resources. Shortage of PPE created difficulties such as having to wear the wrong size PPE.[40 41 51] One study showed that availability of PPE was significantly associated with higher compliance[27] while another found that staff were significantly more compliant with PPE use when eyewear and gloves were readily available at the point of care, but availability did not increase compliance of N95 respirators, surgical masks or gowns.[33] Logistic issues were also reported, such as lack of space in the hospital, making it difficult to use PPE appropriately.[51 54 55]

There may also be issues regarding the perceived difficulty of protective behaviours. Kang et al.'s[54] participants reported difficulties with the complexity of using several PPE items together, and only 35% of Van Dijk et al.'s[53] participants thought the recommended measures were feasible.

Perceived effectiveness of PPE significantly predicted higher compliance in three studies.[27 33 34] In one qualitative study, staff expressed doubts about the quality and effectiveness of PPE.[54] Beliefs in the effectiveness of infection control procedures, as modified by previous experiences, were identified by participants as having a positive impact on compliance.[46]

Hsu et al.[42] reported that failure to recognise the importance of hand hygiene prevented compliance, while Vinck et al.[36] found that the main reason for not complying with the recommendation to consult with a specialist unit for centralised assessment of symptomatic patients was associated with finding it 'unnecessary'.

Participants in many qualitative studies reported not using PPE due to perceived inconvenience and its effect on their ability to do their job[14 27 39-42 54 56 57] – in particular, it appeared that participants found it too time-consuming to change in and out of protective clothing as it slowed them down substantially.

Many of Khalid et al.'s[49] participants reported that having to use PPE was 'stressful'. Many qualitative studies found that discomfort of PPE was reported to be a barrier to compliance.[39-41 46 52 56-58] In several studies, participants reported physical harm due to PPE, including dehydration and skin peeling,[41] difficulty breathing,[40 52 58 59] sweating and dizziness,[58] headaches[40 52 60] and skin rashes.[40 52] Comfort of PPE was examined as a potential predictor

For peer review only - http://bmjopen.bmj.com/site/about/guidelines.xhtml

of compliance in one quantitative study[33] which found that staff who reported always or often feeling comfortable wearing protective eyewear and N95 respirators were significantly more likely to wear them; however, no significant association was found in compliance between staff who reported always or often feeling short of breath, claustrophobic or dizzy when wearing protective eyewear or N95 respirators and those who rarely experienced these symptoms.

Qualitative data also revealed that many healthcare professionals believe that PPE use has an impact on patient care, making it difficult to communicate with patients due to muffled speech,[40 41 52 56 57 59] being unable to establish non-verbal cues with patients[60] and making them less 'visible' to their patients.[61] Participants in Rowlands' study[52] also reported concern that masks could be frightening for psychiatric patients, and Tan et al.'s[40] participants reported that PPE created anxiety in some patients who assumed they were wearing it as they had been exposed to the virus. One quantitative study found that the perception that PPE use would interfere with patient care was significantly associated poor compliance.[27]

Guidance

Participants in many studies reported a lack of guidance on the protocols for caring for infected patients and protecting themselves.[28 30 39 40 41 46 50 51 53-56 58 61-65]

In particular, guidelines were often found to be too long, providing staff with 'information overload'.[30 50 63] The recommended protocols were also reported to change too frequently for staff to keep up[39 41 54-56 58 61 63 64] and staff received conflicting messages from different sources.[39 41 50 51 62-64] Other issues included inconsistent advice about what to do when patients are deemed non-infectious,[41] difficulty working out what should be prioritised[50] and external guidance with little relevance to specific locations.[39] The manner of communication of guidance could also be an issue, with Moore et al.'s[39] participants reporting that new guidelines were sent to them by email, which they did not check before work; Rambaldini et al.'s[64] participants reporting frustration that information was filtered down from other sources rather than given to them first-hand; and Nhan et al.'s[63] participants suggesting that communication about changes to protocols was too slow.

Distress and risk perception

For peer review only - http://bmjopen.bmj.com/site/about/guidelines.xhtml

High levels of distress were associated with higher compliance in two studies: Chia et al.[43] found that higher IES scores were associated with higher use of respiratory protection while Wong et al.[45] found that staff who were highly anxious were more likely to comply with recommended protective behaviours. However, when the protective measure involved quarantine, some staff felt such high stress that they were tempted to break guidelines[47] (it is unclear from the paper what percentage of these actually did break the guidelines, if any).

Risk perception appeared to be associated with compliance. Four studies found that compliance was significantly more likely in staff who perceived the disease to be a serious risk[20 22 34 66] and another found that perceived seriousness of the outbreak was significantly associated with compliance in Hong Kong but not in Singapore or the UK.[23] Three qualitative papers also suggested that participants themselves believed staff complied with recommended behaviours when they perceived the risk to be severe.[39 47 51] However, one study[21] showed a significant negative correlation between protective behaviours and fear of infection.

Attitudes and behaviours of others

Qualitative data revealed that many participants had observed non-compliance in colleagues or managers[27 39] which could lead to non-compliance in the participants themselves.[30,42] DiGiovanni et al.'s[47] participants suggested that managers had modified the recommended quarantine guidelines to allow their staff to return in response to critical staff shortages. Yassi et al.'s[46] participants believed that compliance of other occupational groups within the healthcare setting had an impact on compliance, while more than half of Hsu et al.'s[42] participants believed that senior staff 'leading by example' and complying with recommended behaviours was the single most important strategy in improving staff compliance.

Attitudes of family members were also deemed important in two qualitative studies[39 46] with healthcare staff reporting they were encouraged to comply by anxious family members who were afraid of getting infected.

Discussion

Overall, the studies reviewed provided mixed and sometimes contradictory results. Nonetheless, some risk factors which stood out as being the most promising either for

For peer review only - http://bmjopen.bmj.com/site/about/guidelines.xhtml

identifying specific groups at-risk for poor compliance or for targeting in interventions. These included: working outside of emergency or intensive care settings; not working with confirmed infection cases; lack of concern about risk of infection; lack of monitoring by superiors; observed non-compliance of colleagues; lack of PPE; perceived difficulty using PPE; perceived lack of effectiveness or lack of importance of PPE; perceived inconvenience and discomfort of PPE; perceived negative impact of PPE on patient care; lack of infection control guidance; and inconsistent or unclear guidance. Organisations faced with nosocomial transmission would be wise, as part of their mitigation efforts, to look to these areas in an effort to help staff adhere to protective behaviours.

There was little evidence for an association between compliance with protective behaviours and socio-demographic or personal characteristics. There was some evidence that compliance levels differ across countries: it is not clear whether this is due to between-country differences in the communication of guidance, different risk perceptions due to different countries' media coverage of the outbreak, different levels of training received, or cultural differences in the participants themselves.

This review suggests that compliance may differ between different roles and different settings. Targeted interventions for specific occupational groups with different levels of patient contact, hierarchies and cultures, may be helpful. Whilst it was unclear from the varied data which occupational groups might need more attention, it seems that those not in emergency departments or intensive care units and not working directly with infected patients could benefit from additional focus.

We found little evidence that training and education significantly improved compliance. This initially seems surprising as previous trials of training courses to improve knowledge of infection control practices and encourage compliance with them have suggested positive results.[67-69] However, taken together with the evidence that compliance differs between occupational groups, it may be that staff in different roles require different levels and methods of training. We also found mixed evidence of the impact of knowledge about infection control on compliance with the recommended measures: this may be associated with what is actually covered in training. For example, as it appears that the perceived importance of PPE is related to compliance, it may be that training that covers the 'why' rather than the 'how' of PPE is particularly useful. However, training itself should not be

For peer review only - http://bmjopen.bmj.com/site/about/guidelines.xhtml

viewed as a panacea. Many of the factors identified by our review, and discussed below, are not amenable to better training.

A small amount of evidence suggested that observed non-compliance by others – including colleagues - could affect healthcare workers' own levels of compliance. This is likely to be particularly problematic where managers and supervisors fail to 'lead by example' and ensure that they comply with the recommended policies and procedures. 'Role modelling' by superiors could be useful, with supervisors setting the standards for infection control practices and reinforcing them.

The availability, ease of use, perceived effectiveness, convenience, comfort, and impact on patients of PPE may be other key factors which need to be addressed. This review suggests fairly negative views of PPE, with participants reporting lack of appropriate resources, the inconvenience of having to change in and out of PPE repeatedly, physical discomfort, and a negative impact on their ability to communicate with patients. It is useful to know about these concerns about PPE as this can be a target to change.

Reviewed studies suggest difficulties understanding and keeping up with rapidly changing recommendations. This may be unavoidable during an outbreak of an emerging infectious disease, as new evidence becomes available. Staff should therefore be prepared for the likelihood of changes, both to help maintain trust and to ensure that they monitor for updates.

There was some evidence that higher levels of anxiety and risk perception were associated with higher compliance. This may suggest that, whilst it is important not to create unhealthy anxiety, desensitisation to risk or genuine reductions in risk may lead to a reduction in PPE use. Indeed, it has been reported that staff in industrial settings experience complacency in terms of protective behaviours, as a result of habituation (repeated exposures which do not result in anything bad, lessening fearful responding) and low levels of positive reinforcement for safe behaviour.[70] Particular care may be needed to maintain PPE use as risk changes, or is perceived to change, over the course of an outbreak.

Limitations

We are aware that many papers (particularly on SARS and MERS) have been published in other (predominantly Asian) languages, but due to the rapid nature of this review we limited

For peer review only

For peer review only - http://bmjopen.bmj.com/site/about/guidelines.xhtml

inclusion to English-language papers only. Future reviews should consider translating and analysing the many relevant foreign-language papers. Also due to the rapid nature of the review, quality appraisal of individual papers was not carried out as this is not always deemed necessary when urgent evidence synthesis is required.[71]

Conclusion

Overall this review identified many potential facilitators and barriers to adhering with recommended personal protective behaviours. Although much of the evidence was inconsistent some recommendations can be made. Interventions that may be helpful for improving healthcare workers' compliance include role-specific or setting-specific training; emphasising the importance of protective behaviours and the risk of infection if behaviours are not performed; monitoring of staff behaviours by supervisors and positive reinforcement for correct behaviours; managerial staff leading by example; training focused on the importance and effectiveness of PPE; and better communication of guidelines. The results of this review also suggest that the following interventions would likely *not* be useful: training aimed at specific age or gender groups; training focused on increasing knowledge about the outbreak itself; or training on how to use PPE without also emphasising *why* it is necessary.

Contributorship statement

GJR conceptualised the review. SKB developed the search strategy with input from GJR, NG and SW. SKB reviewed titles, abstracts and full-text papers for eligibility. Any concerns or queries about inclusion were discussed with GJR, NG and SW. SKB extracted data and prepared the manuscript. GJR, NG and SW reviewed and edited the manuscript.

Competing interests

None declared.

Funding

The research was funded by the National Institute for Health Research Health Protection Research Unit (NIHR HPRU) in Emergency Preparedness and Response at King's College London in partnership with Public Health England (PHE), in collaboration with the University of East Anglia. The views expressed are those of the author(s) and not necessarily those of the NHS, the NIHR, the Department of Health and Social Care, or Public Health

For peer review only

England. The funder had no involvement in the study design, collection, analysis or interpretation of data, writing the report, or decision to submit for publication.
Award number: NIHR200890

Data sharing statement

No original data were generated for this study.

References

1. World Health Organization, Regional Office for South-East Asia. (2014). A brief guide to emerging infectious diseases and zoonoses. WHO Regional Office for South-East Asia. https://apps.who.int/iris/handle/10665/204722 [accessed 09 May 2020]

2. Feng D, de Vlas SJ, Fang L-Q, Han XN, Zhao W-J, Sheng S, et al. The SARS epidemic in mainland China: bringing together all epidemiological data. Trop Med Int Health. 2009;14(Suppl 1):4-13.

3. Gamage B, Moore D, Copes R, Yassi A, Bryce E, members of The BC Interdisciplinary Respiratory Protection Study Group Vancouver, British Columbia, Canada. Protecting health care workers from SARS and other respiratory pathogens: A review of the infection control literature. Am J Infect Control. 2005;33(2),114-21.

4. Yap J, Lee VJ, Yau TY, Ng TP, Tor PC. Knowledge, attitudes and practices towards pandemic influenza among cases, close contacts, and healthcare workers in tropical Singapore: a cross-sectional survey. BMC Public Health. 2010;10:442.

5. Xu C, Jin J, Song J, Yang Y, Yao M, Zhang Y, et al. Application of refined management in prevention and control of the coronavirus disease 2019 epidemic in non-isolated areas of a general hospital. Int J Nurs Sci. 2020;7(2):143-7.

6. Sim SW, Moey KSP, Tan NC. The use of facemasks to prevent respiratory infection: a literature review in the context of the Health Belief Model. Singapore Med J. 2014;55(3):160-7.

For peer review only

For peer review only - http://bmjopen.bmj.com/site/about/guidelines.xhtml

7. Rubin GJ, Potts HWW, Michie S. The impact of communications about swine flu (influenza A H1N1v) on public responses to the outbreak: results from 36 national telephone surveys in the UK. Health Technol Assess. 2010;14(34):183-266.

8. Webster RK, Brooks SK, Smith LE, Woodland L, Wessely S, Rubin GJ. How to improve adherence with quarantine: rapid review of the evidence. Public Health. 2020;182:163-9.

9. Houghton C, Meskell P, Delaney H, Smalle M, Glenton C, Booth A, et al. Barriers and facilitators to healthcare workers' adherence with infection prevention and control (IPC) guidelines for respiratory infectious diseases: a rapid qualitative evidence synthesis. Cochrane Database of Systematic Reviews. 2020;4.

10. World Health Organization. (2017). Rapid reviews to strengthen health policy and systems: a practical guide. https://www.who.int/alliance- hpsr/resources/publications/rapid-review-guide/en/ [accessed 04 May 2020]

11. Braun V, Clarke V. Using thematic analysis in psychology. Qual Res Psychol. 2006;3(2):77-101.

12. Al-Amri S, Bharti R, Alsaleem SA, Al-Musa HM, Chaudhary S, Al-Shaikh AA. Knowledge and practices of primary health care physicians regarding updated guidelines of MERS-CoV infection in Abha city. J Family Med Prim Care. 2019;8(2):455-61.

13. Datta SS, Kuppuraman D, Boratne AV, Abraham SB, Singh Z. Knowledge, attitude and practices regarding swine flu among para-medical workers in a tertiary care hospital in Pondicherry. J Commun Dis. 2011;43(1):1-9.

14. de Perio MA, Brueck SE, Mueller CA, Milne CK, Rubin MA, Gundlapalli AV, et al. Evaluation of 2009 pandemic influenza A (H1N1) exposures and illness among physicians in training. Am J Infect Control. 2012;40(7):617-21.

15. Manabe T, Pham Thi Phuong T, Kudo K, Vu Thi Tuong V, Takasaki J, Nguyen Dang T, et al. Impact of education and network for Avian Influenza H5N1 in humans: Knowledge, clinical practice, and motivation on medical providers in Vietnam. Plos One. 2012;7(1).

For peer review only

For peer review only - http://bmjopen.bmj.com/site/about/guidelines.xhtml

16. Nour MO, Babalghith AO, Natto HA, Al-Amin FO, Alawneh SM. Knowledge, attitude and practices of healthcare providers towards MERS-CoV infection at Makkah hospitals, KSA. Int Res J Med Med Sci. 2015;3(4): 103-12.

17. Nour MO, Babalghith AO, Natto HA, Alawneh SM, Elamin FO. Raising awareness of health care providers about MERS-CoV infection in public hospitals in Mecca, Saudi Arabia. East Mediterr Health J. 2017;23(8):534-42.

18. Pratt M, Kerr M, Wong C. The impact of ERI, burnout, and caring for SARS patients on hospital nurses' self-reported compliance with infection control. CJIC. 2009;24(3):167-74.

19. Evirgen O, Savas N, Koksaldi Motor V, Onlen Y, Yengil E. An evaluation of knowledge, attitudes, and behaviors of employees of a university hospital in an H1N1 influenza pandemic. J Infect Dev Ctries. 2014;8(5):561-9.

20. Kim JS, Choi JS. Middle East respiratory syndrome-related knowledge, preventive behaviours and risk perception among nursing students during outbreak. J Clin Nurs. 2016;25(17-18):2542-9.

21. Taghrir MH, Borazjani R, Shiraly R. COVID-19 and Iranian medical students; A survey on their related-knowledge, preventive behaviors and risk perception. Arch Iran Med. 2020;23(4):249-54.

22. Jeong HS, Lee DW, Youn CH, Lee MK, Lee SJ, Suh YS, et al. Perception and performance of preventive behaviors for the pandemic influenza in hospital employees and outpatients. Yonsei Med J. 2011;52(1):181-7.

23. Chor JSY, Pada SK, Stephenson I, Goggins WB, Tambyah PA, Medina M, et al. Differences in the compliance with hospital infection control practices during the 2009 influenza H1N1 pandemic in three countries. J Hosp Infect. 2012;81(2):98-103.

24. Koh GC-H, Abikusno N, Kwing CS, Yee WT, Kusumaratna R, Sundram M, et al. Avian influenza and South Jakarta primary healthcare workers: a controlled mixed-method study. Trop Med Int Health. 2009;14(7):817-29.

For peer review only

25. Wong SYS, Wong W, Jaakkimainen L, Bondy S, Tsang KK, Lee A. Primary care physicians in Hong Kong and Canada - how did their practices differ during the SARS epidemic? Fam Pract. 2005;22(4):361-6.

26. Kamate SK, Sharma S, Thakar S, Srivastava D, Sengupta K, Hadi AJ, et al. Assessing knowledge, attitudes and practices of dental practitioners regarding the covid-19 pandemic: A multinational study. Dent Med Probl. 2020;57(1):11-7.

27. Hu X, Zhang Z, Li N, Liu D, He W, Zhang W, et al. Self-reported use of Personal Protective Equipment among Chinese Critical Care Clinicians during 2009 H1N1 influenza pandemic. PLoS ONE. 2012;7(9).

28. Alsahafi AJ, Cheng AC. Knowledge, attitudes and behaviours of healthcare workers in the Kingdom of Saudi Arabia to MERS Coronavirus and other emerging infectious diseases. Int J Environ Res Public Health. 2016;13(12).

29. Alsubaie S, Hani Temsah M, Al-Eyadhy AA, Gossady I, Hasan GM, Al-Rabiaah A, et al. Middle East Respiratory Syndrome Coronavirus epidemic impact on healthcare workers' risk perceptions, work and personal lives. J Infect Dev Ctries. 2019;13(10):920-6.

30. Chau JP, Thompson DR, Twinn S, Lee DT, Lopez V, Ho LS. An evaluation of SARS and droplet infection control practices in acute and rehabilitation hospitals in Hong Kong. Hong Kong Med J. 2008;14 Suppl 4:44-7.

31. Koh D, Lim MK, Chia SE, Ko SM, Qian F, Ng V, et al. Risk perception and impact of severe acute respiratory syndrome (SARS) on work and personal lives of healthcare workers in Singapore: What can we learn? Med Care. 2005;43(7):676-82.

32. May L, Katz R, Johnston L, Sanza M, Petinaux B. Assessing physicians' in training attitudes and behaviors during the 2009 H1N1 influenza season: a cross-sectional survey of medical students and residents in an urban academic setting. Influenza Other Respir Viruses. 2010;4(5):267-75.

For peer review only

33. Mitchell R, Ogunremi T, Astrakianakis G, Bryce E, Gervais R, Gravel D, et al. Impact of the 2009 influenza A (H1N1) pandemic on Canadian health care workers: A survey on vaccination, illness, absenteeism, and personal protective equipment. Am J Infect Control. 2012;40(7):611-6.

34. Parker MJ, Goldman RD. Paediatric emergency department staff perceptions of infection control measures against severe acute respiratory syndrome. EMJ. 2006;23(5):349-53.

35. Shigayeva A, Green K, Raboud JM, Henry B, Simor AE, Vearncombe M, et al. Factors associated with critical-care healthcare workers' adherence to recommended barrier precautions during the Toronto severe acute respiratory syndrome outbreak. Infect Control Hosp Epidemiol. 2007;28(11):1275-83.

36. Vinck L, Isken L, Hooiveld M, Trompenaars M, Ijzermans J, Timen A. Impact of the 2009 influenza A(H1N1) pandemic on public health workers in the Netherlands. Euro Surveill. 2011;16(7).

37. Alshammari M, Reynolds KA, Verhougstraete M, O'Rourke MK. Comparison of perceived and observed hand hygiene compliance in healthcare workers in MERS-CoV endemic regions. Healthcare (Basel). 2018;6(4).

38. Jaeger JL, Patel M, Dharan N, Hancock K, Meites E, Mattson C, et al. Transmission of 2009 Pandemic Influenza A (H1N1) Virus among healthcare personnel-Southern California, 2009. Infect Control Hosp Epidemiol. 2011;32(12):1149-57.

39. Moore DM, Gilbert M, Saunders S, Bryce E, Yassi A. Occupational health and infection control practices related to severe acute respiratory syndrome: health care worker perceptions. AAOHN J. 2005;53(6):257-66.

40. Tan NC, Goh LG, Lee SS. Family physicians' experiences, behaviour, and use of personal protection equipment during the SARS outbreak in Singapore: Do they fit the Becker Health Belief Model? Asia Pac J Public Health. 2006;18(3):49-56.

For peer review only

For peer review only - http://bmjopen.bmj.com/site/about/guidelines.xhtml

41. Corley A, Hammond NE, Fraser JF. The experiences of health care workers employed in an Australian intensive care unit during the H1N1 influenza pandemic of 2009: A phenomenological study. Int J Nurs Stud. 2010;47(5):577-85.

42. Hsu LY, Jin J, Ang BS, Kurup A, Tambyah PA. Hand hygiene and infection control survey pre- and peri-H1N1-2009 pandemic: knowledge and perceptions of final year medical students in Singapore. Singapore Med J. 2011;52(7):486-90.

43. Chia SE, Koh D, Fones C, Qian F, Ng V, Tan BH, et al. Appropriate use of personal protective equipment among healthcare workers in public sector hospitals and primary healthcare polyclinics during the SARS outbreak in Singapore. Occup Environ Med. 2005;62(7):473-7.

44. Ki HK, Han SK, Son JS, Park SO. Risk of transmission via medical employees and importance of routine infection-prevention policy in a nosocomial outbreak of Middle East respiratory syndrome (MERS): a descriptive analysis from a tertiary care hospital in South Korea. BMC Pulm Med. 2019;19(1).

45. Wong WCW, Lee A, Tsang KK, Wong SYS. How did general practitioners protect themselves, their family, and staff during the SARS epidemic in Hong Kong? J Epidemiol Community Health. 2004;58(3):180-5.

46. Yassi A, Moore D, Fitzgerald JM, Bigelow P, Hon CY, Bryce E, et al. Research gaps in protecting healthcare workers from SARS and other respiratory pathogens: an interdisciplinary, multi-stakeholder, evidence-based approach. J Occup Environ Med. 2005;47(1):41-50.

47. DiGiovanni C, Conley J, Chiu D, Zaborski J. Factors influencing compliance with quarantine in Toronto during the 2003 SARS outbreak. Biosecur Bioterror. 2004;2(4):265-72.

48. Edeghere O, Fowler T, Wilson F, Caspa R, Raichand S, Kara E, et al. Knowledge, attitudes, experience and behaviour of frontline health care workers during the early phase of 2009 influenza A(H1N1) pandemic, Birmingham, UK. J Health Serv Res Pol. 2015;20(1):26-30.

For peer review only - http://bmjopen.bmj.com/site/about/guidelines.xhtml

49. Khalid I, Khalid TJ, Qabajah MR, Barnard AG, Qushmaq IA. Healthcare workers' emotions, perceived stressors and coping strategies during a MERS-CoV outbreak. Clin Med Res. 2016;14(1):7-14.

50. Locatelli SM, LaVela SL, Hogan TP, Kerr AN, Weaver FM. Communication and information sharing at VA facilities during the 2009 novel H1N1 influenza pandemic. Am J Infect Control. 2012;40(7):622-6.

51. Rebmann T, Wagner W. Infection preventionists' experience during the first months of the 2009 novel H1N1 influenza A pandemic. Am J Infect Control. 2009;37(10):e5-16.

52. Rowlands A. (2007). Medical social work practice and SARS in Singapore. Soc Work Health Care. 2007;45(3):57-83.

53. van Dijk CE, Hooiveld M, Jentink A, Isken LD, Timen A, Yzermans CJ. Experiences of General Practitioners and Practice Assistants during the Influenza A (H1N1) Pandemic in the Netherlands: A Cross-Sectional Survey. Plos One. 2015;10(8).

54. Kang J, Kim EJ, Choi JH, Hong HK, Han S-H, Choi IS, et al. Difficulties in using personal protective equipment: Training experiences with the 2015 outbreak of Middle East respiratory syndrome in Korea. Am J Infect Control. 2018;46(2):235-7.

55. Wong ELY, Wong SYS, Lee N, Cheung A, Griffiths, S. Healthcare workers' duty concerns of working in the isolation ward during the novel H1N1 pandemic. J Clin Nurs. 2012;21(9-10):1466-75.

56. Lam KK, Hung SYM. Perceptions of emergency nurses during the human swine influenza outbreak: A qualitative study. Int Emerg Nurs. 2013;21(4):240-6.

57. Nickell LA, Crighton EJ, Tracy CS, Al-Enazy H, Bolaji Y, Hanjrah S, et al. Psychosocial effects of SARS on hospital staff: survey of a large tertiary care institution. CMAJ. 2004;170(5):793-8.

For peer review only

58. Kang HS, Son YD, Chae S-M, Corte C. Working experiences of nurses during the Middle East respiratory syndrome outbreak. Int J Nurs Pract. 2018;24(5):e12664.

59. Khoo KL, Leng PH, Ibrahim IB, Lim TK. The changing face of healthcare worker perceptions on powered air-purifying respirators during the SARS outbreak. Respirology. 2005;10(1):107-10.

60. Straus SE, Wilson K, Rambaldini G, Rath D, Lin Y, Gold WL, et al. Severe acute respiratory syndrome and its impact on professionalism: qualitative study of physicians' behaviour during an emerging healthcare crisis. BMJ. 2004;329(7457):83.

61. Tolomiczenko GS, Kahan M, Ricci M, Strathern L, Jeney C, Patterson K, et al. SARS: Coping with the impact at a community hospital. J Adv Nurs. 2005;50(1):101-10.

62. Hsu CC, Chen T, Chang M, Chang YK. Confidence in controlling a SARS outbreak: Experiences of public health nurses in managing home quarantine measures in Taiwan. Am J Infect Control. 2006;34(4):176-81.

63. Nhan C, Laprise R, Douville-Fradet M, Macdonald ME, Quach C. Coordination and resource-related difficulties encountered by Quebec's public health specialists and infectious diseases/medical microbiologists in the management of A(H1N1) – a mixed-method, exploratory survey. BMC Public Health. 2012;12:115.

64. Rambaldini G, Wilson K, Rath D, Lin Y, Gold WL, Kapral MK, et al. The impact of severe acute respiratory syndrome on medical house staff: A qualitative study. J Gen Intern Med. 2005;20(5):381-5.

65. Robertson E, Hershenfield K, Grace SL, Stewart DE. The psychosocial effects of being quarantined following exposure to SARS: A qualitative study of Toronto health care workers. Can J Psychiatry. 2004;49(6):403-7.

66. Goulia P, Mantas C, Dimitroula D, Mantis D, Hyphantis T. General hospital staff worries, perceived sufficiency of information and associated psychological distress during the A/H1N1 influenza pandemic. BMC Infect Dis. 2010;10 (no pagination).

For peer review only

67. Carrico RM, Coty MB, Goss LK, LaJoie AS. Changing health care worker behavior in relation to respiratory disease transmission with a novel training approach that uses biosimulation. Am J Infect Control. 2007;35(1):14-9.

68. Hon C-Y, Gamage B, Bryce EA, LoChang J, Yassi A, Maultsaid D, et al. Personal protective equipment in health care: Can online infection control courses transfer knowledge and improve proper selection and use? Am J Infect Control. 2008;36(10):E33-7.

69. May L, Lung D, Harter K. An intervention to improve compliance with transmission precautions for influenza in the emergency department: Successes and challenges. J Emerg Med. 2012;42(1):79-85.

70. Hyten C, Ludwig TD. Complacency in process safety: A behaviour analysis toward prevention strategies. J Organ Behav Manage. 2017;37(3-4):240-60.

71. Haby MM, Chapman E, Clark R, Barreto J, Reveiz L, Lavis JN. What are the best methodologies for rapid reviews of the research evidence for evidence-informed decision making in health policy and practice: a rapid review. Health Res Policy Syst. 2016;14(1):83.

For peer review only

Supplementary Table I. Study characteristics

**Authors (year)**	**Country**	**Disease outbreak**	**Participants: N, age, gender**	**Professional role**	**Protective behaviours examined**
Yap et al. (2010)[4]	Singapore	H1N1	331;<br>Mean age 23.2, range 18-61;<br>7.6% female	Healthcare workers	Questions on preventive practices related to mask wearing, vaccination, avoidance behaviours, personal habits (e.g. washing hands regularly, covering mouth when coughing/sneezing), and medical attention seeking practice
Al-Amri et al. (2019)[12]	Saudi Arabia	MERS	85;<br>37.6% under 30; 49.4% aged 30-40; 12.9% aged over 40;<br>38.8% female	General practitioners (62.4%), specialists (27.1%), consultants (10.6%)	'Adherence toward guidelines' assessed through 10 statements; no further information provided
Datta et al. (2011)[13]	India	H1N1	237;<br>58.23% aged 21-25, 24.05% aged 26-30;<br>20.25% female	Tertiary care hospital staff: 113 nurses, 41 laboratory technicians, 20 auxilliary nursing midwives, 61 'other' including medical social	Frequent handwashing, use of face mask, use of gloves, closed-front gown, use of disinfectant, 'others'

For peer review only - http://bmjopen.bmj.com/site/about/guidelines.xhtml

				workers and physiotherapists	
De Perio et al. (2012)[14]	USA	H1N1	88;<br>Median age 30, range 25-53;<br>35% female	Physicians in training: 75 internal medicine residents, 7 cardiology fellows, 6 pulmonary and critical care fellows (only 53 provided sufficient information on protective behaviours)	Respiratory protection: gloves, gowns, surgical masks, fit-tested N95 respirators, powered air-purified respirators, eye protection
Manabe et al. (2012)[15]	Vietnam	Avian influenza	326;<br>Physicians: 39.8% aged 35 or under, 39.8% aged 36-50, 20.4% aged 51 or over, Nurses: 35.6% aged 35 or under, 47.5% aged 36-50, 16.9% aged 51 or over, Lab technicians: 45.5% aged 35 or under, 48.5% aged 36-50, 6.1% aged 51 or over, Other: 43.5% aged	64.7% physicians, 18.1% nurses, 10.1% laboratory technicians, 7.1% other	Wearing PPE to see suspected H5N1 patients

For peer review only

			35 or under, 47.8% aged 36-50, 8.7% aged 51 or over;<br>43.8% female (doctors), 90.0% female (nurses), 84.8% female (lab technicians), 56.5% female (other)		
Nour et al. (2015)[16]	Saudi Arabia	MERS	281;<br>Mean age 30.8, range 21-57;<br>57.7% female	46.3% nurses, 20.6% physicians, 9.6% specialists, 23.5% technicians	Using soap and water to wash hands, covering nose and mouth with a tissue when sneezing or coughing, throwing used tissues away, avoiding touching eyes/nose/mouth, using facemask in crowds, carefully handling suspected patients' belongings, healthy eating/healthy lifestyle, educating patients about disease
Nour et al. (2017)[17]	Saudi Arabia	MERS	188 of the participants in Nour et al. (2015);<br>Age and gender for this sub-group not reported	188 of the participants in Nour et al. (2015);<br>Occupational role for this sub-group not reported	As reported in Nour et al. (2015)

For peer review only - http://bmjopen.bmj.com/site/about/guidelines.xhtml

Pratt et al. (2009)[18]	Canada	SARS	536;<br>Mean age 48.4;<br>96.6% female	Hospital nurses; Primary area of practice: 26.2% medicine, surgery; 19.5% maternal newborn, paediatrics; 29.4% emergency, critical care; 24.9% other	Cleaning hands with water and soap or waterless hand rubs after removing disposable gloves, wearing disposable gloves, wearing a disposable outer garment, wearing an N95 mask, wearing protective eyewear
Evirgen et al. (2014)[19]	Turkey	H1N1	22;<br>Mean age 31.95, range 19-48;<br>59.7% female	University hospital staff: 22 professors, 29 researchers, 26 nurses; 35% from surgical departments, 32% from internal medicine departments; 17% from operation room/intensive care unit; 14% from laboratories	Total score for protective behaviours including clearing the air in the environment, washing hands with soap after practice, rubbing hands with antiseptic after practice, using disinfectant at home, H1N1 vaccination, vaccination of children, recommending vaccination to at-risk groups, using medicines supporting the immune system, using alternative medicine drugs, using antivirals for prophylactic purposes, following scientific journals about H1N1 and looking at media statements on H1N1

For peer review only

Kim & Choi (2016)[20]	South Korea	MERS	249;<br>Mean age 21.9, range 20-32;<br>88.4% female	Nursing students in third or fourth year engaged in clinical practice for 8 hours a day at tertiary hospitals with MERS patients	Nine-item scale consisting of five items about reducing the use of public places in daily life, one item about avoiding people with cough, one item about intensive cleaning and disinfection, one item about handwashing and one item about talking with people nearby
Taghrir et al. (2020)[21]	Iran	COVID-19	240;<br>Mean age 23.67;<br>59.2% female	Fifth to seventh year medical students	Preventive behaviours: cancelling social activities, not using public transport, less frequent shopping, reducing the use of closed spaces, cough etiquette, avoiding large gatherings, increased cleaning and disinfecting of surfaces, handwashing, discussing prevention with family and friends
Jeong et al. (2011)[22]	South Korea	H1N1	880;<br>76.3% aged 18-39, 22.4% aged 40-59, 1.4% 60 or over;<br>64.5% female	Hospital employees	The Korea Center of Disease Control and Prevention’s recommended behaviours, including washing hands, using a tissue when coughing or sneezing, reducing outings

For peer review only

Chor et al. (2012)[23]	China (Hong Kong), Singapore, UK	H1N1	2100 (1556 Hong Kong, 284 Singapore, 260 UK); Mean age 35 (Hong Kong), 28 (Singapore), 34 (UK); Gender not reported	Doctors, nurses, allied healthcare workers	Washing hands before each patient contact, washing hands after each patient contact, wearing gloves during patient contact, wearing a surgical mask even when not involved in direct patient care, wearing a mask during patient contact
Koh et al. (2009)[24]	Indonesia, Singapore	Avian influenza	333 from Indonesia v 1321 from Singapore plus 51 focus group participants from Indonesia; Quantitative study population: Indonesia: 55.4% aged 40 or under, Singapore: 43.6% aged 40 or under, Qualitative study: Age range 22-56; Quantitative study population: Indonesia: 75.7% female, Singapore: 86.4% female, Qualitative study: 94.1% female	Primary healthcare workers. Quantitative study: Indonesia: 17.6% doctors, 14.6% nurses, 67.8% other. Singapore: 14.6%, 34.2%, 51.2%. 'Other' includes therapists, paramedics, ambulance crew, attendants, pharmacists, social workers, cleaners and administrative staff Qualitative study: 13 physicians, 19 paramedics, 19 nurses	'Adhering to infection control protocols and recommended measures'

For peer review only

Wong et al. (2005)[25]	China (Hong Kong) (n=137), Canada (n=51)	SARS	188; Age not reported; Gender not reported	Family medicine tutors (Hong Kong), family physicians (Canada)	Testing patient temperature as a routine procedure, always wearing mask during consultations, cleaning work surface with antiseptics at least once a day, support staff all wearing masks, wearing gowns for patient encounters, washing hands between every patient, closing clinic, quarantine
Kamate et al. (2020)[26]	International (30.7% from Asia, 25.0% from North/South Americas, 16.3% from Europe, 22.6% from Africa, 5.4% from 'other' (Australia	COVID-19	860; Age reported; Gender not reported	Dental practitioners	Staff sensitised as per World Health Organization guidelines for the prevention of COVID-19, including travel history when recording patient histories, discussing risk and preventive measures related to COVID-19 with patients, showing patients signs and symptoms of COVID-19, taking 'preventive measures' against COVID-19, effect on social life

For peer review only - http://bmjopen.bmj.com/site/about/guidelines.xhtml

	and Antarctica)				
Hu et al. (2012)[27]	China	H1N1	650;<br>Median age 28, IQR 25-33;<br>76.3% female	Nurses (421), physicians (229)	Hand hygiene, gloves, gown, mask (surgical and N95 respirator), goggles
Alsahafi & Cheng (2016)[28]	Saudi Arabia	MERS	1216;<br>Age not reported;<br>56.4% female (nurses), 25.5% female (physicians), 23.9% female (other healthcare workers)	Nurses (56.3%), physicians (22%), other healthcare workers (21.7%)	Handwashing after patient contact, wearing of surgical mask during patient contact, wearing of N95 mask during patient contact, training on infectious behaviours, perceived barriers to infection control practices
Alsubaie et al. (2019)[29]	Saudi Arabia	MERS	591;<br>Age not reported;<br>78% female (non-physicians) and 31% female (physicians)	Physicians (55%), non-physicians – including nurses, technicians and respiratory therapists (45%)	Type of preventive behaviours and practices that changed during the outbreak, including hand hygiene and avoidance behaviours such as avoidance of hand-shaking, avoidance of direct contact with people suffering influenza-like illnesses and avoidance of public facilities

For peer review only - http://bmjopen.bmj.com/site/about/guidelines.xhtml

Chau et al. (2008)[30]	China (Hong Kong)	SARS	96 (observational study), 109 (interviews);<br>Age not reported;<br>Gender not reported	Healthcare workers and support workers; for interviews: 32 healthcare workers, 30 health care assistants, 47 managers including nursing officers, ward managers, nurse specialists and infection control nurses	Total performance score based on compliance with hand hygiene, mask use, gown use, goggle/face shield precautions, glove use, handling patient care equipment and articles correctly, routine and terminal cleaning, handling linen and laundry, and terminal cleaning of isolation room
Koh et al. (2005)[31]	Singapore	SARS	15,025;<br>Mean age 36.6;<br>82% female	Healthcare workers from nine major healthcare institutions, incl. doctors, nurses, physiotherapists, attendants, cleaners, pharmacists, managerial staff, clerical staff, service counter staff, other	Hospital's recommended protective practices
May et al. (2010)[32]	USA	H1N1	261;<br>Students: 26% aged under 24, 61% aged 24-29, 10% aged 30-34, 3% 35 and	Medical students (194) and residents (67), at an urban medical teaching centre	Hand sanitiser, handwashing, cough etiquette, disinfection, surgical mask when ill with influenza-like illness, surgical mask in patient care when

For peer review only - http://bmjopen.bmj.com/site/about/guidelines.xhtml

			over, Residents: 55% aged 24-29, 36% aged 30-34, 9% 35 and over; Students: 62% female, Residents: 50% female		seeing patients with influenza-like illness, N95 when seeing patients with influenza-like illness, social distancing
Mitchell et al. (2012)[33]	Canada	H1N1	986; Median age 36, range 21-79; 85% female	Nurses (80%), respiratory therapists (14%), physicians (6%)	Use of PPE
Parker & Goldman (2006)[34]	Canada	SARS	116; 27% aged 20-29, 42% aged 30-39, 23% aged 40-49, 8% aged 50 and over; 70% female	Paediatric emergency department staff: 28% physicians, 41% nursing staff, 21% trainees, 10% paramedical staff involved with direct patient care	Wearing N95 mask when examining patients, wearing gown when examining patients, handwashing before and after patient contact, wearing N95 mask at all times in the emergency department, wearing eye protection when examining patients, wearing gloves when examining patients
Shigayeva et al. (2007)[35]	Canada	SARS	795; Median age 41, range 21-67; 74.3% female	46.3% nurses, 13.5% physicians, 14.2% respiratory therapists, 10.3% medical imaging	Use of barrier precautions, practices for removing PPE, infection control training

For peer review only - http://bmjopen.bmj.com/site/about/guidelines.xhtml

				technologists, 15.7% other incl. service assistants, physiotherapists, lab technologists, paramedics, housekeeping staff, ward clerks and social workers	
Vinck et al. (2011)[36]	Netherlands	H1N1	166;<br>4% aged 25 or under, 26% aged 26-35, 26% aged 36-45, 32% aged 46-55, 10% aged 56-65;<br>66% female	Public health physicians (46%), public health nurses (51%), health department managers (2%)	Compliance with control measures: applying case definition (i.e. how consistently staff applied the criteria for case definition that was issued to identify suspected patients), amount of consultation with the centralised assessment system for the final classification of patients, use of PPE during patient sampling and home visits, and informing patients and contacts about isolation measures
Alshammari et al. (2018)[37]	Saudi Arabia	MERS	87;<br>Age not reported;<br>Gender not reported	Physicians and nurses	Hand hygiene compliance

For peer review only

Jaeger et al. (2011)[38]	USA	H1N1	63;<br>Median age 35, range 19-74;<br>76% female	70% worked in inpatient settings. 38% clinical practitioner, 10% physician/nurse practitioner/physician assistant, 29% Registered nurse/Registered Dietician, 52% allied health staff (incl. radiology, emergency department and respiratory technicians, phlebotomists, licensed vocational nurses and medical and nursing assistants), 10% support staff (incl. office workers, patient transport and environmental services workers)	PPE use, glove use during index patient exposures, mask or N95 respirator use during index patient encounters

For peer review only - http://bmjopen.bmj.com/site/about/guidelines.xhtml

For peer review only

Moore et al. (2005)[39]	Canada	SARS	97;<br>Mean age 43.1, range 26-64;<br>78% female	34% managers, 24% registered nurses, 9% support staff, 8% medical technologists, 6% respiratory therapists, 4% infection control practitioners, 4% physicians, 1% administration staff, 1% pharmacists and 1% physiotherapists	Experience of infection control practices including PPE
Tan et al. (2006)[40]	Singapore	SARS	8;<br>Age not reported;<br>25% female	Family physicians	Participants discussed encounters with suspected SARS patients including their clinical assessment and management of patients in clinics, problems encountered, and use of PPE and other preventive measures
Corley et al. (2010)[41]	Australia	H1N1	32 (questionnaire study), 16 (focus groups)<br>Age not reported;<br>Gender not reported	Questionnaire: 28 nursing staff, 4 medical staff;<br>Focus groups: 4 senior medical staff, 4 senior	How resources were managed during the pandemic

For peer review only

				nursing staff, 8 bedside nursing staff	
Hsu et al. (2011)[42]	Singapore	H1N1	123;<br>Age not reported;<br>Gender not reported	Final-year medical students	Hand hygiene
Chia et al. (2005)[43]	Singapore	SARS	6,198;<br>Mean age 35.8 (doctors), 34.6 (nurses), 38.4 (clerical staff);<br>27.1% female (doctors), 48.8% female (nurses), 77.1% female (clerical staff)	Doctors (14.1%), nurses (4404 – 71%), clerical staff (14.9%)	Perception of risk of infection and preventive measures
Ki et al. (2019)[44]	South Korea	MERS	446;<br>Age not reported;<br>Gender not reported	Medical employees; 228 in the emergency department, 218 in general wards	PPE (face shields or goggles, surgical masks, N95 respirators, gloves, disposable gowns, disposable coveralls, waterproof boots, head cover), hand-washing, hand-rubbing, isolation
Wong et al. (2004)[45]	China (Hong Kong)	SARS	320;<br>Mean age of general practitioners 44.4, mean	General practitioners (137), family medicine tutors (183)	Clinical practices and behaviours: wearing mask during all consultations, wearing goggles, wearing gowns,

For peer review only - http://bmjopen.bmj.com/site/about/guidelines.xhtml

			age of family medicine tutors not reported; 17.6% female (general practitioners), 15.3% female (family medicine tutors)		washing hands after seeing patients, insisting patients wear masks, routinely testing patients' temperatures, keeping greater distance from patients, seeing patients at faster pace, avoiding physical examination, requesting more blood tests/chest radiographs, over-prescribing antibiotics, cancelling own appointments, cancelling specialist appointments, difficulties making specialty referrals, closing clinic, reducing working hours, quarantine, requiring staff to wear masks/disposable gloves, cleaning work surfaces with antiseptics, taking staff temperatures, quarantined leave for staff, showering before leaving work, washing hands before entering home, using disinfectant to clean home, staying away from home, wearing a mask at home, sending family away

For peer review only

Yassi et al. (2005)[46]	Canada	SARS	Participants were those reported in Moore et al. (2005)	Participants were those reported in Moore et al. (2005)	Participants discussed barriers to wearing PPE and following infection control procedures
DiGiovanni et al. (2004)[47]	Canada	SARS	195;<br>Age not reported;<br>Gender not reported	76 physicians, 52 nurses, 67 'other' including receptionists, technicians, social workers, research assistants and clinic/office managers	Quarantine
Edeghere et al. (2015)[48]	UK	H1N1	20;<br>Mean age 43.9;<br>80% female	Frontline healthcare workers: 10 clinical attendants, 7 nurses, 3 general practitioners	Use of PPE and infection control measures
Khalid et al. (2016)[49]	Saudi Arabia	MERS	117;<br>Mean age 38.55;<br>76% female	Healthcare workers who worked in high-risk areas. 76% nurses, 14% physicians, 10% respiratory therapists. 65% worked in critical care units, 20% in emergency	Perceived stressors

For peer review only - http://bmjopen.bmj.com/site/about/guidelines.xhtml

				medicine, 15% in outpatient family medicine	
Locatelli et al. (2012)[50]	USA	H1N1	33;<br>Age not reported;<br>Gender not reported	Infection control key informants at nationally dispersed Veterans Affairs healthcare facilities	Communication about infection control
Rebmann & Wagner (2009)[51]	USA (n=37), UK (n=1, Canada (n=1), Australia (n=1)	H1N1	40;<br>2.5% aged 30-39, 27.5% aged 40-49, 45.0% aged 50-59, 22.5% aged 60-69, 2.5% 70 or over;<br>92% female	Infection preventionists	Issues relating to infection prevention guidance and PPE
Rowlands (2007)[52]	Singapore	SARS	28;<br>Age not reported;<br>75% female	Medical social workers	Experiences of working during the SARS crisis, and the various infection control strategies used at work
Van Dijk et al. (2015)[53]	Netherlands	H1N1	875;<br>General practitioners: 17.5% under 40, 24.5% aged 40-49, 44.9% aged 50-59, 13.2% aged 60+;	General practitioners (372), practice assistants (503)	Experience and acceptance of the national guidelines for infection control

For peer review only - http://bmjopen.bmj.com/site/about/guidelines.xhtml

			age for practice assistants not reported<br>General practitioners: 39.3% female; gender of practice assistants not reported		
Kang et al. (2018)[54]	South Korea	MERS	7;<br>Age not reported;<br>Gender not reported	Infection control nurse leaders from major hospitals	Use of PPE equipment
Wong et al. (2012)[55]	China (Hong Kong)	H1N1	10;<br>Mean age 34.9, range 24-49;<br>70% female	4 physicians, 3 nurses, 3 healthcare assistants all working in isolation wards	Concerns relating to care of patients with H1N1
Lam & Hung (2013)[56]	China (Hong Kong)	H1N1	10;<br>20% aged 20-25, 20% aged 26-30, 20% aged 31-35, 30% aged 36-40, 10% over 40;<br>100% female	Emergency nurses: 80% staff nurse, 10% advanced practice nurse, 10% nursing officer	Use of PPE
Nickell et al. (2004)[57]	Canada	SARS	2001;	33.1% allied health care professionals, 31.9% areas	Precautionary measures in the workplace: mask, restricted access (own

For peer review only

			13.6% aged under 30, 25.7% aged 30-39, 30.6% aged 40-49, 30.2% 50 or over; 78.8% female	other than patient care e.g. administration, food services, maintenance or research, 25.6% nurses, 9.3% doctors	hospital), restricted meetings, restricted access (other hospitals), gloves, goggles, ‘other’
Kang et al. (2018)[58]	South Korea	MERS	27; Mean age 29.5; 92.6% female	Nurses	Working experiences and challenges faced during the outbreak
Khoo et al. (2005)[59]	Singapore	SARS	51; Age not reported; Female:male ratio was 2:1	19 doctors, 31 nurses/nursing aides, 1 respiratory therapist	Opinions on 3M powered air-purifying respirator and Stryker powered air-purifying respirator
Straus et al. (2004)[60]	Canada	SARS	14; Age not reported; Gender not reported	Physicians from divisions of infectious diseases, general internal medicine, and critical care medicine at university hospitals	Participants discussed barrier precautions such as protective clothing
Tolomiczenko et al. (2005)[61]	Canada	SARS	300; Mean age 42.6, range 20-75; 73.9% female	Registered nurses (25.2%), physicians (20.7%), management (9.5%), services (facilities management, food	Participants discussed patient care and communication about directives

For peer review only - http://bmjopen.bmj.com/site/about/guidelines.xhtml

				services, environmental – 3.7%), health professional (occupational therapists, physiotherapists, speech-language pathologists – 17.0%), other (counsellors, technicians, clerks, chaplains – 23.8%)	
Hsu et al. (2006)[62]	Taiwan	SARS	312;<br>4.0% under 30, 32.9% aged 30-39, 48.5% aged 40-49, 14.6% 50 over over;<br>Gender not reported	Public health nurses	Self-protection equipment
Nhan et al. (2012)[63]	Canada	H1N1	129;<br>Age not reported;<br>Gender not reported	68 infectious diseases specialist/medical microbiologists, 61 public health and preventive medicine specialists	Difficulties and frustrations experienced during the pandemic
Rambaldini et al. (2005)[64]	Canada	SARS	17;<br>Age not reported;	Medical house staff allocated to a general	Participants discussed hospitals' communication of directives

For peer review only - http://bmjopen.bmj.com/site/about/guidelines.xhtml

			Gender not reported	internal medicine clinical teaching unit, infectious disease unit, infectious diseases consultation service, or intensive care unit	
Robertson et al. (2004)[65]	Canada	SARS	10;<br>Mean age 43.1;<br>60% female	4 nurses, 1 hospital executive, 1 dentist, 1 social worker, 1 occupational therapist, 1 paramedic, 1 physician	Experiences of quarantine and perceptions of infection control guidelines
Goulia et al. (2010)[66]	Greece	H1N1	469;<br>Mean age 38.4;<br>68.4% female.	Nurses (44.6%), medical staff (25.6%), allied staff (12.6%), auxiliary staff (17.3%)	Restriction of social contacts, isolation, intended work avoidance, sense of duty

For peer review only

Supplementary Table II. Summary of themes

**Theme**	**Significant association with protective behaviours**	**No significant association with protective behaviours**	**Reported, but not statistically analysed as a potential predictor of protective behaviours**
Socio-demographics and personal characteristics			
Age	Kim & Choi (2016)[20]	Al-Amri et al. (2019)[12]<br>Datta et al. (2011)[13]<br>De Perio et al. (2012)[14]<br>Manabe et al. (2012)[15]<br>Nour et al. (2015)[16]<br>Nour et al. (2017)[17]<br>Pratt et al. (2009)[18]	N/A
Gender	Nour et al. (2015)[16]<br>Jeong et al. (2011)[22]<br>Vinck et al. (2011)[36]	Al-Amri et al. (2019)[12]<br>Datta et al. (2011)[13]<br>De Perio et al. (2012)[14]<br>Nour et al. (2017)[17]<br>Evirgen et al. (2014)[19]<br>Kim & Choi (2016)[20]	N/A

For peer review only

		Taghrir et al. (2020)[21]	
Nationality	Al-Amri et al. (2019)[12]	N/A	N/A
Country	Chor et al. (2012)[23]<br>Koh et al. (2009)[24]*<br>Wong et al. (2005)[25]*<br>Kamate et al. (2020)[26]*	Koh et al. (2009)[24]*<br>Wong et al. (2005)[25]*<br>Kamate et al. (2020)[26]*	N/A
Religion	N/A	Kim & Choi (2016)[20]	N/A
Socio-economic status	Jeong et al. (2011)[22]	N/A	N/A
Marital status	N/A	Pratt et al. (2009)[18]<br>Evirgen et al. (2014)[19]	N/A
Level of education	Al-Amri et al. (2019)[12]	Datta et al. (2011)[13]<br>Pratt et al. (2009)[18]<br>Kim & Choi (2016)[20]<br>Taghrir et al. (2020)[21]	N/A
Personal vulnerabilities	N/A	Evirgen et al. (2014)[19]	N/A
Vulnerable people at home	Evirgen et al. (2014)[19]*	Evirgen et al. (2014)[19]*	N/A
Vaccination status	Hu et al. (2012)[27]	N/A	N/A
Occupational role			

For peer review only - http://bmjopen.bmj.com/site/about/guidelines.xhtml

Role	Al-Amri et al. (2019)[12]<br>Pratt et al. (2009)[18]<br>Evirgen et al. (2014)[19]<br>Chor et al. (2012)[23]<br>Alsahafi & Cheng (2016)[28]<br>Alsubaie et al. (2019)[29]<br>Chau et al. (2008)[30]<br>Koh et al. (2005)[31]<br>Mitchell et al. (2012)[33]<br>Parker & Goldman (2006)[34]*<br>Shigayeva et al. (2007)[35]<br>Vinck et al. (2011)[36]<br>Chia et al. (2005)[43]	Datta et al. (2011)[13]<br>Nour et al. (2015)[16]<br>Nour et al. (2017)[17]<br>Parker & Goldman (2006)[34]*<br>Alshammari et al. (2018)[37]<br>Jaeger et al. (2011)[38]	May et al. (2010)[32]
Length of experience	Koh et al. (2005)[31]<br>Mitchell et al. (2012)[33]<br>Vinck et al. (2011)[36]	Al-Amri et al. (2019)[12]<br>Nour et al. (2015)[16]<br>Nour et al. (2017)[17]<br>Pratt et al. (2009)[18]<br>Chau et al. (2008)[30]	N/A
Training and knowledge			

For peer review only - http://bmjopen.bmj.com/site/about/guidelines.xhtml

Receiving training/education	May et al. (2010)[32]*<br>Shigayeva et al. (2007)[35]	Nour et al. (2017)[17]<br>Taghrir et al. (2020)[21]<br>May et al. (2010)[32]*	Alsahafi & Cheng (2016)[28]<br>Moore et al. (2005)[39]<br>Tan et al. (2006)[40]<br>Corley et al. (2010)[41]
Sources of knowledge	Al-Amri et al. (2019) [12]	Kim & Choi (2016)[20]<br>Taghrir et al. (2020)[21]	N/A
Knowledge (of correct practices / outbreak)	Yap et al. (2010)[4]<br>De Perio et al. (2012)[14] *<br>Nour et al. (2015)[16]<br>Kim & Choi (2016)[20]*<br>Jeong et al. (2011)[22]<br>Hu et al. (2012)[27]	Al-Amri et al. (2019)[12]<br>De Perio et al. (2012)[14] *<br>Kim & Choi (2016)[20]*	Alsahafi & Cheng (2016) [28]<br>Hsu et al. (2011)[42]
Work-related factors			
Setting	De Perio et al. (2012)[14]<br>Chau et al. (2008)[30]<br>Mitchell et al. (2012)[33]<br>Shigayeva et al. (2007)[35]<br>Jaeger et al. (2011)[38]<br>Chia et al. (2005)[43]<br>Ki et al. (2019)[44]*<br>Wong et al. (2004)[45]	Evirgen et al. (2014)[19]<br>Taghrir et al. (2020)[21]<br>Ki et al. (2019)[44]*	N/A

For peer review only - http://bmjopen.bmj.com/site/about/guidelines.xhtml

Contact with confirmed cases	De Perio et al. (2012)[14]<br>Pratt et al. (2009)[18]*<br>Shigayeva et al. (2007)[35]<br>Chia et al. (2005)[43]<br>Wong et al. (2004)[45]	Pratt et al. (2009)[18]*<br>Evirgen et al. (2014)[19]	N/A
Workload	Pratt et al. (2009)[18]<br>Chor et al. (2012)[23]*<br>Shigayeva et al. (2007)[35]<br>Vinck et al. (2011)[36]	Chor et al. (2012)[23]*	Chau et al. (2008)[30]<br>Moore et al. (2005)[39]<br>Corley et al. (2010)[41]<br>Yassi et al. (2005)[46]
Monitoring of compliance	Hu et al. (2012)[27]+	N/A	Hu et al. (2012)[27]+<br>Hsu et al. (2011)[42]<br>DiGiovanni et al. (2004)[47]
Patient encounters	De Perio et al. (2012)[14]<br>Shigayeva et al. (2007)[35]	N/A	N/A
Personal protective behaviour-related factors			
Availability of resources	Hu et al. (2012)[27]+<br>Mitchell et al. (2012)[33]*	Mitchell et al. (2012)[33]*	De Perio et al. (2012)[14]<br>Hu et al. (2012)[27]+<br>Moore et al. (2005)[39]<br>Tan et al. (2006)[40]

For peer review only

For peer review only - http://bmjopen.bmj.com/site/about/guidelines.xhtml

			Corley et al. (2010)[41]<br>Edeghere et al. (2015)[48]<br>Khalid et al. (2016)[49]<br>Locatelli et al. (2012)[50]<br>Rebmann & Wagner (2009)[51]<br>Rowlands (2007)[52]<br>Van Dijk et al. (2015)[53]<br>Hsu et al. (2006)[62]
Perceived difficulty of protective behaviour	N/A	N/A	Van Dijk et al. (2015)[53]<br>Kang et al. (2018)[54]
Logistical issues	N/A	N/A	Rebmann & Wagner (2009)[51]<br>Kang et al. (2018)[54]<br>Wong et al. (2012)[55]
Perceived effectiveness	Yap et al. (2010)[4]<br>Hu et al. (2012)[27]<br>Mitchell et al. (2012)[33]<br>Parker & Goldman (2006)[34]*	Parker & Goldman (2006)[34]*	Yassi et al. (2005)[46]<br>Kang et al. (2018)[54]
Perceived importance	N/A	N/A	Vinck et al. (2011)[36]<br>Hsu et al. (2011)[42]

For peer review only

Incentives	N/A	N/A	Jeong et al. (2011)[22]<br>Hsu et al. (2011)[42]<br>Kang et al. (2018)[54]
Convenience	N/A	N/A	De Perio et al. (2012)[14]<br>Hu et al. (2012)[27]<br>Moore et al. (2005)[39]<br>Tan et al. (2006)[40]<br>Corley et al. (2010)[41]<br>Hsu et al. (2011)[42]<br>Kang et al. (2018)[54]<br>Lam & Hung (2013)[56]<br>Nickell et al. (2004)[57]
Comfort	Mitchell et al. (2012)[33]*	Mitchell et al. (2012)[33]*	Moore et al. (2005)[39]<br>Tan et al. (2006)[40]<br>Corley et al. (2010)[41]<br>Yassi et al. (2005)[46]<br>Khalid et al. (2016)[49]<br>Rowlands (2007)[52]<br>Lam & Hung (2013)[56]<br>Nickell et al. (2004)[57]<br>Kang et al. (2018)[58]

For peer review only - http://bmjopen.bmj.com/site/about/guidelines.xhtml

			Khoo et al. (2005)[59]<br>Straus et al. (2004)[60]
Impact on patient care	Hu et al. (2012)[27]+	N/A	Hu et al. (2012)[27]+<br>Tan et al. (2006)[40]<br>Corley et al. (2010)[41]<br>Rowlands (2007)[52]<br>Lam & Hung (2013)[56]<br>Nickell et al. (2004)[57]<br>Khoo et al. (2005)[59]<br>Straus et al. (2004)[60]<br>Tolomiczenko et al. (2005)[61]
Guidance			
Issues with availability or understanding	N/A	N/A	Alsahafi & Cheng (2016)[28]<br>Chau et al. (2008)[30]<br>Moore et al. (2005)[39]<br>Tan et al. (2006)[40]<br>Corley et al. (2010)[41]<br>Yassi et al. (2005)[46]<br>Locatelli et al. (2012)[50]<br>Rebmann & Wagner (2009)[51]<br>Van Dijk et al. (2015)[53]

For peer review only

			Kang et al. (2018)[54]<br>Wong et al. (2012)[55]<br>Lam & Hung (2013)[56]<br>Kang et al. (2018)[58]<br>Tolomiczenko et al. (2005)[61]<br>Hsu et al. (2006)[62]<br>Nhan et al. (2012)[63]<br>Rambaldini et al. (2005)[64]<br>Robertson et al. (2004)[65]
Distress and risk perception			
Distress	Chia et al. (2005)[43]<br>Wong et al. (2004)[45]	N/A	DiGiovanni et al. (2004)[47]
Risk perception	Kim & Choi (2016)[20]<br>Taghrir et al. (2020)[21]<br>Jeong et al. (2011)[22]<br>Chor et al. (2012)[23]*<br>Parker & Goldman (2006)[34]<br>Goulia et al. (2010)[66]	Chor et al. (2012)[23]*	Moore et al. (2005)[39]<br>DiGiovanni et al. (2004)[47]<br>Rebmann & Wagner (2009)[51]
Attitudes and behaviours of others			

For peer review only - http://bmjopen.bmj.com/site/about/guidelines.xhtml

Non-compliance of others	N/A	N/A	Hu et al. (2012)[27]<br>Chau et al. (2008)[30]<br>Moore et al. (2005)[39]<br>Hsu et al. (2011)[42]<br>Yassi et al. (2005)[46]<br>DiGiovanni et al. (2004)[47]<br>Locatelli et al. (2012)[50]
Attitudes of family members	N/A	N/A	Moore et al. (2005)[39]<br>Yassi et al. (2005)[46]
Other potential predictors of compliance			
Complacency	N/A	N/A	Tan et al. (2006)[40]
Commitment	N/A	N/A	Alsahafi & Cheng (2016)[28]<br>Moore et al. (2005)[39]
Previous experience with pandemic	N/A	N/A	Moore et al. (2005)[39]
Passing of time	N/A	N/A	Rebmann & Wagner (2009)[51]
Forgetting	Hu et al. (2012)[27]	N/A	N/A
Death of a doctor	N/A	N/A	Tan et al. (2006)[40]

For peer review only - http://bmjopen.bmj.com/site/about/guidelines.xhtml

Effort-reward imbalance	Pratt et al. (2009)[18]	N/A	N/A
Consulting with experts	N/A	Manabe et al. (2012)[15]	N/A
Lack of staff	N/A	N/A	Moore et al. (2005)[39]<br>Corley et al. (2010)[41]
PPE fit-testing procedures	N/A	N/A	Moore et al. (2005)[39]
Professionalism	N/A	N/A	Yassi et al. (2005)[46]
Use of public transport	N/A	Evirgen et al. (2014)[19]	N/A
Loss of income	N/A	N/A	DiGiovanni et al. (2004)[47]
Overcrowding	N/A	N/A	Alsahafi & Cheng (2016)[28]

*Appears in both ‘significant association’ and ‘no significant association’ column due to both significant and non-significant results being reported for different groups of participants.
+Contains both statistically analysed data and non-statistically analysed (e.g. qualitative data, descriptive survey results).

For peer review only

Supplementary Table III. Summary of evidence

Theme	Evidence
Socio-demographics and personal characteristics	
Age	Al-Amri et al. (2019)[12]: Compliance was not significantly associated with age (p=0.785).
	Datta et al. (2011)[13]: Protective behaviours were not significantly associated with age.
	De Perio et al. (2012)[14]: Compliance was not significantly associated with age.
	Manabe et al. (2012)[15]: For physicians only (other roles not included in analysis), age was not significantly associated with wearing PPE to see suspected avian influenza patients.
	Nour et al. (2015)[16]: There was no significant difference in median protective practice scores between age groups (under 30s v 30 and over) (p=0.582).
	Nour et al. (2017)[17]: There was no significant difference in median protective practice scores between age groups (under 30s v 30 and over).
	Pratt et al. (2009)[18]: Age was not significantly associated with compliance.
	Kim & Choi (2016)[20]: Age was significantly positively correlated with preventive behaviours (p<0.05); older staff were more likely to comply.
Gender	Al-Amri et al. (2019)[12]:Compliance was not significantly associated with gender (p=0.241).
	Datta et al. (2011)[13]: Protective behaviours were not significantly associated with gender.
	DePerio et al. (2012)[14]: Compliance was not significantly associated with gender.

For peer review only - http://bmjopen.bmj.com/site/about/guidelines.xhtml

	Nour et al. (2015)[16]: Female staff were significantly more likely to comply with preventive behaviours (p<0.001).
	Nour et al. (2017)[17]: There was no significant difference in median protective practice scores between genders.
	Evirgen et al. (2014)[19]: Behaviour was not significantly associated with gender.
	Kim & Choi (2016)[20]: Gender was not significantly associated with preventive behaviours.
	Taghrir et al. (2020)[21]: There was no significant association between preventive behaviours and gender.
	Jeong et al. (2011)[22]: Female gender was a significant predictor of preventive behaviours.
	Vinck et al. (2011)[36]: Female staff reported higher compliance than males (p=0.002) and greater use of PPE (p=0.037).
Nationality	Al-Amri et al. (2019)[12]:Compliance was significantly associated with nationality (p=0.047), with Saudi staff showing higher compliance than non-Saudi staff in the same city.
Country	Chor et al. (2012)[23]: Hong Kong reported the highest compliance with all infection control practices, except for wearing a mask during patient contact, which was second to Singapore (70.4% v 87.7%). Wearing masks in clinical areas while not involved with patient contact was lowest for the UK (25.3% v 96.4% for Hong Kong and 82.3% for Singapore), as was use of masks (either surgical or N95) during physical contact with patients (62% for the UK v 70.4% for Hong Kong and 87.7% for Singapore).
	Koh et al. (2009)[24]: Healthcare workers in Singapore were significantly more likely than those in Indonesia to screen patients and clinic visitors for fever (92.9% v 80.8%, p<0.001), limit number of visitors to the clinic (88.7% v 46.0%, p<0.001), post prominent infection control notices to patients/visitors (97.4% v 80.3%, p<0.001), do temperature checks for staff (91.5% v 61.1%, p<0.001); wear gowns (91.1% v 61.5%, p<0.001); wear paper masks (43.2% v 11.7%, p<0.001); wear surgical masks (28.6% v 12.6%, p<0.001); wear N95 masks (94.0% v 82.4%, p=0.004); and carry out regular handwashing (98.6% v 87.0%, p<0.001). However, there were no significant differences between countries in wearing gloves (94.2% v 93.3%, p=0.502); wearing goggles (77.6% v 67.8%, p=0.140); using powered air purifier

For peer review only

For peer review only - http://bmjopen.bmj.com/site/about/guidelines.xhtml

	respirators (86.1% v 85.4%, p=0.550); using alcohol hand rub (95.6% v 90.8%, p=0.183); or using a special room to isolate bird flu patients (97.9% v 97.5%, p=0.254).
	Wong et al. (2005)[25]: Physicians in Hong Kong were less likely than those in Canada to quarantine themselves after contact with a probable or suspected SARS case (19.4% v 77.1%, p<0.01) or give quarantine leave to their staff (59.7% v 95%, p<0.01). However, physicians in Hong Kong were more likely than those in Canada to test patient temperature as a routine procedure (68.1% v 47.1%, p<0.01), wear a mask during consultations (97.7% v 52.9%, p<0.01) and have their support staff wear masks (97.8% v 68.6%, p<0.01). No significant differences between countries were found for wearing gowns for every patient encounter or washing hands between every encounter.
	Kamate et al. (2020)[26]: There were no significant differences between countries in terms of discussing risk of COVID-19 with patients; discussing preventive measures with patients; showing patients the signs and symptoms of COVIF-19; or taking preventive measures themselves. However, there was a significant difference in whether they included travel history while recording patients' histories (99.0% in Asia v 97.2% in Americas v 94.3% in Europe v 94.3% in Africa v 89.4% in Australia/Antarctica, p=0.025).
Religion	Kim & Choi (2016)[20]: Being religious or not was not significantly associated with preventive behaviours.
Socio-economic status	Jeong et al. (2011)[22]: 'High or middle economic state' significantly predicted preventive behaviours.
Marital status	Pratt et al. (2009)[18]: Marital status was not significantly associated with compliance.
	Evirgen et al. (2014)[19]: Behaviour was not significantly associated with marital status.

For peer review only - http://bmjopen.bmj.com/site/about/guidelines.xhtml

Level of education	Al-Amri et al. (2019)[12]: Compliance was significantly associated with qualifications (p=0.037) with more highly-qualified staff being more likely to comply. Percentage of poor, good and excellent preventive practices, by qualification: MBBS: 30.6%, 69.4%, 0.0%; Diploma: 46.7%, 46.7%, 6.7%; Masters: 43.8%, 43.8%, 12.5%; Doctorate/fellowship: 66.7%, 22.2%, 11.1%.
	Datta et al. (2011)[13]: Protective behaviours were not significantly associated with level of education.
	Pratt et al. (2009)[18]: Highest level of education was not significantly associated with compliance.
	Kim & Choi (2016)[20]: Level of education (junior/senior grade) was not significantly associated with compliance.
	Taghrir et al. (2020)[21]: There was no significant association between preventive behaviours and education level.
Personal vulnerabilities	Evirgen et al. (2014)[19]: Behaviour was not significantly associated with being pregnant or having a chronic disease.
Vulnerable people at home	Evirgen et al. (2014)[19]: Behaviour was not significantly associated with having a pregnant spouse, elderly person, or school-aged child at home. However, staff with babies at home were more likely to comply with protective behaviours (p<0.005).
Vaccination status	Hu et al. (2012)[27]: Vaccination for H1N1 influenza was an independent predictor of high compliance to PPE (p<0.001).
Occupational role	
Role	Al-Amri et al. (2019)[12]: Practice-related adherence to guidelines differed significantly according to position (p=0.035), with specialists having the highest percentage of excellent practice.
	Datta et al. (2011)[13]: Protective behaviours were not significantly associated with occupation.
	Nour et al. (2015)[16]: There was no significant difference in median protective practice scores between occupational groups (p=0.433).

For peer review only - http://bmjopen.bmj.com/site/about/guidelines.xhtml

	Nour et al. (2017)[17]: There was no significant difference in median protective practice scores between occupational groups (nurses v physicians v specialists v technicians).
	Pratt et al. (2009)[18]: Job status (full-time, part-time or casual) was reported to be significantly associated with compliance although the authors do not state which group were more likely to comply. Medical/surgical unit as a primary area of practice (compared to maternal/newborn or paediatrics, emergency or critical care, or 'other' roles) was also reported to be significantly associated with compliance but again the authors do not state in which way.
	Evirgen et al. (2014)[19]: Professors had the highest average score for positive behaviours.
	Chor et al. (2012)[23]: In Hong Kong and Singapore, employment type was the strongest predictor of adoption of infection control practices, with nurses showing significantly higher compliance with most measures compared to doctors in both countries, and allied healthcare workers also reporting significantly higher compliance with most measures compared to doctors in Hong Kong. Administrative staff in Hong Kong reported compliance similar to that of other healthcare workers, whereas administrative staff in the UK reported significantly lower compliance than doctors.
	Alsahafi & Cheng (2016)[28]: There appeared to be significant differences between physicians, nurses and other healthcare staff in terms of handwashing after patient contact ($p=0.04$), wearing of surgical mask during patient contact ($p<0.001$), and wearing of N95 mask during patient contact ($p=0.009$). It is not clear from the data presented which groups are significantly more likely to comply, but perhaps nurses are slightly more likely to comply than physicians who are slightly more likely to comply than other healthcare staff.
	Alsubaie et al. (2019)[29]: Non-physicians were significantly more likely than physicians to report improvement (that is, improvement since pre-outbreak) in hand hygiene compliance at the hospital (90% v 80%, $p<0.002$), improvement in compliance with universal precautions at the hospital (85.5% v 69%, $p=0.001$), increase in avoidance behaviour towards people with flu-like symptoms (83% v 72%, $p<0.009$), decrease in using public facilities (66% v 28.7%,

For peer review only - http://bmjopen.bmj.com/site/about/guidelines.xhtml

	p=0.001), decrease in hand-shaking (60% v 27.7%, p<0.001), and decrease in social visits e.g. to friends (60% v 21.7%, p<0.001).
	Chau et al. (2008)[30]: Nurses performed better in a number of isolation precautions (including hand-washing, hand rub use, glove use, handling of linen and laundry, and handling of patient care equipment) than other roles. Doctors performed better when it came to wearing masks and putting on gowns.
	Koh et al. (2005)[31]: All other occupational groups, including nurses (OR 0.49; 95% CI 0.39-0.61) reported greater difficulty adhering to preventive measures compared with doctors.
	May et al. (2010)[32]: Medical students appeared more likely than residents to use hand sanitiser both at work and home, wash hands both at work and home, and practice cough etiquette at work and home, while residents appeared more likely to use disinfectant and use surgical masks and N95 masks when seeing patients with influenza-like illness – however, no statistical analysis is presented.
	Mitchell et al. (2012)[33]: Compared with physicians, nurses and respiratory therapists were significantly more compliant with wearing N95 respirators and gloves for aerosol-generating medical procedures and with wearing protective eyewear and gloves for patient care not involving these procedures. Nurses and respiratory therapists were also significantly more compliant with performing a user seal check after donning an N95 respirator than physicians (35% and 40% v 19%, p=0.023).
	Parker & Goldman (2006)[34]: Physicians reported more handwashing before and after all patient contacts compared with nurses and trainees (p<0.05); however, there were no significant differences between in occupational groups in compliance with wearing an N95 mask when examining patients, wearing a gown when examining patients, wearing an N95 mask at all times in the emergency department, wearing eye protection when examining patients, or wearing gloves when examining patients.

For peer review only

For peer review only - http://bmjopen.bmj.com/site/about/guidelines.xhtml

	Shigayeva et al. (2007)[35]: Clinically trained health professional status was reported to be a significant predictor of consistent adherence (statistics not reported). Safe removal of PPE was significantly associated with being a nurse (OR 1.3, 95% CI, 1.1-1.6).
	Vinck et al. (2011)[36]: Public health physicians followed the case definition less strictly than public health nurses (p=0.000).
	Alshammari et al. (2018)[37]: No significant difference in hand hygiene compliance between physicians and nurses.
	Jaeger et al. (2011)[38]: Use of PPE stratified by healthcare role did not yield significant differences.
	Chia et al. (2005)[43]: The risk of using a less effective level of respiratory protection was lowest among doctors, followed by nurses and clerical staff.
Length of experience	Al-Amri et al. (2019)[12]:Compliance was not significantly associated with length of experience in primary care (p=0.265).
	Nour et al. (2015)[16]: There was no significant association between median protective practice scores and years of experience (p=0.674).
	Nour et al. (2017)[17]: There was no significant association between median protective practice scores and years of experience.
	Pratt et al. (2009)[18]: Years of nursing experience were not significantly associated with compliance.
	Chau et al. (2008)[30]: Compliance was not significantly associated with years of experience.
	Koh et al. (2005)[31]: The longer the work experience, the less likely staff were to experience difficulty in adhering to preventive measures (OR 1.21; 95% CI 1.13-1.29).

For peer review only - http://bmjopen.bmj.com/site/about/guidelines.xhtml

	Mitchell et al. (2012)[33]: Staff who had worked in health care for more than eleven years were significantly more compliant with wearing protective eyewear during aerosol-generating medical procedures than those who had worked for eleven years or less (69% v 54%, p<0.001), and also with wearing gowns (82% v 69%, p<0.001), gloves (92% v 85%, p=0.006) and N95 respirators (75% v 60%, p<0.001). For patient care not involving aerosol-generating medical procedures, staff with more than eleven years of experience were again significantly more likely to comply with wearing protective eyewear (38% v 31%, p=0.04), gowns (82% v 69%, p<0.001), N95 respirators (43% v 31%, p=0.003) and surgical masks (18% v 11%, p<0.016).
	Vinck et al. (2011)[36]: Compliance was higher in those who had been working in the public health service for one to ten years than those who had worked for eleven years or longer (p=0.034).
Training and knowledge	
Receiving training/education	Nour et al. (2017)[17]: After the training intervention in the study, there was a non-significant increase in preventive practices (p=0.168) – but compliance was good pre-intervention too.
	Taghrir et al. (2020)[21]: There was no significant association between preventive behaviours and having received education on COVID-19.
	Alsahafi & Cheng (2016)[28]: 22.8% of participants reported having received training about dealing with infectious disease outbreaks, 37.1% training in infection control policies and procedures, 54.4% training in hand hygiene and 45.6% training in N95 mask-wearing techniques. A high proportion of participants believed that insufficient training in infection control procedures contributed to poor compliance and the risk of infection (92.4% of nurses, 91% of physicians and 90.9% of other healthcare staff).

For peer review only - http://bmjopen.bmj.com/site/about/guidelines.xhtml

	May et al. (2010)[32]: There was a significant association between level of training and use of currently recommended CDC guidelines for patients with influenza-like illness, for fellows only (p=0.003); however, the number of fellows within the sample was small (n=5).
	Shigayeva et al. (2007)[35]: Recent infection control training was a significant predictor of consistent adherence to recommended behaviours (interactive training: OR 2.7, 95% CI, 1.7-4.4; passive training: OR 1.7, 95% CI, 1.0-3.0).
	Moore et al. (2005)[39]: Many participants reported having received poor prior training in infection control and therefore having to use new procedures and equipment without training or experience.
	Tan et al. (2006)[40]: Participants reported that little information on the disease or methods of control were available from previous education and training, which made them feel helpless.
	Corley et al. (2010)[41]: Participants believed that an infection control course with yearly refreshers would be useful and help improve compliance.
Sources of knowledge	Al-Amri et al. (2019)[12]: Those who attended CME activities had significantly higher levels of excellent practice than those who did not attend a CME activity (11.1% vs 1.5%, p=0.002). Participants who had textbooks as a source of knowledge had the highest percent of excellent practice (p<0.001).
	Kim & Choi (2016)[20]: Source of knowledge of MERS (television, or other) was not significantly associated with preventive behaviours.
	Taghrir et al. (2020)[21]: There was no significant association between preventive behaviours and source of information (WHO, CDC, or UpToDate v national guidelines v both v others).
Knowledge (of correct practices / outbreak)	Yap et al. (2010)[4]: There was a significant positive correlation between knowledge about H1N1/preventive behaviours, and practice of preventive behaviours (p<0.01).
	Al-Amri et al. (2019)[12]: Practice-related adherence to guidelines was not significantly associated with knowledge.

For peer review only - http://bmjopen.bmj.com/site/about/guidelines.xhtml

	De Perio et al. (2012)[14]: Knowledge of correct PPE was significantly associated with compliance at one hospital (p=0.047) but not the other three hospitals in the study.
	Nour et al. (2015)[16]: There was a significant positive association between knowledge and practice (p<0.001).
	Kim & Choi (2016)[20]: Having received MERS education was not significantly associated with preventive behaviours (p=0.209). However, in a correlational analysis, preventive behaviours were significantly correlated with MERS-related knowledge (p<0.01).
	Jeong et al. (2011)[22]: A score for 'cues to action' (consisting of consulting news or articles about the pandemic, being interested in methods for health promotion, and wishing to be educated on effective ways of preventing infection) was significantly associated with compliance (OR 2.63, CI 1.81-3.81).
	Hu et al. (2012)[27]: Self-reported adequate knowledge of self-protection and patient protection was significantly correlated with the perception of further improvement of PPE compliance (p<0.001).
	Alsahafi & Cheng (2016)[28]: A high proportion of participants believed that lack of knowledge about the mode of transmission contributed to poor compliance (94.2% of nurses, 90% of physicians and 90.2% of other healthcare staff).
	Hsu et al. (2011)[42]: 1.9% of participants believed lack of education about correct practices prevented hand hygiene compliance. 6.5% of participants believed improving undergraduate education efforts was the most important strategy for improving hand hygiene compliance. 7.3% of participants believed incorporating hand hygiene into examinations and the student internship marking scheme was the most important strategy.
Work-related factors	
Setting	De Perio et al. (2012)[14]: Staff who worked on an intensive care unit rotation during the study period were more likely to be in the 'high adherence' group (OR, 3.43; 95% CI, 1.02-11.56). Staff who worked on an inpatient ward rotation were less likely to be classified as high adherence (OR, 0.22, 95% CI, 0.06-0.80).

For peer review only

	Evirgen et al. (2014)[19]: Behaviour was not significantly associated with department at work or the department where the participant spends the most time.
	Taghrir et al. (2020)[21]: There was no significant association between preventive behaviours and ward worked on.
	Hu et al. (2012)[27]: Compliance rates significantly differed between institutions, from 0% to 88.1%, although all institutions were adult intensive care units.
	Chau et al. (2008)[30]: Those working in rehabilitation hospitals were significantly more likely to comply with protective behaviours overall, and use hand rub, than participants working in acute hospitals.
	Mitchell et al. (2012)[33]: Staff working in an intensive care unit or designated influenza ward were more compliant with wearing an N95 respirator for aerosol-generating medical procedures than those working in an emergency department (78% and 67% v 47%, p<0.001).
	Shigayeva et al. (2007)[35]: Working in a SARS unit was significantly associated with consistent adherence to recommended guidelines (OR 4.0, 95% CI, 1.8-8.9), as was working in an intensive care unit (OR 4.3, 95% CI, 2.0-9.0). Employment in a teaching hospital (OR 7.6, 95% CI, 4.8-11.9), SARS unit (OR 3.4, 95% CI, 1.3-9.4) or an intensive care unit (OR 1.9, 95% CI, 0.7-5.0) were associated with safer removal of PPE.
	Jaeger et al. (2011)[38]: 68% of staff in outpatient settings reported no PPE use with index patients, compared to 9% of staff in inpatient setting (p<0.01). Mask or N95 respirator use during index patient contact was more common in inpatient (41%) than outpatient settings (5%) (p<0.01). Glove use was more common among inpatient (57%) than outpatient (21%) settings. Always donning gloves, gown and either mask or N95 respirator was more common in inpatient (18%) than outpatient (5%) (p<0.01).
	Chia et al. (2005)[43]: Using administrative staff as the reference group, individuals working in surgical, medical and intensive care units were more likely to wear the appropriate respiratory protection. However, there was no significant

For peer review only - http://bmjopen.bmj.com/site/about/guidelines.xhtml

	difference for those individuals in the other areas (A&E, radiographic services, laboratory services, and others) compared to administrative personnel.
	Ki et al. (2019)[44]: Staff working in the emergency department were significantly more likely than staff in general wards to wear surgical masks (93.0% v 1.8%, p<0.001); however, there were no significant differences in hand-washing or wearing gloves.
	Wong et al. (2004)[45]: Staff working in high-infection districts were significantly more likely to wear gowns (63.0% v 42.0%, p<0.05), wash their hands before going home (82.6% v 63.0%, p<0.05) and use disinfectants to clean surfaces regularly (87.0% v 65.4%, p<0.01); however, they were less likely to quarantine themselves (6.5% v 27.5%, p<0.01) or grant leave to staff until confirmation of diagnosis (41.3% v 71.3%, p<0.01). Staff in public clinics were significantly more likely to wear gowns during consultations than staff in private clinics (81.3% v 45.3%, p<0.05); however, staff in private clinics were more likely to quarantine themselves for a 10-day period after SARS contact than their public sector colleagues (58.1% v 31.3%, p<0.05). Staff in the public sector were more likely to stay away from home.
Contact with confirmed cases	De Perio et al. (2012)[14]: Staff who were present at an aerosol-generating procedure on a patient with pH1N1 (OR 5.20, 95% CI, 1.54-17.55) were more likely to be in the high adherence group.
	Pratt et al. (2009)[18]: Nurses' experience of working directly with confirmed SARS cases was a significant predictor of compliance (p<0.001), with nurses who worked with SARS patients more likely to comply than those who did not. However, this was no longer significant after combining the impact of effort-reward imbalance and burnout.
	Evirgen et al. (2014)[19]: Behaviour was not significantly associated with awareness of suspected or confirmed H1N1 infection in their hospital or their department, or having contact with H1N1 cases.
	Shigayeva et al. (2007)[35]: Recognition of the patient as a SARS case was a significant predictor of consistent adherence to recommended barrier precautions (OR 2.5, 95% CI, 1.5-4.5).

For peer review only - http://bmjopen.bmj.com/site/about/guidelines.xhtml

	Chia et al. (2005)[43]: Staff who regularly came into contact with SARS patients were more likely to wear appropriate respiratory protection compared to those who did not (OR 0.673, 95% CI, 0.587-0.771).
	Wong et al. (2004)[45]: SARS-exposed staff were significantly more likely to insist on patients wearing masks (65.1% v 48.1%, p<0.05) and more likely to ensure their staff wore gloves and masks all the time (33.7% v 11.5%, p<0.01 for gloves, 100% v 92.3%, p<0.01 for masks). However, they were also less likely to quarantine themselves until confirmation of diagnosis (10.8% v 33.3%, p<0.01).
Workload	Pratt et al. (2009)[18]: Hours worked per week were reportedly significantly associated with compliance; however, the authors do not give details about whether more or less hours are associated with greater or lower compliance.
	Chor et al. (2012)[23]: There was a significant decrease in compliance with handwashing before patient contact with the increase in number of weekly patient contacts only in Hong Kong; no significant difference was noted in Singapore or the UK.
	Chau et al. (2008)[30]: Workload was a common barrier to compliance identified by participants.
	Shigayeva et al. (2007)[35]: Work on shifts requiring more frequent patient room entry was associated with lower compliance (OR for 6 or more entries per shift: 0.5, 95% CI, 0.32-0.86). The reported rate of consistent adherence was lower for staff members who reported entering a patient's room more than 5 times per shift: consistent adherence was reported for 84.1% of procedures involving staff members who reported entering the room 1-5 times, compared with 75.5% of procedures involving staff members who reported entering the room 6 or more times (p<0.001).
	Vinck et al. (2011)[36]: The compliance to measures to properly identify suspected cases of respondents who regularly worked overtime was reduced compared with those who did not (p=0.024). Conversely, working overtime was associated with increased compliance with informing patients that they were not supposed to leave their home while ill (p=0.048) and providing patients with the information leaflet (p=0.002). Additionally, compliance with control

For peer review only - http://bmjopen.bmj.com/site/about/guidelines.xhtml

	measures was not influenced by the number of inhabitants within the catchment area of the public health service or by the number of cases for which consultation within the centralised assessment system of each public health service with the Preparedness and Response Unit was carried out (objective workload).
	Moore et al. (2005)[39]: Fatigue was cited as a major cause of failing to follow the proper guidelines, with exhaustion leading to 'sloppy practice'.
	Corley et al. (2010)[41]: Isolation procedures and preventive behaviours created extra workload, frustration and confusion - educating and monitoring external staff and patient's relatives regarding PPE was an extra duty for already overstretched staff and was time-consuming and frustrating.
	Yassi et al. (2005)[46]: The increased time constraints and increased workload were felt to be important barriers to worker adherence to recommendations.
Monitoring of compliance	Hu et al. (2012)[27]: Significantly more nurses than physicians (92.1% v 86.4%, p=0.020) reported being reprimanded by their supervisor for non-compliance. Perceived reprimand by supervisor for non-compliance was a significant predictor of compliance (p=0.035).
	Hsu et al. (2011)[42]: 4.1% of participants believed better policing by infection control staff was the most important strategy for improving hand hygiene, and 3.3% thought that improving public awareness (patients monitoring doctors) was the most important.
	DiGiovanni et al. (2004)[47]: Public health authorities' monitoring of compliance played an important role in establishing the credibility of quarantine for 75% of physicians, 81% of nurses, and 85% of other healthcare workers: however, 58% of physicians, 37% of nurses and 40% of other staff rated the monitoring of their own compliance as bad.

For peer review only

For peer review only - http://bmjopen.bmj.com/site/about/guidelines.xhtml

Patient encounters	De Perio et al. (2012)[14]: 55% of those who reported not using the recommended PPE (n=40) stated it was because they did not know the patient had H1N1 or an influenza-like illness; 20% stated they did not think PPE was needed for the particular activity they were doing; 18% stated it was because the only entered the patients' room for a brief time; 13% stated it was because they did not touch the patient and 10% stated it was because they did not come within six feet of the patient.
	Shigayeva et al. (2007)[35]: Lower rates of consistent adherence were found among staff providing care for patients with higher Acute Physiology and Chronic Health Evaluation (APACHE) II scores – i.e. more severe illness (OR for score APACHE II of 20 or greater: 0.4, 95% CI, 0.28-0.68). Reported rates of consistent adherence were higher when healthcare workers were themselves performing procedures (adjusted OR: 1.7, 95% CI, 1.4-2.2) or assisting with them (adjusted OR: 1.5, 95% CI, 1.1-2.1), compared with procedures that they observed (81% v 79.1% v 74.2%, $p<0.001$).
Personal protective behaviour-related factors	
Availability of resources	De Perio et al. (2012)[14]: 18% of those who reported not using the recommended PPE (n=40) stated it was because their facility had run out of PPE, while 10% reported that their facility did not provide PPE.
	Hu et al. (2012)[27]: 63.2% reported that appropriate PPE was readily available. Availability of PPE in the ICU was significantly associated with compliance.
	Mitchell et al. (2012)[33]: Staff were significantly more compliant with PPE use when eyewear and gloves were readily available at the point of care; however, PPE availability did not increase compliance with the use of N95 respirators, surgical masks, or gowns.
	Moore et al. (2005)[39]: Participants reported supply problems with face shields and goggles.

For peer review only - http://bmjopen.bmj.com/site/about/guidelines.xhtml

	Tan et al. (2006)[40]: Many participants reported that they had not been properly mask-fitted due to lack of proper testing equipment in their clinic. Many were not provided with gowns. Several participants reported lack of goggles and visual shields in their clinics.
	Corley et al. (2010)[41]: Participants believed there were not enough PPE supplies and that guidelines changed depending on supplies running out – for example, surgical masks and plastic aprons became acceptable due to the lack of other PPE. One staff member reported wearing an incorrectly sized mask as their correct size was not in stock.
	Edeghere et al. (2015)[48]: Only 20% of participants experienced barriers in applying infection control measures; the main reported barrier was the occasional unavailability of recommended PPE, most often eye protection.
	Khalid et al. (2016)[49]: 60% perceived inadequate protective measures available to them and cited this as a stressor.
	Locatelli et al. (2012)[50]: Participants reported that stocks of N95 masks ran low so they started using surgical masks, which made it difficult to enforce the use of N95s.
	Rebmann & Wagner (2009)[51]: Many participants reported a lack of resources. Less than half of those whose facility had requested supplies from external agencies actually received them, whereas three participants whose facilities had not requested supplies did receive them. Many reported that supplies were back-ordered and they could not receive them in a timely manner. Theft of supplies was also an issue, with masks and respirators presumably being used or taken by visitors. 26.5% said their facility ran out of respiratory protection. Many reported staff in their facilities were wearing the wrong sizes as they were unable to get the correct sizes.
	Rowlands (2007)[52]: Participants reported that there were not enough masks for staff.
	Van Dijk et al. (2015)[53]: 73.5% believed they had sufficient personal protective materials; 26.5% believed they were insufficient.
	Hsu et al. (2006)[62]: 44% expressed dissatisfaction with shortage of protective equipment such as N95 masks and gloves.

For peer review only - http://bmjopen.bmj.com/site/about/guidelines.xhtml

Perceived difficulty of protective behaviour	Van Dijk et al. (2015)[53]: 35.0% believed recommended personal protective measures were feasible.
	Kang et al. (2018)[54]: Participants reported difficulties with the complexity of using several PPE items together.
Logistical issues	Rebmann & Wagner (2009)[51]: Participants reported logistical issues with needing to fit-test large numbers of staff in a short time.
	Kang et al. (2018)[54]: Limited hospital layouts disrupted space management for PPE use.
	Wong et al. (2012)[55]: Barriers to PPE use included inadequate shower facilities for staff, the small size of the changing room, and the physical layout of the two-bed isolation room which was poor for infectious case management.
Perceived effectiveness	Yap et al. (2010)[4]: There was a significant correlation between attitudes (an 'attitude' score was based on responses to questions on attitudes towards the use of PPE, vaccination, Tamiflu, help-seeking and risk perception) ($r=0.24$, $p<0.01$); there was no such correlation for cases or close contacts who answered the same questions.
	Hu et al. (2012)[27]: Positive attitude towards PPE use (i.e. belief that it provided adequate protection) significantly predicted compliance ($p=0.001$).
	Mitchell et al. (2012)[33]: Staff who felt better protected against influenza when they wore an N95 respirator were significantly more compliant with N95 respirator use than those who did not ($p<0.001$ for aerosol-generating medical procedures and $p=0.04$ for patient care not involving these procedures).
	Parker & Goldman (2006)[34]: Staff considering the combination of all infection control measures to be highly effective in protecting patients from SARS reported increased use of a mask when examining patients compared with staff considering all measures less effective ($p=0.05$). However, handwashing, glove use, gown use, eye protection use, and wearing a mask at all times in the emergency department did not significantly differ between the two groups.

For peer review only - http://bmjopen.bmj.com/site/about/guidelines.xhtml

	Yassi et al. (2005)[46]: Beliefs in the effectiveness of infection control guidelines, as modified by past experiences, were identified as having important influence on worker adherence to procedures.
	Kang et al. (2018)[54]: Participants expressed doubts about the quality and effectiveness of PPE.
Perceived importance	Vinck et al. (2011)[36]: Compliance with the recommendation to consult the Preparedness and Response Unit for centralised assessment of patients was fairly low, with the main reasons for non-compliance being finding it unnecessary (38.3%) or time-consuming (22.7%).
	Hsu et al. (2011)[42]: 20.1% believed that failure to recognise the importance of hand hygiene prevented compliance
Incentives	Jeong et al. (2011)[22]: Perceived benefits of the preventive behaviours significantly predicted higher compliance (OR 3.18, CI 2.15-4.68).
	Hsu et al. (2011)[42]: 19.9% of participants believed that providing positive incentives for hand hygiene was the single most important factor in improving compliance.
	Kang et al. (2018)[54]: Participants reported a lack of incentives to comply with preventive behaviours.
Convenience	De Perio et al. (2012)[14]: 30% of those who reported not using the recommended PPE (n=40) stated it was because the PPE was not available near their patients' room; 10% felt too busy to wear PPE; and 10% stated it was inconvenient to use.
	Hu et al. (2012)[27]: Half of participants reported that PPE use was inconvenient.
	Moore et al. (2005)[39]: Some participants reported that PPE was time-consuming due to having to constantly gown, de-gown, and re-gown.
	Tan et al. (2006)[40]: Participants reported that gowns were inconvenient during toilet breaks.

For peer review only - http://bmjopen.bmj.com/site/about/guidelines.xhtml

	Corley et al. (2010)[41]: Application and removal of PPE was reported to be time-consuming.
	Hsu et al. (2011)[42]: 60.4% believed lack of time prevented hand hygiene compliance.
	Kang et al. (2018)[54]: PPE use was inconvenient in the presence of hospital visitors.
	Lam & Hung (2013)[56]: Participants reported that gowning and de-gowning was very time-consuming.
	Nickell et al. (2004)[57]: 41.7% of participants felt precautionary measures affected their ability to do their job.
Comfort	Mitchell et al. (2012)[33]: Staff who reported always or often feeling comfortable wearing protective eyewear were significantly more likely to wear it (p<0.001). Similarly, staff who reported always or often feeling comfortable wearing an N95 respirator were significantly more compliant with N95 use. Less than 19% of staff reported always or often feeling short of breath, claustrophobic or dizzy when wearing protective eyewear or N95 respirators; no significant differences in compliance were observed between these staff and staff who reported never or rarely feeling short of breath, claustrophobic or dizzy.
	Moore et al. (2005)[39]: Some participants reported that PPE was uncomfortable.
	Tan et al. (2006)[40]: Participants reported physical discomfort from prolonged use of N95 masks, including difficulty breathing, headache, and development of allergic facial rash; they reported moments of complacency where they removed masks intermittently in order to allow normal breathing.
	Corley et al. (2010)[41]: Wearing PPE for extended periods was difficult as it was uncomfortable and could cause dehydration and injuries such as skin peeling.
	Yassi et al. (2005)[46]: Participants identified the need to address the increased amount of worker fatigue that existed when staff worked with full PPE, noting that the discomfort associated with wearing PPE was an important barriers to worker adherence to recommendations.

For peer review only - http://bmjopen.bmj.com/site/about/guidelines.xhtml

	Khalid et al. (2016)[49]: Participants reported that having to wear PPE was stressful.
	Rowlands (2007)[52]: Participants reported that masks were warm and uncomfortable, often leading them to develop headaches or skin rashes, and made it hard to breathe.
	Kang et al. (2018)[54]: Many participants reported having ill-fitted PPE.
	Lam & Hung (2013)[56]: Participants reported that PPE was uncomfortable.
	Nickell et al. (2004)[57]: 85.4% of participants found masks bothersome; of these, 92.9% stated they caused physical discomfort and 13.0% said they gave them a sense of isolation.
	Kang et al. (2018)[58]: Participants reported finding it stressful that their masks did not fit properly and emphasised the discomfort of PPE: they found it hard to breathe or see clearly while wearing it, which made their work harder, and it made them sweaty and dizzy when they wore it for too long.
	Khoo et al. (2005)[59]: Participant opinions on 3M powered air-purifying respirator: 2.3% found it uncomfortable, 11.6% reported moderate discomfort, 32.6% found it tolerable, 30.2% reported slight discomfort, and 23.3% found it very comfortable. 2.3% reported unacceptable visual impairment. 2.3% felt it was suffocating, 4.6% found breathing uncomfortable, 7.0% reported it was comfortable but moderately hard to breathe, 60.5% found it comfortable with minimal difficulty breathing, and 25.6% reported no breathing discomfort. Compared to Stryker powered air-purifying respirator: 4.5% found it uncomfortable, 4.5% reported moderate discomfort, 13.7% found it tolerable, 31.8% reported slight discomfort, and 45.5% found it very comfortable. 4.5% reported unacceptable visual impairment. 0% felt suffocated.
	Straus et al. (2004)[60]: Participants reported that masks hurt, made them sweat and gave them headaches.

For peer review only - http://bmjopen.bmj.com/site/about/guidelines.xhtml

Impact on patient care	Hu et al. (2012)[27]: 21.2% believed that PPE use would interfere with patient care (no significant difference between nurses and physicians). The perception that PPE use would interfere with patient care was significantly associated with poor compliance (p=0.002).
	Tan et al. (2006)[40]: Staff reported that masks muffled their speech and they had to raise their voices to communicate with patients. They also reported that patient reactions to PPE ranged from amusement to apprehension and anxiety; some saw their doctor wearing PPE and assumed they had been exposed to SARS and could infect them and their families.
	Corley et al. (2010)[41]: Wearing PPE was reported to make communication difficult.
	Rowlands (2007)[52]: Participants reported that masks impacted the patient counselling process as they compromised their communication skills and made their speech muffled. They also believed that psychiatric patients with paranoia were sensitive to masks.
	Lam & Hung (2013)[56]: Participants reported that PPE affected their communication with patients, making them have to speak very loudly in order to be heard.
	Nickell et al. (2004)[57]: 85.4% of participants found masks bothersome; of these, 47.0% stated that they caused difficulties communicating.
	Khoo et al. (2005)[59]: Some difficulties regarding communication. Opinions on the 3M powered air-purifying respirator: 0% felt they had to shout but 25.6% felt they had to raise their voice significantly, 37.2% had to raise voice moderately, 32.6% raised voice normally, and 4.6% could speak normally. 2.3% said it caused unacceptable hearing impairment, 11.6% significant, 27.9% moderate, 41.9% mild and 16.3% hearing impairment. Compared to the Stryker powered air-purifying respirator: 0% had to shout, 27.3% raised voice significantly, 31.8% raised voice moderately, 27.3% raised voice mildly, and 13.7% could speak normally. 0% reported unacceptable hearing impairment. About two-thirds of

For peer review only - http://bmjopen.bmj.com/site/about/guidelines.xhtml

	respondents agreed (22%) or strongly agreed (42%) that they looked frightening to their patients whenever they used the PAPR.
	Straus et al. (2004)[60]: Participants reported that wearing protective equipment made assessing and communicating with patients a challenge, as they could not establish any of the usual non-verbal cues with patients. They were concerned that patients may have been seen less frequently than usual because of the time required to get into protective clothing.
	Tolomiczenko et al. (2005)[61]: Nurses, more than other occupational groups, reported that infection control measures had an adverse impact on patient care because they were less 'visible' to their patients when gowned, masked and gloved.
Guidance	
Issues with availability or understanding	Alsahafi & Cheng (2016)[28]: Almost two thirds of participants were unaware of guidelines or protocols for the care of infected patients. A high proportion believed that compliance was negatively affected by the lack of policies and procedures (87.3% of nurses, 85% of doctors, 89.4% of other healthcare staff).
	Chau et al. (2008)[30]: Issues with communication and understanding were cited as barriers to compliance. Participants expressed mixed views on the guidelines; some described them as easy to follow and facilitating increased alertness, while others found them difficult to follow and too long (and suggested they be given summaries instead, to get a basic idea of the principles). Other suggestions included tailored information; more programmes in Chinese with a more interactive approach to learning; making courses more practical; and educating the public and patients too.
	Moore et al. (2005)[39]: Participants reported a lack of consistency with safety instructions and frequently changing directives. New levels of enforcement by regulatory agencies interfered with rational infection control practice and infection control policies developed elsewhere had little perceived relevance to their own workplace. Basic infection control policies and procedures were frequently perceived as not well-developed. A major problem was perceived to be

For peer review only - http://bmjopen.bmj.com/site/about/guidelines.xhtml

	issues with communicating the guidelines: participants reported it was difficult to disseminate information simultaneously to all staff; often new information was sent by email, which was not checked before going to work.
	Tan et al. (2006)[40]: Some participants reported not wearing gowns or gloves due to inadequate dissemination of information/instructions from health authorities. However, a SARS seminar and hotline were organised which were perceived to be an effective channel of communication to address concerns and queries on PPE.
	Corley et al. (2010)[41]: Participants perceived a lack of firm recommendations/guidelines regarding what specific PPE was required, which created confusion and made them feel unprotected. They also remarked that current guidelines changed too frequently to keep up. Participants also perceived a lack of clear guidelines and conflicting advice about what to do when a patient is deemed non-infectious.
	Yassi et al. (2005)[46]: To improve adherence to infection control guidelines, participants felt that better enforcement of infection control guidelines was needed but did not want such enforcement to rely on nurses "policing" other professionals.
	Locatelli et al. (2012)[50]: Participants reported many barriers to effective communication about infection control, including information overload, often contradictory information from different sources, and difficulty integrating information from various sources and working out what was a priority. 10 participants reported not having a pre-defined communication plan, or discovered flaws with their plan. Some reported that everyone had to develop their own guidelines, with no clear guidance or resources on how to do so provided. This resulted in confusion about the use of PPE.
	Rebmann & Wagner (2009)[51]: Participants reported a lack of guidance for alternate care sites and non-acute care settings such as physician offices, ambulatory care clinics, long-term care and nursing homes, as well as a general lack of infection prevention information and guidance related to occupational health issues. Rapidly-changing practice

For peer review only

	recommendations were often contradictory which made it difficult to maintain staff compliance and trust, and to stay abreast of the latest recommendations. There was a perceived lack of consistency between sources, with CDC, state health departments, and city health departments all saying different things. Participants reported gaps in communication between departments, or between hospitals and physician offices. Many did not know which type of mask or respirator to use when caring for H1N1 patients, and reported conflicting guidelines related to isolation, masks, and respirator use. 34.3% reported their facility had changed PPE use guidelines and 34.3% reported isolation guidelines had been changed midway through the H1N1 response.
	Van Dijk et al. (2015)[53]: 71.8% believed information provision was well-timed, 65.1% believed information provision was complete, 59.9% believed infection prevention policy changes were well-communicated, 59.2% believed information was explicitly formulated, and 68.5% felt they were clear on when to take protection measures.
	Kang et al. (2018)[54]: Participants described confusion from non-standardized and constantly evolving protocols, making it difficult to develop appropriate PPE protocols and training.
	Wong et al. (2012)[55]: Participants reported difficulties with guidelines as they changed so often.
	Lam & Hung (2013)[56]: Half the participants were satisfied that departmental communication about guidelines was effective, but others had problems with excessive guidelines and rapidly-changing regulations.
	Kang et al. (2018)[58]: Participants cited frequently changing guidelines as a stressor.
	Tolomiczenko et al. (2005)[61]: Emergency room personnel and intensive care unit personnel, more than other groups, reported poor communication about SARS and false ideas about safety precautions, adding that they were not kept up-to-date about what to wear and that personnel at the hospital entrance had no idea what the rules of the day were regarding what should be worn and when.

For peer review only

	Hsu et al. (2006)[62]: 37% participants reported poor communication between agencies and 30% reported a lack of standard operating procedures.
	Nhan et al. (2012)[63]: Participants reported that communication of guidelines was a barrier to compliance: guidelines were inconsistent between different pandemic management levels and advisory committees, often lacked clarity, were from an overwhelming number of different sources, frequently changed, and were slow to be communicated. Guidelines were also perceived as too rigid to accommodate particular regional or local situations.
	Rambaldini et al. (2005)[64]: Participants reported variability in the information made available at different hospitals, the perceived inconsistency of interhospital and interdepartmental interpretation of public health directives, and the lack of communication between institutions. Some reported frustration at receiving information filtered down from other sources, rather than first-hand. Residents at other hospitals, however, reported receiving regular updates from program directors, chief medical residents and infection control specialists in a variety of forms including websites, email and direct communication via personal conversations or group meetings. The most important information was deemed to be changes to infection control protocols, the status of SARS at their hospital and within the community, and the clinical condition of affected health care colleagues.
	Robertson et al. (2004)[65]: Participants reported a lack of clear guidelines about how to minimise infection at home and in quarantine and uncertainty about effective risk control, leading them to fear contaminating their family members. Unclear guidelines meant that staff developed their own thoughts about which behaviours were necessary and which were not.
Distress and risk perception	

For peer review only - http://bmjopen.bmj.com/site/about/guidelines.xhtml

Distress	Chia et al. (2005)[43]: Staff with IES scores of 20 or greater were more likely to wear more effective respiratory protection than those with scores of 19 or less.
	Wong et al. (2004)[45]: Staff who were highly anxious/frightened of dealing with SARS were significantly more likely to insist on patients wearing masks (68.1% v 48.5%, $p<0.05$), to ensure their staff wore gloves at work (32.4% v 17.9%, $p<0.05$), and to wash their hands before entering their home (79.4% v 61.2%, $p<0.05$).
	DiGiovanni et al. (2004)[47]: 5% reported such high stress they were tempted to break quarantine.
Risk perception	Kim & Choi (2016)[20]: Risk perception was significantly positively correlated with preventive behaviours ($p<0.01$).
	Taghrir et al. (2020)[21]: There was a significant negative correlation between preventive behaviours and fear (fear of becoming infected and thinking they are more likely to become infected than others) – as preventive behaviours increased, risk perception declined.
	Jeong et al. (2011)[22]: Perceived susceptibility to the pandemic significantly predicted preventive behaviours.
	Chor et al. (2012)[23]: In Hong Kong, compliance with infection control practices (wearing gloves, changing gloves and washing hands before each patient contact) was associated with the perceived seriousness of the likely impact of pandemic H1N1 on their life; however, compliance with these practices did not correlate with the perceived disease severity among staff in Singapore or the UK.
	Parker & Goldman (2006)[34]: Considering SARS to be a high, rather than low, public health threat was significantly associated with increased compliance with hand-washing, wearing a mask at all times in the emergency department, and wearing gloves when examining patients ($p<0.05$).
	Moore et al. (2005)[39]: Some participants reported that nobody ‘cut corners’ as fear of infection was very real.

For peer review only - http://bmjopen.bmj.com/site/about/guidelines.xhtml

	DiGiovanni et al. (2004)[47]: Staff who directly cared for SARS patients feared becoming infected and passing along infection to their families, so they not only complied with quarantine but also restricted social contact more strictly and for longer than required, with many sending their families away or living alone in their basements; 94% reported the most important reason for complying was to reduce the risk of transmission to others.
	Rebmann & Wagner (2009)[51]: Participants believed that healthcare workers' perceptions of H1N1 severity led to poor compliance with PPE; for example, one participant reported that staff chose not to wear PPE with the idea that they wanted to get mild flu so they wouldn't get a more virulent form of the disease in the autumn.
	Goulia et al. (2010)[66]: Degree of worry about H1N1 was significantly associated with restricting social contacts outside of work because the work environment was considered dangerous ($p<0.0005$).
Attitudes and behaviours of others	
Non-compliance of others	Hu et al. (2012)[27]: 21% reported their colleagues often forgot to use PPE during patient care.
	Chau et al. (2008)[30]: A common barrier to compliance identified by participants was poor preventive practices by colleagues, patients and visitors.
	Moore et al. (2005)[39]: Basic infection control policies and procedures were frequently perceived as not being enforced; participants identified various deficiencies including tracking who received training, lack of consistent policies for quarantining people, reuse of masks, and lack of consistency in deciding which patients require negative pressure rooms.
	Hsu et al. (2011)[42]: 56.1% of participants believed that senior clinicians leading by example was the most important strategy for improving hand hygiene compliance, and 10.1% believed that peer example (i.e. senior staff not practicing

For peer review only - http://bmjopen.bmj.com/site/about/guidelines.xhtml

	hand hygiene) was a reason for poor compliance. 2.4% of participants believed support and emphasis of guidelines by top administrative staff was the most important strategy for improving hand hygiene compliance.
	Yassi et al. (2005)[46]: The peer environment, especially the compliance of other occupational groups (including physicians), and the feedback from peers were identified as factors that could exert positive or negative influence on individual worker actions.
	DiGiovanni et al. (2004)[47]: As hospitals began to experience critical staff shortages, many modified the recommended quarantine guidelines to allow their staff to return.
	Locatelli et al. (2012)[50]: Participants reported resistance from administration staff about putting up signs with precautions, because they thought it might cause panic.
Attitudes of family members	Moore et al. (2005)[39]: Participants reported that a facilitator of compliance was the attitude of family members, who wanted them to comply with recommended measures as they were scared of getting sick and were often angry or upset that their healthcare worker family member was continuing to work.
	Yassi et al. (2005)[46]: Attitudes of family members, in particular the fear that family members expressed toward contracting SARS, influenced workers' behaviour.
Other potential predictors of compliance	
Complacency	Tan et al. (2006)[40]: Participants reported not wearing gowns due to complacency.
Commitment	Alsahafi & Cheng (2016)[28]: 96.2% of nurses, 93.6% of physicians and 98.4% of other healthcare staff believed that healthcare workers' lack of commitment to policies and procedures reduced compliance.

For peer review only - http://bmjopen.bmj.com/site/about/guidelines.xhtml

	Moore et al. (2005)[39]: Participants reported a lack of commitment to occupational health and safety by workers and management.
Previous experience with pandemic	Moore et al. (2005)[39]: Some participants reported that past exposures to disease led to decreased compliance when experience demonstrated that protective behaviours are not needed 100% of the time.
Passing of time	Rebmann & Wagner (2009)[51]: Participants reported that compliance lowered with time, as it was hard to keep vigilant in the middle of outbreak.
Forgetting	Hu et al. (2012)[27]: Approximately 21% reported they forgot to change PPE between patients. Forgetting to use or change PPE was significantly associated with poor compliance ($p<0.001$).
Death of a doctor	Tan et al. (2006)[40]: Compliance improved after the death of a doctor friend.
Effort-reward imbalance	Pratt et al. (2009)[18]: Effort-reward imbalance ratio was significantly predictive of decreased compliance ($p<0.05$).
Consulting with experts	Manabe et al. (2012)[15]: Consulting with experts with experience of caring for H5N1 patients did not significantly predict compliance with recommended behaviours ($p=0.776$).
Lack of staff	Moore et al. (2005)[39]: Participants felt there were not enough infection control practitioners, or they were not visible enough.
	Corley et al. (2010)[41]: Participants reported that not having enough staff (or enough experienced staff working at once) was a barrier to compliance.
PPE fit-testing procedures	Moore et al. (2005)[39]: Participants reported the fit-testing process for PPE was not standardised and some felt it was unnecessary.
Professionalism	Yassi et al. (2005)[46]: Professionalism was identified as having an important influence on adherence to procedures.

For peer review only - http://bmjopen.bmj.com/site/about/guidelines.xhtml

Use of public transport	Evirgen et al. (2014)[19]: Behaviour was not significantly associated with using public transportation to get to work.
Loss of income	DiGiovanni et al. (2004)[47]: Fear of loss of income was an obstacle to complying with quarantine, particularly among those unconvinced quarantine was necessary – 60% of doctors, 76% of nurses and 70% of other healthcare workers said they would need fairly detailed information about when, how, and how much compensation they would receive as encouragement to comply with quarantine.
Overcrowding	Alsahafi & Cheng (2016)[28]: Overcrowding in the emergency department was cited as a reason for poor compliance (97.2% of nurses, 96.6% of physicians, 93.2% of other healthcare staff)

For peer review only

For peer review only - http://bmjopen.bmj.com/site/about/guidelines.xhtml

Appendix I. Search strategy

Search 1: Compliance-related terms

compliance OR comply OR adhere* OR attitude* OR behaviour* OR behavior*

Search 2: Infection control-related terms

hygien* OR handwashing OR hand-washing OR infection control OR mask* OR PPE OR protective equipment OR social distanc*

Search 3: Healthcare worker-related terms

healthcare worker* OR healthcare staff OR healthcare personnel OR "medical personnel" OR "hospital personnel" OR frontline OR medical staff OR medical worker* OR doctor* OR dentist* OR midwife* OR midwives OR surgeon* OR nurse* OR gp* OR general practitioner* OR physician* OR health care worker* OR health care staff OR hospital employee* OR medical employee* OR healthcare employee* OR health care employee* OR hospital worker* OR hospital staff

Search 4: Emerging infectious disease-related terms

SARS OR severe acute respiratory syndrome OR MERS OR middle east respiratory syndrome OR H1N1 OR swine flu OR H5N1 OR avian influenza OR bird flu OR pandemic* OR coronavirus OR COVID-19 OR n-COV OR SARS-COV-2

Searches 1, 2, 3 and 4 were combined with AND.

For peer review only

For peer review only - http://bmjopen.bmj.com/site/about/guidelines.xhtml

Appendix II. Flow diagram of screening process

PRISMA 2009 Flow Diagram

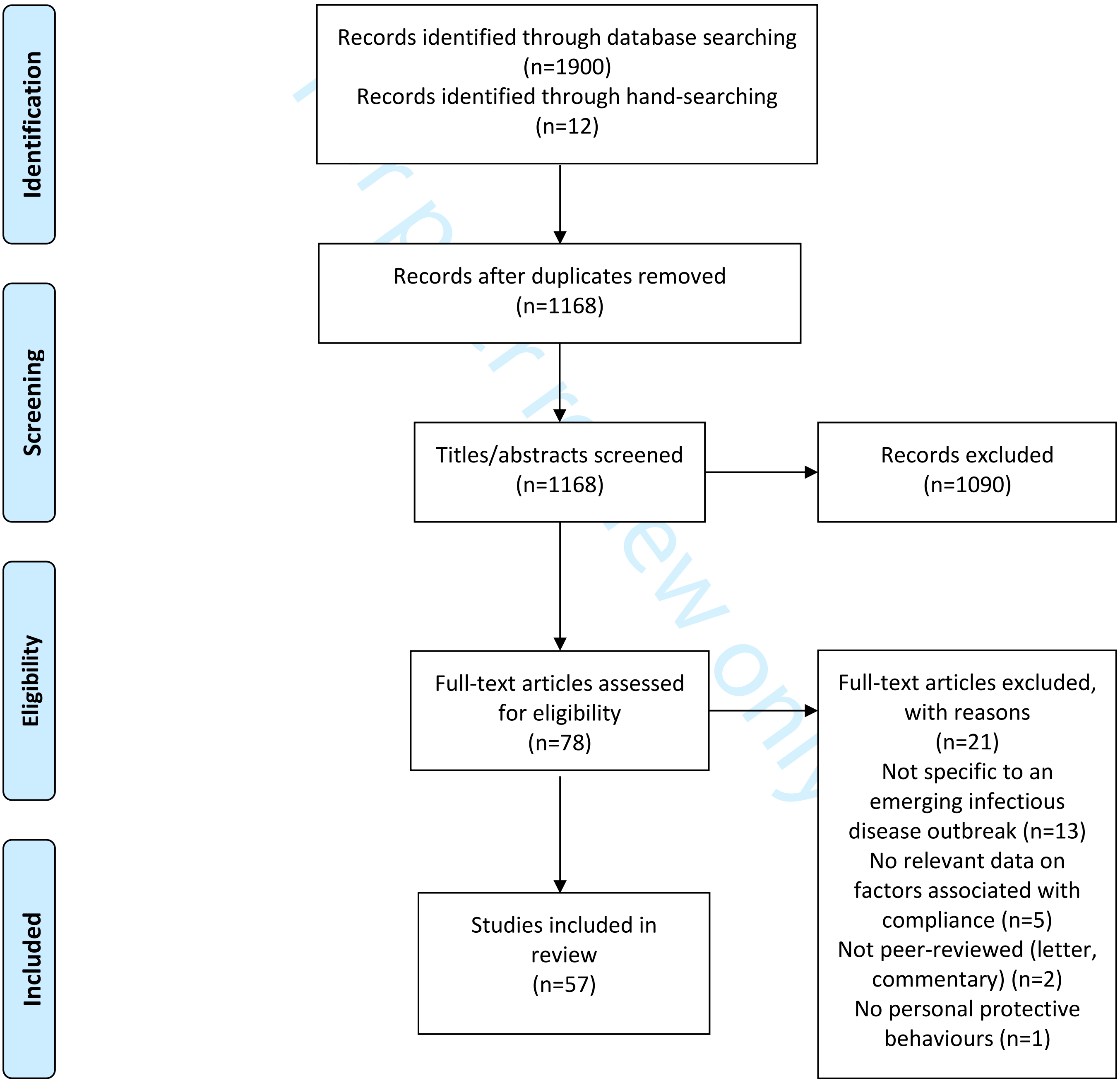

For peer review only - http://bmjopen.bmj.com/site/about/guidelines.xhtml

PRISMA 2009 Checklist

Section/topic	#	Checklist item	Reported on page #
**TITLE**			
Title	1	Identify the report as a systematic review, meta-analysis, or both.	1
**ABSTRACT**			
Structured summary	2	Provide a structured summary including, as applicable: background; objectives; data sources; study eligibility criteria, participants, and interventions; study appraisal and synthesis methods; results; limitations; conclusions and implications of key findings; systematic review registration number.	2
**INTRODUCTION**			
Rationale	3	Describe the rationale for the review in the context of what is already known.	3
Objectives	4	Provide an explicit statement of questions being addressed with reference to participants, interventions, comparisons, outcomes, and study design (PICOS).	3
**METHODS**			
Protocol and registration	5	Indicate if a review protocol exists, if and where it can be accessed (e.g., Web address), and, if available, provide registration information including registration number.	n/a
Eligibility criteria	6	Specify study characteristics (e.g., PICOS, length of follow-up) and report characteristics (e.g., years considered, language, publication status) used as criteria for eligibility, giving rationale.	4
Information sources	7	Describe all information sources (e.g., databases with dates of coverage, contact with study authors to identify additional studies) in the search and date last searched.	4
Search	8	Present full electronic search strategy for at least one database, including any limits used, such that it could be repeated.	Supplementary material
Study selection	9	State the process for selecting studies (i.e., screening, eligibility, included in systematic review, and, if applicable, included in the meta-analysis).	4
Data collection process	10	Describe method of data extraction from reports (e.g., piloted forms, independently, in duplicate) and any processes for obtaining and confirming data from investigators.	4
Data items	11	List and define all variables for which data were sought (e.g., PICOS, funding sources) and any assumptions and simplifications made.	4
Risk of bias in individual studies	12	Describe methods used for assessing risk of bias of individual studies (including specification of whether this was done at the study or outcome level), and how this information is to be used in any data synthesis.	n/a – rapid review so not all steps of a systematic review were

For peer review only - http://bmjopen.bmj.com/site/about/guidelines.xhtml

PRISMA 2009 Checklist

			followed, as recommended by WHO (2017) guidelines
Summary measures	13	State the principal summary measures (e.g., risk ratio, difference in means).	n/a
Synthesis of results	14	Describe the methods of handling data and combining results of studies, if done, including measures of consistency (e.g., I^2) for each meta-analysis.	4

Section/topic	#	Checklist item	Reported on page #
Risk of bias across studies	15	Specify any assessment of risk of bias that may affect the cumulative evidence (e.g., publication bias, selective reporting within studies).	n/a
Additional analyses	16	Describe methods of additional analyses (e.g., sensitivity or subgroup analyses, meta-regression), if done, indicating which were pre-specified.	n/a
**RESULTS**			
Study selection	17	Give numbers of studies screened, assessed for eligibility, and included in the review, with reasons for exclusions at each stage, ideally with a flow diagram.	5 / supplementary material
Study characteristics	18	For each study, present characteristics for which data were extracted (e.g., study size, PICOS, follow-up period) and provide the citations.	Supplementary material
Risk of bias within studies	19	Present data on risk of bias of each study and, if available, any outcome level assessment (see item 12).	n/a
Results of individual studies	20	For all outcomes considered (benefits or harms), present, for each study: (a) simple summary data for each intervention group (b) effect estimates and confidence intervals, ideally with a forest plot.	n/a
Synthesis of results	21	Present results of each meta-analysis done, including confidence intervals and measures of consistency.	n/a
Risk of bias across studies	22	Present results of any assessment of risk of bias across studies (see Item 15).	n/a
Additional analysis	23	Give results of additional analyses, if done (e.g., sensitivity or subgroup analyses, meta-regression [see Item 16]).	n/a
**DISCUSSION**			
Summary of evidence	24	Summarize the main findings including the strength of evidence for each main outcome; consider their relevance to key groups (e.g., healthcare providers, users, and policy makers).	11-12

For peer review only

PRISMA 2009 Checklist

Limitations	25	Discuss limitations at study and outcome level (e.g., risk of bias), and at review-level (e.g., incomplete retrieval of identified research, reporting bias).	13-14
Conclusions	26	Provide a general interpretation of the results in the context of other evidence, and implications for future research.	14
**FUNDING**			
Funding	27	Describe sources of funding for the systematic review and other support (e.g., supply of data); role of funders for the systematic review.	Title page (submitted as supplementary material for editor)

From: Moher D, Liberati A, Tetzlaff J, Altman DG, The PRISMA Group (2009). Preferred Reporting Items for Systematic Reviews and Meta-Analyses: The PRISMA Statement. PLoS Med 6(7): e1000097. doi:10.1371/journal.pmed1000097

For more information, visit: **www.prisma-statement.org**.

For peer review only

For peer review only - http://bmjopen.bmj.com/site/about/guidelines.xhtml

BMJ Open

Factors affecting healthcare workers' compliance with social and behavioural infection control measures during emerging infectious disease outbreaks: Rapid evidence review.

Journal:	*BMJ Open*
Manuscript ID	bmjopen-2021-049857.R1
Article Type:	Original research
Date Submitted by the Author:	03-Jun-2021

Complete List of Authors:	BROOKS, SAMANTHA; King's College London, Department of Psychological Medicine<br>Greenberg , N; King's College London, Department of Psychological Medicine<br>Wessely, Simon; King's College London, Department of Psychological Medicine<br>Rubin, GJ; King's College London, Department of Psychological Medicine

**Primary Subject Heading**:	Infectious diseases
Secondary Subject Heading:	Occupational and environmental medicine
Keywords:	COVID-19, Public health < INFECTIOUS DISEASES, Infection control < INFECTIOUS DISEASES

SCHOLARONE™
Manuscripts

I, the Submitting Author has the right to grant and does grant on behalf of all authors of the Work (as defined in the below author licence), an exclusive licence and/or a non-exclusive licence for contributions from authors who are: i) UK Crown employees; ii) where BMJ has agreed a CC-BY licence shall apply, and/or iii) in accordance with the terms applicable for US Federal Government officers or employees acting as part of their official duties; on a worldwide, perpetual, irrevocable, royalty-free basis to BMJ Publishing Group Ltd ("BMJ") its licensees and where the relevant Journal is co-owned by BMJ to the co-owners of the Journal, to publish the Work in this journal and any other BMJ products and to exploit all rights, as set out in our licence.

The Submitting Author accepts and understands that any supply made under these terms is made by BMJ to the Submitting Author unless you are acting as an employee on behalf of your employer or a postgraduate student of an affiliated institution which is paying any applicable article publishing charge ("APC") for Open Access articles. Where the Submitting Author wishes to make the Work available on an Open Access basis (and intends to pay the relevant APC), the terms of reuse of such Open Access shall be governed by a Creative Commons licence – details of these licences and which Creative Commons licence will apply to this Work are set out in our licence referred to above.

Other than as permitted in any relevant BMJ Author's Self Archiving Policies, I confirm this Work has not been accepted for publication elsewhere, is not being considered for publication elsewhere and does not duplicate material already published. I confirm all authors consent to publication of this Work and authorise the granting of this licence.

For peer review only

Factors affecting healthcare workers' compliance with social and behavioural infection control measures during emerging infectious disease outbreaks: Rapid evidence review.

Dr Samantha K. Brooks PhD[1], Professor Neil Greenberg FRCPsych[1], Professor Sir Simon Wessely FMedSci[1], Dr G. James Rubin PhD[1]

[1]King's College London, Department of Psychological Medicine, Cutcombe Road, London SE5 9RJ, United Kingdom

Corresponding author: Dr S.K. Brooks, King's College London, Department of Psychological Medicine, Cutcombe Road, London SE5 9RJ, United Kingdom. Email: samantha.k.brooks@kcl.ac.uk Telephone: 020 7848 0432

Keywords Compliance, COVID-19, healthcare workers, infection control, public health

Word count 4,281

For peer review only

Abstract

Objective The 2019-2020 outbreak of novel coronavirus has raised concerns about nosocomial transmission. This review's aim was to explore the existing literature on emerging infectious disease outbreaks to identify factors associated with compliance with infection control measures among healthcare staff.

Methods A rapid evidence review for primary studies relevant to healthcare workers' compliance with infection control measures.

Results Fifty-six papers were reviewed. Staff working in emergency or intensive care settings or with contact with confirmed cases appeared more likely to comply with recommendations. There was some evidence anxiety and concern about the risk of infection were more associated with compliance, and that monitoring from superiors could improve compliance. Observed non-compliance of colleagues could hinder compliance. Staff identified many barriers to compliance related to personal protective equipment, including availability; perceived difficulty and effectiveness; inconvenience; discomfort; and a negative impact on patient care. There were many issues regarding the communication and ease of understanding of infection control guidance.

Conclusion We recommend provision of training and education tailored for different occupational roles within the healthcare setting; managerial staff 'leading by example'; ensuring adequate resources for infection control; and timely provision of practical evidence-based infection control guidelines.

For peer review only

Strengths and limitations of this study

- This review synthesises existing literature on healthcare workers' compliance with infection control measures during infectious disease outbreaks, providing an overview of what has already been established and identifying gaps in the literature.
- The search strategy was broad, and reference lists of included papers were hand-searched for any additional relevant papers which may have been missed.
- The search was limited to English-language papers due to the rapid nature of the review.
- Included papers did not undergo formal quality appraisal, again due to the rapid nature of the review.

Introduction

Recent years have seen frequent outbreaks of emerging infectious diseases.[1] Examples include severe acute respiratory syndrome (SARS) in 2003, avian influenza (H5N1) in 2004, swine flu (H1N1) in 2009, Middle East respiratory syndrome (MERS) in 2015 and most recently coronavirus SARS-CoV-2 originating in 2019. Previous outbreaks saw high levels of nosocomial transmission (hospital-acquired infections).[2]

A major cause of nosocomial transmission is poor compliance with personal protective behaviours among healthcare staff.[3 4] Early reports from the COVID-19 frontline have noted poor compliance of healthcare workers with recommended behaviours: in one hospital in China, many had their masks hung from one ear or pulled downwards, while more than half had inadequate hand hygiene.[5]

Compliance with infection control behaviours can be difficult. Previous literature has reported on difficulties in the general population with adhering to protective behaviours such as facemask-wearing,[6] using hand sanitising gel[7] and quarantine[8] during infectious disease outbreaks. The main facilitators of compliance in the general population appear to be perceived susceptibility, perceived severity of being afflicted and perceived benefits of

For peer review only - http://bmjopen.bmj.com/site/about/guidelines.xhtml

compliance as well as accurate knowledge about the disease and the recommended behaviours, while major barriers include discomfort, embarrassment and practical issues.[6 8] A review[9] of healthcare workers' perceptions of barriers and facilitators to compliance with guidelines during respiratory outbreaks suggested that protective practices are influenced by understanding of guidelines, support received from managers, communication about guidelines, sufficient resources, perceived value of following guidance, comfort of personal protective equipment (PPE), perceived impact of PPE on patients, and workplace culture. However, this review focused only on qualitative literature, the majority of which related to tuberculosis.

We systematically reviewed existing literature on compliance with social and behavioural protective behaviours among staff involved in healthcare, specifically during outbreaks of emerging infectious diseases and encompassing quantitative and qualitative research.

For peer review only

For peer review only - http://bmjopen.bmj.com/site/about/guidelines.xhtml

Methods

This rapid evidence review was carried out according to World Health Organization guidelines:[10] the basic principles of a systematic literature review were followed, with certain aspects simplified in order to produce evidence rapidly at a time when urgent evidence synthesis is required. Searching of grey literature and quality appraisal of included studies were not carried out.

Search strategy

The search strategy consisted of four search strings (adherence terms; protective behaviour terms; emerging infectious disease terms; and healthcare worker terms). The full search strategy can be seen in Appendix I. Five databases were searched from date of inception to May 4th, 2020: MEDLINE®, PsycINFO®, Embase, Global Health, and Web of Science.

Selection criteria

To be included, studies had to: i) contain primary data; ii) be published in peer-reviewed journals in English; iii) include participants who worked in healthcare; iv) include data on factors predicting adherence to social or behavioural infection control practices during emerging infectious disease pandemics. For the latter criterion, quantitative data needed to report statistics on factors associated with compliance, while the qualitative component of the review considered studies reporting on participants' beliefs about facilitators and barriers to compliance as well as any reported difficulties in complying with protective behaviours.

Screening

One author (SKB) ran the searches on all databases on May 4th, 2020. Resulting citations were downloaded to EndNote© version X9 (Thomson Reuters, New York, USA). The same author evaluated titles for relevance, then used the inclusion criteria to screen abstracts and then full texts of remaining citations and exclude any which were irrelevant. Any queries or uncertainties about inclusion were discussed with the wider research team. Reference lists of all remaining papers were hand-searched for additional relevant studies.

Data extraction and synthesis

SKB extracted the following data from papers: authors, publication year, country of study, design, participants (including *n*, demographic information and profession), disease outbreak,

For peer review only - http://bmjopen.bmj.com/site/about/guidelines.xhtml

protective behaviours measured, measures used, and key results. We used thematic analysis[11] to synthesise the data and group results into themes.

Patient and public involvement

No patients were involved in this review study.

Results

The initial search strategy yielded 1900 papers, of which 744 duplicates were removed and 1090 were excluded based on title or abstract. An additional twelve papers were found via hand-searching the reference lists of included papers. After full-text screening, 56 papers remained for inclusion. A PRISMA flow diagram of the process can be seen in Appendix II.

Countries represented in the literature included Canada (n=13), Saudi Arabia (n=7), Singapore (n=7), China (n=5), South Korea (n=5), USA (n=4), Netherlands (n=2), Australia (n=1), Greece (n=1), India (n=1), Iran (n=1), Taiwan (n=1), Turkey (n=1), UK (n=1) and Vietnam (n=1). A further five papers included participants from multiple countries. Papers discussed H1N1 (n=21), SARS (n=20), MERS (n=11), avian influenza (n=2) and COVID-19 (n=2). The participants represented in the literature were from a wide range of roles and departments in the healthcare profession, and a wide range of protective behaviours were considered. A full overview of study characteristics is presented in Supplementary Table I.

Eight main themes were identified. These were socio-demographics and personal characteristics; occupational role; training and knowledge; work-related factors; personal protective behaviour-related factors; guidance; distress and risk perception; and attitudes and behaviours of others. Supplementary Table II provides a summary of themes and sub-themes and identifies for each theme which papers showed a significant association with protective behaviours, which papers found no significant association, and which papers supplemented these findings by reporting on the theme but without statistical analysis (e.g. qualitative papers and papers with descriptive statistics only). Supplementary Table III summarises the evidence extracted from the literature for each theme. A number of other potential predictors of compliance were considered but only appeared in one paper each; these are presented in Supplementary Tables I and III but not covered in the text.

For peer review only - http://bmjopen.bmj.com/site/about/guidelines.xhtml

Socio-demographics and personal characteristics

Overall, there appeared to be no significant association between age[12-18] or gender[12-14 17 19-21] and protective behaviour. Only one study[20] found that older age was significantly associated with protective behaviours, while two found that female staff were significantly more likely to comply with protective behaviours.[16 22]

One study examined nationality as a predictor[12] and found that Saudi staff were significantly more likely to comply with protective behaviours than non-Saudi staff working in the same city. Mixed findings were reported in the studies comparing behaviours across countries. Staff in Hong Kong and Singapore were more likely to comply than UK staff[23] whereas Koh et al.[24] and Wong et al.[25] found that staff in Singapore vs Indonesia or Hong Kong vs Canada respectively were more likely to comply with some recommendations but not others (see Supplementary Table III for details). A worldwide study[26] found no significant differences between countries in terms of taking protective measures.

There was no evidence of association between compliance and religion[20] or marital status.[18 19] One paper found that staff in 'high or middle' socioeconomic status were more likely to comply than those of lower socioeconomic status.[22] Four studies found that level of education was not associated with compliance[13 18 20 21] while one found a significant association between compliance and qualification[12] (see Table III for details). One study found that H1N1 influenza vaccination was significantly associated with high compliance.[27] Having a chronic illness, being pregnant, or having a pregnant spouse, elderly person or school-aged child at home were not associated with compliance, but staff with babies at home were more likely to comply with protective behaviours.[19]

Occupational role

Many studies which examined role as a predictor of compliance found a significant relationship;[12 19 23 28-36] however, due to the variety of different roles compared across studies, it was not possible to identify an exact pattern (see Supplementary Table III for details). Five studies found no significant association between role and compliance.[13 16 17 37 38]

Length of time in role was not significantly associated with compliance in five studies[12 16-18 30] while two studies suggested that longer experience of working in healthcare was associated

For peer review only - http://bmjopen.bmj.com/site/about/guidelines.xhtml

For peer review only

with greater compliance[31 33] and one suggested that less than ten years' experience was associated with significantly higher compliance than more experience.[36]

Training and knowledge

There was mixed evidence on the effectiveness of outbreak-specific training and education: Taghrir et al.[21] found no significant association between protective behaviours and having received education; Nour et al.[17] found a non-significant increase in protective practices post-training; and Shigayeva et al.[35] found that recent infection control training was a significant predictor of compliance with recommended behaviours. Jeong et al.[22] found that staff who sought information about the outbreak and infection control were more likely to comply with recommended behaviours. Qualitative evidence suggested that staff felt their prior training and education were not useful in dealing with the rapidly-changing nature of emerging infectious disease outbreaks.[39 40] Participants themselves believed that inadequate training was a barrier to compliance[28] and that infection control training with annual refresher courses would benefit them.[41]

Sources of knowledge about the outbreak and protective behaviours were not associated with protective behaviours in two studies[20 21] while knowledge from textbooks and attending Continuing Medical Education activities were significantly associated with higher levels of protective practice in one study.[12] Receiving outbreak-specific training was not significantly associated with compliance, but higher outbreak-related knowledge did result in significantly higher compliance.[20] Another study examining knowledge of the outbreak itself[28] found that the majority of participants believed lack of knowledge about mode of transmission contributed to poor compliance. Knowledge of current recommendations was associated with compliance in three studies;[4 16 27] associated with compliance in one hospital but not three other hospitals in another study;[14] and was not associated with compliance in one further study.[12] Hsu et al.[42] found that a minority of participants believed that lack of education explained lack of compliance.

Work-related factors

Compliance appeared to be higher in higher acuity settings such as emergency, intensive care or inpatient departments.[14 35 38 43 44] Wong et al.[45] found that staff in high-infection districts were more likely to wear gowns, wash hands and use disinfectants but less likely to comply

For peer review only - http://bmjopen.bmj.com/site/about/guidelines.xhtml

with quarantine measures. Two studies found no significant association between setting and compliance.[19 21]

Having contact with confirmed cases was associated with higher compliance in three studies[14 35 43] whereas one study[19] found no association. A further study[18] found higher compliance in staff who worked directly with confirmed cases. Wong et al.[45] found that SARS-exposed staff were more likely than non-SARS-exposed staff to comply with mask guidance, but less likely to quarantine themselves.

There was some evidence that high workload may be a barrier to compliance with recommended personal protective behaviours,[23 30 35 36 39 41 46] although one study also suggested that higher workload (in terms of working overtime) was associated with increased compliance in terms of giving patients appropriate infection control advice.[36]

Two studies suggested that monitoring of compliance by superiors or public health authorities encouraged compliance,[27 47] while a small minority of participants in Hsu et al.'s[42] study reported that better policing by infection control staff was the most important strategy for improving compliance.

Two studies suggested compliance was associated with characteristics of the patient encounter. Many of De Perio et al.'s[14] participants reported not using recommended PPE if they did not know the patient had H1N1 or an influenza-like illness, if they did not think it was needed for the particular activity they were doing, if they only entered the patient's room for a brief time, if they did not touch the patient or if they did not come within six feet of the patient. Meanwhile, Shigayeva et al.'s[35] participants were less likely to comply with recommended behaviours when providing care for patients with more severe illness (which the authors suggest may be due to the time required to don barrier equipment leading staff to put patient safety above self-protection) and if they were only observing procedures, rather than performing or assisting with them.

Personal protective behaviour-related factors

Many studies, although mostly without statistical analysis, reported issues in performing personal protective behaviours, most notably due to lack of availability of appropriate PPE,

For peer review only - http://bmjopen.bmj.com/site/about/guidelines.xhtml

perceived difficulty of protective behaviours, logistic issues, perceived effectiveness, perceived importance, convenience, comfort, and the impact on patient care.

Participants in several studies raised concerns about lack of PPE,[14 39-42 48-53] mostly due to insufficient resources. Shortage of PPE created difficulties such as having to wear the wrong size PPE.[40 41 51] One study showed that availability of PPE was significantly associated with higher compliance[27] while another found that staff were significantly more compliant with PPE use when eyewear and gloves were readily available at the point of care, but availability did not increase compliance of N95 respirators, surgical masks or gowns.[33] Logistic issues were also reported, such as lack of space in the hospital, making it difficult to use PPE appropriately.[51 54 55]

There may also be issues regarding the perceived difficulty of protective behaviours. Kang et al.'s[54] participants reported difficulties with the complexity of using several PPE items together, and only 35% of Van Dijk et al.'s[53] participants thought the recommended measures were feasible.

Perceived effectiveness of PPE significantly predicted higher compliance in three studies.[27 33 34] In one qualitative study, staff expressed doubts about the quality and effectiveness of PPE.[54] Beliefs in the effectiveness of infection control procedures, as modified by previous experiences, were identified by participants as having a positive impact on compliance.[46]

Hsu et al.[42] reported that failure to recognise the importance of hand hygiene prevented compliance, while Vinck et al.[36] found that the main reason for not complying with the recommendation to consult with a specialist unit for centralised assessment of symptomatic patients was associated with finding it 'unnecessary'.

Participants in many qualitative studies reported not using PPE due to perceived inconvenience and its effect on their ability to do their job[14 27 39-42 54 56 57] – in particular, it appeared that participants found it too time-consuming to change in and out of protective clothing as it slowed them down substantially.

Many of Khalid et al.'s[49] participants reported that having to use PPE was 'stressful'. Many qualitative studies found that discomfort of PPE was reported to be a barrier to compliance.[39-

For peer review only

[41 46 52 56-58] In several studies, participants reported physical harm due to PPE, including dehydration and skin peeling,[41] difficulty breathing,[40 52 58 59] sweating and dizziness,[58] headaches[40 52 60] and skin rashes.[40 52] Comfort of PPE was examined as a potential predictor of compliance in one quantitative study[33] which found that staff who reported always or often feeling comfortable wearing protective eyewear and N95 respirators were significantly more likely to wear them; however, no significant association was found in compliance between staff who reported always or often feeling short of breath, claustrophobic or dizzy when wearing protective eyewear or N95 respirators and those who rarely experienced these symptoms.

Qualitative data also revealed that many healthcare professionals believe that PPE use has an impact on patient care, making it difficult to communicate with patients due to muffled speech,[40 41 52 56 57 59] being unable to establish non-verbal cues with patients[60] and making them less 'visible' to their patients.[61] Participants in Rowlands' study[52] also reported concern that masks could be frightening for psychiatric patients, and Tan et al.'s[40] participants reported that PPE created anxiety in some patients who assumed they were wearing it as they had been exposed to the virus. One quantitative study found that the perception that PPE use would interfere with patient care was significantly associated poor compliance.[27]

Guidance

Participants in many studies reported a lack of guidance on the protocols for caring for infected patients and protecting themselves.[28 30 39 40 41 46 50 51 53-56 58 61-65]

In particular, guidelines were often found to be too long, providing staff with 'information overload'.[30 50 63] The recommended protocols were also reported to change too frequently for staff to keep up[39 41 54-56 58 61 63 64] and staff received conflicting messages from different sources.[39 41 50 51 62-64] Other issues included inconsistent advice about what to do when patients are deemed non-infectious,[41] difficulty working out what should be prioritised[50] and external guidance with little relevance to specific locations.[39] The manner of communication of guidance could also be an issue, with Moore et al.'s[39] participants reporting that new guidelines were sent to them by email, which they did not check before work; Rambaldini et al.'s[64] participants reporting frustration that information was filtered down from other sources rather than given to them first-hand; and Nhan et al.'s[63] participants suggesting that communication about changes to protocols was too slow.

For peer review only - http://bmjopen.bmj.com/site/about/guidelines.xhtml

Distress and risk perception

High levels of distress were associated with higher compliance in two studies: Chia et al.[43] found that higher IES scores were associated with higher use of respiratory protection while Wong et al.[45] found that staff who were highly anxious were more likely to comply with recommended protective behaviours. However, when the protective measure involved quarantine, some staff felt such high stress that they were tempted to break guidelines[47] (it is unclear from the paper what percentage of these actually did break the guidelines, if any).

Risk perception appeared to be associated with compliance. Four studies found that compliance was significantly more likely in staff who perceived the disease to be a serious risk[20 22 34 66] and another found that perceived seriousness of the outbreak was significantly associated with compliance in Hong Kong but not in Singapore or the UK.[23] Three qualitative papers also suggested that participants themselves believed staff complied with recommended behaviours when they perceived the risk to be severe.[39 47 51] However, one study[21] showed a significant negative correlation between protective behaviours and fear of infection.

Attitudes and behaviours of others

Qualitative data revealed that many participants had observed non-compliance in colleagues or managers[27 39] which could lead to non-compliance in the participants themselves.[30,42] DiGiovanni et al.'s[47] participants suggested that managers had modified the recommended quarantine guidelines to allow their staff to return in response to critical staff shortages. Yassi et al.'s[46] participants believed that compliance of other occupational groups within the healthcare setting had an impact on compliance, while more than half of Hsu et al.'s[42] participants believed that senior staff 'leading by example' and complying with recommended behaviours was the single most important strategy in improving staff compliance.

Attitudes of family members were also deemed important in two qualitative studies[39 46] with healthcare staff reporting they were encouraged to comply by anxious family members who were afraid of getting infected.

Discussion

For peer review only - http://bmjopen.bmj.com/site/about/guidelines.xhtml

Overall, the studies reviewed provided mixed and sometimes contradictory results. Nonetheless, some risk factors which stood out as being the most promising either for identifying specific groups at-risk for poor compliance or for targeting in interventions. These included: working outside of emergency or intensive care settings; not working with confirmed infection cases; lack of concern about risk of infection; lack of monitoring by superiors; observed non-compliance of colleagues; lack of PPE; perceived difficulty using PPE; perceived lack of effectiveness or lack of importance of PPE; perceived inconvenience and discomfort of PPE; perceived negative impact of PPE on patient care; lack of infection control guidance; and inconsistent or unclear guidance. Organisations faced with nosocomial transmission would be wise, as part of their mitigation efforts, to look to these areas in an effort to help staff adhere to protective behaviours.

There was little evidence for an association between compliance with protective behaviours and socio-demographic or personal characteristics. There was some evidence that compliance levels differ across countries: it is not clear whether this is due to between-country differences in the communication of guidance, different risk perceptions due to different countries' media coverage of the outbreak, different levels of training received, or cultural differences in the participants themselves.

This review suggests that compliance may differ between different roles and different settings. Targeted interventions for specific occupational groups with different levels of patient contact, hierarchies and cultures, may be helpful. Whilst it was unclear from the varied data which occupational groups might need more attention, it seems that those not in emergency departments or intensive care units and not working directly with infected patients could benefit from additional focus.

We found little evidence that training and education significantly improved compliance. This initially seems surprising as previous trials of training courses to improve knowledge of infection control practices and encourage compliance with them have suggested positive results.[67-69] However, taken together with the evidence that compliance differs between occupational groups, it may be that staff in different roles require different levels and methods of training. We also found mixed evidence of the impact of knowledge about infection control on compliance with the recommended measures: this may be associated with what is actually covered in training. For example, as it appears that the perceived

For peer review only - http://bmjopen.bmj.com/site/about/guidelines.xhtml

importance of PPE is related to compliance, it may be that training that covers the 'why' rather than the 'how' of PPE is particularly useful. However, training itself should not be viewed as a panacea. Many of the factors identified by our review, and discussed below, are not amenable to better training.

A small amount of evidence suggested that observed non-compliance by others – including colleagues - could affect healthcare workers' own levels of compliance. This is likely to be particularly problematic where managers and supervisors fail to 'lead by example' and ensure that they comply with the recommended policies and procedures. 'Role modelling' by superiors could be useful, with supervisors setting the standards for infection control practices and reinforcing them.

The availability, ease of use, perceived effectiveness, convenience, comfort, and impact on patients of PPE may be other key factors which need to be addressed. This review suggests fairly negative views of PPE, with participants reporting lack of appropriate resources, the inconvenience of having to change in and out of PPE repeatedly, physical discomfort, and a negative impact on their ability to communicate with patients. It is useful to know about these concerns about PPE as this can be a target to change.

Reviewed studies suggest difficulties understanding and keeping up with rapidly changing recommendations. This may be unavoidable during an outbreak of an emerging infectious disease, as new evidence becomes available. Staff should therefore be prepared for the likelihood of changes, both to help maintain trust and to ensure that they monitor for updates.

There was some evidence that higher levels of anxiety and risk perception were associated with higher compliance. This may suggest that, whilst it is important not to create unhealthy anxiety, desensitisation to risk or genuine reductions in risk may lead to a reduction in PPE use. Indeed, it has been reported that staff in industrial settings experience complacency in terms of protective behaviours, as a result of habituation (repeated exposures which do not result in anything bad, lessening fearful responding) and low levels of positive reinforcement for safe behaviour.[70] Particular care may be needed to maintain PPE use as risk changes, or is perceived to change, over the course of an outbreak.

Limitations

For peer review only - http://bmjopen.bmj.com/site/about/guidelines.xhtml

We are aware that many papers (particularly on SARS and MERS) have been published in other (predominantly Asian) languages, but due to the rapid nature of this review we limited inclusion to English-language papers only. Future reviews should consider translating and analysing the many relevant foreign-language papers. Also due to the rapid nature of the review, quality appraisal of individual papers was not carried out as this is not always deemed necessary when urgent evidence synthesis is required.[71] Our searches were carried out in May 2020 in order to provide an early look at the factors associated with nosocomial transmission at the start of the COVID-19 pandemic, and we are aware that new relevant studies have been published since this date. It would be useful for future reviews to consider the newer literature on COVID-19 and compare the results to our own.

Conclusion

Interventions that may be helpful for improving healthcare workers' compliance include role-specific or setting-specific training; emphasising the importance of protective behaviours and the risk of infection if behaviours are not performed; monitoring of staff behaviours by supervisors and positive reinforcement for correct behaviours; managerial staff leading by example; training focused on the importance and effectiveness of PPE; and better communication of guidelines. The results of this review also suggest that the following interventions would likely *not* be useful: training aimed at specific age or gender groups; training focused on increasing knowledge about the outbreak itself; or training on how to use PPE without also emphasising *why* it is necessary.

Contributorship statement

GJR conceived the work. SKB conducted the literature search and screened search results for inclusion, and GJR, NG and SW resolved any queries about inclusion. All authors contributed to the qualitative results synthesis, prepared the first draft of the manuscript and approved the final manuscript.

Competing interests

None declared.

Funding

The research was funded by the National Institute for Health Research Health Protection Research Unit (NIHR HPRU) in Emergency Preparedness and Response at King's College

For peer review only - http://bmjopen.bmj.com/site/about/guidelines.xhtml

London in partnership with Public Health England (PHE), in collaboration with the University of East Anglia (funding reference: NIHR200890). The views expressed are those of the author(s) and not necessarily those of the NHS, the NIHR, the Department of Health and Social Care, or Public Health England. The funder had no involvement in the study design, collection, analysis or interpretation of data, writing the report, or decision to submit for publication.

Data sharing statement

No original data were generated for this study.

Ethics

Not needed - no original data generated / no participants.

References

1. World Health Organization, Regional Office for South-East Asia. (2014). A brief guide to emerging infectious diseases and zoonoses. WHO Regional Office for South-East Asia. https://apps.who.int/iris/handle/10665/204722 [accessed 09 May 2020]

2. Feng D, de Vlas SJ, Fang L-Q, Han XN, Zhao W-J, Sheng S, et al. The SARS epidemic in mainland China: bringing together all epidemiological data. Trop Med Int Health. 2009;14(Suppl 1):4-13.

3. Gamage B, Moore D, Copes R, Yassi A, Bryce E, members of The BC Interdisciplinary Respiratory Protection Study Group Vancouver, British Columbia, Canada. Protecting health care workers from SARS and other respiratory pathogens: A review of the infection control literature. Am J Infect Control. 2005;33(2),114-21.

4. Yap J, Lee VJ, Yau TY, Ng TP, Tor PC. Knowledge, attitudes and practices towards pandemic influenza among cases, close contacts, and healthcare workers in tropical Singapore: a cross-sectional survey. BMC Public Health. 2010;10:442.

For peer review only - http://bmjopen.bmj.com/site/about/guidelines.xhtml

5. Xu C, Jin J, Song J, Yang Y, Yao M, Zhang Y, et al. Application of refined management in prevention and control of the coronavirus disease 2019 epidemic in non-isolated areas of a general hospital. Int J Nurs Sci. 2020;7(2):143-7.

6. Sim SW, Moey KSP, Tan NC. The use of facemasks to prevent respiratory infection: a literature review in the context of the Health Belief Model. Singapore Med J. 2014;55(3):160-7.

7. Rubin GJ, Potts HWW, Michie S. The impact of communications about swine flu (influenza A H1N1v) on public responses to the outbreak: results from 36 national telephone surveys in the UK. Health Technol Assess. 2010;14(34):183-266.

8. Webster RK, Brooks SK, Smith LE, Woodland L, Wessely S, Rubin GJ. How to improve adherence with quarantine: rapid review of the evidence. Public Health. 2020;182:163-9.

9. Houghton C, Meskell P, Delaney H, Smalle M, Glenton C, Booth A, et al. Barriers and facilitators to healthcare workers' adherence with infection prevention and control (IPC) guidelines for respiratory infectious diseases: a rapid qualitative evidence synthesis. Cochrane Database of Systematic Reviews. 2020;4.

10. World Health Organization. (2017). Rapid reviews to strengthen health policy and systems: a practical guide. https://www.who.int/alliance- hpsr/resources/publications/rapid-review-guide/en/ [accessed 04 May 2020]

11. Braun V, Clarke V. Using thematic analysis in psychology. Qual Res Psychol. 2006;3(2):77-101.

12. Al-Amri S, Bharti R, Alsaleem SA, Al-Musa HM, Chaudhary S, Al-Shaikh AA. Knowledge and practices of primary health care physicians regarding updated guidelines of MERS-CoV infection in Abha city. J Family Med Prim Care. 2019;8(2):455-61.

13. Datta SS, Kuppuraman D, Boratne AV, Abraham SB, Singh Z. Knowledge, attitude and practices regarding swine flu among para-medical workers in a tertiary care hospital in Pondicherry. J Commun Dis. 2011;43(1):1-9.

For peer review only

14. de Perio MA, Brueck SE, Mueller CA, Milne CK, Rubin MA, Gundlapalli AV, et al. Evaluation of 2009 pandemic influenza A (H1N1) exposures and illness among physicians in training. Am J Infect Control. 2012;40(7):617-21.

15. Manabe T, Pham Thi Phuong T, Kudo K, Vu Thi Tuong V, Takasaki J, Nguyen Dang T, et al. Impact of education and network for Avian Influenza H5N1 in humans: Knowledge, clinical practice, and motivation on medical providers in Vietnam. Plos One. 2012;7(1).

16. Nour MO, Babalghith AO, Natto HA, Al-Amin FO, Alawneh SM. Knowledge, attitude and practices of healthcare providers towards MERS-CoV infection at Makkah hospitals, KSA. Int Res J Med Med Sci. 2015;3(4): 103-12.

17. Nour MO, Babalghith AO, Natto HA, Alawneh SM, Elamin FO. Raising awareness of health care providers about MERS-CoV infection in public hospitals in Mecca, Saudi Arabia. East Mediterr Health J. 2017;23(8):534-42.

18. Pratt M, Kerr M, Wong C. The impact of ERI, burnout, and caring for SARS patients on hospital nurses' self-reported compliance with infection control. CJIC. 2009;24(3):167-74.

19. Evirgen O, Savas N, Koksaldi Motor V, Onlen Y, Yengil E. An evaluation of knowledge, attitudes, and behaviors of employees of a university hospital in an H1N1 influenza pandemic. J Infect Dev Ctries. 2014;8(5):561-9.

20. Kim JS, Choi JS. Middle East respiratory syndrome-related knowledge, preventive behaviours and risk perception among nursing students during outbreak. J Clin Nurs. 2016;25(17-18):2542-9.

21. Taghrir MH, Borazjani R, Shiraly R. COVID-19 and Iranian medical students; A survey on their related-knowledge, preventive behaviors and risk perception. Arch Iran Med. 2020;23(4):249-54.

22. Jeong HS, Lee DW, Youn CH, Lee MK, Lee SJ, Suh YS, et al. Perception and performance of preventive behaviors for the pandemic influenza in hospital employees and outpatients. Yonsei Med J. 2011;52(1):181-7.

For peer review only

23. Chor JSY, Pada SK, Stephenson I, Goggins WB, Tambyah PA, Medina M, et al. Differences in the compliance with hospital infection control practices during the 2009 influenza H1N1 pandemic in three countries. J Hosp Infect. 2012;81(2):98-103.

24. Koh GC-H, Abikusno N, Kwing CS, Yee WT, Kusumaratna R, Sundram M, et al. Avian influenza and South Jakarta primary healthcare workers: a controlled mixed-method study. Trop Med Int Health. 2009;14(7):817-29.

25. Wong SYS, Wong W, Jaakkimainen L, Bondy S, Tsang KK, Lee A. Primary care physicians in Hong Kong and Canada - how did their practices differ during the SARS epidemic? Fam Pract. 2005;22(4):361-6.

26. Kamate SK, Sharma S, Thakar S, Srivastava D, Sengupta K, Hadi AJ, et al. Assessing knowledge, attitudes and practices of dental practitioners regarding the covid-19 pandemic: A multinational study. Dent Med Probl. 2020;57(1):11-7.

27. Hu X, Zhang Z, Li N, Liu D, He W, Zhang W, et al. Self-reported use of Personal Protective Equipment among Chinese Critical Care Clinicians during 2009 H1N1 influenza pandemic. PLoS ONE. 2012;7(9).

28. Alsahafi AJ, Cheng AC. Knowledge, attitudes and behaviours of healthcare workers in the Kingdom of Saudi Arabia to MERS Coronavirus and other emerging infectious diseases. Int J Environ Res Public Health. 2016;13(12).

29. Alsubaie S, Hani Temsah M, Al-Eyadhy AA, Gossady I, Hasan GM, Al-Rabiaah A, et al. Middle East Respiratory Syndrome Coronavirus epidemic impact on healthcare workers' risk perceptions, work and personal lives. J Infect Dev Ctries. 2019;13(10):920-6.

30. Chau JP, Thompson DR, Twinn S, Lee DT, Lopez V, Ho LS. An evaluation of SARS and droplet infection control practices in acute and rehabilitation hospitals in Hong Kong. Hong Kong Med J. 2008;14 Suppl 4:44-7.

For peer review only

For peer review only - http://bmjopen.bmj.com/site/about/guidelines.xhtml

31. Koh D, Lim MK, Chia SE, Ko SM, Qian F, Ng V, et al. Risk perception and impact of severe acute respiratory syndrome (SARS) on work and personal lives of healthcare workers in Singapore: What can we learn? Med Care. 2005;43(7):676-82.

32. May L, Katz R, Johnston L, Sanza M, Petinaux B. Assessing physicians' in training attitudes and behaviors during the 2009 H1N1 influenza season: a cross-sectional survey of medical students and residents in an urban academic setting. Influenza Other Respir Viruses. 2010;4(5):267-75.

33. Mitchell R, Ogunremi T, Astrakianakis G, Bryce E, Gervais R, Gravel D, et al. Impact of the 2009 influenza A (H1N1) pandemic on Canadian health care workers: A survey on vaccination, illness, absenteeism, and personal protective equipment. Am J Infect Control. 2012;40(7):611-6.

34. Parker MJ, Goldman RD. Paediatric emergency department staff perceptions of infection control measures against severe acute respiratory syndrome. EMJ. 2006;23(5):349-53.

35. Shigayeva A, Green K, Raboud JM, Henry B, Simor AE, Vearncombe M, et al. Factors associated with critical-care healthcare workers' adherence to recommended barrier precautions during the Toronto severe acute respiratory syndrome outbreak. Infect Control Hosp Epidemiol. 2007;28(11):1275-83.

36. Vinck L, Isken L, Hooiveld M, Trompenaars M, Ijzermans J, Timen A. Impact of the 2009 influenza A(H1N1) pandemic on public health workers in the Netherlands. Euro Surveill. 2011;16(7).

37. Alshammari M, Reynolds KA, Verhougstraete M, O'Rourke MK. Comparison of perceived and observed hand hygiene compliance in healthcare workers in MERS-CoV endemic regions. Healthcare (Basel). 2018;6(4).

38. Jaeger JL, Patel M, Dharan N, Hancock K, Meites E, Mattson C, et al. Transmission of 2009 Pandemic Influenza A (H1N1) Virus among healthcare personnel-Southern California, 2009. Infect Control Hosp Epidemiol. 2011;32(12):1149-57.

For peer review only

39. Moore DM, Gilbert M, Saunders S, Bryce E, Yassi A. Occupational health and infection control practices related to severe acute respiratory syndrome: health care worker perceptions. AAOHN J. 2005;53(6):257-66.

40. Tan NC, Goh LG, Lee SS. Family physicians' experiences, behaviour, and use of personal protection equipment during the SARS outbreak in Singapore: Do they fit the Becker Health Belief Model? Asia Pac J Public Health. 2006;18(3):49-56.

41. Corley A, Hammond NE, Fraser JF. The experiences of health care workers employed in an Australian intensive care unit during the H1N1 influenza pandemic of 2009: A phenomenological study. Int J Nurs Stud. 2010;47(5):577-85.

42. Hsu LY, Jin J, Ang BS, Kurup A, Tambyah PA. Hand hygiene and infection control survey pre- and peri-H1N1-2009 pandemic: knowledge and perceptions of final year medical students in Singapore. Singapore Med J. 2011;52(7):486-90.

43. Chia SE, Koh D, Fones C, Qian F, Ng V, Tan BH, et al. Appropriate use of personal protective equipment among healthcare workers in public sector hospitals and primary healthcare polyclinics during the SARS outbreak in Singapore. Occup Environ Med. 2005;62(7):473-7.

44. Ki HK, Han SK, Son JS, Park SO. Risk of transmission via medical employees and importance of routine infection-prevention policy in a nosocomial outbreak of Middle East respiratory syndrome (MERS): a descriptive analysis from a tertiary care hospital in South Korea. BMC Pulm Med. 2019;19(1).

45. Wong WCW, Lee A, Tsang KK, Wong SYS. How did general practitioners protect themselves, their family, and staff during the SARS epidemic in Hong Kong? J Epidemiol Community Health. 2004;58(3):180-5.

46. Yassi A, Moore D, Fitzgerald JM, Bigelow P, Hon CY, Bryce E, et al. Research gaps in protecting healthcare workers from SARS and other respiratory pathogens: an interdisciplinary, multi-stakeholder, evidence-based approach. J Occup Environ Med. 2005;47(1):41-50.

For peer review only

47. DiGiovanni C, Conley J, Chiu D, Zaborski J. Factors influencing compliance with quarantine in Toronto during the 2003 SARS outbreak. Biosecur Bioterror. 2004;2(4):265-72.

48. Edeghere O, Fowler T, Wilson F, Caspa R, Raichand S, Kara E, et al. Knowledge, attitudes, experience and behaviour of frontline health care workers during the early phase of 2009 influenza A(H1N1) pandemic, Birmingham, UK. J Health Serv Res Pol. 2015;20(1):26-30.

49. Khalid I, Khalid TJ, Qabajah MR, Barnard AG, Qushmaq IA. Healthcare workers' emotions, perceived stressors and coping strategies during a MERS-CoV outbreak. Clin Med Res. 2016;14(1):7-14.

50. Locatelli SM, LaVela SL, Hogan TP, Kerr AN, Weaver FM. Communication and information sharing at VA facilities during the 2009 novel H1N1 influenza pandemic. Am J Infect Control. 2012;40(7):622-6.

51. Rebmann T, Wagner W. Infection preventionists' experience during the first months of the 2009 novel H1N1 influenza A pandemic. Am J Infect Control. 2009;37(10):e5-16.

52. Rowlands A. (2007). Medical social work practice and SARS in Singapore. Soc Work Health Care. 2007;45(3):57-83.

53. van Dijk CE, Hooiveld M, Jentink A, Isken LD, Timen A, Yzermans CJ. Experiences of General Practitioners and Practice Assistants during the Influenza A (H1N1) Pandemic in the Netherlands: A Cross-Sectional Survey. Plos One. 2015;10(8).

54. Kang J, Kim EJ, Choi JH, Hong HK, Han S-H, Choi IS, et al. Difficulties in using personal protective equipment: Training experiences with the 2015 outbreak of Middle East respiratory syndrome in Korea. Am J Infect Control. 2018;46(2):235-7.

55. Wong ELY, Wong SYS, Lee N, Cheung A, Griffiths, S. Healthcare workers' duty concerns of working in the isolation ward during the novel H1N1 pandemic. J Clin Nurs. 2012;21(9-10):1466-75.

For peer review only

56. Lam KK, Hung SYM. Perceptions of emergency nurses during the human swine influenza outbreak: A qualitative study. Int Emerg Nurs. 2013;21(4):240-6.

57. Nickell LA, Crighton EJ, Tracy CS, Al-Enazy H, Bolaji Y, Hanjrah S, et al. Psychosocial effects of SARS on hospital staff: survey of a large tertiary care institution. CMAJ. 2004;170(5):793-8.

58. Kang HS, Son YD, Chae S-M, Corte C. Working experiences of nurses during the Middle East respiratory syndrome outbreak. Int J Nurs Pract. 2018;24(5):e12664.

59. Khoo KL, Leng PH, Ibrahim IB, Lim TK. The changing face of healthcare worker perceptions on powered air-purifying respirators during the SARS outbreak. Respirology. 2005;10(1):107-10.

60. Straus SE, Wilson K, Rambaldini G, Rath D, Lin Y, Gold WL, et al. Severe acute respiratory syndrome and its impact on professionalism: qualitative study of physicians' behaviour during an emerging healthcare crisis. BMJ. 2004;329(7457):83.

61. Tolomiczenko GS, Kahan M, Ricci M, Strathern L, Jeney C, Patterson K, et al. SARS: Coping with the impact at a community hospital. J Adv Nurs. 2005;50(1):101-10.

62. Hsu CC, Chen T, Chang M, Chang YK. Confidence in controlling a SARS outbreak: Experiences of public health nurses in managing home quarantine measures in Taiwan. Am J Infect Control. 2006;34(4):176-81.

63. Nhan C, Laprise R, Douville-Fradet M, Macdonald ME, Quach C. Coordination and resource-related difficulties encountered by Quebec's public health specialists and infectious diseases/medical microbiologists in the management of A(H1N1) – a mixed-method, exploratory survey. BMC Public Health. 2012;12:115.

64. Rambaldini G, Wilson K, Rath D, Lin Y, Gold WL, Kapral MK, et al. The impact of severe acute respiratory syndrome on medical house staff: A qualitative study. J Gen Intern Med. 2005;20(5):381-5.

For peer review only

65. Robertson E, Hershenfield K, Grace SL, Stewart DE. The psychosocial effects of being quarantined following exposure to SARS: A qualitative study of Toronto health care workers. Can J Psychiatry. 2004;49(6):403-7.

66. Goulia P, Mantas C, Dimitroula D, Mantis D, Hyphantis T. General hospital staff worries, perceived sufficiency of information and associated psychological distress during the A/H1N1 influenza pandemic. BMC Infect Dis. 2010;10 (no pagination).

67. Carrico RM, Coty MB, Goss LK, LaJoie AS. Changing health care worker behavior in relation to respiratory disease transmission with a novel training approach that uses biosimulation. Am J Infect Control. 2007;35(1):14-9.

68. Hon C-Y, Gamage B, Bryce EA, LoChang J, Yassi A, Maultsaid D, et al. Personal protective equipment in health care: Can online infection control courses transfer knowledge and improve proper selection and use? Am J Infect Control. 2008;36(10):E33-7.

69. May L, Lung D, Harter K. An intervention to improve compliance with transmission precautions for influenza in the emergency department: Successes and challenges. J Emerg Med. 2012;42(1):79-85.

70. Hyten C, Ludwig TD. Complacency in process safety: A behaviour analysis toward prevention strategies. J Organ Behav Manage. 2017;37(3-4):240-60.

71. Haby MM, Chapman E, Clark R, Barreto J, Reveiz L, Lavis JN. What are the best methodologies for rapid reviews of the research evidence for evidence-informed decision making in health policy and practice: a rapid review. Health Res Policy Syst. 2016;14(1):83.

For peer review only

For peer review only

For peer review only - http://bmjopen.bmj.com/site/about/guidelines.xhtml

Supplementary Table I. Study characteristics

Authors (year)	Country	Disease outbreak	Participants: N, age, gender	Professional role	Protective behaviours examined
Yap et al. (2010)[4]	Singapore	H1N1	331;<br>Mean age 23.2, range 18-61;<br>7.6% female	Healthcare workers	Questions on preventive practices related to mask wearing, vaccination, avoidance behaviours, personal habits (e.g. washing hands regularly, covering mouth when coughing/sneezing), and medical attention seeking practice
Al-Amri et al. (2019)[12]	Saudi Arabia	MERS	85;<br>37.6% under 30; 49.4% aged 30-40; 12.9% aged over 40;<br>38.8% female	General practitioners (62.4%), specialists (27.1%), consultants (10.6%)	'Adherence toward guidelines' assessed through 10 statements; no further information provided
Datta et al. (2011)[13]	India	H1N1	237;<br>58.23% aged 21-25, 24.05% aged 26-30;<br>20.25% female	Tertiary care hospital staff: 113 nurses, 41 laboratory technicians, 20 auxilliary nursing midwives, 61 'other' including medical social	Frequent handwashing, use of face mask, use of gloves, closed-front gown, use of disinfectant, 'others'

For peer review only

				workers and physiotherapists	
De Perio et al. (2012)[14]	USA	H1N1	88;<br>Median age 30, range 25-53;<br>35% female	Physicians in training: 75 internal medicine residents, 7 cardiology fellows, 6 pulmonary and critical care fellows (only 53 provided sufficient information on protective behaviours)	Respiratory protection: gloves, gowns, surgical masks, fit-tested N95 respirators, powered air-purified respirators, eye protection
Manabe et al. (2012)[15]	Vietnam	Avian influenza	326;<br>Physicians: 39.8% aged 35 or under, 39.8% aged 36-50, 20.4% aged 51 or over, Nurses: 35.6% aged 35 or under, 47.5% aged 36-50, 16.9% aged 51 or over, Lab technicians: 45.5% aged 35 or under, 48.5% aged 36-50, 6.1% aged 51 or over, Other: 43.5% aged	64.7% physicians, 18.1% nurses, 10.1% laboratory technicians, 7.1% other	Wearing PPE to see suspected H5N1 patients

For peer review only - http://bmjopen.bmj.com/site/about/guidelines.xhtml

			35 or under, 47.8% aged 36-50, 8.7% aged 51 or over;<br>43.8% female (doctors), 90.0% female (nurses), 84.8% female (lab technicians), 56.5% female (other)		
Nour et al. (2015)[16]	Saudi Arabia	MERS	281;<br>Mean age 30.8, range 21-57;<br>57.7% female	46.3% nurses, 20.6% physicians, 9.6% specialists, 23.5% technicians	Using soap and water to wash hands, covering nose and mouth with a tissue when sneezing or coughing, throwing used tissues away, avoiding touching eyes/nose/mouth, using facemask in crowds, carefully handling suspected patients' belongings, healthy eating/healthy lifestyle, educating patients about disease
Nour et al. (2017)[17]	Saudi Arabia	MERS	188 of the participants in Nour et al. (2015);<br>Age and gender for this sub-group not reported	188 of the participants in Nour et al. (2015);<br>Occupational role for this sub-group not reported	As reported in Nour et al. (2015)

For peer review only - http://bmjopen.bmj.com/site/about/guidelines.xhtml

Pratt et al. (2009)[18]	Canada	SARS	536;<br>Mean age 48.4;<br>96.6% female	Hospital nurses; Primary area of practice: 26.2% medicine, surgery; 19.5% maternal newborn, paediatrics; 29.4% emergency, critical care; 24.9% other	Cleaning hands with water and soap or waterless hand rubs after removing disposable gloves, wearing disposable gloves, wearing a disposable outer garment, wearing an N95 mask, wearing protective eyewear
Evirgen et al. (2014)[19]	Turkey	H1N1	22;<br>Mean age 31.95, range 19-48;<br>59.7% female	University hospital staff: 22 professors, 29 researchers, 26 nurses; 35% from surgical departments, 32% from internal medicine departments; 17% from operation room/intensive care unit; 14% from laboratories	Total score for protective behaviours including clearing the air in the environment, washing hands with soap after practice, rubbing hands with antiseptic after practice, using disinfectant at home, H1N1 vaccination, vaccination of children, recommending vaccination to at-risk groups, using medicines supporting the immune system, using alternative medicine drugs, using antivirals for prophylactic purposes, following scientific journals about H1N1 and looking at media statements on H1N1

For peer review only - http://bmjopen.bmj.com/site/about/guidelines.xhtml

Kim & Choi (2016)[20]	South Korea	MERS	249;<br>Mean age 21.9, range 20-32;<br>88.4% female	Nursing students in third or fourth year engaged in clinical practice for 8 hours a day at tertiary hospitals with MERS patients	Nine-item scale consisting of five items about reducing the use of public places in daily life, one item about avoiding people with cough, one item about intensive cleaning and disinfection, one item about handwashing and one item about talking with people nearby
Taghrir et al. (2020)[21]	Iran	COVID-19	240;<br>Mean age 23.67;<br>59.2% female	Fifth to seventh year medical students	Preventive behaviours: cancelling social activities, not using public transport, less frequent shopping, reducing the use of closed spaces, cough etiquette, avoiding large gatherings, increased cleaning and disinfecting of surfaces, handwashing, discussing prevention with family and friends
Jeong et al. (2011)[22]	South Korea	H1N1	880;<br>76.3% aged 18-39, 22.4% aged 40-59, 1.4% 60 or over;<br>64.5% female	Hospital employees	The Korea Center of Disease Control and Prevention's recommended behaviours, including washing hands, using a tissue when coughing or sneezing, reducing outings

For peer review only - http://bmjopen.bmj.com/site/about/guidelines.xhtml

Chor et al. (2012)[23]	China (Hong Kong), Singapore, UK	H1N1	2100 (1556 Hong Kong, 284 Singapore, 260 UK); Mean age 35 (Hong Kong), 28 (Singapore), 34 (UK); Gender not reported	Doctors, nurses, allied healthcare workers	Washing hands before each patient contact, washing hands after each patient contact, wearing gloves during patient contact, wearing a surgical mask even when not involved in direct patient care, wearing a mask during patient contact
Koh et al. (2009)[24]	Indonesia, Singapore	Avian influenza	333 from Indonesia v 1321 from Singapore plus 51 focus group participants from Indonesia; Quantitative study population: Indonesia: 55.4% aged 40 or under, Singapore: 43.6% aged 40 or under, Qualitative study: Age range 22-56; Quantitative study population: Indonesia: 75.7% female, Singapore: 86.4% female, Qualitative study: 94.1% female	Primary healthcare workers. Quantitative study: Indonesia: 17.6% doctors, 14.6% nurses, 67.8% other. Singapore: 14.6%, 34.2%, 51.2%. 'Other' includes therapists, paramedics, ambulance crew, attendants, pharmacists, social workers, cleaners and administrative staff Qualitative study: 13 physicians, 19 paramedics, 19 nurses	'Adhering to infection control protocols and recommended measures'

For peer review only

Wong et al. (2005)[25]	China (Hong Kong) (n=137), Canada (n=51)	SARS	188;<br>Age not reported;<br>Gender not reported	Family medicine tutors (Hong Kong), family physicians (Canada)	Testing patient temperature as a routine procedure, always wearing mask during consultations, cleaning work surface with antiseptics at least once a day, support staff all wearing masks, wearing gowns for patient encounters, washing hands between every patient, closing clinic, quarantine
Kamate et al. (2020)[26]	International (30.7% from Asia, 25.0% from North/South Americas, 16.3% from Europe, 22.6% from Africa, 5.4% from 'other' (Australia	COVID-19	860;<br>Age reported;<br>Gender not reported	Dental practitioners	Staff sensitised as per World Health Organization guidelines for the prevention of COVID-19, including travel history when recording patient histories, discussing risk and preventive measures related to COVID-19 with patients, showing patients signs and symptoms of COVID-19, taking 'preventive measures' against COVID-19, effect on social life

For peer review only - http://bmjopen.bmj.com/site/about/guidelines.xhtml

	and Antarctica)				
Hu et al. (2012)[27]	China	H1N1	650;<br>Median age 28, IQR 25-33;<br>76.3% female	Nurses (421), physicians (229)	Hand hygiene, gloves, gown, mask (surgical and N95 respirator), goggles
Alsahafi & Cheng (2016)[28]	Saudi Arabia	MERS	1216;<br>Age not reported;<br>56.4% female (nurses), 25.5% female (physicians), 23.9% female (other healthcare workers)	Nurses (56.3%), physicians (22%), other healthcare workers (21.7%)	Handwashing after patient contact, wearing of surgical mask during patient contact, wearing of N95 mask during patient contact, training on infectious behaviours, perceived barriers to infection control practices
Alsubaie et al. (2019)[29]	Saudi Arabia	MERS	591;<br>Age not reported;<br>78% female (non-physicians) and 31% female (physicians)	Physicians (55%), non-physicians – including nurses, technicians and respiratory therapists (45%)	Type of preventive behaviours and practices that changed during the outbreak, including hand hygiene and avoidance behaviours such as avoidance of hand-shaking, avoidance of direct contact with people suffering influenza-like illnesses and avoidance of public facilities

For peer review only

Chau et al. (2008)[30]	China (Hong Kong)	SARS	96 (observational study), 109 (interviews); Age not reported; Gender not reported	Healthcare workers and support workers; for interviews: 32 healthcare workers, 30 health care assistants, 47 managers including nursing officers, ward managers, nurse specialists and infection control nurses	Total performance score based on compliance with hand hygiene, mask use, gown use, goggle/face shield precautions, glove use, handling patient care equipment and articles correctly, routine and terminal cleaning, handling linen and laundry, and terminal cleaning of isolation room
Koh et al. (2005)[31]	Singapore	SARS	15,025; Mean age 36.6; 82% female	Healthcare workers from nine major healthcare institutions, incl. doctors, nurses, physiotherapists, attendants, cleaners, pharmacists, managerial staff, clerical staff, service counter staff, other	Hospital's recommended protective practices
May et al. (2010)[32]	USA	H1N1	261; Students: 26% aged under 24, 61% aged 24-29, 10% aged 30-34, 3% 35 and	Medical students (194) and residents (67), at an urban medical teaching centre	Hand sanitiser, handwashing, cough etiquette, disinfection, surgical mask when ill with influenza-like illness, surgical mask in patient care when

For peer review only - http://bmjopen.bmj.com/site/about/guidelines.xhtml

			over, Residents: 55% aged 24-29, 36% aged 30-34, 9% 35 and over; Students: 62% female, Residents: 50% female		seeing patients with influenza-like illness, N95 when seeing patients with influenza-like illness, social distancing
Mitchell et al. (2012)[33]	Canada	H1N1	986; Median age 36, range 21-79; 85% female	Nurses (80%), respiratory therapists (14%), physicians (6%)	Use of PPE
Parker & Goldman (2006)[34]	Canada	SARS	116; 27% aged 20-29, 42% aged 30-39, 23% aged 40-49, 8% aged 50 and over; 70% female	Paediatric emergency department staff: 28% physicians, 41% nursing staff, 21% trainees, 10% paramedical staff involved with direct patient care	Wearing N95 mask when examining patients, wearing gown when examining patients, handwashing before and after patient contact, wearing N95 mask at all times in the emergency department, wearing eye protection when examining patients, wearing gloves when examining patients
Shigayeva et al. (2007)[35]	Canada	SARS	795; Median age 41, range 21-67; 74.3% female	46.3% nurses, 13.5% physicians, 14.2% respiratory therapists, 10.3% medical imaging	Use of barrier precautions, practices for removing PPE, infection control training

For peer review only - http://bmjopen.bmj.com/site/about/guidelines.xhtml

				technologists, 15.7% other incl. service assistants, physiotherapists, lab technologists, paramedics, housekeeping staff, ward clerks and social workers	
Vinck et al. (2011)[36]	Netherlands	H1N1	166;<br>4% aged 25 or under, 26% aged 26-35, 26% aged 36-45, 32% aged 46-55, 10% aged 56-65;<br>66% female	Public health physicians (46%), public health nurses (51%), health department managers (2%)	Compliance with control measures: applying case definition (i.e. how consistently staff applied the criteria for case definition that was issued to identify suspected patients), amount of consultation with the centralised assessment system for the final classification of patients, use of PPE during patient sampling and home visits, and informing patients and contacts about isolation measures
Alshammari et al. (2018)[37]	Saudi Arabia	MERS	87;<br>Age not reported;<br>Gender not reported	Physicians and nurses	Hand hygiene compliance

For peer review only

Jaeger et al. (2011)[38]	USA	H1N1	63;<br>Median age 35, range 19-74;<br>76% female	70% worked in inpatient settings. 38% clinical practitioner, 10% physician/nurse practitioner/physician assistant, 29% Registered nurse/Registered Dietician, 52% allied health staff (incl. radiology, emergency department and respiratory technicians, phlebotomists, licensed vocational nurses and medical and nursing assistants), 10% support staff (incl. office workers, patient transport and environmental services workers)	PPE use, glove use during index patient exposures, mask or N95 respirator use during index patient encounters

For peer review only

Moore et al. (2005)[39]	Canada	SARS	97;<br>Mean age 43.1, range 26-64;<br>78% female	34% managers, 24% registered nurses, 9% support staff, 8% medical technologists, 6% respiratory therapists, 4% infection control practitioners, 4% physicians, 1% administration staff, 1% pharmacists and 1% physiotherapists	Experience of infection control practices including PPE
Tan et al. (2006)[40]	Singapore	SARS	8;<br>Age not reported;<br>25% female	Family physicians	Participants discussed encounters with suspected SARS patients including their clinical assessment and management of patients in clinics, problems encountered, and use of PPE and other preventive measures
Corley et al. (2010)[41]	Australia	H1N1	32 (questionnaire study), 16 (focus groups)<br>Age not reported;<br>Gender not reported	Questionnaire: 28 nursing staff, 4 medical staff;<br>Focus groups: 4 senior medical staff, 4 senior	How resources were managed during the pandemic

For peer review only - http://bmjopen.bmj.com/site/about/guidelines.xhtml

				nursing staff, 8 bedside nursing staff	
Hsu et al. (2011)[42]	Singapore	H1N1	123;<br>Age not reported;<br>Gender not reported	Final-year medical students	Hand hygiene
Chia et al. (2005)[43]	Singapore	SARS	6,198;<br>Mean age 35.8 (doctors), 34.6 (nurses), 38.4 (clerical staff);<br>27.1% female (doctors), 48.8% female (nurses), 77.1% female (clerical staff)	Doctors (14.1%), nurses (4404 – 71%), clerical staff (14.9%)	Perception of risk of infection and preventive measures
Ki et al. (2019)[44]	South Korea	MERS	446;<br>Age not reported;<br>Gender not reported	Medical employees; 228 in the emergency department, 218 in general wards	PPE (face shields or goggles, surgical masks, N95 respirators, gloves, disposable gowns, disposable coveralls, waterproof boots, head cover), hand-washing, hand-rubbing, isolation
Wong et al. (2004)[45]	China (Hong Kong)	SARS	320;<br>Mean age of general practitioners 44.4, mean	General practitioners (137), family medicine tutors (183)	Clinical practices and behaviours: wearing mask during all consultations, wearing goggles, wearing gowns,

For peer review only - http://bmjopen.bmj.com/site/about/guidelines.xhtml

			age of family medicine tutors not reported; 17.6% female (general practitioners), 15.3% female (family medicine tutors)		washing hands after seeing patients, insisting patients wear masks, routinely testing patients' temperatures, keeping greater distance from patients, seeing patients at faster pace, avoiding physical examination, requesting more blood tests/chest radiographs, over-prescribing antibiotics, cancelling own appointments, cancelling specialist appointments, difficulties making specialty referrals, closing clinic, reducing working hours, quarantine, requiring staff to wear masks/disposable gloves, cleaning work surfaces with antiseptics, taking staff temperatures, quarantined leave for staff, showering before leaving work, washing hands before entering home, using disinfectant to clean home, staying away from home, wearing a mask at home, sending family away

For peer review only

For peer review only - http://bmjopen.bmj.com/site/about/guidelines.xhtml

Yassi et al. (2005)[46]	Canada	SARS	Participants were those reported in Moore et al. (2005)	Participants were those reported in Moore et al. (2005)	Participants discussed barriers to wearing PPE and following infection control procedures
DiGiovanni et al. (2004)[47]	Canada	SARS	195;<br>Age not reported;<br>Gender not reported	76 physicians, 52 nurses, 67 'other' including receptionists, technicians, social workers, research assistants and clinic/office managers	Quarantine
Edeghere et al. (2015)[48]	UK	H1N1	20;<br>Mean age 43.9;<br>80% female	Frontline healthcare workers: 10 clinical attendants, 7 nurses, 3 general practitioners	Use of PPE and infection control measures
Khalid et al. (2016)[49]	Saudi Arabia	MERS	117;<br>Mean age 38.55;<br>76% female	Healthcare workers who worked in high-risk areas. 76% nurses, 14% physicians, 10% respiratory therapists. 65% worked in critical care units, 20% in emergency	Perceived stressors

For peer review only - http://bmjopen.bmj.com/site/about/guidelines.xhtml

				medicine, 15% in outpatient family medicine	
Locatelli et al. (2012)[50]	USA	H1N1	33;<br>Age not reported;<br>Gender not reported	Infection control key informants at nationally dispersed Veterans Affairs healthcare facilities	Communication about infection control
Rebmann & Wagner (2009)[51]	USA (n=37), UK (n=1, Canada (n=1), Australia (n=1)	H1N1	40;<br>2.5% aged 30-39, 27.5% aged 40-49, 45.0% aged 50-59, 22.5% aged 60-69, 2.5% 70 or over;<br>92% female	Infection preventionists	Issues relating to infection prevention guidance and PPE
Rowlands (2007)[52]	Singapore	SARS	28;<br>Age not reported;<br>75% female	Medical social workers	Experiences of working during the SARS crisis, and the various infection control strategies used at work
Van Dijk et al. (2015)[53]	Netherlands	H1N1	875;<br>General practitioners: 17.5% under 40, 24.5% aged 40-49, 44.9% aged 50-59, 13.2% aged 60+;	General practitioners (372), practice assistants (503)	Experience and acceptance of the national guidelines for infection control

For peer review only - http://bmjopen.bmj.com/site/about/guidelines.xhtml

			age for practice assistants not reported<br>General practitioners: 39.3% female; gender of practice assistants not reported		
Kang et al. (2018)[54]	South Korea	MERS	7;<br>Age not reported;<br>Gender not reported	Infection control nurse leaders from major hospitals	Use of PPE equipment
Wong et al. (2012)[55]	China (Hong Kong)	H1N1	10;<br>Mean age 34.9, range 24-49;<br>70% female	4 physicians, 3 nurses, 3 healthcare assistants all working in isolation wards	Concerns relating to care of patients with H1N1
Lam & Hung (2013)[56]	China (Hong Kong)	H1N1	10;<br>20% aged 20-25, 20% aged 26-30, 20% aged 31-35, 30% aged 36-40, 10% over 40;<br>100% female	Emergency nurses: 80% staff nurse, 10% advanced practice nurse, 10% nursing officer	Use of PPE
Nickell et al. (2004)[57]	Canada	SARS	2001;	33.1% allied health care professionals, 31.9% areas	Precautionary measures in the workplace: mask, restricted access (own

For peer review only - http://bmjopen.bmj.com/site/about/guidelines.xhtml

			13.6% aged under 30, 25.7% aged 30-39, 30.6% aged 40-49, 30.2% 50 or over; 78.8% female	other than patient care e.g. administration, food services, maintenance or research, 25.6% nurses, 9.3% doctors	hospital), restricted meetings, restricted access (other hospitals), gloves, goggles, 'other'
Kang et al. (2018)[58]	South Korea	MERS	27; Mean age 29.5; 92.6% female	Nurses	Working experiences and challenges faced during the outbreak
Khoo et al. (2005)[59]	Singapore	SARS	51; Age not reported; Female:male ratio was 2:1	19 doctors, 31 nurses/nursing aides, 1 respiratory therapist	Opinions on 3M powered air-purifying respirator and Stryker powered air-purifying respirator
Straus et al. (2004)[60]	Canada	SARS	14; Age not reported; Gender not reported	Physicians from divisions of infectious diseases, general internal medicine, and critical care medicine at university hospitals	Participants discussed barrier precautions such as protective clothing
Tolomiczenko et al. (2005)[61]	Canada	SARS	300; Mean age 42.6, range 20-75; 73.9% female	Registered nurses (25.2%), physicians (20.7%), management (9.5%), services (facilities management, food	Participants discussed patient care and communication about directives

For peer review only - http://bmjopen.bmj.com/site/about/guidelines.xhtml

				services, environmental – 3.7%), health professional (occupational therapists, physiotherapists, speech-language pathologists – 17.0%), other (counsellors, technicians, clerks, chaplains – 23.8%)	
Hsu et al. (2006)[62]	Taiwan	SARS	312;<br>4.0% under 30, 32.9% aged 30-39, 48.5% aged 40-49, 14.6% 50 over over;<br>Gender not reported	Public health nurses	Self-protection equipment
Nhan et al. (2012)[63]	Canada	H1N1	129;<br>Age not reported;<br>Gender not reported	68 infectious diseases specialist/medical microbiologists, 61 public health and preventive medicine specialists	Difficulties and frustrations experienced during the pandemic
Rambaldini et al. (2005)[64]	Canada	SARS	17;<br>Age not reported;	Medical house staff allocated to a general	Participants discussed hospitals' communication of directives

For peer review only - http://bmjopen.bmj.com/site/about/guidelines.xhtml

			Gender not reported	internal medicine clinical teaching unit, infectious disease unit, infectious diseases consultation service, or intensive care unit	
Robertson et al. (2004)[65]	Canada	SARS	10;<br>Mean age 43.1;<br>60% female	4 nurses, 1 hospital executive, 1 dentist, 1 social worker, 1 occupational therapist, 1 paramedic, 1 physician	Experiences of quarantine and perceptions of infection control guidelines
Goulia et al. (2010)[66]	Greece	H1N1	469;<br>Mean age 38.4;<br>68.4% female.	Nurses (44.6%), medical staff (25.6%), allied staff (12.6%), auxiliary staff (17.3%)	Restriction of social contacts, isolation, intended work avoidance, sense of duty

For peer review only

Supplementary Table II. Summary of themes

**Theme**	**Significant association with protective behaviours**	**No significant association with protective behaviours**	**Reported, but not statistically analysed as a potential predictor of protective behaviours**
Socio-demographics and personal characteristics			
Age	Kim & Choi (2016)[20]	Al-Amri et al. (2019)[12]<br>Datta et al. (2011)[13]<br>De Perio et al. (2012)[14]<br>Manabe et al. (2012)[15]<br>Nour et al. (2015)[16]<br>Nour et al. (2017)[17]<br>Pratt et al. (2009)[18]	N/A
Gender	Nour et al. (2015)[16]<br>Jeong et al. (2011)[22]<br>Vinck et al. (2011)[36]	Al-Amri et al. (2019)[12]<br>Datta et al. (2011)[13]<br>De Perio et al. (2012)[14]<br>Nour et al. (2017)[17]<br>Evirgen et al. (2014)[19]<br>Kim & Choi (2016)[20]	N/A

For peer review only - http://bmjopen.bmj.com/site/about/guidelines.xhtml

		Taghrir et al. (2020)[21]	
Nationality	Al-Amri et al. (2019)[12]	N/A	N/A
Country	Chor et al. (2012)[23]<br>Koh et al. (2009)[24]*<br>Wong et al. (2005)[25]*<br>Kamate et al. (2020)[26]*	Koh et al. (2009)[24]*<br>Wong et al. (2005)[25]*<br>Kamate et al. (2020)[26]*	N/A
Religion	N/A	Kim & Choi (2016)[20]	N/A
Socio-economic status	Jeong et al. (2011)[22]	N/A	N/A
Marital status	N/A	Pratt et al. (2009)[18]<br>Evirgen et al. (2014)[19]	N/A
Level of education	Al-Amri et al. (2019)[12]	Datta et al. (2011)[13]<br>Pratt et al. (2009)[18]<br>Kim & Choi (2016)[20]<br>Taghrir et al. (2020)[21]	N/A
Personal vulnerabilities	N/A	Evirgen et al. (2014)[19]	N/A
Vulnerable people at home	Evirgen et al. (2014)[19]*	Evirgen et al. (2014)[19]*	N/A
Vaccination status	Hu et al. (2012)[27]	N/A	N/A
Occupational role			

For peer review only - http://bmjopen.bmj.com/site/about/guidelines.xhtml

Role	Al-Amri et al. (2019)[12]<br>Pratt et al. (2009)[18]<br>Evirgen et al. (2014)[19]<br>Chor et al. (2012)[23]<br>Alsahafi & Cheng (2016)[28]<br>Alsubaie et al. (2019)[29]<br>Chau et al. (2008)[30]<br>Koh et al. (2005)[31]<br>Mitchell et al. (2012)[33]<br>Parker & Goldman (2006)[34]*<br>Shigayeva et al. (2007)[35]<br>Vinck et al. (2011)[36]<br>Chia et al. (2005)[43]	Datta et al. (2011)[13]<br>Nour et al. (2015)[16]<br>Nour et al. (2017)[17]<br>Parker & Goldman (2006)[34]*<br>Alshammari et al. (2018)[37]<br>Jaeger et al. (2011)[38]	May et al. (2010)[32]
Length of experience	Koh et al. (2005)[31]<br>Mitchell et al. (2012)[33]<br>Vinck et al. (2011)[36]	Al-Amri et al. (2019)[12]<br>Nour et al. (2015)[16]<br>Nour et al. (2017)[17]<br>Pratt et al. (2009)[18]<br>Chau et al. (2008)[30]	N/A
Training and knowledge			

For peer review only

Receiving training/education	May et al. (2010)[32]*<br>Shigayeva et al. (2007)[35]	Nour et al. (2017)[17]<br>Taghrir et al. (2020)[21]<br>May et al. (2010)[32]*	Alsahafi & Cheng (2016)[28]<br>Moore et al. (2005)[39]<br>Tan et al. (2006)[40]<br>Corley et al. (2010)[41]
Sources of knowledge	Al-Amri et al. (2019) [12]	Kim & Choi (2016)[20]<br>Taghrir et al. (2020)[21]	N/A
Knowledge (of correct practices / outbreak)	Yap et al. (2010)[4]<br>De Perio et al. (2012)[14] *<br>Nour et al. (2015)[16]<br>Kim & Choi (2016)[20]*<br>Jeong et al. (2011)[22]<br>Hu et al. (2012)[27]	Al-Amri et al. (2019)[12]<br>De Perio et al. (2012)[14] *<br>Kim & Choi (2016)[20]*	Alsahafi & Cheng (2016) [28]<br>Hsu et al. (2011)[42]
Work-related factors			
Setting	De Perio et al. (2012)[14]<br>Chau et al. (2008)[30]<br>Mitchell et al. (2012)[33]<br>Shigayeva et al. (2007)[35]<br>Jaeger et al. (2011)[38]<br>Chia et al. (2005)[43]<br>Ki et al. (2019)[44]*<br>Wong et al. (2004)[45]	Evirgen et al. (2014)[19]<br>Taghrir et al. (2020)[21]<br>Ki et al. (2019)[44]*	N/A

For peer review only - http://bmjopen.bmj.com/site/about/guidelines.xhtml

Contact with confirmed cases	De Perio et al. (2012)[14]<br>Pratt et al. (2009)[18]*<br>Shigayeva et al. (2007)[35]<br>Chia et al. (2005)[43]<br>Wong et al. (2004)[45]	Pratt et al. (2009)[18]*<br>Evirgen et al. (2014)[19]	N/A
Workload	Pratt et al. (2009)[18]<br>Chor et al. (2012)[23]*<br>Shigayeva et al. (2007)[35]<br>Vinck et al. (2011)[36]	Chor et al. (2012)[23]*	Chau et al. (2008)[30]<br>Moore et al. (2005)[39]<br>Corley et al. (2010)[41]<br>Yassi et al. (2005)[46]
Monitoring of compliance	Hu et al. (2012)[27]+	N/A	Hu et al. (2012)[27]+<br>Hsu et al. (2011)[42]<br>DiGiovanni et al. (2004)[47]
Patient encounters	De Perio et al. (2012)[14]<br>Shigayeva et al. (2007)[35]	N/A	N/A
Personal protective behaviour-related factors			
Availability of resources	Hu et al. (2012)[27]+<br>Mitchell et al. (2012)[33]*	Mitchell et al. (2012)[33]*	De Perio et al. (2012)[14]<br>Hu et al. (2012)[27]+<br>Moore et al. (2005)[39]<br>Tan et al. (2006)[40]

For peer review only - http://bmjopen.bmj.com/site/about/guidelines.xhtml

			Corley et al. (2010)[41]<br>Edeghere et al. (2015)[48]<br>Khalid et al. (2016)[49]<br>Locatelli et al. (2012)[50]<br>Rebmann & Wagner (2009)[51]<br>Rowlands (2007)[52]<br>Van Dijk et al. (2015)[53]<br>Hsu et al. (2006)[62]
Perceived difficulty of protective behaviour	N/A	N/A	Van Dijk et al. (2015)[53]<br>Kang et al. (2018)[54]
Logistical issues	N/A	N/A	Rebmann & Wagner (2009)[51]<br>Kang et al. (2018)[54]<br>Wong et al. (2012)[55]
Perceived effectiveness	Yap et al. (2010)[4]<br>Hu et al. (2012)[27]<br>Mitchell et al. (2012)[33]<br>Parker & Goldman (2006)[34]*	Parker & Goldman (2006)[34]*	Yassi et al. (2005)[46]<br>Kang et al. (2018)[54]
Perceived importance	N/A	N/A	Vinck et al. (2011)[36]<br>Hsu et al. (2011)[42]

For peer review only

Incentives	N/A	N/A	Jeong et al. (2011)[22]<br>Hsu et al. (2011)[42]<br>Kang et al. (2018)[54]
Convenience	N/A	N/A	De Perio et al. (2012)[14]<br>Hu et al. (2012)[27]<br>Moore et al. (2005)[39]<br>Tan et al. (2006)[40]<br>Corley et al. (2010)[41]<br>Hsu et al. (2011)[42]<br>Kang et al. (2018)[54]<br>Lam & Hung (2013)[56]<br>Nickell et al. (2004)[57]
Comfort	Mitchell et al. (2012)[33]*	Mitchell et al. (2012)[33]*	Moore et al. (2005)[39]<br>Tan et al. (2006)[40]<br>Corley et al. (2010)[41]<br>Yassi et al. (2005)[46]<br>Khalid et al. (2016)[49]<br>Rowlands (2007)[52]<br>Lam & Hung (2013)[56]<br>Nickell et al. (2004)[57]<br>Kang et al. (2018)[58]

For peer review only - http://bmjopen.bmj.com/site/about/guidelines.xhtml

			Khoo et al. (2005)[59]<br>Straus et al. (2004)[60]
Impact on patient care	Hu et al. (2012)[27]+	N/A	Hu et al. (2012)[27]+<br>Tan et al. (2006)[40]<br>Corley et al. (2010)[41]<br>Rowlands (2007)[52]<br>Lam & Hung (2013)[56]<br>Nickell et al. (2004)[57]<br>Khoo et al. (2005)[59]<br>Straus et al. (2004)[60]<br>Tolomiczenko et al. (2005)[61]
Guidance			
Issues with availability or understanding	N/A	N/A	Alsahafi & Cheng (2016)[28]<br>Chau et al. (2008)[30]<br>Moore et al. (2005)[39]<br>Tan et al. (2006)[40]<br>Corley et al. (2010)[41]<br>Yassi et al. (2005)[46]<br>Locatelli et al. (2012)[50]<br>Rebmann & Wagner (2009)[51]<br>Van Dijk et al. (2015)[53]

For peer review only

For peer review only - http://bmjopen.bmj.com/site/about/guidelines.xhtml

			Kang et al. (2018)[54]<br>Wong et al. (2012)[55]<br>Lam & Hung (2013)[56]<br>Kang et al. (2018)[58]<br>Tolomiczenko et al. (2005)[61]<br>Hsu et al. (2006)[62]<br>Nhan et al. (2012)[63]<br>Rambaldini et al. (2005)[64]<br>Robertson et al. (2004)[65]
Distress and risk perception			
Distress	Chia et al. (2005)[43]<br>Wong et al. (2004)[45]	N/A	DiGiovanni et al. (2004)[47]
Risk perception	Kim & Choi (2016)[20]<br>Taghrir et al. (2020)[21]<br>Jeong et al. (2011)[22]<br>Chor et al. (2012)[23]*<br>Parker & Goldman (2006)[34]<br>Goulia et al. (2010)[66]	Chor et al. (2012)[23]*	Moore et al. (2005)[39]<br>DiGiovanni et al. (2004)[47]<br>Rebmann & Wagner (2009)[51]
Attitudes and behaviours of others			

For peer review only

Non-compliance of others	N/A	N/A	Hu et al. (2012)[27]<br>Chau et al. (2008)[30]<br>Moore et al. (2005)[39]<br>Hsu et al. (2011)[42]<br>Yassi et al. (2005)[46]<br>DiGiovanni et al. (2004)[47]<br>Locatelli et al. (2012)[50]
Attitudes of family members	N/A	N/A	Moore et al. (2005)[39]<br>Yassi et al. (2005)[46]
Other potential predictors of compliance			
Complacency	N/A	N/A	Tan et al. (2006)[40]
Commitment	N/A	N/A	Alsahafi & Cheng (2016)[28]<br>Moore et al. (2005)[39]
Previous experience with pandemic	N/A	N/A	Moore et al. (2005)[39]
Passing of time	N/A	N/A	Rebmann & Wagner (2009)[51]
Forgetting	Hu et al. (2012)[27]	N/A	N/A
Death of a doctor	N/A	N/A	Tan et al. (2006)[40]

For peer review only - http://bmjopen.bmj.com/site/about/guidelines.xhtml

Effort-reward imbalance	Pratt et al. (2009)[18]	N/A	N/A
Consulting with experts	N/A	Manabe et al. (2012)[15]	N/A
Lack of staff	N/A	N/A	Moore et al. (2005)[39]<br>Corley et al. (2010)[41]
PPE fit-testing procedures	N/A	N/A	Moore et al. (2005)[39]
Professionalism	N/A	N/A	Yassi et al. (2005)[46]
Use of public transport	N/A	Evirgen et al. (2014)[19]	N/A
Loss of income	N/A	N/A	DiGiovanni et al. (2004)[47]
Overcrowding	N/A	N/A	Alsahafi & Cheng (2016)[28]

*Appears in both 'significant association' and 'no significant association' column due to both significant and non-significant results being reported for different groups of participants.
+Contains both statistically analysed data and non-statistically analysed (e.g. qualitative data, descriptive survey results).

For peer review only

For peer review only - http://bmjopen.bmj.com/site/about/guidelines.xhtml

Supplementary Table III. Summary of evidence

Theme	Evidence
Socio-demographics and personal characteristics	
Age	Al-Amri et al. (2019)[12]: Compliance was not significantly associated with age (p=0.785).
	Datta et al. (2011)[13]: Protective behaviours were not significantly associated with age.
	De Perio et al. (2012)[14]: Compliance was not significantly associated with age.
	Manabe et al. (2012)[15]: For physicians only (other roles not included in analysis), age was not significantly associated with wearing PPE to see suspected avian influenza patients.
	Nour et al. (2015)[16]: There was no significant difference in median protective practice scores between age groups (under 30s v 30 and over) (p=0.582).
	Nour et al. (2017)[17]: There was no significant difference in median protective practice scores between age groups (under 30s v 30 and over).
	Pratt et al. (2009)[18]: Age was not significantly associated with compliance.
	Kim & Choi (2016)[20]: Age was significantly positively correlated with preventive behaviours (p<0.05); older staff were more likely to comply.
Gender	Al-Amri et al. (2019)[12]:Compliance was not significantly associated with gender (p=0.241).
	Datta et al. (2011)[13]: Protective behaviours were not significantly associated with gender.
	DePerio et al. (2012)[14]: Compliance was not significantly associated with gender.

For peer review only - http://bmjopen.bmj.com/site/about/guidelines.xhtml

	Nour et al. (2015)[16]: Female staff were significantly more likely to comply with preventive behaviours (p<0.001).
	Nour et al. (2017)[17]: There was no significant difference in median protective practice scores between genders.
	Evirgen et al. (2014)[19]: Behaviour was not significantly associated with gender.
	Kim & Choi (2016)[20]: Gender was not significantly associated with preventive behaviours.
	Taghrir et al. (2020)[21]: There was no significant association between preventive behaviours and gender.
	Jeong et al. (2011)[22]: Female gender was a significant predictor of preventive behaviours.
	Vinck et al. (2011)[36]: Female staff reported higher compliance than males (p=0.002) and greater use of PPE (p=0.037).
Nationality	Al-Amri et al. (2019)[12]:Compliance was significantly associated with nationality (p=0.047), with Saudi staff showing higher compliance than non-Saudi staff in the same city.
Country	Chor et al. (2012)[23]: Hong Kong reported the highest compliance with all infection control practices, except for wearing a mask during patient contact, which was second to Singapore (70.4% v 87.7%). Wearing masks in clinical areas while not involved with patient contact was lowest for the UK (25.3% v 96.4% for Hong Kong and 82.3% for Singapore), as was use of masks (either surgical or N95) during physical contact with patients (62% for the UK v 70.4% for Hong Kong and 87.7% for Singapore).
	Koh et al. (2009)[24]: Healthcare workers in Singapore were significantly more likely than those in Indonesia to screen patients and clinic visitors for fever (92.9% v 80.8%, p<0.001), limit number of visitors to the clinic (88.7% v 46.0%, p<0.001), post prominent infection control notices to patients/visitors (97.4% v 80.3%, p<0.001), do temperature checks for staff (91.5% v 61.1%, p<0.001); wear gowns (91.1% v 61.5%, p<0.001); wear paper masks (43.2% v 11.7%, p<0.001); wear surgical masks (28.6% v 12.6%, p<0.001); wear N95 masks (94.0% v 82.4%, p=0.004); and carry out regular handwashing (98.6% v 87.0%, p<0.001). However, there were no significant differences between countries in wearing gloves (94.2% v 93.3%, p=0.502); wearing goggles (77.6% v 67.8%, p=0.140); using powered air purifier

For peer review only - http://bmjopen.bmj.com/site/about/guidelines.xhtml

	respirators (86.1% v 85.4%, p=0.550); using alcohol hand rub (95.6% v 90.8%, p=0.183); or using a special room to isolate bird flu patients (97.9% v 97.5%, p=0.254).
	Wong et al. (2005)[25]: Physicians in Hong Kong were less likely than those in Canada to quarantine themselves after contact with a probable or suspected SARS case (19.4% v 77.1%, p<0.01) or give quarantine leave to their staff (59.7% v 95%, p<0.01). However, physicians in Hong Kong were more likely than those in Canada to test patient temperature as a routine procedure (68.1% v 47.1%, p<0.01), wear a mask during consultations (97.7% v 52.9%, p<0.01) and have their support staff wear masks (97.8% v 68.6%, p<0.01). No significant differences between countries were found for wearing gowns for every patient encounter or washing hands between every encounter.
	Kamate et al. (2020)[26]: There were no significant differences between countries in terms of discussing risk of COVID-19 with patients; discussing preventive measures with patients; showing patients the signs and symptoms of COVIF-19; or taking preventive measures themselves. However, there was a significant difference in whether they included travel history while recording patients' histories (99.0% in Asia v 97.2% in Americas v 94.3% in Europe v 94.3% in Africa v 89.4% in Australia/Antarctica, p=0.025).
Religion	Kim & Choi (2016)[20]: Being religious or not was not significantly associated with preventive behaviours.
Socio-economic status	Jeong et al. (2011)[22]: 'High or middle economic state' significantly predicted preventive behaviours.
Marital status	Pratt et al. (2009)[18]: Marital status was not significantly associated with compliance.
	Evirgen et al. (2014)[19]: Behaviour was not significantly associated with marital status.

For peer review only - http://bmjopen.bmj.com/site/about/guidelines.xhtml

Level of education	Al-Amri et al. (2019)[12]: Compliance was significantly associated with qualifications (p=0.037) with more highly-qualified staff being more likely to comply. Percentage of poor, good and excellent preventive practices, by qualification: MBBS: 30.6%, 69.4%, 0.0%; Diploma: 46.7%, 46.7%, 6.7%; Masters: 43.8%, 43.8%, 12.5%; Doctorate/fellowship: 66.7%, 22.2%, 11.1%.
	Datta et al. (2011)[13]: Protective behaviours were not significantly associated with level of education.
	Pratt et al. (2009)[18]: Highest level of education was not significantly associated with compliance.
	Kim & Choi (2016)[20]: Level of education (junior/senior grade) was not significantly associated with compliance.
	Taghrir et al. (2020)[21]: There was no significant association between preventive behaviours and education level.
Personal vulnerabilities	Evirgen et al. (2014)[19]: Behaviour was not significantly associated with being pregnant or having a chronic disease.
Vulnerable people at home	Evirgen et al. (2014)[19]: Behaviour was not significantly associated with having a pregnant spouse, elderly person, or school-aged child at home. However, staff with babies at home were more likely to comply with protective behaviours ($p<0.005$).
Vaccination status	Hu et al. (2012)[27]: Vaccination for H1N1 influenza was an independent predictor of high compliance to PPE ($p<0.001$).
Occupational role	
Role	Al-Amri et al. (2019)[12]: Practice-related adherence to guidelines differed significantly according to position (p=0.035), with specialists having the highest percentage of excellent practice.
	Datta et al. (2011)[13]: Protective behaviours were not significantly associated with occupation.
	Nour et al. (2015)[16]: There was no significant difference in median protective practice scores between occupational groups (p=0.433).

For peer review only - http://bmjopen.bmj.com/site/about/guidelines.xhtml

	Nour et al. (2017)[17]: There was no significant difference in median protective practice scores between occupational groups (nurses v physicians v specialists v technicians).
	Pratt et al. (2009)[18]: Job status (full-time, part-time or casual) was reported to be significantly associated with compliance although the authors do not state which group were more likely to comply. Medical/surgical unit as a primary area of practice (compared to maternal/newborn or paediatrics, emergency or critical care, or 'other' roles) was also reported to be significantly associated with compliance but again the authors do not state in which way.
	Evirgen et al. (2014)[19]: Professors had the highest average score for positive behaviours.
	Chor et al. (2012)[23]: In Hong Kong and Singapore, employment type was the strongest predictor of adoption of infection control practices, with nurses showing significantly higher compliance with most measures compared to doctors in both countries, and allied healthcare workers also reporting significantly higher compliance with most measures compared to doctors in Hong Kong. Administrative staff in Hong Kong reported compliance similar to that of other healthcare workers, whereas administrative staff in the UK reported significantly lower compliance than doctors.
	Alsahafi & Cheng (2016)[28]: There appeared to be significant differences between physicians, nurses and other healthcare staff in terms of handwashing after patient contact ($p=0.04$), wearing of surgical mask during patient contact ($p<0.001$), and wearing of N95 mask during patient contact ($p=0.009$). It is not clear from the data presented which groups are significantly more likely to comply, but perhaps nurses are slightly more likely to comply than physicians who are slightly more likely to comply than other healthcare staff.
	Alsubaie et al. (2019)[29]: Non-physicians were significantly more likely than physicians to report improvement (that is, improvement since pre-outbreak) in hand hygiene compliance at the hospital (90% v 80%, $p<0.002$), improvement in compliance with universal precautions at the hospital (85.5% v 69%, $p=0.001$), increase in avoidance behaviour towards people with flu-like symptoms (83% v 72%, $p<0.009$), decrease in using public facilities (66% v 28.7%,

	p=0.001), decrease in hand-shaking (60% v 27.7%, p<0.001), and decrease in social visits e.g. to friends (60% v 21.7%, p<0.001).
	Chau et al. (2008)[30]: Nurses performed better in a number of isolation precautions (including hand-washing, hand rub use, glove use, handling of linen and laundry, and handling of patient care equipment) than other roles. Doctors performed better when it came to wearing masks and putting on gowns.
	Koh et al. (2005)[31]: All other occupational groups, including nurses (OR 0.49; 95% CI 0.39-0.61) reported greater difficulty adhering to preventive measures compared with doctors.
	May et al. (2010)[32]: Medical students appeared more likely than residents to use hand sanitiser both at work and home, wash hands both at work and home, and practice cough etiquette at work and home, while residents appeared more likely to use disinfectant and use surgical masks and N95 masks when seeing patients with influenza-like illness – however, no statistical analysis is presented.
	Mitchell et al. (2012)[33]: Compared with physicians, nurses and respiratory therapists were significantly more compliant with wearing N95 respirators and gloves for aerosol-generating medical procedures and with wearing protective eyewear and gloves for patient care not involving these procedures. Nurses and respiratory therapists were also significantly more compliant with performing a user seal check after donning an N95 respirator than physicians (35% and 40% v 19%, p=0.023).
	Parker & Goldman (2006)[34]: Physicians reported more handwashing before and after all patient contacts compared with nurses and trainees (p<0.05); however, there were no significant differences between in occupational groups in compliance with wearing an N95 mask when examining patients, wearing a gown when examining patients, wearing an N95 mask at all times in the emergency department, wearing eye protection when examining patients, or wearing gloves when examining patients.

For peer review only - http://bmjopen.bmj.com/site/about/guidelines.xhtml

	Shigayeva et al. (2007)[35]: Clinically trained health professional status was reported to be a significant predictor of consistent adherence (statistics not reported). Safe removal of PPE was significantly associated with being a nurse (OR 1.3, 95% CI, 1.1-1.6).
	Vinck et al. (2011)[36]: Public health physicians followed the case definition less strictly than public health nurses (p=0.000).
	Alshammari et al. (2018)[37]: No significant difference in hand hygiene compliance between physicians and nurses.
	Jaeger et al. (2011)[38]: Use of PPE stratified by healthcare role did not yield significant differences.
	Chia et al. (2005)[43]: The risk of using a less effective level of respiratory protection was lowest among doctors, followed by nurses and clerical staff.
Length of experience	Al-Amri et al. (2019)[12]:Compliance was not significantly associated with length of experience in primary care (p=0.265).
	Nour et al. (2015)[16]: There was no significant association between median protective practice scores and years of experience (p=0.674).
	Nour et al. (2017)[17]: There was no significant association between median protective practice scores and years of experience.
	Pratt et al. (2009)[18]: Years of nursing experience were not significantly associated with compliance.
	Chau et al. (2008)[30]: Compliance was not significantly associated with years of experience.
	Koh et al. (2005)[31]: The longer the work experience, the less likely staff were to experience difficulty in adhering to preventive measures (OR 1.21; 95% CI 1.13-1.29).

For peer review only

For peer review only

	Mitchell et al. (2012)[33]: Staff who had worked in health care for more than eleven years were significantly more compliant with wearing protective eyewear during aerosol-generating medical procedures than those who had worked for eleven years or less (69% v 54%, p<0.001), and also with wearing gowns (82% v 69%, p<0.001), gloves (92% v 85%, p=0.006) and N95 respirators (75% v 60%, p<0.001). For patient care not involving aerosol-generating medical procedures, staff with more than eleven years of experience were again significantly more likely to comply with wearing protective eyewear (38% v 31%, p=0.04), gowns (82% v 69%, p<0.001), N95 respirators (43% v 31%, p=0.003) and surgical masks (18% v 11%, p<0.016).
	Vinck et al. (2011)[36]: Compliance was higher in those who had been working in the public health service for one to ten years than those who had worked for eleven years or longer (p=0.034).
Training and knowledge	
Receiving training/education	Nour et al. (2017)[17]: After the training intervention in the study, there was a non-significant increase in preventive practices (p=0.168) – but compliance was good pre-intervention too.
	Taghrir et al. (2020)[21]: There was no significant association between preventive behaviours and having received education on COVID-19.
	Alsahafi & Cheng (2016)[28]: 22.8% of participants reported having received training about dealing with infectious disease outbreaks, 37.1% training in infection control policies and procedures, 54.4% training in hand hygiene and 45.6% training in N95 mask-wearing techniques. A high proportion of participants believed that insufficient training in infection control procedures contributed to poor compliance and the risk of infection (92.4% of nurses, 91% of physicians and 90.9% of other healthcare staff).

	May et al. (2010)[32]: There was a significant association between level of training and use of currently recommended CDC guidelines for patients with influenza-like illness, for fellows only (p=0.003); however, the number of fellows within the sample was small (n=5).
	Shigayeva et al. (2007)[35]: Recent infection control training was a significant predictor of consistent adherence to recommended behaviours (interactive training: OR 2.7, 95% CI, 1.7-4.4; passive training: OR 1.7, 95% CI, 1.0-3.0).
	Moore et al. (2005)[39]: Many participants reported having received poor prior training in infection control and therefore having to use new procedures and equipment without training or experience.
	Tan et al. (2006)[40]: Participants reported that little information on the disease or methods of control were available from previous education and training, which made them feel helpless.
	Corley et al. (2010)[41]: Participants believed that an infection control course with yearly refreshers would be useful and help improve compliance.
Sources of knowledge	Al-Amri et al. (2019)[12]: Those who attended CME activities had significantly higher levels of excellent practice than those who did not attend a CME activity (11.1% vs 1.5%, p=0.002). Participants who had textbooks as a source of knowledge had the highest percent of excellent practice (p<0.001).
	Kim & Choi (2016)[20]: Source of knowledge of MERS (television, or other) was not significantly associated with preventive behaviours.
	Taghrir et al. (2020)[21]: There was no significant association between preventive behaviours and source of information (WHO, CDC, or UpToDate v national guidelines v both v others).
Knowledge (of correct practices / outbreak)	Yap et al. (2010)[4]: There was a significant positive correlation between knowledge about H1N1/preventive behaviours, and practice of preventive behaviours (p<0.01).
	Al-Amri et al. (2019)[12]: Practice-related adherence to guidelines was not significantly associated with knowledge.

For peer review only

	De Perio et al. (2012)[14]: Knowledge of correct PPE was significantly associated with compliance at one hospital (p=0.047) but not the other three hospitals in the study.
	Nour et al. (2015)[16]: There was a significant positive association between knowledge and practice (p<0.001).
	Kim & Choi (2016)[20]: Having received MERS education was not significantly associated with preventive behaviours (p=0.209). However, in a correlational analysis, preventive behaviours were significantly correlated with MERS-related knowledge (p<0.01).
	Jeong et al. (2011)[22]: A score for 'cues to action' (consisting of consulting news or articles about the pandemic, being interested in methods for health promotion, and wishing to be educated on effective ways of preventing infection) was significantly associated with compliance (OR 2.63, CI 1.81-3.81).
	Hu et al. (2012)[27]: Self-reported adequate knowledge of self-protection and patient protection was significantly correlated with the perception of further improvement of PPE compliance (p<0.001).
	Alsahafi & Cheng (2016)[28]: A high proportion of participants believed that lack of knowledge about the mode of transmission contributed to poor compliance (94.2% of nurses, 90% of physicians and 90.2% of other healthcare staff).
	Hsu et al. (2011)[42]: 1.9% of participants believed lack of education about correct practices prevented hand hygiene compliance. 6.5% of participants believed improving undergraduate education efforts was the most important strategy for improving hand hygiene compliance. 7.3% of participants believed incorporating hand hygiene into examinations and the student internship marking scheme was the most important strategy.
Work-related factors	
Setting	De Perio et al. (2012)[14]: Staff who worked on an intensive care unit rotation during the study period were more likely to be in the 'high adherence' group (OR, 3.43; 95% CI, 1.02-11.56). Staff who worked on an inpatient ward rotation were less likely to be classified as high adherence (OR, 0.22, 95% CI, 0.06-0.80).

For peer review only - http://bmjopen.bmj.com/site/about/guidelines.xhtml

	Evirgen et al. (2014)[19]: Behaviour was not significantly associated with department at work or the department where the participant spends the most time.
	Taghrir et al. (2020)[21]: There was no significant association between preventive behaviours and ward worked on.
	Hu et al. (2012)[27]: Compliance rates significantly differed between institutions, from 0% to 88.1%, although all institutions were adult intensive care units.
	Chau et al. (2008)[30]: Those working in rehabilitation hospitals were significantly more likely to comply with protective behaviours overall, and use hand rub, than participants working in acute hospitals.
	Mitchell et al. (2012)[33]: Staff working in an intensive care unit or designated influenza ward were more compliant with wearing an N95 respirator for aerosol-generating medical procedures than those working in an emergency department (78% and 67% v 47%, p<0.001).
	Shigayeva et al. (2007)[35]: Working in a SARS unit was significantly associated with consistent adherence to recommended guidelines (OR 4.0, 95% CI, 1.8-8.9), as was working in an intensive care unit (OR 4.3, 95% CI, 2.0-9.0). Employment in a teaching hospital (OR 7.6, 95% CI, 4.8-11.9), SARS unit (OR 3.4, 95% CI, 1.3-9.4) or an intensive care unit (OR 1.9, 95% CI, 0.7-5.0) were associated with safer removal of PPE.
	Jaeger et al. (2011)[38]: 68% of staff in outpatient settings reported no PPE use with index patients, compared to 9% of staff in inpatient setting (p<0.01). Mask or N95 respirator use during index patient contact was more common in inpatient (41%) than outpatient settings (5%) (p<0.01). Glove use was more common among inpatient (57%) than outpatient (21%) settings. Always donning gloves, gown and either mask or N95 respirator was more common in inpatient (18%) than outpatient (5%) (p<0.01).
	Chia et al. (2005)[43]: Using administrative staff as the reference group, individuals working in surgical, medical and intensive care units were more likely to wear the appropriate respiratory protection. However, there was no significant

For peer review only - http://bmjopen.bmj.com/site/about/guidelines.xhtml

	difference for those individuals in the other areas (A&E, radiographic services, laboratory services, and others) compared to administrative personnel.
	Ki et al. (2019)[44]: Staff working in the emergency department were significantly more likely than staff in general wards to wear surgical masks (93.0% v 1.8%, p<0.001); however, there were no significant differences in hand-washing or wearing gloves.
	Wong et al. (2004)[45]: Staff working in high-infection districts were significantly more likely to wear gowns (63.0% v 42.0%, p<0.05), wash their hands before going home (82.6% v 63.0%, p<0.05) and use disinfectants to clean surfaces regularly (87.0% v 65.4%, p<0.01); however, they were less likely to quarantine themselves (6.5% v 27.5%, p<0.01) or grant leave to staff until confirmation of diagnosis (41.3% v 71.3%, p<0.01). Staff in public clinics were significantly more likely to wear gowns during consultations than staff in private clinics (81.3% v 45.3%, p<0.05); however, staff in private clinics were more likely to quarantine themselves for a 10-day period after SARS contact than their public sector colleagues (58.1% v 31.3%, p<0.05). Staff in the public sector were more likely to stay away from home.
Contact with confirmed cases	De Perio et al. (2012)[14]: Staff who were present at an aerosol-generating procedure on a patient with pH1N1 (OR 5.20, 95% CI, 1.54-17.55) were more likely to be in the high adherence group.
	Pratt et al. (2009)[18]: Nurses' experience of working directly with confirmed SARS cases was a significant predictor of compliance (p<0.001), with nurses who worked with SARS patients more likely to comply than those who did not. However, this was no longer significant after combining the impact of effort-reward imbalance and burnout.
	Evirgen et al. (2014)[19]: Behaviour was not significantly associated with awareness of suspected or confirmed H1N1 infection in their hospital or their department, or having contact with H1N1 cases.
	Shigayeva et al. (2007)[35]: Recognition of the patient as a SARS case was a significant predictor of consistent adherence to recommended barrier precautions (OR 2.5, 95% CI, 1.5-4.5).

For peer review only - http://bmjopen.bmj.com/site/about/guidelines.xhtml

	Chia et al. (2005)[43]: Staff who regularly came into contact with SARS patients were more likely to wear appropriate respiratory protection compared to those who did not (OR 0.673, 95% CI, 0.587-0.771).
	Wong et al. (2004)[45]: SARS-exposed staff were significantly more likely to insist on patients wearing masks (65.1% v 48.1%, p<0.05) and more likely to ensure their staff wore gloves and masks all the time (33.7% v 11.5%, p<0.01 for gloves, 100% v 92.3%, p<0.01 for masks). However, they were also less likely to quarantine themselves until confirmation of diagnosis (10.8% v 33.3%, p<0.01).
Workload	Pratt et al. (2009)[18]: Hours worked per week were reportedly significantly associated with compliance; however, the authors do not give details about whether more or less hours are associated with greater or lower compliance.
	Chor et al. (2012)[23]: There was a significant decrease in compliance with handwashing before patient contact with the increase in number of weekly patient contacts only in Hong Kong; no significant difference was noted in Singapore or the UK.
	Chau et al. (2008)[30]: Workload was a common barrier to compliance identified by participants.
	Shigayeva et al. (2007)[35]: Work on shifts requiring more frequent patient room entry was associated with lower compliance (OR for 6 or more entries per shift: 0.5, 95% CI, 0.32-0.86). The reported rate of consistent adherence was lower for staff members who reported entering a patient's room more than 5 times per shift: consistent adherence was reported for 84.1% of procedures involving staff members who reported entering the room 1-5 times, compared with 75.5% of procedures involving staff members who reported entering the room 6 or more times (p<0.001).
	Vinck et al. (2011)[36]: The compliance to measures to properly identify suspected cases of respondents who regularly worked overtime was reduced compared with those who did not (p=0.024). Conversely, working overtime was associated with increased compliance with informing patients that they were not supposed to leave their home while ill (p=0.048) and providing patients with the information leaflet (p=0.002). Additionally, compliance with control

For peer review only - http://bmjopen.bmj.com/site/about/guidelines.xhtml

	measures was not influenced by the number of inhabitants within the catchment area of the public health service or by the number of cases for which consultation within the centralised assessment system of each public health service with the Preparedness and Response Unit was carried out (objective workload).
	Moore et al. (2005)[39]: Fatigue was cited as a major cause of failing to follow the proper guidelines, with exhaustion leading to 'sloppy practice'.
	Corley et al. (2010)[41]: Isolation procedures and preventive behaviours created extra workload, frustration and confusion - educating and monitoring external staff and patient's relatives regarding PPE was an extra duty for already overstretched staff and was time-consuming and frustrating.
	Yassi et al. (2005)[46]: The increased time constraints and increased workload were felt to be important barriers to worker adherence to recommendations.
Monitoring of compliance	Hu et al. (2012)[27]: Significantly more nurses than physicians (92.1% v 86.4%, p=0.020) reported being reprimanded by their supervisor for non-compliance. Perceived reprimand by supervisor for non-compliance was a significant predictor of compliance (p=0.035).
	Hsu et al. (2011)[42]: 4.1% of participants believed better policing by infection control staff was the most important strategy for improving hand hygiene, and 3.3% thought that improving public awareness (patients monitoring doctors) was the most important.
	DiGiovanni et al. (2004)[47]: Public health authorities' monitoring of compliance played an important role in establishing the credibility of quarantine for 75% of physicians, 81% of nurses, and 85% of other healthcare workers: however, 58% of physicians, 37% of nurses and 40% of other staff rated the monitoring of their own compliance as bad.

For peer review only - http://bmjopen.bmj.com/site/about/guidelines.xhtml

Patient encounters	De Perio et al. (2012)[14]: 55% of those who reported not using the recommended PPE (n=40) stated it was because they did not know the patient had H1N1 or an influenza-like illness; 20% stated they did not think PPE was needed for the particular activity they were doing; 18% stated it was because the only entered the patients' room for a brief time; 13% stated it was because they did not touch the patient and 10% stated it was because they did not come within six feet of the patient.
	Shigayeva et al. (2007)[35]: Lower rates of consistent adherence were found among staff providing care for patients with higher Acute Physiology and Chronic Health Evaluation (APACHE) II scores – i.e. more severe illness (OR for score APACHE II of 20 or greater: 0.4, 95% CI, 0.28-0.68). Reported rates of consistent adherence were higher when healthcare workers were themselves performing procedures (adjusted OR: 1.7, 95% CI, 1.4-2.2) or assisting with them (adjusted OR: 1.5, 95% CI, 1.1-2.1), compared with procedures that they observed (81% v 79.1% v 74.2%, p<0.001).
Personal protective behaviour-related factors	
Availability of resources	De Perio et al. (2012)[14]: 18% of those who reported not using the recommended PPE (n=40) stated it was because their facility had run out of PPE, while 10% reported that their facility did not provide PPE.
	Hu et al. (2012)[27]: 63.2% reported that appropriate PPE was readily available. Availability of PPE in the ICU was significantly associated with compliance.
	Mitchell et al. (2012)[33]: Staff were significantly more compliant with PPE use when eyewear and gloves were readily available at the point of care; however, PPE availability did not increase compliance with the use of N95 respirators, surgical masks, or gowns.
	Moore et al. (2005)[39]: Participants reported supply problems with face shields and goggles.

For peer review only - http://bmjopen.bmj.com/site/about/guidelines.xhtml

	Tan et al. (2006)[40]: Many participants reported that they had not been properly mask-fitted due to lack of proper testing equipment in their clinic. Many were not provided with gowns. Several participants reported lack of goggles and visual shields in their clinics.
	Corley et al. (2010)[41]: Participants believed there were not enough PPE supplies and that guidelines changed depending on supplies running out – for example, surgical masks and plastic aprons became acceptable due to the lack of other PPE. One staff member reported wearing an incorrectly sized mask as their correct size was not in stock.
	Edeghere et al. (2015)[48]: Only 20% of participants experienced barriers in applying infection control measures; the main reported barrier was the occasional unavailability of recommended PPE, most often eye protection.
	Khalid et al. (2016)[49]: 60% perceived inadequate protective measures available to them and cited this as a stressor.
	Locatelli et al. (2012)[50]: Participants reported that stocks of N95 masks ran low so they started using surgical masks, which made it difficult to enforce the use of N95s.
	Rebmann & Wagner (2009)[51]: Many participants reported a lack of resources. Less than half of those whose facility had requested supplies from external agencies actually received them, whereas three participants whose facilities had not requested supplies did receive them. Many reported that supplies were back-ordered and they could not receive them in a timely manner. Theft of supplies was also an issue, with masks and respirators presumably being used or taken by visitors. 26.5% said their facility ran out of respiratory protection. Many reported staff in their facilities were wearing the wrong sizes as they were unable to get the correct sizes.
	Rowlands (2007)[52]: Participants reported that there were not enough masks for staff.
	Van Dijk et al. (2015)[53]: 73.5% believed they had sufficient personal protective materials; 26.5% believed they were insufficient.
	Hsu et al. (2006)[62]: 44% expressed dissatisfaction with shortage of protective equipment such as N95 masks and gloves.

For peer review only - http://bmjopen.bmj.com/site/about/guidelines.xhtml

Perceived difficulty of protective behaviour	Van Dijk et al. (2015)[53]: 35.0% believed recommended personal protective measures were feasible.
	Kang et al. (2018)[54]: Participants reported difficulties with the complexity of using several PPE items together.
Logistical issues	Rebmann & Wagner (2009)[51]: Participants reported logistical issues with needing to fit-test large numbers of staff in a short time.
	Kang et al. (2018)[54]: Limited hospital layouts disrupted space management for PPE use.
	Wong et al. (2012)[55]: Barriers to PPE use included inadequate shower facilities for staff, the small size of the changing room, and the physical layout of the two-bed isolation room which was poor for infectious case management.
Perceived effectiveness	Yap et al. (2010)[4]: There was a significant correlation between attitudes (an 'attitude' score was based on responses to questions on attitudes towards the use of PPE, vaccination, Tamiflu, help-seeking and risk perception) ($r=0.24$, $p<0.01$); there was no such correlation for cases or close contacts who answered the same questions.
	Hu et al. (2012)[27]: Positive attitude towards PPE use (i.e. belief that it provided adequate protection) significantly predicted compliance ($p=0.001$).
	Mitchell et al. (2012)[33]: Staff who felt better protected against influenza when they wore an N95 respirator were significantly more compliant with N95 respirator use than those who did not ($p<0.001$ for aerosol-generating medical procedures and $p=0.04$ for patient care not involving these procedures).
	Parker & Goldman (2006)[34]: Staff considering the combination of all infection control measures to be highly effective in protecting patients from SARS reported increased use of a mask when examining patients compared with staff considering all measures less effective ($p=0.05$). However, handwashing, glove use, gown use, eye protection use, and wearing a mask at all times in the emergency department did not significantly differ between the two groups.

For peer review only - http://bmjopen.bmj.com/site/about/guidelines.xhtml

	Yassi et al. (2005)[46]: Beliefs in the effectiveness of infection control guidelines, as modified by past experiences, were identified as having important influence on worker adherence to procedures.
	Kang et al. (2018)[54]: Participants expressed doubts about the quality and effectiveness of PPE.
Perceived importance	Vinck et al. (2011)[36]: Compliance with the recommendation to consult the Preparedness and Response Unit for centralised assessment of patients was fairly low, with the main reasons for non-compliance being finding it unnecessary (38.3%) or time-consuming (22.7%).
	Hsu et al. (2011)[42]: 20.1% believed that failure to recognise the importance of hand hygiene prevented compliance
Incentives	Jeong et al. (2011)[22]: Perceived benefits of the preventive behaviours significantly predicted higher compliance (OR 3.18, CI 2.15-4.68).
	Hsu et al. (2011)[42]: 19.9% of participants believed that providing positive incentives for hand hygiene was the single most important factor in improving compliance.
	Kang et al. (2018)[54]: Participants reported a lack of incentives to comply with preventive behaviours.
Convenience	De Perio et al. (2012)[14]: 30% of those who reported not using the recommended PPE (n=40) stated it was because the PPE was not available near their patients' room; 10% felt too busy to wear PPE; and 10% stated it was inconvenient to use.
	Hu et al. (2012)[27]: Half of participants reported that PPE use was inconvenient.
	Moore et al. (2005)[39]: Some participants reported that PPE was time-consuming due to having to constantly gown, de-gown, and re-gown.
	Tan et al. (2006)[40]: Participants reported that gowns were inconvenient during toilet breaks.

For peer review only - http://bmjopen.bmj.com/site/about/guidelines.xhtml

	Corley et al. (2010)[41]: Application and removal of PPE was reported to be time-consuming.
	Hsu et al. (2011)[42]: 60.4% believed lack of time prevented hand hygiene compliance.
	Kang et al. (2018)[54]: PPE use was inconvenient in the presence of hospital visitors.
	Lam & Hung (2013)[56]: Participants reported that gowning and de-gowning was very time-consuming.
	Nickell et al. (2004)[57]: 41.7% of participants felt precautionary measures affected their ability to do their job.
Comfort	Mitchell et al. (2012)[33]: Staff who reported always or often feeling comfortable wearing protective eyewear were significantly more likely to wear it ($p<0.001$). Similarly, staff who reported always or often feeling comfortable wearing an N95 respirator were significantly more compliant with N95 use. Less than 19% of staff reported always or often feeling short of breath, claustrophobic or dizzy when wearing protective eyewear or N95 respirators; no significant differences in compliance were observed between these staff and staff who reported never or rarely feeling short of breath, claustrophobic or dizzy.
	Moore et al. (2005)[39]: Some participants reported that PPE was uncomfortable.
	Tan et al. (2006)[40]: Participants reported physical discomfort from prolonged use of N95 masks, including difficulty breathing, headache, and development of allergic facial rash; they reported moments of complacency where they removed masks intermittently in order to allow normal breathing.
	Corley et al. (2010)[41]: Wearing PPE for extended periods was difficult as it was uncomfortable and could cause dehydration and injuries such as skin peeling.
	Yassi et al. (2005)[46]: Participants identified the need to address the increased amount of worker fatigue that existed when staff worked with full PPE, noting that the discomfort associated with wearing PPE was an important barriers to worker adherence to recommendations.

For peer review only

	Khalid et al. (2016)[49]: Participants reported that having to wear PPE was stressful.
	Rowlands (2007)[52]: Participants reported that masks were warm and uncomfortable, often leading them to develop headaches or skin rashes, and made it hard to breathe.
	Kang et al. (2018)[54]: Many participants reported having ill-fitted PPE.
	Lam & Hung (2013)[56]: Participants reported that PPE was uncomfortable.
	Nickell et al. (2004)[57]: 85.4% of participants found masks bothersome; of these, 92.9% stated they caused physical discomfort and 13.0% said they gave them a sense of isolation.
	Kang et al. (2018)[58]: Participants reported finding it stressful that their masks did not fit properly and emphasised the discomfort of PPE: they found it hard to breathe or see clearly while wearing it, which made their work harder, and it made them sweaty and dizzy when they wore it for too long.
	Khoo et al. (2005)[59]: Participant opinions on 3M powered air-purifying respirator: 2.3% found it uncomfortable, 11.6% reported moderate discomfort, 32.6% found it tolerable, 30.2% reported slight discomfort, and 23.3% found it very comfortable. 2.3% reported unacceptable visual impairment. 2.3% felt it was suffocating, 4.6% found breathing uncomfortable, 7.0% reported it was comfortable but moderately hard to breathe, 60.5% found it comfortable with minimal difficulty breathing, and 25.6% reported no breathing discomfort. Compared to Stryker powered air-purifying respirator: 4.5% found it uncomfortable, 4.5% reported moderate discomfort, 13.7% found it tolerable, 31.8% reported slight discomfort, and 45.5% found it very comfortable. 4.5% reported unacceptable visual impairment. 0% felt suffocated.
	Straus et al. (2004)[60]: Participants reported that masks hurt, made them sweat and gave them headaches.

For peer review only

Impact on patient care	Hu et al. (2012)[27]: 21.2% believed that PPE use would interfere with patient care (no significant difference between nurses and physicians). The perception that PPE use would interfere with patient care was significantly associated with poor compliance (p=0.002).
	Tan et al. (2006)[40]: Staff reported that masks muffled their speech and they had to raise their voices to communicate with patients. They also reported that patient reactions to PPE ranged from amusement to apprehension and anxiety; some saw their doctor wearing PPE and assumed they had been exposed to SARS and could infect them and their families.
	Corley et al. (2010)[41]: Wearing PPE was reported to make communication difficult.
	Rowlands (2007)[52]: Participants reported that masks impacted the patient counselling process as they compromised their communication skills and made their speech muffled. They also believed that psychiatric patients with paranoia were sensitive to masks.
	Lam & Hung (2013)[56]: Participants reported that PPE affected their communication with patients, making them have to speak very loudly in order to be heard.
	Nickell et al. (2004)[57]: 85.4% of participants found masks bothersome; of these, 47.0% stated that they caused difficulties communicating.
	Khoo et al. (2005)[59]: Some difficulties regarding communication. Opinions on the 3M powered air-purifying respirator: 0% felt they had to shout but 25.6% felt they had to raise their voice significantly, 37.2% had to raise voice moderately, 32.6% raised voice normally, and 4.6% could speak normally. 2.3% said it caused unacceptable hearing impairment, 11.6% significant, 27.9% moderate, 41.9% mild and 16.3% hearing impairment. Compared to the Stryker powered air-purifying respirator: 0% had to shout, 27.3% raised voice significantly, 31.8% raised voice moderately, 27.3% raised voice mildly, and 13.7% could speak normally. 0% reported unacceptable hearing impairment. About two-thirds of

For peer review only

	respondents agreed (22%) or strongly agreed (42%) that they looked frightening to their patients whenever they used the PAPR.
	Straus et al. (2004)[60]: Participants reported that wearing protective equipment made assessing and communicating with patients a challenge, as they could not establish any of the usual non-verbal cues with patients. They were concerned that patients may have been seen less frequently than usual because of the time required to get into protective clothing.
	Tolomiczenko et al. (2005)[61]: Nurses, more than other occupational groups, reported that infection control measures had an adverse impact on patient care because they were less ‘visible’ to their patients when gowned, masked and gloved.
Guidance	
Issues with availability or understanding	Alsahafi & Cheng (2016)[28]: Almost two thirds of participants were unaware of guidelines or protocols for the care of infected patients. A high proportion believed that compliance was negatively affected by the lack of policies and procedures (87.3% of nurses, 85% of doctors, 89.4% of other healthcare staff).
	Chau et al. (2008)[30]: Issues with communication and understanding were cited as barriers to compliance. Participants expressed mixed views on the guidelines; some described them as easy to follow and facilitating increased alertness, while others found them difficult to follow and too long (and suggested they be given summaries instead, to get a basic idea of the principles). Other suggestions included tailored information; more programmes in Chinese with a more interactive approach to learning; making courses more practical; and educating the public and patients too.
	Moore et al. (2005)[39]: Participants reported a lack of consistency with safety instructions and frequently changing directives. New levels of enforcement by regulatory agencies interfered with rational infection control practice and infection control policies developed elsewhere had little perceived relevance to their own workplace. Basic infection control policies and procedures were frequently perceived as not well-developed. A major problem was perceived to be

For peer review only - http://bmjopen.bmj.com/site/about/guidelines.xhtml

	issues with communicating the guidelines: participants reported it was difficult to disseminate information simultaneously to all staff; often new information was sent by email, which was not checked before going to work.
	Tan et al. (2006)[40]: Some participants reported not wearing gowns or gloves due to inadequate dissemination of information/instructions from health authorities. However, a SARS seminar and hotline were organised which were perceived to be an effective channel of communication to address concerns and queries on PPE.
	Corley et al. (2010)[41]: Participants perceived a lack of firm recommendations/guidelines regarding what specific PPE was required, which created confusion and made them feel unprotected. They also remarked that current guidelines changed too frequently to keep up. Participants also perceived a lack of clear guidelines and conflicting advice about what to do when a patient is deemed non-infectious.
	Yassi et al. (2005)[46]: To improve adherence to infection control guidelines, participants felt that better enforcement of infection control guidelines was needed but did not want such enforcement to rely on nurses "policing" other professionals.
	Locatelli et al. (2012)[50]: Participants reported many barriers to effective communication about infection control, including information overload, often contradictory information from different sources, and difficulty integrating information from various sources and working out what was a priority. 10 participants reported not having a pre-defined communication plan, or discovered flaws with their plan. Some reported that everyone had to develop their own guidelines, with no clear guidance or resources on how to do so provided. This resulted in confusion about the use of PPE.
	Rebmann & Wagner (2009)[51]: Participants reported a lack of guidance for alternate care sites and non-acute care settings such as physician offices, ambulatory care clinics, long-term care and nursing homes, as well as a general lack of infection prevention information and guidance related to occupational health issues. Rapidly-changing practice

For peer review only - http://bmjopen.bmj.com/site/about/guidelines.xhtml

	recommendations were often contradictory which made it difficult to maintain staff compliance and trust, and to stay abreast of the latest recommendations. There was a perceived lack of consistency between sources, with CDC, state health departments, and city health departments all saying different things. Participants reported gaps in communication between departments, or between hospitals and physician offices. Many did not know which type of mask or respirator to use when caring for H1N1 patients, and reported conflicting guidelines related to isolation, masks, and respirator use. 34.3% reported their facility had changed PPE use guidelines and 34.3% reported isolation guidelines had been changed midway through the H1N1 response.
	Van Dijk et al. (2015)[53]: 71.8% believed information provision was well-timed, 65.1% believed information provision was complete, 59.9% believed infection prevention policy changes were well-communicated, 59.2% believed information was explicitly formulated, and 68.5% felt they were clear on when to take protection measures.
	Kang et al. (2018)[54]: Participants described confusion from non-standardized and constantly evolving protocols, making it difficult to develop appropriate PPE protocols and training.
	Wong et al. (2012)[55]: Participants reported difficulties with guidelines as they changed so often.
	Lam & Hung (2013)[56]: Half the participants were satisfied that departmental communication about guidelines was effective, but others had problems with excessive guidelines and rapidly-changing regulations.
	Kang et al. (2018)[58]: Participants cited frequently changing guidelines as a stressor.
	Tolomiczenko et al. (2005)[61]: Emergency room personnel and intensive care unit personnel, more than other groups, reported poor communication about SARS and false ideas about safety precautions, adding that they were not kept up-to-date about what to wear and that personnel at the hospital entrance had no idea what the rules of the day were regarding what should be worn and when.

For peer review only - http://bmjopen.bmj.com/site/about/guidelines.xhtml

	Hsu et al. (2006)[62]: 37% participants reported poor communication between agencies and 30% reported a lack of standard operating procedures.
	Nhan et al. (2012)[63]: Participants reported that communication of guidelines was a barrier to compliance: guidelines were inconsistent between different pandemic management levels and advisory committees, often lacked clarity, were from an overwhelming number of different sources, frequently changed, and were slow to be communicated. Guidelines were also perceived as too rigid to accommodate particular regional or local situations.
	Rambaldini et al. (2005)[64]: Participants reported variability in the information made available at different hospitals, the perceived inconsistency of interhospital and interdepartmental interpretation of public health directives, and the lack of communication between institutions. Some reported frustration at receiving information filtered down from other sources, rather than first-hand. Residents at other hospitals, however, reported receiving regular updates from program directors, chief medical residents and infection control specialists in a variety of forms including websites, email and direct communication via personal conversations or group meetings. The most important information was deemed to be changes to infection control protocols, the status of SARS at their hospital and within the community, and the clinical condition of affected health care colleagues.
	Robertson et al. (2004)[65]: Participants reported a lack of clear guidelines about how to minimise infection at home and in quarantine and uncertainty about effective risk control, leading them to fear contaminating their family members. Unclear guidelines meant that staff developed their own thoughts about which behaviours were necessary and which were not.
Distress and risk perception	

For peer review only - http://bmjopen.bmj.com/site/about/guidelines.xhtml

Distress	Chia et al. (2005)[43]: Staff with IES scores of 20 or greater were more likely to wear more effective respiratory protection than those with scores of 19 or less.
	Wong et al. (2004)[45]: Staff who were highly anxious/frightened of dealing with SARS were significantly more likely to insist on patients wearing masks (68.1% v 48.5%, $p<0.05$), to ensure their staff wore gloves at work (32.4% v 17.9%, $p<0.05$), and to wash their hands before entering their home (79.4% v 61.2%, $p<0.05$).
	DiGiovanni et al. (2004)[47]: 5% reported such high stress they were tempted to break quarantine.
Risk perception	Kim & Choi (2016)[20]: Risk perception was significantly positively correlated with preventive behaviours ($p<0.01$).
	Taghrir et al. (2020)[21]: There was a significant negative correlation between preventive behaviours and fear (fear of becoming infected and thinking they are more likely to become infected than others) – as preventive behaviours increased, risk perception declined.
	Jeong et al. (2011)[22]: Perceived susceptibility to the pandemic significantly predicted preventive behaviours.
	Chor et al. (2012)[23]: In Hong Kong, compliance with infection control practices (wearing gloves, changing gloves and washing hands before each patient contact) was associated with the perceived seriousness of the likely impact of pandemic H1N1 on their life; however, compliance with these practices did not correlate with the perceived disease severity among staff in Singapore or the UK.
	Parker & Goldman (2006)[34]: Considering SARS to be a high, rather than low, public health threat was significantly associated with increased compliance with hand-washing, wearing a mask at all times in the emergency department, and wearing gloves when examining patients ($p<0.05$).
	Moore et al. (2005)[39]: Some participants reported that nobody ‘cut corners’ as fear of infection was very real.

For peer review only - http://bmjopen.bmj.com/site/about/guidelines.xhtml

	DiGiovanni et al. (2004)[47]: Staff who directly cared for SARS patients feared becoming infected and passing along infection to their families, so they not only complied with quarantine but also restricted social contact more strictly and for longer than required, with many sending their families away or living alone in their basements; 94% reported the most important reason for complying was to reduce the risk of transmission to others.
	Rebmann & Wagner (2009)[51]: Participants believed that healthcare workers' perceptions of H1N1 severity led to poor compliance with PPE; for example, one participant reported that staff chose not to wear PPE with the idea that they wanted to get mild flu so they wouldn't get a more virulent form of the disease in the autumn.
	Goulia et al. (2010)[66]: Degree of worry about H1N1 was significantly associated with restricting social contacts outside of work because the work environment was considered dangerous ($p<0.0005$).
Attitudes and behaviours of others	
Non-compliance of others	Hu et al. (2012)[27]: 21% reported their colleagues often forgot to use PPE during patient care.
	Chau et al. (2008)[30]: A common barrier to compliance identified by participants was poor preventive practices by colleagues, patients and visitors.
	Moore et al. (2005)[39]: Basic infection control policies and procedures were frequently perceived as not being enforced; participants identified various deficiencies including tracking who received training, lack of consistent policies for quarantining people, reuse of masks, and lack of consistency in deciding which patients require negative pressure rooms.
	Hsu et al. (2011)[42]: 56.1% of participants believed that senior clinicians leading by example was the most important strategy for improving hand hygiene compliance, and 10.1% believed that peer example (i.e. senior staff not practicing

For peer review only - http://bmjopen.bmj.com/site/about/guidelines.xhtml

	hand hygiene) was a reason for poor compliance. 2.4% of participants believed support and emphasis of guidelines by top administrative staff was the most important strategy for improving hand hygiene compliance.
	Yassi et al. (2005)[46]: The peer environment, especially the compliance of other occupational groups (including physicians), and the feedback from peers were identified as factors that could exert positive or negative influence on individual worker actions.
	DiGiovanni et al. (2004)[47]: As hospitals began to experience critical staff shortages, many modified the recommended quarantine guidelines to allow their staff to return.
	Locatelli et al. (2012)[50]: Participants reported resistance from administration staff about putting up signs with precautions, because they thought it might cause panic.
Attitudes of family members	Moore et al. (2005)[39]: Participants reported that a facilitator of compliance was the attitude of family members, who wanted them to comply with recommended measures as they were scared of getting sick and were often angry or upset that their healthcare worker family member was continuing to work.
	Yassi et al. (2005)[46]: Attitudes of family members, in particular the fear that family members expressed toward contracting SARS, influenced workers' behaviour.
Other potential predictors of compliance	
Complacency	Tan et al. (2006)[40]: Participants reported not wearing gowns due to complacency.
Commitment	Alsahafi & Cheng (2016)[28]: 96.2% of nurses, 93.6% of physicians and 98.4% of other healthcare staff believed that healthcare workers' lack of commitment to policies and procedures reduced compliance.

For peer review only - http://bmjopen.bmj.com/site/about/guidelines.xhtml

	Moore et al. (2005)[39]: Participants reported a lack of commitment to occupational health and safety by workers and management.
Previous experience with pandemic	Moore et al. (2005)[39]: Some participants reported that past exposures to disease led to decreased compliance when experience demonstrated that protective behaviours are not needed 100% of the time.
Passing of time	Rebmann & Wagner (2009)[51]: Participants reported that compliance lowered with time, as it was hard to keep vigilant in the middle of outbreak.
Forgetting	Hu et al. (2012)[27]: Approximately 21% reported they forgot to change PPE between patients. Forgetting to use or change PPE was significantly associated with poor compliance ($p<0.001$).
Death of a doctor	Tan et al. (2006)[40]: Compliance improved after the death of a doctor friend.
Effort-reward imbalance	Pratt et al. (2009)[18]: Effort-reward imbalance ratio was significantly predictive of decreased compliance ($p<0.05$).
Consulting with experts	Manabe et al. (2012)[15]: Consulting with experts with experience of caring for H5N1 patients did not significantly predict compliance with recommended behaviours ($p=0.776$).
Lack of staff	Moore et al. (2005)[39]: Participants felt there were not enough infection control practitioners, or they were not visible enough.
	Corley et al. (2010)[41]: Participants reported that not having enough staff (or enough experienced staff working at once) was a barrier to compliance.
PPE fit-testing procedures	Moore et al. (2005)[39]: Participants reported the fit-testing process for PPE was not standardised and some felt it was unnecessary.
Professionalism	Yassi et al. (2005)[46]: Professionalism was identified as having an important influence on adherence to procedures.

For peer review only - http://bmjopen.bmj.com/site/about/guidelines.xhtml

Use of public transport	Evirgen et al. (2014)[19]: Behaviour was not significantly associated with using public transportation to get to work.
Loss of income	DiGiovanni et al. (2004)[47]: Fear of loss of income was an obstacle to complying with quarantine, particularly among those unconvinced quarantine was necessary – 60% of doctors, 76% of nurses and 70% of other healthcare workers said they would need fairly detailed information about when, how, and how much compensation they would receive as encouragement to comply with quarantine.
Overcrowding	Alsahafi & Cheng (2016)[28]: Overcrowding in the emergency department was cited as a reason for poor compliance (97.2% of nurses, 96.6% of physicians, 93.2% of other healthcare staff)

For peer review only

For peer review only - http://bmjopen.bmj.com/site/about/guidelines.xhtml

Appendix I. Search strategy

Search 1: Compliance-related terms

compliance OR comply OR adhere* OR attitude* OR behaviour* OR behavior*

Search 2: Infection control-related terms

hygien* OR handwashing OR hand-washing OR infection control OR mask* OR PPE OR protective equipment OR social distanc*

Search 3: Healthcare worker-related terms

healthcare worker* OR healthcare staff OR healthcare personnel OR "medical personnel" OR "hospital personnel" OR frontline OR medical staff OR medical worker* OR doctor* OR dentist* OR midwife* OR midwives OR surgeon* OR nurse* OR gp* OR general practitioner* OR physician* OR health care worker* OR health care staff OR hospital employee* OR medical employee* OR healthcare employee* OR health care employee* OR hospital worker* OR hospital staff

Search 4: Emerging infectious disease-related terms

SARS OR severe acute respiratory syndrome OR MERS OR middle east respiratory syndrome OR H1N1 OR swine flu OR H5N1 OR avian influenza OR bird flu OR pandemic* OR coronavirus OR COVID-19 OR n-COV OR SARS-COV-2

Searches 1, 2, 3 and 4 were combined with AND.

For peer review only

Appendix II. Flow diagram of screening process

PRISMA 2009 Flow Diagram

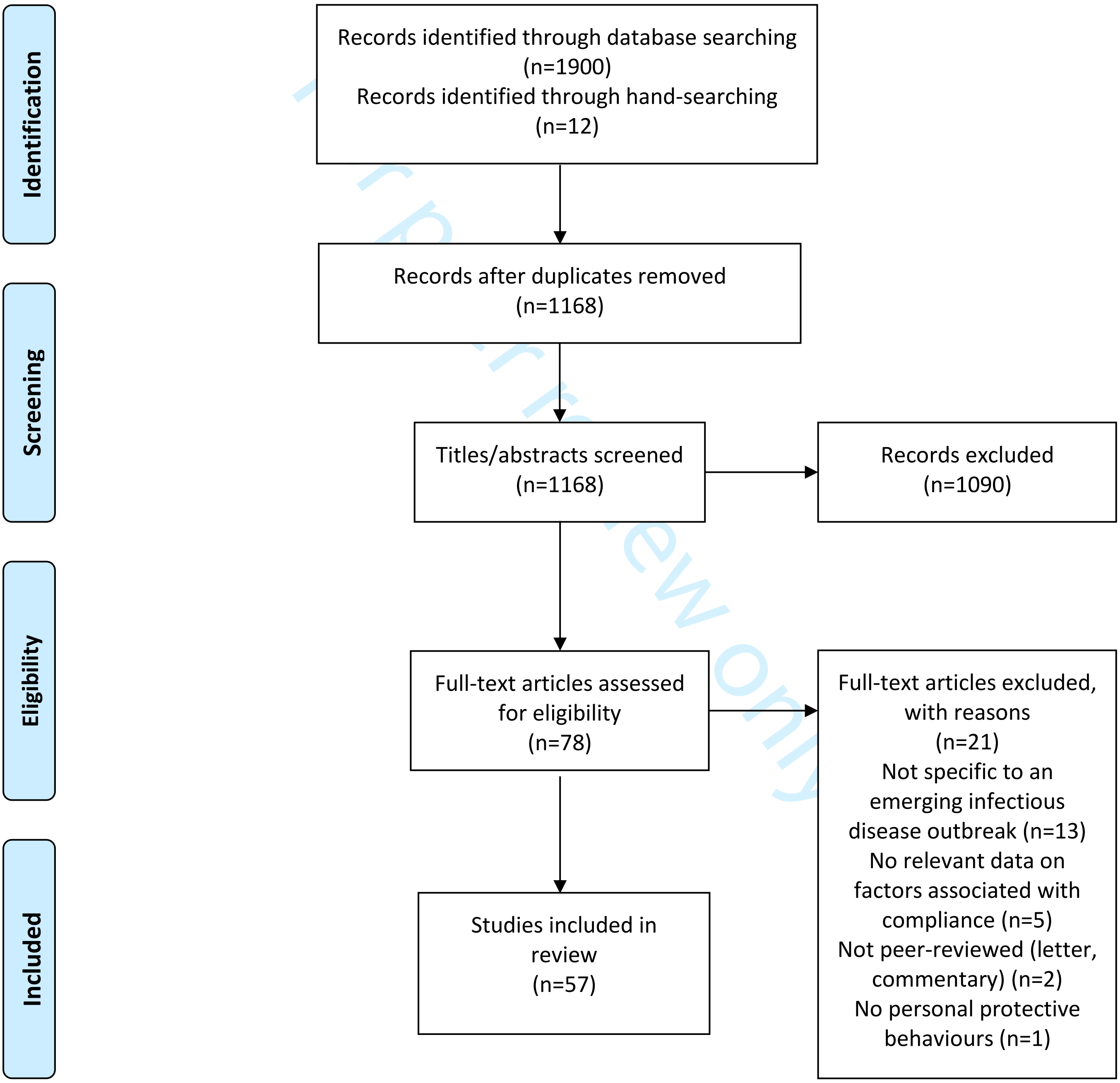

For peer review only - http://bmjopen.bmj.com/site/about/guidelines.xhtml

PRISMA 2009 Checklist

Section/topic	#	Checklist item	Reported on page #
**TITLE**			
Title	1	Identify the report as a systematic review, meta-analysis, or both.	1
**ABSTRACT**			
Structured summary	2	Provide a structured summary including, as applicable: background; objectives; data sources; study eligibility criteria, participants, and interventions; study appraisal and synthesis methods; results; limitations; conclusions and implications of key findings; systematic review registration number.	2
**INTRODUCTION**			
Rationale	3	Describe the rationale for the review in the context of what is already known.	3
Objectives	4	Provide an explicit statement of questions being addressed with reference to participants, interventions, comparisons, outcomes, and study design (PICOS).	3
**METHODS**			
Protocol and registration	5	Indicate if a review protocol exists, if and where it can be accessed (e.g., Web address), and, if available, provide registration information including registration number.	n/a
Eligibility criteria	6	Specify study characteristics (e.g., PICOS, length of follow-up) and report characteristics (e.g., years considered, language, publication status) used as criteria for eligibility, giving rationale.	4
Information sources	7	Describe all information sources (e.g., databases with dates of coverage, contact with study authors to identify additional studies) in the search and date last searched.	4
Search	8	Present full electronic search strategy for at least one database, including any limits used, such that it could be repeated.	Supplementary material
Study selection	9	State the process for selecting studies (i.e., screening, eligibility, included in systematic review, and, if applicable, included in the meta-analysis).	4
Data collection process	10	Describe method of data extraction from reports (e.g., piloted forms, independently, in duplicate) and any processes for obtaining and confirming data from investigators.	4
Data items	11	List and define all variables for which data were sought (e.g., PICOS, funding sources) and any assumptions and simplifications made.	4
Risk of bias in individual studies	12	Describe methods used for assessing risk of bias of individual studies (including specification of whether this was done at the study or outcome level), and how this information is to be used in any data synthesis.	n/a – rapid review so not all steps of a systematic review were

For peer review only - http://bmjopen.bmj.com/site/about/guidelines.xhtml

PRISMA 2009 Checklist

Section/topic	#	Checklist item	Reported on page #
			followed, as recommended by WHO (2017) guidelines
Summary measures	13	State the principal summary measures (e.g., risk ratio, difference in means).	n/a
Synthesis of results	14	Describe the methods of handling data and combining results of studies, if done, including measures of consistency (e.g., I^2) for each meta-analysis.	4
Risk of bias across studies	15	Specify any assessment of risk of bias that may affect the cumulative evidence (e.g., publication bias, selective reporting within studies).	n/a
Additional analyses	16	Describe methods of additional analyses (e.g., sensitivity or subgroup analyses, meta-regression), if done, indicating which were pre-specified.	n/a
**RESULTS**			
Study selection	17	Give numbers of studies screened, assessed for eligibility, and included in the review, with reasons for exclusions at each stage, ideally with a flow diagram.	5 / supplementary material
Study characteristics	18	For each study, present characteristics for which data were extracted (e.g., study size, PICOS, follow-up period) and provide the citations.	Supplementary material
Risk of bias within studies	19	Present data on risk of bias of each study and, if available, any outcome level assessment (see item 12).	n/a
Results of individual studies	20	For all outcomes considered (benefits or harms), present, for each study: (a) simple summary data for each intervention group (b) effect estimates and confidence intervals, ideally with a forest plot.	n/a
Synthesis of results	21	Present results of each meta-analysis done, including confidence intervals and measures of consistency.	n/a
Risk of bias across studies	22	Present results of any assessment of risk of bias across studies (see Item 15).	n/a
Additional analysis	23	Give results of additional analyses, if done (e.g., sensitivity or subgroup analyses, meta-regression [see Item 16]).	n/a
**DISCUSSION**			
Summary of evidence	24	Summarize the main findings including the strength of evidence for each main outcome; consider their relevance to key groups (e.g., healthcare providers, users, and policy makers).	11-12

For peer review only - http://bmjopen.bmj.com/site/about/guidelines.xhtml

PRISMA 2009 Checklist

Limitations	25	Discuss limitations at study and outcome level (e.g., risk of bias), and at review-level (e.g., incomplete retrieval of identified research, reporting bias).	13-14
Conclusions	26	Provide a general interpretation of the results in the context of other evidence, and implications for future research.	14
**FUNDING**			
Funding	27	Describe sources of funding for the systematic review and other support (e.g., supply of data); role of funders for the systematic review.	Title page (submitted as supplementary material for editor)

From: Moher D, Liberati A, Tetzlaff J, Altman DG, The PRISMA Group (2009). Preferred Reporting Items for Systematic Reviews and Meta-Analyses: The PRISMA Statement. PLoS Med 6(7): e1000097. doi:10.1371/journal.pmed1000097

For more information, visit: **www.prisma-statement.org**.

For peer review only

BMJ Open

Factors affecting healthcare workers’ compliance with social and behavioural infection control measures during emerging infectious disease outbreaks: Rapid evidence review.

Journal:	*BMJ Open*
Manuscript ID	bmjopen-2021-049857.R2
Article Type:	Original research
Date Submitted by the Author:	22-Jun-2021
Complete List of Authors:	BROOKS, SAMANTHA; King's College London, Department of Psychological Medicine<br>Greenberg , N; King's College London, Department of Psychological Medicine<br>Wessely, Simon; King's College London, Department of Psychological Medicine<br>Rubin, GJ; King's College London, Department of Psychological Medicine
**Primary Subject Heading**:	Infectious diseases
Secondary Subject Heading:	Occupational and environmental medicine
Keywords:	COVID-19, Public health < INFECTIOUS DISEASES, Infection control < INFECTIOUS DISEASES

SCHOLARONE™
Manuscripts

I, the Submitting Author has the right to grant and does grant on behalf of all authors of the Work (as defined in the below author licence), an exclusive licence and/or a non-exclusive licence for contributions from authors who are: i) UK Crown employees; ii) where BMJ has agreed a CC-BY licence shall apply, and/or iii) in accordance with the terms applicable for US Federal Government officers or employees acting as part of their official duties; on a worldwide, perpetual, irrevocable, royalty-free basis to BMJ Publishing Group Ltd ("BMJ") its licensees and where the relevant Journal is co-owned by BMJ to the co-owners of the Journal, to publish the Work in this journal and any other BMJ products and to exploit all rights, as set out in our licence.

The Submitting Author accepts and understands that any supply made under these terms is made by BMJ to the Submitting Author unless you are acting as an employee on behalf of your employer or a postgraduate student of an affiliated institution which is paying any applicable article publishing charge ("APC") for Open Access articles. Where the Submitting Author wishes to make the Work available on an Open Access basis (and intends to pay the relevant APC), the terms of reuse of such Open Access shall be governed by a Creative Commons licence – details of these licences and which Creative Commons licence will apply to this Work are set out in our licence referred to above.

Other than as permitted in any relevant BMJ Author's Self Archiving Policies, I confirm this Work has not been accepted for publication elsewhere, is not being considered for publication elsewhere and does not duplicate material already published. I confirm all authors consent to publication of this Work and authorise the granting of this licence.

For peer review only

Factors affecting healthcare workers' compliance with social and behavioural infection control measures during emerging infectious disease outbreaks: Rapid evidence review.

Dr Samantha K. Brooks PhD[1], Professor Neil Greenberg FRCPsych[1], Professor Sir Simon Wessely FMedSci[1], Dr G. James Rubin PhD[1]

[1]King's College London, Department of Psychological Medicine, Cutcombe Road, London SE5 9RJ, United Kingdom

Corresponding author: Dr S.K. Brooks, King's College London, Department of Psychological Medicine, Cutcombe Road, London SE5 9RJ, United Kingdom. Email: samantha.k.brooks@kcl.ac.uk Telephone: 020 7848 0432

Keywords Compliance, COVID-19, healthcare workers, infection control, public health

Word count 4,281

For peer review only

Abstract

Objective The 2019-2020 outbreak of novel coronavirus has raised concerns about nosocomial transmission. This review's aim was to explore the existing literature on emerging infectious disease outbreaks to identify factors associated with compliance with infection control measures among healthcare staff.

Methods A rapid evidence review for primary studies relevant to healthcare workers' compliance with infection control measures.

Results Fifty-six papers were reviewed. Staff working in emergency or intensive care settings or with contact with confirmed cases appeared more likely to comply with recommendations. There was some evidence anxiety and concern about the risk of infection were more associated with compliance, and that monitoring from superiors could improve compliance. Observed non-compliance of colleagues could hinder compliance. Staff identified many barriers to compliance related to personal protective equipment, including availability; perceived difficulty and effectiveness; inconvenience; discomfort; and a negative impact on patient care. There were many issues regarding the communication and ease of understanding of infection control guidance.

Conclusion We recommend provision of training and education tailored for different occupational roles within the healthcare setting; managerial staff 'leading by example'; ensuring adequate resources for infection control; and timely provision of practical evidence-based infection control guidelines.

For peer review only

For peer review only - http://bmjopen.bmj.com/site/about/guidelines.xhtml

Strengths and limitations of this study

- This review synthesises existing literature on healthcare workers' compliance with infection control measures during infectious disease outbreaks, providing an overview of what has already been established and identifying gaps in the literature.
- The search strategy was broad, and reference lists of included papers were hand-searched for any additional relevant papers which may have been missed.
- The search was limited to English-language papers due to the rapid nature of the review.
- Included papers did not undergo formal quality appraisal, again due to the rapid nature of the review.

Introduction

Recent years have seen frequent outbreaks of emerging infectious diseases.[1] Examples include severe acute respiratory syndrome (SARS) in 2003, avian influenza (H5N1) in 2004, swine flu (H1N1) in 2009, Middle East respiratory syndrome (MERS) in 2015 and most recently coronavirus SARS-CoV-2 originating in 2019. Previous outbreaks saw high levels of nosocomial transmission (hospital-acquired infections).[2]

A major cause of nosocomial transmission is poor compliance with personal protective behaviours among healthcare staff.[3 4] Early reports from the COVID-19 frontline have noted poor compliance of healthcare workers with recommended behaviours: in one hospital in China, many had their masks hung from one ear or pulled downwards, while more than half had inadequate hand hygiene.[5]

Compliance with infection control behaviours can be difficult. Previous literature has reported on difficulties in the general population with adhering to protective behaviours such as facemask-wearing,[6] using hand sanitising gel[7] and quarantine[8] during infectious disease outbreaks. The main facilitators of compliance in the general population appear to be perceived susceptibility, perceived severity of being afflicted and perceived benefits of

For peer review only - http://bmjopen.bmj.com/site/about/guidelines.xhtml

compliance as well as accurate knowledge about the disease and the recommended behaviours, while major barriers include discomfort, embarrassment and practical issues.[6 8] A review[9] of healthcare workers' perceptions of barriers and facilitators to compliance with guidelines during respiratory outbreaks suggested that protective practices are influenced by understanding of guidelines, support received from managers, communication about guidelines, sufficient resources, perceived value of following guidance, comfort of personal protective equipment (PPE), perceived impact of PPE on patients, and workplace culture. However, this review focused only on qualitative literature, the majority of which related to tuberculosis.

We systematically reviewed existing literature on compliance with social and behavioural protective behaviours among staff involved in healthcare, specifically during outbreaks of emerging infectious diseases and encompassing quantitative and qualitative research.

For peer review only

For peer review only - http://bmjopen.bmj.com/site/about/guidelines.xhtml

Methods

This rapid evidence review was carried out according to World Health Organization guidelines:[10] the basic principles of a systematic literature review were followed, with certain aspects simplified in order to produce evidence rapidly at a time when urgent evidence synthesis is required. Searching of grey literature and quality appraisal of included studies were not carried out.

Search strategy

The search strategy consisted of four search strings (adherence terms; protective behaviour terms; emerging infectious disease terms; and healthcare worker terms). The full search strategy can be seen in Appendix I. Five databases were searched from date of inception to May 4th, 2020: MEDLINE®, PsycINFO®, Embase, Global Health, and Web of Science.

Selection criteria

To be included, studies had to: i) contain primary data; ii) be published in peer-reviewed journals in English; iii) include participants who worked in healthcare; iv) include data on factors predicting adherence to social or behavioural infection control practices during emerging infectious disease pandemics. For the latter criterion, quantitative data needed to report statistics on factors associated with compliance, while the qualitative component of the review considered studies reporting on participants' beliefs about facilitators and barriers to compliance as well as any reported difficulties in complying with protective behaviours.

Screening

One author (SKB) ran the searches on all databases on May 4th, 2020. Resulting citations were downloaded to EndNote© version X9 (Thomson Reuters, New York, USA). The same author evaluated titles for relevance, then used the inclusion criteria to screen abstracts and then full texts of remaining citations and exclude any which were irrelevant. Any queries or uncertainties about inclusion were discussed with the wider research team. Reference lists of all remaining papers were hand-searched for additional relevant studies.

Data extraction and synthesis

SKB extracted the following data from papers: authors, publication year, country of study, design, participants (including *n,* demographic information and profession), disease outbreak,

For peer review only - http://bmjopen.bmj.com/site/about/guidelines.xhtml

protective behaviours measured, measures used, and key results. We used thematic analysis[11] to synthesise the data and group results into themes.

Patient and public involvement

No patients were involved in this review study.

Results

The initial search strategy yielded 1900 papers, of which 744 duplicates were removed and 1090 were excluded based on title or abstract. An additional twelve papers were found via hand-searching the reference lists of included papers. After full-text screening, 56 papers remained for inclusion. A PRISMA flow diagram of the process can be seen in Appendix II.

Countries represented in the literature included Canada (n=13), Saudi Arabia (n=7), Singapore (n=7), China (n=5), South Korea (n=5), USA (n=4), Netherlands (n=2), Australia (n=1), Greece (n=1), India (n=1), Iran (n=1), Taiwan (n=1), Turkey (n=1), UK (n=1) and Vietnam (n=1). A further five papers included participants from multiple countries. Papers discussed H1N1 (n=21), SARS (n=20), MERS (n=11), avian influenza (n=2) and COVID-19 (n=2). The participants represented in the literature were from a wide range of roles and departments in the healthcare profession, and a wide range of protective behaviours were considered. A full overview of study characteristics is presented in Supplementary Table I.

Eight main themes were identified. These were socio-demographics and personal characteristics; occupational role; training and knowledge; work-related factors; personal protective behaviour-related factors; guidance; distress and risk perception; and attitudes and behaviours of others. Supplementary Table II provides a summary of themes and sub-themes and identifies for each theme which papers showed a significant association with protective behaviours, which papers found no significant association, and which papers supplemented these findings by reporting on the theme but without statistical analysis (e.g. qualitative papers and papers with descriptive statistics only). Supplementary Table III summarises the evidence extracted from the literature for each theme. A number of other potential predictors of compliance were considered but only appeared in one paper each; these are presented in Supplementary Tables I and III but not covered in the text.

For peer review only

Socio-demographics and personal characteristics

Overall, there appeared to be no significant association between age[12-18] or gender[12-14 17 19-21] and protective behaviour. Only one study[20] found that older age was significantly associated with protective behaviours, while two found that female staff were significantly more likely to comply with protective behaviours.[16 22]

One study examined nationality as a predictor[12] and found that Saudi staff were significantly more likely to comply with protective behaviours than non-Saudi staff working in the same city. Mixed findings were reported in the studies comparing behaviours across countries. Staff in Hong Kong and Singapore were more likely to comply than UK staff[23] whereas Koh et al.[24] and Wong et al.[25] found that staff in Singapore vs Indonesia or Hong Kong vs Canada respectively were more likely to comply with some recommendations but not others (see Supplementary Table III for details). A worldwide study[26] found no significant differences between countries in terms of taking protective measures.

There was no evidence of association between compliance and religion[20] or marital status.[18 19] One paper found that staff in 'high or middle' socioeconomic status were more likely to comply than those of lower socioeconomic status.[22] Four studies found that level of education was not associated with compliance[13 18 20 21] while one found a significant association between compliance and qualification[12] (see Table III for details). One study found that H1N1 influenza vaccination was significantly associated with high compliance.[27] Having a chronic illness, being pregnant, or having a pregnant spouse, elderly person or school-aged child at home were not associated with compliance, but staff with babies at home were more likely to comply with protective behaviours.[19]

Occupational role

Many studies which examined role as a predictor of compliance found a significant relationship;[12 19 23 28-36] however, due to the variety of different roles compared across studies, it was not possible to identify an exact pattern (see Supplementary Table III for details). Five studies found no significant association between role and compliance.[13 16 17 37 38]

Length of time in role was not significantly associated with compliance in five studies[12 16-18 30] while two studies suggested that longer experience of working in healthcare was associated

For peer review only - http://bmjopen.bmj.com/site/about/guidelines.xhtml

with greater compliance[31 33] and one suggested that less than ten years' experience was associated with significantly higher compliance than more experience.[36]

Training and knowledge

There was mixed evidence on the effectiveness of outbreak-specific training and education: Taghrir et al.[21] found no significant association between protective behaviours and having received education; Nour et al.[17] found a non-significant increase in protective practices post-training; and Shigayeva et al.[35] found that recent infection control training was a significant predictor of compliance with recommended behaviours. Jeong et al.[22] found that staff who sought information about the outbreak and infection control were more likely to comply with recommended behaviours. Qualitative evidence suggested that staff felt their prior training and education were not useful in dealing with the rapidly-changing nature of emerging infectious disease outbreaks.[39 40] Participants themselves believed that inadequate training was a barrier to compliance[28] and that infection control training with annual refresher courses would benefit them.[41]

Sources of knowledge about the outbreak and protective behaviours were not associated with protective behaviours in two studies[20 21] while knowledge from textbooks and attending Continuing Medical Education activities were significantly associated with higher levels of protective practice in one study.[12] Receiving outbreak-specific training was not significantly associated with compliance, but higher outbreak-related knowledge did result in significantly higher compliance.[20] Another study examining knowledge of the outbreak itself[28] found that the majority of participants believed lack of knowledge about mode of transmission contributed to poor compliance. Knowledge of current recommendations was associated with compliance in three studies;[4 16 27] associated with compliance in one hospital but not three other hospitals in another study;[14] and was not associated with compliance in one further study.[12] Hsu et al.[42] found that a minority of participants believed that lack of education explained lack of compliance.

Work-related factors

Compliance appeared to be higher in higher acuity settings such as emergency, intensive care or inpatient departments.[14 35 38 43 44] Wong et al.[45] found that staff in high-infection districts were more likely to wear gowns, wash hands and use disinfectants but less likely to comply

For peer review only - http://bmjopen.bmj.com/site/about/guidelines.xhtml

with quarantine measures. Two studies found no significant association between setting and compliance.[19 21]

Having contact with confirmed cases was associated with higher compliance in three studies[14 35 43] whereas one study[19] found no association. A further study[18] found higher compliance in staff who worked directly with confirmed cases. Wong et al.[45] found that SARS-exposed staff were more likely than non-SARS-exposed staff to comply with mask guidance, but less likely to quarantine themselves.

There was some evidence that high workload may be a barrier to compliance with recommended personal protective behaviours,[23 30 35 36 39 41 46] although one study also suggested that higher workload (in terms of working overtime) was associated with increased compliance in terms of giving patients appropriate infection control advice.[36]

Two studies suggested that monitoring of compliance by superiors or public health authorities encouraged compliance,[27 47] while a small minority of participants in Hsu et al.'s[42] study reported that better policing by infection control staff was the most important strategy for improving compliance.

Two studies suggested compliance was associated with characteristics of the patient encounter. Many of De Perio et al.'s[14] participants reported not using recommended PPE if they did not know the patient had H1N1 or an influenza-like illness, if they did not think it was needed for the particular activity they were doing, if they only entered the patient's room for a brief time, if they did not touch the patient or if they did not come within six feet of the patient. Meanwhile, Shigayeva et al.'s[35] participants were less likely to comply with recommended behaviours when providing care for patients with more severe illness (which the authors suggest may be due to the time required to don barrier equipment leading staff to put patient safety above self-protection) and if they were only observing procedures, rather than performing or assisting with them.

Personal protective behaviour-related factors

Many studies, although mostly without statistical analysis, reported issues in performing personal protective behaviours, most notably due to lack of availability of appropriate PPE,

For peer review only

perceived difficulty of protective behaviours, logistic issues, perceived effectiveness, perceived importance, convenience, comfort, and the impact on patient care.

Participants in several studies raised concerns about lack of PPE,[14 39-42 48-53] mostly due to insufficient resources. Shortage of PPE created difficulties such as having to wear the wrong size PPE.[40 41 51] One study showed that availability of PPE was significantly associated with higher compliance[27] while another found that staff were significantly more compliant with PPE use when eyewear and gloves were readily available at the point of care, but availability did not increase compliance of N95 respirators, surgical masks or gowns.[33] Logistic issues were also reported, such as lack of space in the hospital, making it difficult to use PPE appropriately.[51 54 55]

There may also be issues regarding the perceived difficulty of protective behaviours. Kang et al.'s[54] participants reported difficulties with the complexity of using several PPE items together, and only 35% of Van Dijk et al.'s[53] participants thought the recommended measures were feasible.

Perceived effectiveness of PPE significantly predicted higher compliance in three studies.[27 33 34] In one qualitative study, staff expressed doubts about the quality and effectiveness of PPE.[54] Beliefs in the effectiveness of infection control procedures, as modified by previous experiences, were identified by participants as having a positive impact on compliance.[46]

Hsu et al.[42] reported that failure to recognise the importance of hand hygiene prevented compliance, while Vinck et al.[36] found that the main reason for not complying with the recommendation to consult with a specialist unit for centralised assessment of symptomatic patients was associated with finding it 'unnecessary'.

Participants in many qualitative studies reported not using PPE due to perceived inconvenience and its effect on their ability to do their job[14 27 39-42 54 56 57] – in particular, it appeared that participants found it too time-consuming to change in and out of protective clothing as it slowed them down substantially.

Many of Khalid et al.'s[49] participants reported that having to use PPE was 'stressful'. Many qualitative studies found that discomfort of PPE was reported to be a barrier to compliance.[39-

For peer review only - http://bmjopen.bmj.com/site/about/guidelines.xhtml

[41 46 52 56-58] In several studies, participants reported physical harm due to PPE, including dehydration and skin peeling,[41] difficulty breathing,[40 52 58 59] sweating and dizziness,[58] headaches[40 52 60] and skin rashes.[40 52] Comfort of PPE was examined as a potential predictor of compliance in one quantitative study[33] which found that staff who reported always or often feeling comfortable wearing protective eyewear and N95 respirators were significantly more likely to wear them; however, no significant association was found in compliance between staff who reported always or often feeling short of breath, claustrophobic or dizzy when wearing protective eyewear or N95 respirators and those who rarely experienced these symptoms.

For peer review only

Qualitative data also revealed that many healthcare professionals believe that PPE use has an impact on patient care, making it difficult to communicate with patients due to muffled speech,[40 41 52 56 57 59] being unable to establish non-verbal cues with patients[60] and making them less 'visible' to their patients.[61] Participants in Rowlands' study[52] also reported concern that masks could be frightening for psychiatric patients, and Tan et al.'s[40] participants reported that PPE created anxiety in some patients who assumed they were wearing it as they had been exposed to the virus. One quantitative study found that the perception that PPE use would interfere with patient care was significantly associated poor compliance.[27]

Guidance

Participants in many studies reported a lack of guidance on the protocols for caring for infected patients and protecting themselves.[28 30 39 40 41 46 50 51 53-56 58 61-65]

In particular, guidelines were often found to be too long, providing staff with 'information overload'.[30 50 63] The recommended protocols were also reported to change too frequently for staff to keep up[39 41 54-56 58 61 63 64] and staff received conflicting messages from different sources.[39 41 50 51 62-64] Other issues included inconsistent advice about what to do when patients are deemed non-infectious,[41] difficulty working out what should be prioritised[50] and external guidance with little relevance to specific locations.[39] The manner of communication of guidance could also be an issue, with Moore et al.'s[39] participants reporting that new guidelines were sent to them by email, which they did not check before work; Rambaldini et al.'s[64] participants reporting frustration that information was filtered down from other sources rather than given to them first-hand; and Nhan et al.'s[63] participants suggesting that communication about changes to protocols was too slow.

For peer review only - http://bmjopen.bmj.com/site/about/guidelines.xhtml

Distress and risk perception

High levels of distress were associated with higher compliance in two studies: Chia et al.[43] found that higher IES scores were associated with higher use of respiratory protection while Wong et al.[45] found that staff who were highly anxious were more likely to comply with recommended protective behaviours. However, when the protective measure involved quarantine, some staff felt such high stress that they were tempted to break guidelines[47] (it is unclear from the paper what percentage of these actually did break the guidelines, if any).

Risk perception appeared to be associated with compliance. Four studies found that compliance was significantly more likely in staff who perceived the disease to be a serious risk[20 22 34 66] and another found that perceived seriousness of the outbreak was significantly associated with compliance in Hong Kong but not in Singapore or the UK.[23] Three qualitative papers also suggested that participants themselves believed staff complied with recommended behaviours when they perceived the risk to be severe.[39 47 51] However, one study[21] showed a significant negative correlation between protective behaviours and fear of infection.

Attitudes and behaviours of others

Qualitative data revealed that many participants had observed non-compliance in colleagues or managers[27 39] which could lead to non-compliance in the participants themselves.[30,42] DiGiovanni et al.'s[47] participants suggested that managers had modified the recommended quarantine guidelines to allow their staff to return in response to critical staff shortages. Yassi et al.'s[46] participants believed that compliance of other occupational groups within the healthcare setting had an impact on compliance, while more than half of Hsu et al.'s[42] participants believed that senior staff 'leading by example' and complying with recommended behaviours was the single most important strategy in improving staff compliance.

Attitudes of family members were also deemed important in two qualitative studies[39 46] with healthcare staff reporting they were encouraged to comply by anxious family members who were afraid of getting infected.

Discussion

For peer review only - http://bmjopen.bmj.com/site/about/guidelines.xhtml

Overall, the studies reviewed provided mixed and sometimes contradictory results. Nonetheless, some risk factors which stood out as being the most promising either for identifying specific groups at-risk for poor compliance or for targeting in interventions. These included: working outside of emergency or intensive care settings; not working with confirmed infection cases; lack of concern about risk of infection; lack of monitoring by superiors; observed non-compliance of colleagues; lack of PPE; perceived difficulty using PPE; perceived lack of effectiveness or lack of importance of PPE; perceived inconvenience and discomfort of PPE; perceived negative impact of PPE on patient care; lack of infection control guidance; and inconsistent or unclear guidance. Organisations faced with nosocomial transmission would be wise, as part of their mitigation efforts, to look to these areas in an effort to help staff adhere to protective behaviours.

There was little evidence for an association between compliance with protective behaviours and socio-demographic or personal characteristics. There was some evidence that compliance levels differ across countries: it is not clear whether this is due to between-country differences in the communication of guidance, different risk perceptions due to different countries' media coverage of the outbreak, different levels of training received, or cultural differences in the participants themselves.

This review suggests that compliance may differ between different roles and different settings. Targeted interventions for specific occupational groups with different levels of patient contact, hierarchies and cultures, may be helpful. Whilst it was unclear from the varied data which occupational groups might need more attention, it seems that those not in emergency departments or intensive care units and not working directly with infected patients could benefit from additional focus.

We found little evidence that training and education significantly improved compliance. This initially seems surprising as previous trials of training courses to improve knowledge of infection control practices and encourage compliance with them have suggested positive results.[67-69] However, taken together with the evidence that compliance differs between occupational groups, it may be that staff in different roles require different levels and methods of training. We also found mixed evidence of the impact of knowledge about infection control on compliance with the recommended measures: this may be associated with what is actually covered in training. For example, as it appears that the perceived

For peer review only - http://bmjopen.bmj.com/site/about/guidelines.xhtml

importance of PPE is related to compliance, it may be that training that covers the 'why' rather than the 'how' of PPE is particularly useful. However, training itself should not be viewed as a panacea. Many of the factors identified by our review, and discussed below, are not amenable to better training.

A small amount of evidence suggested that observed non-compliance by others – including colleagues - could affect healthcare workers' own levels of compliance. This is likely to be particularly problematic where managers and supervisors fail to 'lead by example' and ensure that they comply with the recommended policies and procedures. 'Role modelling' by superiors could be useful, with supervisors setting the standards for infection control practices and reinforcing them.

The availability, ease of use, perceived effectiveness, convenience, comfort, and impact on patients of PPE may be other key factors which need to be addressed. This review suggests fairly negative views of PPE, with participants reporting lack of appropriate resources, the inconvenience of having to change in and out of PPE repeatedly, physical discomfort, and a negative impact on their ability to communicate with patients. It is useful to know about these concerns about PPE as this can be a target to change.

Reviewed studies suggest difficulties understanding and keeping up with rapidly changing recommendations. This may be unavoidable during an outbreak of an emerging infectious disease, as new evidence becomes available. Staff should therefore be prepared for the likelihood of changes, both to help maintain trust and to ensure that they monitor for updates.

There was some evidence that higher levels of anxiety and risk perception were associated with higher compliance. This may suggest that, whilst it is important not to create unhealthy anxiety, desensitisation to risk or genuine reductions in risk may lead to a reduction in PPE use. Indeed, it has been reported that staff in industrial settings experience complacency in terms of protective behaviours, as a result of habituation (repeated exposures which do not result in anything bad, lessening fearful responding) and low levels of positive reinforcement for safe behaviour.[70] Particular care may be needed to maintain PPE use as risk changes, or is perceived to change, over the course of an outbreak.

Limitations

For peer review only - http://bmjopen.bmj.com/site/about/guidelines.xhtml

We are aware that many papers (particularly on SARS and MERS) have been published in other (predominantly Asian) languages, but due to the rapid nature of this review we limited inclusion to English-language papers only. Future reviews should consider translating and analysing the many relevant foreign-language papers. Also due to the rapid nature of the review, quality appraisal of individual papers was not carried out as this is not always deemed necessary when urgent evidence synthesis is required.[71] Our searches were carried out in May 2020 in order to provide an early look at the factors associated with nosocomial transmission at the start of the COVID-19 pandemic, and we are aware that new relevant studies have been published since this date. It would be useful for future reviews to consider the newer literature on COVID-19 and compare the results to our own. We also note that how adherence changes over the course of a long outbreak that is characterised by periods of high and low transmission is not clear – the studies we included did not address this, as most focused on relatively short outbreaks and the few COVID-19 papers that were included were from the early days of the pandemic. It is plausible that the factors driving behaviour change as time goes on, and more research is needed.

Conclusion

Interventions that may be helpful for improving healthcare workers' compliance include role-specific or setting-specific training; emphasising the importance of protective behaviours and the risk of infection if behaviours are not performed; monitoring of staff behaviours by supervisors and positive reinforcement for correct behaviours; managerial staff leading by example; training focused on the importance and effectiveness of PPE; and better communication of guidelines. The results of this review also suggest that the following interventions would likely *not* be useful: training aimed at specific age or gender groups; training focused on increasing knowledge about the outbreak itself; or training on how to use PPE without also emphasising *why* it is necessary.

Contributorship statement

GJR conceived the work. SKB conducted the literature search and screened search results for inclusion, and GJR, NG and SW resolved any queries about inclusion. All authors contributed to the qualitative results synthesis, prepared the first draft of the manuscript and approved the final manuscript.

Competing interests

For peer review only - http://bmjopen.bmj.com/site/about/guidelines.xhtml

None declared.

Funding

The research was funded by the National Institute for Health Research Health Protection Research Unit (NIHR HPRU) in Emergency Preparedness and Response at King's College London in partnership with Public Health England (PHE), in collaboration with the University of East Anglia (funding reference: NIHR200890). The views expressed are those of the author(s) and not necessarily those of the NHS, the NIHR, the Department of Health and Social Care, or Public Health England. The funder had no involvement in the study design, collection, analysis or interpretation of data, writing the report, or decision to submit for publication.

Data sharing statement

No original data were generated for this study.

Ethics

Not needed - no original data generated / no participants.

Executive license

I, the Submitting Author has the right to grant and does grant on behalf of all authors of the Work (as defined in the below author licence), an exclusive licence and/or a non-exclusive licence for contributions from authors who are: i) UK Crown employees; ii) where BMJ has agreed a CC-BY licence shall apply, and/or iii) in accordance with the terms applicable for US Federal Government officers or employees acting as part of their official duties; on a worldwide, perpetual, irrevocable, royalty-free basis to BMJ Publishing Group Ltd ("BMJ") its licensees and where the relevant Journal is co-owned by BMJ to the co-owners of the Journal, to publish the Work in BMJ Open and any other BMJ products and to exploit all rights, as set out in our licence.

The Submitting Author accepts and understands that any supply made under these terms is made by BMJ to the Submitting Author unless you are acting as an employee on behalf of your employer or a postgraduate student of an affiliated institution which is paying any applicable article publishing charge ("APC") for Open Access articles. Where the Submitting Author wishes to make the Work available on an Open Access basis (and intends to pay the

For peer review only - http://bmjopen.bmj.com/site/about/guidelines.xhtml

relevant APC), the terms of reuse of such Open Access shall be governed by a Creative Commons licence – details of these licences and which Creative Commons licence will apply to this Work are set out in our licence referred to above.

References

1. World Health Organization, Regional Office for South-East Asia. (2014). A brief guide to emerging infectious diseases and zoonoses. WHO Regional Office for South-East Asia. https://apps.who.int/iris/handle/10665/204722 [accessed 09 May 2020]

2. Feng D, de Vlas SJ, Fang L-Q, Han XN, Zhao W-J, Sheng S, et al. The SARS epidemic in mainland China: bringing together all epidemiological data. Trop Med Int Health. 2009;14(Suppl 1):4-13.

3. Gamage B, Moore D, Copes R, Yassi A, Bryce E, members of The BC Interdisciplinary Respiratory Protection Study Group Vancouver, British Columbia, Canada. Protecting health care workers from SARS and other respiratory pathogens: A review of the infection control literature. Am J Infect Control. 2005;33(2),114-21.

4. Yap J, Lee VJ, Yau TY, Ng TP, Tor PC. Knowledge, attitudes and practices towards pandemic influenza among cases, close contacts, and healthcare workers in tropical Singapore: a cross-sectional survey. BMC Public Health. 2010;10:442.

5. Xu C, Jin J, Song J, Yang Y, Yao M, Zhang Y, et al. Application of refined management in prevention and control of the coronavirus disease 2019 epidemic in non-isolated areas of a general hospital. Int J Nurs Sci. 2020;7(2):143-7.

6. Sim SW, Moey KSP, Tan NC. The use of facemasks to prevent respiratory infection: a literature review in the context of the Health Belief Model. Singapore Med J. 2014;55(3):160-7.

For peer review only

7. Rubin GJ, Potts HWW, Michie S. The impact of communications about swine flu (influenza A H1N1v) on public responses to the outbreak: results from 36 national telephone surveys in the UK. Health Technol Assess. 2010;14(34):183-266.

8. Webster RK, Brooks SK, Smith LE, Woodland L, Wessely S, Rubin GJ. How to improve adherence with quarantine: rapid review of the evidence. Public Health. 2020;182:163-9.

9. Houghton C, Meskell P, Delaney H, Smalle M, Glenton C, Booth A, et al. Barriers and facilitators to healthcare workers' adherence with infection prevention and control (IPC) guidelines for respiratory infectious diseases: a rapid qualitative evidence synthesis. Cochrane Database of Systematic Reviews. 2020;4.

10. World Health Organization. (2017). Rapid reviews to strengthen health policy and systems: a practical guide. https://www.who.int/alliance- hpsr/resources/publications/rapid-review-guide/en/ [accessed 04 May 2020]

11. Braun V, Clarke V. Using thematic analysis in psychology. Qual Res Psychol. 2006;3(2):77-101.

12. Al-Amri S, Bharti R, Alsaleem SA, Al-Musa HM, Chaudhary S, Al-Shaikh AA. Knowledge and practices of primary health care physicians regarding updated guidelines of MERS-CoV infection in Abha city. J Family Med Prim Care. 2019;8(2):455-61.

13. Datta SS, Kuppuraman D, Boratne AV, Abraham SB, Singh Z. Knowledge, attitude and practices regarding swine flu among para-medical workers in a tertiary care hospital in Pondicherry. J Commun Dis. 2011;43(1):1-9.

14. de Perio MA, Brueck SE, Mueller CA, Milne CK, Rubin MA, Gundlapalli AV, et al. Evaluation of 2009 pandemic influenza A (H1N1) exposures and illness among physicians in training. Am J Infect Control. 2012;40(7):617-21.

15. Manabe T, Pham Thi Phuong T, Kudo K, Vu Thi Tuong V, Takasaki J, Nguyen Dang T, et al. Impact of education and network for Avian Influenza H5N1 in humans: Knowledge, clinical practice, and motivation on medical providers in Vietnam. Plos One. 2012;7(1).

For peer review only

For peer review only - http://bmjopen.bmj.com/site/about/guidelines.xhtml

16. Nour MO, Babalghith AO, Natto HA, Al-Amin FO, Alawneh SM. Knowledge, attitude and practices of healthcare providers towards MERS-CoV infection at Makkah hospitals, KSA. Int Res J Med Med Sci. 2015;3(4): 103-12.

17. Nour MO, Babalghith AO, Natto HA, Alawneh SM, Elamin FO. Raising awareness of health care providers about MERS-CoV infection in public hospitals in Mecca, Saudi Arabia. East Mediterr Health J. 2017;23(8):534-42.

18. Pratt M, Kerr M, Wong C. The impact of ERI, burnout, and caring for SARS patients on hospital nurses' self-reported compliance with infection control. CJIC. 2009;24(3):167-74.

19. Evirgen O, Savas N, Koksaldi Motor V, Onlen Y, Yengil E. An evaluation of knowledge, attitudes, and behaviors of employees of a university hospital in an H1N1 influenza pandemic. J Infect Dev Ctries. 2014;8(5):561-9.

20. Kim JS, Choi JS. Middle East respiratory syndrome-related knowledge, preventive behaviours and risk perception among nursing students during outbreak. J Clin Nurs. 2016;25(17-18):2542-9.

21. Taghrir MH, Borazjani R, Shiraly R. COVID-19 and Iranian medical students; A survey on their related-knowledge, preventive behaviors and risk perception. Arch Iran Med. 2020;23(4):249-54.

22. Jeong HS, Lee DW, Youn CH, Lee MK, Lee SJ, Suh YS, et al. Perception and performance of preventive behaviors for the pandemic influenza in hospital employees and outpatients. Yonsei Med J. 2011;52(1):181-7.

23. Chor JSY, Pada SK, Stephenson I, Goggins WB, Tambyah PA, Medina M, et al. Differences in the compliance with hospital infection control practices during the 2009 influenza H1N1 pandemic in three countries. J Hosp Infect. 2012;81(2):98-103.

24. Koh GC-H, Abikusno N, Kwing CS, Yee WT, Kusumaratna R, Sundram M, et al. Avian influenza and South Jakarta primary healthcare workers: a controlled mixed-method study. Trop Med Int Health. 2009;14(7):817-29.

For peer review only

For peer review only - http://bmjopen.bmj.com/site/about/guidelines.xhtml

25. Wong SYS, Wong W, Jaakkimainen L, Bondy S, Tsang KK, Lee A. Primary care physicians in Hong Kong and Canada - how did their practices differ during the SARS epidemic? Fam Pract. 2005;22(4):361-6.

26. Kamate SK, Sharma S, Thakar S, Srivastava D, Sengupta K, Hadi AJ, et al. Assessing knowledge, attitudes and practices of dental practitioners regarding the covid-19 pandemic: A multinational study. Dent Med Probl. 2020;57(1):11-7.

27. Hu X, Zhang Z, Li N, Liu D, He W, Zhang W, et al. Self-reported use of Personal Protective Equipment among Chinese Critical Care Clinicians during 2009 H1N1 influenza pandemic. PLoS ONE. 2012;7(9).

28. Alsahafi AJ, Cheng AC. Knowledge, attitudes and behaviours of healthcare workers in the Kingdom of Saudi Arabia to MERS Coronavirus and other emerging infectious diseases. Int J Environ Res Public Health. 2016;13(12).

29. Alsubaie S, Hani Temsah M, Al-Eyadhy AA, Gossady I, Hasan GM, Al-Rabiaah A, et al. Middle East Respiratory Syndrome Coronavirus epidemic impact on healthcare workers' risk perceptions, work and personal lives. J Infect Dev Ctries. 2019;13(10):920-6.

30. Chau JP, Thompson DR, Twinn S, Lee DT, Lopez V, Ho LS. An evaluation of SARS and droplet infection control practices in acute and rehabilitation hospitals in Hong Kong. Hong Kong Med J. 2008;14 Suppl 4:44-7.

31. Koh D, Lim MK, Chia SE, Ko SM, Qian F, Ng V, et al. Risk perception and impact of severe acute respiratory syndrome (SARS) on work and personal lives of healthcare workers in Singapore: What can we learn? Med Care. 2005;43(7):676-82.

32. May L, Katz R, Johnston L, Sanza M, Petinaux B. Assessing physicians' in training attitudes and behaviors during the 2009 H1N1 influenza season: a cross-sectional survey of medical students and residents in an urban academic setting. Influenza Other Respir Viruses. 2010;4(5):267-75.

For peer review only

33. Mitchell R, Ogunremi T, Astrakianakis G, Bryce E, Gervais R, Gravel D, et al. Impact of the 2009 influenza A (H1N1) pandemic on Canadian health care workers: A survey on vaccination, illness, absenteeism, and personal protective equipment. Am J Infect Control. 2012;40(7):611-6.

34. Parker MJ, Goldman RD. Paediatric emergency department staff perceptions of infection control measures against severe acute respiratory syndrome. EMJ. 2006;23(5):349-53.

35. Shigayeva A, Green K, Raboud JM, Henry B, Simor AE, Vearncombe M, et al. Factors associated with critical-care healthcare workers' adherence to recommended barrier precautions during the Toronto severe acute respiratory syndrome outbreak. Infect Control Hosp Epidemiol. 2007;28(11):1275-83.

36. Vinck L, Isken L, Hooiveld M, Trompenaars M, Ijzermans J, Timen A. Impact of the 2009 influenza A(H1N1) pandemic on public health workers in the Netherlands. Euro Surveill. 2011;16(7).

37. Alshammari M, Reynolds KA, Verhougstraete M, O'Rourke MK. Comparison of perceived and observed hand hygiene compliance in healthcare workers in MERS-CoV endemic regions. Healthcare (Basel). 2018;6(4).

38. Jaeger JL, Patel M, Dharan N, Hancock K, Meites E, Mattson C, et al. Transmission of 2009 Pandemic Influenza A (H1N1) Virus among healthcare personnel-Southern California, 2009. Infect Control Hosp Epidemiol. 2011;32(12):1149-57.

39. Moore DM, Gilbert M, Saunders S, Bryce E, Yassi A. Occupational health and infection control practices related to severe acute respiratory syndrome: health care worker perceptions. AAOHN J. 2005;53(6):257-66.

40. Tan NC, Goh LG, Lee SS. Family physicians' experiences, behaviour, and use of personal protection equipment during the SARS outbreak in Singapore: Do they fit the Becker Health Belief Model? Asia Pac J Public Health. 2006;18(3):49-56.

For peer review only

41. Corley A, Hammond NE, Fraser JF. The experiences of health care workers employed in an Australian intensive care unit during the H1N1 influenza pandemic of 2009: A phenomenological study. Int J Nurs Stud. 2010;47(5):577-85.

42. Hsu LY, Jin J, Ang BS, Kurup A, Tambyah PA. Hand hygiene and infection control survey pre- and peri-H1N1-2009 pandemic: knowledge and perceptions of final year medical students in Singapore. Singapore Med J. 2011;52(7):486-90.

43. Chia SE, Koh D, Fones C, Qian F, Ng V, Tan BH, et al. Appropriate use of personal protective equipment among healthcare workers in public sector hospitals and primary healthcare polyclinics during the SARS outbreak in Singapore. Occup Environ Med. 2005;62(7):473-7.

44. Ki HK, Han SK, Son JS, Park SO. Risk of transmission via medical employees and importance of routine infection-prevention policy in a nosocomial outbreak of Middle East respiratory syndrome (MERS): a descriptive analysis from a tertiary care hospital in South Korea. BMC Pulm Med. 2019;19(1).

45. Wong WCW, Lee A, Tsang KK, Wong SYS. How did general practitioners protect themselves, their family, and staff during the SARS epidemic in Hong Kong? J Epidemiol Community Health. 2004;58(3):180-5.

46. Yassi A, Moore D, Fitzgerald JM, Bigelow P, Hon CY, Bryce E, et al. Research gaps in protecting healthcare workers from SARS and other respiratory pathogens: an interdisciplinary, multi-stakeholder, evidence-based approach. J Occup Environ Med. 2005;47(1):41-50.

47. DiGiovanni C, Conley J, Chiu D, Zaborski J. Factors influencing compliance with quarantine in Toronto during the 2003 SARS outbreak. Biosecur Bioterror. 2004;2(4):265-72.

48. Edeghere O, Fowler T, Wilson F, Caspa R, Raichand S, Kara E, et al. Knowledge, attitudes, experience and behaviour of frontline health care workers during the early phase of 2009 influenza A(H1N1) pandemic, Birmingham, UK. J Health Serv Res Pol. 2015;20(1):26-30.

For peer review only - http://bmjopen.bmj.com/site/about/guidelines.xhtml

49. Khalid I, Khalid TJ, Qabajah MR, Barnard AG, Qushmaq IA. Healthcare workers' emotions, perceived stressors and coping strategies during a MERS-CoV outbreak. Clin Med Res. 2016;14(1):7-14.

50. Locatelli SM, LaVela SL, Hogan TP, Kerr AN, Weaver FM. Communication and information sharing at VA facilities during the 2009 novel H1N1 influenza pandemic. Am J Infect Control. 2012;40(7):622-6.

51. Rebmann T, Wagner W. Infection preventionists' experience during the first months of the 2009 novel H1N1 influenza A pandemic. Am J Infect Control. 2009;37(10):e5-16.

52. Rowlands A. (2007). Medical social work practice and SARS in Singapore. Soc Work Health Care. 2007;45(3):57-83.

53. van Dijk CE, Hooiveld M, Jentink A, Isken LD, Timen A, Yzermans CJ. Experiences of General Practitioners and Practice Assistants during the Influenza A (H1N1) Pandemic in the Netherlands: A Cross-Sectional Survey. Plos One. 2015;10(8).

54. Kang J, Kim EJ, Choi JH, Hong HK, Han S-H, Choi IS, et al. Difficulties in using personal protective equipment: Training experiences with the 2015 outbreak of Middle East respiratory syndrome in Korea. Am J Infect Control. 2018;46(2):235-7.

55. Wong ELY, Wong SYS, Lee N, Cheung A, Griffiths, S. Healthcare workers' duty concerns of working in the isolation ward during the novel H1N1 pandemic. J Clin Nurs. 2012;21(9-10):1466-75.

56. Lam KK, Hung SYM. Perceptions of emergency nurses during the human swine influenza outbreak: A qualitative study. Int Emerg Nurs. 2013;21(4):240-6.

57. Nickell LA, Crighton EJ, Tracy CS, Al-Enazy H, Bolaji Y, Hanjrah S, et al. Psychosocial effects of SARS on hospital staff: survey of a large tertiary care institution. CMAJ. 2004;170(5):793-8.

For peer review only

58. Kang HS, Son YD, Chae S-M, Corte C. Working experiences of nurses during the Middle East respiratory syndrome outbreak. Int J Nurs Pract. 2018;24(5):e12664.

59. Khoo KL, Leng PH, Ibrahim IB, Lim TK. The changing face of healthcare worker perceptions on powered air-purifying respirators during the SARS outbreak. Respirology. 2005;10(1):107-10.

60. Straus SE, Wilson K, Rambaldini G, Rath D, Lin Y, Gold WL, et al. Severe acute respiratory syndrome and its impact on professionalism: qualitative study of physicians' behaviour during an emerging healthcare crisis. BMJ. 2004;329(7457):83.

61. Tolomiczenko GS, Kahan M, Ricci M, Strathern L, Jeney C, Patterson K, et al. SARS: Coping with the impact at a community hospital. J Adv Nurs. 2005;50(1):101-10.

62. Hsu CC, Chen T, Chang M, Chang YK. Confidence in controlling a SARS outbreak: Experiences of public health nurses in managing home quarantine measures in Taiwan. Am J Infect Control. 2006;34(4):176-81.

63. Nhan C, Laprise R, Douville-Fradet M, Macdonald ME, Quach C. Coordination and resource-related difficulties encountered by Quebec's public health specialists and infectious diseases/medical microbiologists in the management of A(H1N1) – a mixed-method, exploratory survey. BMC Public Health. 2012;12:115.

64. Rambaldini G, Wilson K, Rath D, Lin Y, Gold WL, Kapral MK, et al. The impact of severe acute respiratory syndrome on medical house staff: A qualitative study. J Gen Intern Med. 2005;20(5):381-5.

65. Robertson E, Hershenfield K, Grace SL, Stewart DE. The psychosocial effects of being quarantined following exposure to SARS: A qualitative study of Toronto health care workers. Can J Psychiatry. 2004;49(6):403-7.

66. Goulia P, Mantas C, Dimitroula D, Mantis D, Hyphantis T. General hospital staff worries, perceived sufficiency of information and associated psychological distress during the A/H1N1 influenza pandemic. BMC Infect Dis. 2010;10 (no pagination).

For peer review only

67. Carrico RM, Coty MB, Goss LK, LaJoie AS. Changing health care worker behavior in relation to respiratory disease transmission with a novel training approach that uses biosimulation. Am J Infect Control. 2007;35(1):14-9.

68. Hon C-Y, Gamage B, Bryce EA, LoChang J, Yassi A, Maultsaid D, et al. Personal protective equipment in health care: Can online infection control courses transfer knowledge and improve proper selection and use? Am J Infect Control. 2008;36(10):E33-7.

69. May L, Lung D, Harter K. An intervention to improve compliance with transmission precautions for influenza in the emergency department: Successes and challenges. J Emerg Med. 2012;42(1):79-85.

70. Hyten C, Ludwig TD. Complacency in process safety: A behaviour analysis toward prevention strategies. J Organ Behav Manage. 2017;37(3-4):240-60.

71. Haby MM, Chapman E, Clark R, Barreto J, Reveiz L, Lavis JN. What are the best methodologies for rapid reviews of the research evidence for evidence-informed decision making in health policy and practice: a rapid review. Health Res Policy Syst. 2016;14(1):83.

For peer review only

Supplementary Table I. Study characteristics

**Authors (year)**	**Country**	**Disease outbreak**	**Participants: N, age, gender**	**Professional role**	**Protective behaviours examined**
Yap et al. (2010)[4]	Singapore	H1N1	331;<br>Mean age 23.2, range 18-61;<br>7.6% female	Healthcare workers	Questions on preventive practices related to mask wearing, vaccination, avoidance behaviours, personal habits (e.g. washing hands regularly, covering mouth when coughing/sneezing), and medical attention seeking practice
Al-Amri et al. (2019)[12]	Saudi Arabia	MERS	85;<br>37.6% under 30; 49.4% aged 30-40; 12.9% aged over 40;<br>38.8% female	General practitioners (62.4%), specialists (27.1%), consultants (10.6%)	'Adherence toward guidelines' assessed through 10 statements; no further information provided
Datta et al. (2011)[13]	India	H1N1	237;<br>58.23% aged 21-25, 24.05% aged 26-30;<br>20.25% female	Tertiary care hospital staff: 113 nurses, 41 laboratory technicians, 20 auxilliary nursing midwives, 61 'other' including medical social	Frequent handwashing, use of face mask, use of gloves, closed-front gown, use of disinfectant, 'others'

For peer review only - http://bmjopen.bmj.com/site/about/guidelines.xhtml

				workers and physiotherapists	
De Perio et al. (2012)[14]	USA	H1N1	88;<br>Median age 30, range 25-53;<br>35% female	Physicians in training: 75 internal medicine residents, 7 cardiology fellows, 6 pulmonary and critical care fellows (only 53 provided sufficient information on protective behaviours)	Respiratory protection: gloves, gowns, surgical masks, fit-tested N95 respirators, powered air-purified respirators, eye protection
Manabe et al. (2012)[15]	Vietnam	Avian influenza	326;<br>Physicians: 39.8% aged 35 or under, 39.8% aged 36-50, 20.4% aged 51 or over, Nurses: 35.6% aged 35 or under, 47.5% aged 36-50, 16.9% aged 51 or over, Lab technicians: 45.5% aged 35 or under, 48.5% aged 36-50, 6.1% aged 51 or over, Other: 43.5% aged	64.7% physicians, 18.1% nurses, 10.1% laboratory technicians, 7.1% other	Wearing PPE to see suspected H5N1 patients

For peer review only - http://bmjopen.bmj.com/site/about/guidelines.xhtml

			35 or under, 47.8% aged 36-50, 8.7% aged 51 or over;<br>43.8% female (doctors), 90.0% female (nurses), 84.8% female (lab technicians), 56.5% female (other)		
Nour et al. (2015)[16]	Saudi Arabia	MERS	281;<br>Mean age 30.8, range 21-57;<br>57.7% female	46.3% nurses, 20.6% physicians, 9.6% specialists, 23.5% technicians	Using soap and water to wash hands, covering nose and mouth with a tissue when sneezing or coughing, throwing used tissues away, avoiding touching eyes/nose/mouth, using facemask in crowds, carefully handling suspected patients' belongings, healthy eating/healthy lifestyle, educating patients about disease
Nour et al. (2017)[17]	Saudi Arabia	MERS	188 of the participants in Nour et al. (2015);<br>Age and gender for this sub-group not reported	188 of the participants in Nour et al. (2015);<br>Occupational role for this sub-group not reported	As reported in Nour et al. (2015)

For peer review only - http://bmjopen.bmj.com/site/about/guidelines.xhtml

Pratt et al. (2009)[18]	Canada	SARS	536;<br>Mean age 48.4;<br>96.6% female	Hospital nurses; Primary area of practice: 26.2% medicine, surgery; 19.5% maternal newborn, paediatrics; 29.4% emergency, critical care; 24.9% other	Cleaning hands with water and soap or waterless hand rubs after removing disposable gloves, wearing disposable gloves, wearing a disposable outer garment, wearing an N95 mask, wearing protective eyewear
Evirgen et al. (2014)[19]	Turkey	H1N1	22;<br>Mean age 31.95, range 19-48;<br>59.7% female	University hospital staff: 22 professors, 29 researchers, 26 nurses; 35% from surgical departments, 32% from internal medicine departments; 17% from operation room/intensive care unit; 14% from laboratories	Total score for protective behaviours including clearing the air in the environment, washing hands with soap after practice, rubbing hands with antiseptic after practice, using disinfectant at home, H1N1 vaccination, vaccination of children, recommending vaccination to at-risk groups, using medicines supporting the immune system, using alternative medicine drugs, using antivirals for prophylactic purposes, following scientific journals about H1N1 and looking at media statements on H1N1

For peer review only - http://bmjopen.bmj.com/site/about/guidelines.xhtml

Kim & Choi (2016)[20]	South Korea	MERS	249;<br>Mean age 21.9, range 20-32;<br>88.4% female	Nursing students in third or fourth year engaged in clinical practice for 8 hours a day at tertiary hospitals with MERS patients	Nine-item scale consisting of five items about reducing the use of public places in daily life, one item about avoiding people with cough, one item about intensive cleaning and disinfection, one item about handwashing and one item about talking with people nearby
Taghrir et al. (2020)[21]	Iran	COVID-19	240;<br>Mean age 23.67;<br>59.2% female	Fifth to seventh year medical students	Preventive behaviours: cancelling social activities, not using public transport, less frequent shopping, reducing the use of closed spaces, cough etiquette, avoiding large gatherings, increased cleaning and disinfecting of surfaces, handwashing, discussing prevention with family and friends
Jeong et al. (2011)[22]	South Korea	H1N1	880;<br>76.3% aged 18-39, 22.4% aged 40-59, 1.4% 60 or over;<br>64.5% female	Hospital employees	The Korea Center of Disease Control and Prevention's recommended behaviours, including washing hands, using a tissue when coughing or sneezing, reducing outings

For peer review only - http://bmjopen.bmj.com/site/about/guidelines.xhtml

Chor et al. (2012)[23]	China (Hong Kong), Singapore, UK	H1N1	2100 (1556 Hong Kong, 284 Singapore, 260 UK); Mean age 35 (Hong Kong), 28 (Singapore), 34 (UK); Gender not reported	Doctors, nurses, allied healthcare workers	Washing hands before each patient contact, washing hands after each patient contact, wearing gloves during patient contact, wearing a surgical mask even when not involved in direct patient care, wearing a mask during patient contact
Koh et al. (2009)[24]	Indonesia, Singapore	Avian influenza	333 from Indonesia v 1321 from Singapore plus 51 focus group participants from Indonesia; Quantitative study population: Indonesia: 55.4% aged 40 or under, Singapore: 43.6% aged 40 or under, Qualitative study: Age range 22-56; Quantitative study population: Indonesia: 75.7% female, Singapore: 86.4% female, Qualitative study: 94.1% female	Primary healthcare workers. Quantitative study: Indonesia: 17.6% doctors, 14.6% nurses, 67.8% other. Singapore: 14.6%, 34.2%, 51.2%. 'Other' includes therapists, paramedics, ambulance crew, attendants, pharmacists, social workers, cleaners and administrative staff Qualitative study: 13 physicians, 19 paramedics, 19 nurses	'Adhering to infection control protocols and recommended measures'

For peer review only

Wong et al. (2005)[25]	China (Hong Kong) (n=137), Canada (n=51)	SARS	188;<br>Age not reported;<br>Gender not reported	Family medicine tutors (Hong Kong), family physicians (Canada)	Testing patient temperature as a routine procedure, always wearing mask during consultations, cleaning work surface with antiseptics at least once a day, support staff all wearing masks, wearing gowns for patient encounters, washing hands between every patient, closing clinic, quarantine
Kamate et al. (2020)[26]	International (30.7% from Asia, 25.0% from North/South Americas, 16.3% from Europe, 22.6% from Africa, 5.4% from 'other' (Australia	COVID-19	860;<br>Age reported;<br>Gender not reported	Dental practitioners	Staff sensitised as per World Health Organization guidelines for the prevention of COVID-19, including travel history when recording patient histories, discussing risk and preventive measures related to COVID-19 with patients, showing patients signs and symptoms of COVID-19, taking 'preventive measures' against COVID-19, effect on social life

For peer review only - http://bmjopen.bmj.com/site/about/guidelines.xhtml

	and Antarctica)				
Hu et al. (2012)[27]	China	H1N1	650; Median age 28, IQR 25-33; 76.3% female	Nurses (421), physicians (229)	Hand hygiene, gloves, gown, mask (surgical and N95 respirator), goggles
Alsahafi & Cheng (2016)[28]	Saudi Arabia	MERS	1216; Age not reported; 56.4% female (nurses), 25.5% female (physicians), 23.9% female (other healthcare workers)	Nurses (56.3%), physicians (22%), other healthcare workers (21.7%)	Handwashing after patient contact, wearing of surgical mask during patient contact, wearing of N95 mask during patient contact, training on infectious behaviours, perceived barriers to infection control practices
Alsubaie et al. (2019)[29]	Saudi Arabia	MERS	591; Age not reported; 78% female (non-physicians) and 31% female (physicians)	Physicians (55%), non-physicians – including nurses, technicians and respiratory therapists (45%)	Type of preventive behaviours and practices that changed during the outbreak, including hand hygiene and avoidance behaviours such as avoidance of hand-shaking, avoidance of direct contact with people suffering influenza-like illnesses and avoidance of public facilities

For peer review only

Chau et al. (2008)[30]	China (Hong Kong)	SARS	96 (observational study), 109 (interviews); Age not reported; Gender not reported	Healthcare workers and support workers; for interviews: 32 healthcare workers, 30 health care assistants, 47 managers including nursing officers, ward managers, nurse specialists and infection control nurses	Total performance score based on compliance with hand hygiene, mask use, gown use, goggle/face shield precautions, glove use, handling patient care equipment and articles correctly, routine and terminal cleaning, handling linen and laundry, and terminal cleaning of isolation room
Koh et al. (2005)[31]	Singapore	SARS	15,025; Mean age 36.6; 82% female	Healthcare workers from nine major healthcare institutions, incl. doctors, nurses, physiotherapists, attendants, cleaners, pharmacists, managerial staff, clerical staff, service counter staff, other	Hospital's recommended protective practices
May et al. (2010)[32]	USA	H1N1	261; Students: 26% aged under 24, 61% aged 24-29, 10% aged 30-34, 3% 35 and	Medical students (194) and residents (67), at an urban medical teaching centre	Hand sanitiser, handwashing, cough etiquette, disinfection, surgical mask when ill with influenza-like illness, surgical mask in patient care when

For peer review only

			over, Residents: 55% aged 24-29, 36% aged 30-34, 9% 35 and over; Students: 62% female, Residents: 50% female		seeing patients with influenza-like illness, N95 when seeing patients with influenza-like illness, social distancing
Mitchell et al. (2012)[33]	Canada	H1N1	986; Median age 36, range 21-79; 85% female	Nurses (80%), respiratory therapists (14%), physicians (6%)	Use of PPE
Parker & Goldman (2006)[34]	Canada	SARS	116; 27% aged 20-29, 42% aged 30-39, 23% aged 40-49, 8% aged 50 and over; 70% female	Paediatric emergency department staff: 28% physicians, 41% nursing staff, 21% trainees, 10% paramedical staff involved with direct patient care	Wearing N95 mask when examining patients, wearing gown when examining patients, handwashing before and after patient contact, wearing N95 mask at all times in the emergency department, wearing eye protection when examining patients, wearing gloves when examining patients
Shigayeva et al. (2007)[35]	Canada	SARS	795; Median age 41, range 21-67; 74.3% female	46.3% nurses, 13.5% physicians, 14.2% respiratory therapists, 10.3% medical imaging	Use of barrier precautions, practices for removing PPE, infection control training

For peer review only - http://bmjopen.bmj.com/site/about/guidelines.xhtml

				technologists, 15.7% other incl. service assistants, physiotherapists, lab technologists, paramedics, housekeeping staff, ward clerks and social workers	
Vinck et al. (2011)[36]	Netherlands	H1N1	166;<br>4% aged 25 or under, 26% aged 26-35, 26% aged 36-45, 32% aged 46-55, 10% aged 56-65;<br>66% female	Public health physicians (46%), public health nurses (51%), health department managers (2%)	Compliance with control measures: applying case definition (i.e. how consistently staff applied the criteria for case definition that was issued to identify suspected patients), amount of consultation with the centralised assessment system for the final classification of patients, use of PPE during patient sampling and home visits, and informing patients and contacts about isolation measures
Alshammari et al. (2018)[37]	Saudi Arabia	MERS	87;<br>Age not reported;<br>Gender not reported	Physicians and nurses	Hand hygiene compliance

For peer review only

Jaeger et al. (2011)[38]	USA	H1N1	63;<br>Median age 35, range 19-74;<br>76% female	70% worked in inpatient settings. 38% clinical practitioner, 10% physician/nurse practitioner/physician assistant, 29% Registered nurse/Registered Dietician, 52% allied health staff (incl. radiology, emergency department and respiratory technicians, phlebotomists, licensed vocational nurses and medical and nursing assistants), 10% support staff (incl. office workers, patient transport and environmental services workers)	PPE use, glove use during index patient exposures, mask or N95 respirator use during index patient encounters

For peer review only

Moore et al. (2005)[39]	Canada	SARS	97;<br>Mean age 43.1, range 26-64;<br>78% female	34% managers, 24% registered nurses, 9% support staff, 8% medical technologists, 6% respiratory therapists, 4% infection control practitioners, 4% physicians, 1% administration staff, 1% pharmacists and 1% physiotherapists	Experience of infection control practices including PPE
Tan et al. (2006)[40]	Singapore	SARS	8;<br>Age not reported;<br>25% female	Family physicians	Participants discussed encounters with suspected SARS patients including their clinical assessment and management of patients in clinics, problems encountered, and use of PPE and other preventive measures
Corley et al. (2010)[41]	Australia	H1N1	32 (questionnaire study), 16 (focus groups)<br>Age not reported;<br>Gender not reported	Questionnaire: 28 nursing staff, 4 medical staff;<br>Focus groups: 4 senior medical staff, 4 senior	How resources were managed during the pandemic

For peer review only - http://bmjopen.bmj.com/site/about/guidelines.xhtml

				nursing staff, 8 bedside nursing staff	
Hsu et al. (2011)[42]	Singapore	H1N1	123;<br>Age not reported;<br>Gender not reported	Final-year medical students	Hand hygiene
Chia et al. (2005)[43]	Singapore	SARS	6,198;<br>Mean age 35.8 (doctors), 34.6 (nurses), 38.4 (clerical staff);<br>27.1% female (doctors), 48.8% female (nurses), 77.1% female (clerical staff)	Doctors (14.1%), nurses (4404 – 71%), clerical staff (14.9%)	Perception of risk of infection and preventive measures
Ki et al. (2019)[44]	South Korea	MERS	446;<br>Age not reported;<br>Gender not reported	Medical employees; 228 in the emergency department, 218 in general wards	PPE (face shields or goggles, surgical masks, N95 respirators, gloves, disposable gowns, disposable coveralls, waterproof boots, head cover), hand-washing, hand-rubbing, isolation
Wong et al. (2004)[45]	China (Hong Kong)	SARS	320;<br>Mean age of general practitioners 44.4, mean	General practitioners (137), family medicine tutors (183)	Clinical practices and behaviours: wearing mask during all consultations, wearing goggles, wearing gowns,

For peer review only - http://bmjopen.bmj.com/site/about/guidelines.xhtml

			age of family medicine tutors not reported; 17.6% female (general practitioners), 15.3% female (family medicine tutors)		washing hands after seeing patients, insisting patients wear masks, routinely testing patients' temperatures, keeping greater distance from patients, seeing patients at faster pace, avoiding physical examination, requesting more blood tests/chest radiographs, over-prescribing antibiotics, cancelling own appointments, cancelling specialist appointments, difficulties making specialty referrals, closing clinic, reducing working hours, quarantine, requiring staff to wear masks/disposable gloves, cleaning work surfaces with antiseptics, taking staff temperatures, quarantined leave for staff, showering before leaving work, washing hands before entering home, using disinfectant to clean home, staying away from home, wearing a mask at home, sending family away

For peer review only

For peer review only - http://bmjopen.bmj.com/site/about/guidelines.xhtml

Yassi et al. (2005)[46]	Canada	SARS	Participants were those reported in Moore et al. (2005)	Participants were those reported in Moore et al. (2005)	Participants discussed barriers to wearing PPE and following infection control procedures
DiGiovanni et al. (2004)[47]	Canada	SARS	195;<br>Age not reported;<br>Gender not reported	76 physicians, 52 nurses, 67 'other' including receptionists, technicians, social workers, research assistants and clinic/office managers	Quarantine
Edeghere et al. (2015)[48]	UK	H1N1	20;<br>Mean age 43.9;<br>80% female	Frontline healthcare workers: 10 clinical attendants, 7 nurses, 3 general practitioners	Use of PPE and infection control measures
Khalid et al. (2016)[49]	Saudi Arabia	MERS	117;<br>Mean age 38.55;<br>76% female	Healthcare workers who worked in high-risk areas. 76% nurses, 14% physicians, 10% respiratory therapists. 65% worked in critical care units, 20% in emergency	Perceived stressors

For peer review only - http://bmjopen.bmj.com/site/about/guidelines.xhtml

				medicine, 15% in outpatient family medicine	
Locatelli et al. (2012)[50]	USA	H1N1	33;<br>Age not reported;<br>Gender not reported	Infection control key informants at nationally dispersed Veterans Affairs healthcare facilities	Communication about infection control
Rebmann & Wagner (2009)[51]	USA (n=37), UK (n=1, Canada (n=1), Australia (n=1)	H1N1	40;<br>2.5% aged 30-39, 27.5% aged 40-49, 45.0% aged 50-59, 22.5% aged 60-69, 2.5% 70 or over;<br>92% female	Infection preventionists	Issues relating to infection prevention guidance and PPE
Rowlands (2007)[52]	Singapore	SARS	28;<br>Age not reported;<br>75% female	Medical social workers	Experiences of working during the SARS crisis, and the various infection control strategies used at work
Van Dijk et al. (2015)[53]	Netherlands	H1N1	875;<br>General practitioners: 17.5% under 40, 24.5% aged 40-49, 44.9% aged 50-59, 13.2% aged 60+;	General practitioners (372), practice assistants (503)	Experience and acceptance of the national guidelines for infection control

For peer review only - http://bmjopen.bmj.com/site/about/guidelines.xhtml

			age for practice assistants not reported<br>General practitioners: 39.3% female; gender of practice assistants not reported		
Kang et al. (2018)[54]	South Korea	MERS	7;<br>Age not reported;<br>Gender not reported	Infection control nurse leaders from major hospitals	Use of PPE equipment
Wong et al. (2012)[55]	China (Hong Kong)	H1N1	10;<br>Mean age 34.9, range 24-49;<br>70% female	4 physicians, 3 nurses, 3 healthcare assistants all working in isolation wards	Concerns relating to care of patients with H1N1
Lam & Hung (2013)[56]	China (Hong Kong)	H1N1	10;<br>20% aged 20-25, 20% aged 26-30, 20% aged 31-35, 30% aged 36-40, 10% over 40;<br>100% female	Emergency nurses: 80% staff nurse, 10% advanced practice nurse, 10% nursing officer	Use of PPE
Nickell et al. (2004)[57]	Canada	SARS	2001;	33.1% allied health care professionals, 31.9% areas	Precautionary measures in the workplace: mask, restricted access (own

For peer review only - http://bmjopen.bmj.com/site/about/guidelines.xhtml

			13.6% aged under 30, 25.7% aged 30-39, 30.6% aged 40-49, 30.2% 50 or over;<br>78.8% female	other than patient care e.g. administration, food services, maintenance or research, 25.6% nurses, 9.3% doctors	hospital), restricted meetings, restricted access (other hospitals), gloves, goggles, 'other'
Kang et al. (2018)[58]	South Korea	MERS	27;<br>Mean age 29.5;<br>92.6% female	Nurses	Working experiences and challenges faced during the outbreak
Khoo et al. (2005)[59]	Singapore	SARS	51;<br>Age not reported;<br>Female:male ratio was 2:1	19 doctors, 31 nurses/nursing aides, 1 respiratory therapist	Opinions on 3M powered air-purifying respirator and Stryker powered air-purifying respirator
Straus et al. (2004)[60]	Canada	SARS	14;<br>Age not reported;<br>Gender not reported	Physicians from divisions of infectious diseases, general internal medicine, and critical care medicine at university hospitals	Participants discussed barrier precautions such as protective clothing
Tolomiczenko et al. (2005)[61]	Canada	SARS	300;<br>Mean age 42.6, range 20-75;<br>73.9% female	Registered nurses (25.2%), physicians (20.7%), management (9.5%), services (facilities management, food	Participants discussed patient care and communication about directives

For peer review only - http://bmjopen.bmj.com/site/about/guidelines.xhtml

				services, environmental – 3.7%), health professional (occupational therapists, physiotherapists, speech-language pathologists – 17.0%), other (counsellors, technicians, clerks, chaplains – 23.8%)	
Hsu et al. (2006)[62]	Taiwan	SARS	312;<br>4.0% under 30, 32.9% aged 30-39, 48.5% aged 40-49, 14.6% 50 over over;<br>Gender not reported	Public health nurses	Self-protection equipment
Nhan et al. (2012)[63]	Canada	H1N1	129;<br>Age not reported;<br>Gender not reported	68 infectious diseases specialist/medical microbiologists, 61 public health and preventive medicine specialists	Difficulties and frustrations experienced during the pandemic
Rambaldini et al. (2005)[64]	Canada	SARS	17;<br>Age not reported;	Medical house staff allocated to a general	Participants discussed hospitals' communication of directives

For peer review only - http://bmjopen.bmj.com/site/about/guidelines.xhtml

			Gender not reported	internal medicine clinical teaching unit, infectious disease unit, infectious diseases consultation service, or intensive care unit	
Robertson et al. (2004)[65]	Canada	SARS	10;<br>Mean age 43.1;<br>60% female	4 nurses, 1 hospital executive, 1 dentist, 1 social worker, 1 occupational therapist, 1 paramedic, 1 physician	Experiences of quarantine and perceptions of infection control guidelines
Goulia et al. (2010)[66]	Greece	H1N1	469;<br>Mean age 38.4;<br>68.4% female.	Nurses (44.6%), medical staff (25.6%), allied staff (12.6%), auxiliary staff (17.3%)	Restriction of social contacts, isolation, intended work avoidance, sense of duty

For peer review only

Supplementary Table II. Summary of themes

**Theme**	**Significant association with protective behaviours**	**No significant association with protective behaviours**	**Reported, but not statistically analysed as a potential predictor of protective behaviours**
Socio-demographics and personal characteristics			
Age	Kim & Choi (2016)[20]	Al-Amri et al. (2019)[12]<br>Datta et al. (2011)[13]<br>De Perio et al. (2012)[14]<br>Manabe et al. (2012)[15]<br>Nour et al. (2015)[16]<br>Nour et al. (2017)[17]<br>Pratt et al. (2009)[18]	N/A
Gender	Nour et al. (2015)[16]<br>Jeong et al. (2011)[22]<br>Vinck et al. (2011)[36]	Al-Amri et al. (2019)[12]<br>Datta et al. (2011)[13]<br>De Perio et al. (2012)[14]<br>Nour et al. (2017)[17]<br>Evirgen et al. (2014)[19]<br>Kim & Choi (2016)[20]	N/A

For peer review only

		Taghrir et al. (2020)[21]	
Nationality	Al-Amri et al. (2019)[12]	N/A	N/A
Country	Chor et al. (2012)[23]<br>Koh et al. (2009)[24]*<br>Wong et al. (2005)[25]*<br>Kamate et al. (2020)[26]*	Koh et al. (2009)[24]*<br>Wong et al. (2005)[25]*<br>Kamate et al. (2020)[26]*	N/A
Religion	N/A	Kim & Choi (2016)[20]	N/A
Socio-economic status	Jeong et al. (2011)[22]	N/A	N/A
Marital status	N/A	Pratt et al. (2009)[18]<br>Evirgen et al. (2014)[19]	N/A
Level of education	Al-Amri et al. (2019)[12]	Datta et al. (2011)[13]<br>Pratt et al. (2009)[18]<br>Kim & Choi (2016)[20]<br>Taghrir et al. (2020)[21]	N/A
Personal vulnerabilities	N/A	Evirgen et al. (2014)[19]	N/A
Vulnerable people at home	Evirgen et al. (2014)[19]*	Evirgen et al. (2014)[19]*	N/A
Vaccination status	Hu et al. (2012)[27]	N/A	N/A
Occupational role			

For peer review only

Role	Al-Amri et al. (2019)[12]<br>Pratt et al. (2009)[18]<br>Evirgen et al. (2014)[19]<br>Chor et al. (2012)[23]<br>Alsahafi & Cheng (2016)[28]<br>Alsubaie et al. (2019)[29]<br>Chau et al. (2008)[30]<br>Koh et al. (2005)[31]<br>Mitchell et al. (2012)[33]<br>Parker & Goldman (2006)[34]*<br>Shigayeva et al. (2007)[35]<br>Vinck et al. (2011)[36]<br>Chia et al. (2005)[43]	Datta et al. (2011)[13]<br>Nour et al. (2015)[16]<br>Nour et al. (2017)[17]<br>Parker & Goldman (2006)[34]*<br>Alshammari et al. (2018)[37]<br>Jaeger et al. (2011)[38]	May et al. (2010)[32]
Length of experience	Koh et al. (2005)[31]<br>Mitchell et al. (2012)[33]<br>Vinck et al. (2011)[36]	Al-Amri et al. (2019)[12]<br>Nour et al. (2015)[16]<br>Nour et al. (2017)[17]<br>Pratt et al. (2009)[18]<br>Chau et al. (2008)[30]	N/A
Training and knowledge			

For peer review only - http://bmjopen.bmj.com/site/about/guidelines.xhtml

Receiving training/education	May et al. (2010)[32]*<br>Shigayeva et al. (2007)[35]	Nour et al. (2017)[17]<br>Taghrir et al. (2020)[21]<br>May et al. (2010)[32]*	Alsahafi & Cheng (2016)[28]<br>Moore et al. (2005)[39]<br>Tan et al. (2006)[40]<br>Corley et al. (2010)[41]
Sources of knowledge	Al-Amri et al. (2019) [12]	Kim & Choi (2016)[20]<br>Taghrir et al. (2020)[21]	N/A
Knowledge (of correct practices / outbreak)	Yap et al. (2010)[4]<br>De Perio et al. (2012)[14] *<br>Nour et al. (2015)[16]<br>Kim & Choi (2016)[20]*<br>Jeong et al. (2011)[22]<br>Hu et al. (2012)[27]	Al-Amri et al. (2019)[12]<br>De Perio et al. (2012)[14] *<br>Kim & Choi (2016)[20]*	Alsahafi & Cheng (2016) [28]<br>Hsu et al. (2011)[42]
Work-related factors			
Setting	De Perio et al. (2012)[14]<br>Chau et al. (2008)[30]<br>Mitchell et al. (2012)[33]<br>Shigayeva et al. (2007)[35]<br>Jaeger et al. (2011)[38]<br>Chia et al. (2005)[43]<br>Ki et al. (2019)[44]*<br>Wong et al. (2004)[45]	Evirgen et al. (2014)[19]<br>Taghrir et al. (2020)[21]<br>Ki et al. (2019)[44]*	N/A

For peer review only - http://bmjopen.bmj.com/site/about/guidelines.xhtml

Contact with confirmed cases	De Perio et al. (2012)[14]<br>Pratt et al. (2009)[18]*<br>Shigayeva et al. (2007)[35]<br>Chia et al. (2005)[43]<br>Wong et al. (2004)[45]	Pratt et al. (2009)[18]*<br>Evirgen et al. (2014)[19]	N/A
Workload	Pratt et al. (2009)[18]<br>Chor et al. (2012)[23]*<br>Shigayeva et al. (2007)[35]<br>Vinck et al. (2011)[36]	Chor et al. (2012)[23]*	Chau et al. (2008)[30]<br>Moore et al. (2005)[39]<br>Corley et al. (2010)[41]<br>Yassi et al. (2005)[46]
Monitoring of compliance	Hu et al. (2012)[27]+	N/A	Hu et al. (2012)[27]+<br>Hsu et al. (2011)[42]<br>DiGiovanni et al. (2004)[47]
Patient encounters	De Perio et al. (2012)[14]<br>Shigayeva et al. (2007)[35]	N/A	N/A
Personal protective behaviour-related factors			
Availability of resources	Hu et al. (2012)[27]+<br>Mitchell et al. (2012)[33]*	Mitchell et al. (2012)[33]*	De Perio et al. (2012)[14]<br>Hu et al. (2012)[27]+<br>Moore et al. (2005)[39]<br>Tan et al. (2006)[40]

For peer review only - http://bmjopen.bmj.com/site/about/guidelines.xhtml

For peer review only

			Corley et al. (2010)[41]<br>Edeghere et al. (2015)[48]<br>Khalid et al. (2016)[49]<br>Locatelli et al. (2012)[50]<br>Rebmann & Wagner (2009)[51]<br>Rowlands (2007)[52]<br>Van Dijk et al. (2015)[53]<br>Hsu et al. (2006)[62]
Perceived difficulty of protective behaviour	N/A	N/A	Van Dijk et al. (2015)[53]<br>Kang et al. (2018)[54]
Logistical issues	N/A	N/A	Rebmann & Wagner (2009)[51]<br>Kang et al. (2018)[54]<br>Wong et al. (2012)[55]
Perceived effectiveness	Yap et al. (2010)[4]<br>Hu et al. (2012)[27]<br>Mitchell et al. (2012)[33]<br>Parker & Goldman (2006)[34]*	Parker & Goldman (2006)[34]*	Yassi et al. (2005)[46]<br>Kang et al. (2018)[54]
Perceived importance	N/A	N/A	Vinck et al. (2011)[36]<br>Hsu et al. (2011)[42]

For peer review only - http://bmjopen.bmj.com/site/about/guidelines.xhtml

Incentives	N/A	N/A	Jeong et al. (2011)[22]<br>Hsu et al. (2011)[42]<br>Kang et al. (2018)[54]
Convenience	N/A	N/A	De Perio et al. (2012)[14]<br>Hu et al. (2012)[27]<br>Moore et al. (2005)[39]<br>Tan et al. (2006)[40]<br>Corley et al. (2010)[41]<br>Hsu et al. (2011)[42]<br>Kang et al. (2018)[54]<br>Lam & Hung (2013)[56]<br>Nickell et al. (2004)[57]
Comfort	Mitchell et al. (2012)[33]*	Mitchell et al. (2012)[33]*	Moore et al. (2005)[39]<br>Tan et al. (2006)[40]<br>Corley et al. (2010)[41]<br>Yassi et al. (2005)[46]<br>Khalid et al. (2016)[49]<br>Rowlands (2007)[52]<br>Lam & Hung (2013)[56]<br>Nickell et al. (2004)[57]<br>Kang et al. (2018)[58]

For peer review only

			Khoo et al. (2005)[59]<br>Straus et al. (2004)[60]
Impact on patient care	Hu et al. (2012)[27]+	N/A	Hu et al. (2012)[27]+<br>Tan et al. (2006)[40]<br>Corley et al. (2010)[41]<br>Rowlands (2007)[52]<br>Lam & Hung (2013)[56]<br>Nickell et al. (2004)[57]<br>Khoo et al. (2005)[59]<br>Straus et al. (2004)[60]<br>Tolomiczenko et al. (2005)[61]
Guidance			
Issues with availability or understanding	N/A	N/A	Alsahafi & Cheng (2016)[28]<br>Chau et al. (2008)[30]<br>Moore et al. (2005)[39]<br>Tan et al. (2006)[40]<br>Corley et al. (2010)[41]<br>Yassi et al. (2005)[46]<br>Locatelli et al. (2012)[50]<br>Rebmann & Wagner (2009)[51]<br>Van Dijk et al. (2015)[53]

For peer review only

			Kang et al. (2018)[54]<br>Wong et al. (2012)[55]<br>Lam & Hung (2013)[56]<br>Kang et al. (2018)[58]<br>Tolomiczenko et al. (2005)[61]<br>Hsu et al. (2006)[62]<br>Nhan et al. (2012)[63]<br>Rambaldini et al. (2005)[64]<br>Robertson et al. (2004)[65]
Distress and risk perception			
Distress	Chia et al. (2005)[43]<br>Wong et al. (2004)[45]	N/A	DiGiovanni et al. (2004)[47]
Risk perception	Kim & Choi (2016)[20]<br>Taghrir et al. (2020)[21]<br>Jeong et al. (2011)[22]<br>Chor et al. (2012)[23]*<br>Parker & Goldman (2006)[34]<br>Goulia et al. (2010)[66]	Chor et al. (2012)[23]*	Moore et al. (2005)[39]<br>DiGiovanni et al. (2004)[47]<br>Rebmann & Wagner (2009)[51]
Attitudes and behaviours of others			

For peer review only

Non-compliance of others	N/A	N/A	Hu et al. (2012)[27]<br>Chau et al. (2008)[30]<br>Moore et al. (2005)[39]<br>Hsu et al. (2011)[42]<br>Yassi et al. (2005)[46]<br>DiGiovanni et al. (2004)[47]<br>Locatelli et al. (2012)[50]
Attitudes of family members	N/A	N/A	Moore et al. (2005)[39]<br>Yassi et al. (2005)[46]
Other potential predictors of compliance			
Complacency	N/A	N/A	Tan et al. (2006)[40]
Commitment	N/A	N/A	Alsahafi & Cheng (2016)[28]<br>Moore et al. (2005)[39]
Previous experience with pandemic	N/A	N/A	Moore et al. (2005)[39]
Passing of time	N/A	N/A	Rebmann & Wagner (2009)[51]
Forgetting	Hu et al. (2012)[27]	N/A	N/A
Death of a doctor	N/A	N/A	Tan et al. (2006)[40]

For peer review only - http://bmjopen.bmj.com/site/about/guidelines.xhtml

Effort-reward imbalance	Pratt et al. (2009)[18]	N/A	N/A
Consulting with experts	N/A	Manabe et al. (2012)[15]	N/A
Lack of staff	N/A	N/A	Moore et al. (2005)[39]<br>Corley et al. (2010)[41]
PPE fit-testing procedures	N/A	N/A	Moore et al. (2005)[39]
Professionalism	N/A	N/A	Yassi et al. (2005)[46]
Use of public transport	N/A	Evirgen et al. (2014)[19]	N/A
Loss of income	N/A	N/A	DiGiovanni et al. (2004)[47]
Overcrowding	N/A	N/A	Alsahafi & Cheng (2016)[28]

*Appears in both 'significant association' and 'no significant association' column due to both significant and non-significant results being reported for different groups of participants.
+Contains both statistically analysed data and non-statistically analysed (e.g. qualitative data, descriptive survey results).

For peer review only

Supplementary Table III. Summary of evidence

Theme	Evidence
Socio-demographics and personal characteristics	
Age	Al-Amri et al. (2019)[12]: Compliance was not significantly associated with age (p=0.785).
	Datta et al. (2011)[13]: Protective behaviours were not significantly associated with age.
	De Perio et al. (2012)[14]: Compliance was not significantly associated with age.
	Manabe et al. (2012)[15]: For physicians only (other roles not included in analysis), age was not significantly associated with wearing PPE to see suspected avian influenza patients.
	Nour et al. (2015)[16]: There was no significant difference in median protective practice scores between age groups (under 30s v 30 and over) (p=0.582).
	Nour et al. (2017)[17]: There was no significant difference in median protective practice scores between age groups (under 30s v 30 and over).
	Pratt et al. (2009)[18]: Age was not significantly associated with compliance.
	Kim & Choi (2016)[20]: Age was significantly positively correlated with preventive behaviours (p<0.05); older staff were more likely to comply.
Gender	Al-Amri et al. (2019)[12]:Compliance was not significantly associated with gender (p=0.241).
	Datta et al. (2011)[13]: Protective behaviours were not significantly associated with gender.
	DePerio et al. (2012)[14]: Compliance was not significantly associated with gender.

For peer review only - http://bmjopen.bmj.com/site/about/guidelines.xhtml

	Nour et al. (2015)[16]: Female staff were significantly more likely to comply with preventive behaviours (p<0.001).
	Nour et al. (2017)[17]: There was no significant difference in median protective practice scores between genders.
	Evirgen et al. (2014)[19]: Behaviour was not significantly associated with gender.
	Kim & Choi (2016)[20]: Gender was not significantly associated with preventive behaviours.
	Taghrir et al. (2020)[21]: There was no significant association between preventive behaviours and gender.
	Jeong et al. (2011)[22]: Female gender was a significant predictor of preventive behaviours.
	Vinck et al. (2011)[36]: Female staff reported higher compliance than males (p=0.002) and greater use of PPE (p=0.037).
Nationality	Al-Amri et al. (2019)[12]:Compliance was significantly associated with nationality (p=0.047), with Saudi staff showing higher compliance than non-Saudi staff in the same city.
Country	Chor et al. (2012)[23]: Hong Kong reported the highest compliance with all infection control practices, except for wearing a mask during patient contact, which was second to Singapore (70.4% v 87.7%). Wearing masks in clinical areas while not involved with patient contact was lowest for the UK (25.3% v 96.4% for Hong Kong and 82.3% for Singapore), as was use of masks (either surgical or N95) during physical contact with patients (62% for the UK v 70.4% for Hong Kong and 87.7% for Singapore).
	Koh et al. (2009)[24]: Healthcare workers in Singapore were significantly more likely than those in Indonesia to screen patients and clinic visitors for fever (92.9% v 80.8%, p<0.001), limit number of visitors to the clinic (88.7% v 46.0%, p<0.001), post prominent infection control notices to patients/visitors (97.4% v 80.3%, p<0.001), do temperature checks for staff (91.5% v 61.1%, p<0.001); wear gowns (91.1% v 61.5%, p<0.001); wear paper masks (43.2% v 11.7%, p<0.001); wear surgical masks (28.6% v 12.6%, p<0.001); wear N95 masks (94.0% v 82.4%, p=0.004); and carry out regular handwashing (98.6% v 87.0%, p<0.001). However, there were no significant differences between countries in wearing gloves (94.2% v 93.3%, p=0.502); wearing goggles (77.6% v 67.8%, p=0.140); using powered air purifier

For peer review only - http://bmjopen.bmj.com/site/about/guidelines.xhtml

	respirators (86.1% v 85.4%, p=0.550); using alcohol hand rub (95.6% v 90.8%, p=0.183); or using a special room to isolate bird flu patients (97.9% v 97.5%, p=0.254).
	Wong et al. (2005)[25]: Physicians in Hong Kong were less likely than those in Canada to quarantine themselves after contact with a probable or suspected SARS case (19.4% v 77.1%, p<0.01) or give quarantine leave to their staff (59.7% v 95%, p<0.01). However, physicians in Hong Kong were more likely than those in Canada to test patient temperature as a routine procedure (68.1% v 47.1%, p<0.01), wear a mask during consultations (97.7% v 52.9%, p<0.01) and have their support staff wear masks (97.8% v 68.6%, p<0.01). No significant differences between countries were found for wearing gowns for every patient encounter or washing hands between every encounter.
	Kamate et al. (2020)[26]: There were no significant differences between countries in terms of discussing risk of COVID-19 with patients; discussing preventive measures with patients; showing patients the signs and symptoms of COVIF-19; or taking preventive measures themselves. However, there was a significant difference in whether they included travel history while recording patients' histories (99.0% in Asia v 97.2% in Americas v 94.3% in Europe v 94.3% in Africa v 89.4% in Australia/Antarctica, p=0.025).
Religion	Kim & Choi (2016)[20]: Being religious or not was not significantly associated with preventive behaviours.
Socio-economic status	Jeong et al. (2011)[22]: 'High or middle economic state' significantly predicted preventive behaviours.
Marital status	Pratt et al. (2009)[18]: Marital status was not significantly associated with compliance.
	Evirgen et al. (2014)[19]: Behaviour was not significantly associated with marital status.

For peer review only

For peer review only - http://bmjopen.bmj.com/site/about/guidelines.xhtml

Level of education	Al-Amri et al. (2019)[12]: Compliance was significantly associated with qualifications (p=0.037) with more highly-qualified staff being more likely to comply. Percentage of poor, good and excellent preventive practices, by qualification: MBBS: 30.6%, 69.4%, 0.0%; Diploma: 46.7%, 46.7%, 6.7%; Masters: 43.8%, 43.8%, 12.5%; Doctorate/fellowship: 66.7%, 22.2%, 11.1%.
	Datta et al. (2011)[13]: Protective behaviours were not significantly associated with level of education.
	Pratt et al. (2009)[18]: Highest level of education was not significantly associated with compliance.
	Kim & Choi (2016)[20]: Level of education (junior/senior grade) was not significantly associated with compliance.
	Taghrir et al. (2020)[21]: There was no significant association between preventive behaviours and education level.
Personal vulnerabilities	Evirgen et al. (2014)[19]: Behaviour was not significantly associated with being pregnant or having a chronic disease.
Vulnerable people at home	Evirgen et al. (2014)[19]: Behaviour was not significantly associated with having a pregnant spouse, elderly person, or school-aged child at home. However, staff with babies at home were more likely to comply with protective behaviours (p<0.005).
Vaccination status	Hu et al. (2012)[27]: Vaccination for H1N1 influenza was an independent predictor of high compliance to PPE (p<0.001).
Occupational role	
Role	Al-Amri et al. (2019)[12]: Practice-related adherence to guidelines differed significantly according to position (p=0.035), with specialists having the highest percentage of excellent practice.
	Datta et al. (2011)[13]: Protective behaviours were not significantly associated with occupation.
	Nour et al. (2015)[16]: There was no significant difference in median protective practice scores between occupational groups (p=0.433).

For peer review only - http://bmjopen.bmj.com/site/about/guidelines.xhtml

	Nour et al. (2017)[17]: There was no significant difference in median protective practice scores between occupational groups (nurses v physicians v specialists v technicians).
	Pratt et al. (2009)[18]: Job status (full-time, part-time or casual) was reported to be significantly associated with compliance although the authors do not state which group were more likely to comply. Medical/surgical unit as a primary area of practice (compared to maternal/newborn or paediatrics, emergency or critical care, or 'other' roles) was also reported to be significantly associated with compliance but again the authors do not state in which way.
	Evirgen et al. (2014)[19]: Professors had the highest average score for positive behaviours.
	Chor et al. (2012)[23]: In Hong Kong and Singapore, employment type was the strongest predictor of adoption of infection control practices, with nurses showing significantly higher compliance with most measures compared to doctors in both countries, and allied healthcare workers also reporting significantly higher compliance with most measures compared to doctors in Hong Kong. Administrative staff in Hong Kong reported compliance similar to that of other healthcare workers, whereas administrative staff in the UK reported significantly lower compliance than doctors.
	Alsahafi & Cheng (2016)[28]: There appeared to be significant differences between physicians, nurses and other healthcare staff in terms of handwashing after patient contact ($p=0.04$), wearing of surgical mask during patient contact ($p<0.001$), and wearing of N95 mask during patient contact ($p=0.009$). It is not clear from the data presented which groups are significantly more likely to comply, but perhaps nurses are slightly more likely to comply than physicians who are slightly more likely to comply than other healthcare staff.
	Alsubaie et al. (2019)[29]: Non-physicians were significantly more likely than physicians to report improvement (that is, improvement since pre-outbreak) in hand hygiene compliance at the hospital (90% v 80%, $p<0.002$), improvement in compliance with universal precautions at the hospital (85.5% v 69%, $p=0.001$), increase in avoidance behaviour towards people with flu-like symptoms (83% v 72%, $p<0.009$), decrease in using public facilities (66% v 28.7%,

For peer review only - http://bmjopen.bmj.com/site/about/guidelines.xhtml

	p=0.001), decrease in hand-shaking (60% v 27.7%, p<0.001), and decrease in social visits e.g. to friends (60% v 21.7%, p<0.001).
	Chau et al. (2008)[30]: Nurses performed better in a number of isolation precautions (including hand-washing, hand rub use, glove use, handling of linen and laundry, and handling of patient care equipment) than other roles. Doctors performed better when it came to wearing masks and putting on gowns.
	Koh et al. (2005)[31]: All other occupational groups, including nurses (OR 0.49; 95% CI 0.39-0.61) reported greater difficulty adhering to preventive measures compared with doctors.
	May et al. (2010)[32]: Medical students appeared more likely than residents to use hand sanitiser both at work and home, wash hands both at work and home, and practice cough etiquette at work and home, while residents appeared more likely to use disinfectant and use surgical masks and N95 masks when seeing patients with influenza-like illness – however, no statistical analysis is presented.
	Mitchell et al. (2012)[33]: Compared with physicians, nurses and respiratory therapists were significantly more compliant with wearing N95 respirators and gloves for aerosol-generating medical procedures and with wearing protective eyewear and gloves for patient care not involving these procedures. Nurses and respiratory therapists were also significantly more compliant with performing a user seal check after donning an N95 respirator than physicians (35% and 40% v 19%, p=0.023).
	Parker & Goldman (2006)[34]: Physicians reported more handwashing before and after all patient contacts compared with nurses and trainees (p<0.05); however, there were no significant differences between in occupational groups in compliance with wearing an N95 mask when examining patients, wearing a gown when examining patients, wearing an N95 mask at all times in the emergency department, wearing eye protection when examining patients, or wearing gloves when examining patients.

For peer review only - http://bmjopen.bmj.com/site/about/guidelines.xhtml

	Shigayeva et al. (2007)[35]: Clinically trained health professional status was reported to be a significant predictor of consistent adherence (statistics not reported). Safe removal of PPE was significantly associated with being a nurse (OR 1.3, 95% CI, 1.1-1.6).
	Vinck et al. (2011)[36]: Public health physicians followed the case definition less strictly than public health nurses (p=0.000).
	Alshammari et al. (2018)[37]: No significant difference in hand hygiene compliance between physicians and nurses.
	Jaeger et al. (2011)[38]: Use of PPE stratified by healthcare role did not yield significant differences.
	Chia et al. (2005)[43]: The risk of using a less effective level of respiratory protection was lowest among doctors, followed by nurses and clerical staff.
Length of experience	Al-Amri et al. (2019)[12]:Compliance was not significantly associated with length of experience in primary care (p=0.265).
	Nour et al. (2015)[16]: There was no significant association between median protective practice scores and years of experience (p=0.674).
	Nour et al. (2017)[17]: There was no significant association between median protective practice scores and years of experience.
	Pratt et al. (2009)[18]: Years of nursing experience were not significantly associated with compliance.
	Chau et al. (2008)[30]: Compliance was not significantly associated with years of experience.
	Koh et al. (2005)[31]: The longer the work experience, the less likely staff were to experience difficulty in adhering to preventive measures (OR 1.21; 95% CI 1.13-1.29).

For peer review only

	Mitchell et al. (2012)[33]: Staff who had worked in health care for more than eleven years were significantly more compliant with wearing protective eyewear during aerosol-generating medical procedures than those who had worked for eleven years or less (69% v 54%, p<0.001), and also with wearing gowns (82% v 69%, p<0.001), gloves (92% v 85%, p=0.006) and N95 respirators (75% v 60%, p<0.001). For patient care not involving aerosol-generating medical procedures, staff with more than eleven years of experience were again significantly more likely to comply with wearing protective eyewear (38% v 31%, p=0.04), gowns (82% v 69%, p<0.001), N95 respirators (43% v 31%, p=0.003) and surgical masks (18% v 11%, p<0.016).
	Vinck et al. (2011)[36]: Compliance was higher in those who had been working in the public health service for one to ten years than those who had worked for eleven years or longer (p=0.034).
Training and knowledge	
Receiving training/education	Nour et al. (2017)[17]: After the training intervention in the study, there was a non-significant increase in preventive practices (p=0.168) – but compliance was good pre-intervention too.
	Taghrir et al. (2020)[21]: There was no significant association between preventive behaviours and having received education on COVID-19.
	Alsahafi & Cheng (2016)[28]: 22.8% of participants reported having received training about dealing with infectious disease outbreaks, 37.1% training in infection control policies and procedures, 54.4% training in hand hygiene and 45.6% training in N95 mask-wearing techniques. A high proportion of participants believed that insufficient training in infection control procedures contributed to poor compliance and the risk of infection (92.4% of nurses, 91% of physicians and 90.9% of other healthcare staff).

For peer review only

For peer review only - http://bmjopen.bmj.com/site/about/guidelines.xhtml

	May et al. (2010)[32]: There was a significant association between level of training and use of currently recommended CDC guidelines for patients with influenza-like illness, for fellows only (p=0.003); however, the number of fellows within the sample was small (n=5).
	Shigayeva et al. (2007)[35]: Recent infection control training was a significant predictor of consistent adherence to recommended behaviours (interactive training: OR 2.7, 95% CI, 1.7-4.4; passive training: OR 1.7, 95% CI, 1.0-3.0).
	Moore et al. (2005)[39]: Many participants reported having received poor prior training in infection control and therefore having to use new procedures and equipment without training or experience.
	Tan et al. (2006)[40]: Participants reported that little information on the disease or methods of control were available from previous education and training, which made them feel helpless.
	Corley et al. (2010)[41]: Participants believed that an infection control course with yearly refreshers would be useful and help improve compliance.
Sources of knowledge	Al-Amri et al. (2019)[12]: Those who attended CME activities had significantly higher levels of excellent practice than those who did not attend a CME activity (11.1% vs 1.5%, p=0.002). Participants who had textbooks as a source of knowledge had the highest percent of excellent practice (p<0.001).
	Kim & Choi (2016)[20]: Source of knowledge of MERS (television, or other) was not significantly associated with preventive behaviours.
	Taghrir et al. (2020)[21]: There was no significant association between preventive behaviours and source of information (WHO, CDC, or UpToDate v national guidelines v both v others).
Knowledge (of correct practices / outbreak)	Yap et al. (2010)[4]: There was a significant positive correlation between knowledge about H1N1/preventive behaviours, and practice of preventive behaviours (p<0.01).
	Al-Amri et al. (2019)[12]: Practice-related adherence to guidelines was not significantly associated with knowledge.

For peer review only - http://bmjopen.bmj.com/site/about/guidelines.xhtml

	De Perio et al. (2012)[14]: Knowledge of correct PPE was significantly associated with compliance at one hospital (p=0.047) but not the other three hospitals in the study.
	Nour et al. (2015)[16]: There was a significant positive association between knowledge and practice (p<0.001).
	Kim & Choi (2016)[20]: Having received MERS education was not significantly associated with preventive behaviours (p=0.209). However, in a correlational analysis, preventive behaviours were significantly correlated with MERS-related knowledge (p<0.01).
	Jeong et al. (2011)[22]: A score for 'cues to action' (consisting of consulting news or articles about the pandemic, being interested in methods for health promotion, and wishing to be educated on effective ways of preventing infection) was significantly associated with compliance (OR 2.63, CI 1.81-3.81).
	Hu et al. (2012)[27]: Self-reported adequate knowledge of self-protection and patient protection was significantly correlated with the perception of further improvement of PPE compliance (p<0.001).
	Alsahafi & Cheng (2016)[28]: A high proportion of participants believed that lack of knowledge about the mode of transmission contributed to poor compliance (94.2% of nurses, 90% of physicians and 90.2% of other healthcare staff).
	Hsu et al. (2011)[42]: 1.9% of participants believed lack of education about correct practices prevented hand hygiene compliance. 6.5% of participants believed improving undergraduate education efforts was the most important strategy for improving hand hygiene compliance. 7.3% of participants believed incorporating hand hygiene into examinations and the student internship marking scheme was the most important strategy.
Work-related factors	
Setting	De Perio et al. (2012)[14]: Staff who worked on an intensive care unit rotation during the study period were more likely to be in the 'high adherence' group (OR, 3.43; 95% CI, 1.02-11.56). Staff who worked on an inpatient ward rotation were less likely to be classified as high adherence (OR, 0.22, 95% CI, 0.06-0.80).

For peer review only - http://bmjopen.bmj.com/site/about/guidelines.xhtml

	Evirgen et al. (2014)[19]: Behaviour was not significantly associated with department at work or the department where the participant spends the most time.
	Taghrir et al. (2020)[21]: There was no significant association between preventive behaviours and ward worked on.
	Hu et al. (2012)[27]: Compliance rates significantly differed between institutions, from 0% to 88.1%, although all institutions were adult intensive care units.
	Chau et al. (2008)[30]: Those working in rehabilitation hospitals were significantly more likely to comply with protective behaviours overall, and use hand rub, than participants working in acute hospitals.
	Mitchell et al. (2012)[33]: Staff working in an intensive care unit or designated influenza ward were more compliant with wearing an N95 respirator for aerosol-generating medical procedures than those working in an emergency department (78% and 67% v 47%, $p<0.001$).
	Shigayeva et al. (2007)[35]: Working in a SARS unit was significantly associated with consistent adherence to recommended guidelines (OR 4.0, 95% CI, 1.8-8.9), as was working in an intensive care unit (OR 4.3, 95% CI, 2.0-9.0). Employment in a teaching hospital (OR 7.6, 95% CI, 4.8-11.9), SARS unit (OR 3.4, 95% CI, 1.3-9.4) or an intensive care unit (OR 1.9, 95% CI, 0.7-5.0) were associated with safer removal of PPE.
	Jaeger et al. (2011)[38]: 68% of staff in outpatient settings reported no PPE use with index patients, compared to 9% of staff in inpatient setting ($p<0.01$). Mask or N95 respirator use during index patient contact was more common in inpatient (41%) than outpatient settings (5%) ($p<0.01$). Glove use was more common among inpatient (57%) than outpatient (21%) settings. Always donning gloves, gown and either mask or N95 respirator was more common in inpatient (18%) than outpatient (5%) ($p<0.01$).
	Chia et al. (2005)[43]: Using administrative staff as the reference group, individuals working in surgical, medical and intensive care units were more likely to wear the appropriate respiratory protection. However, there was no significant

For peer review only

	difference for those individuals in the other areas (A&E, radiographic services, laboratory services, and others) compared to administrative personnel.
	Ki et al. (2019)[44]: Staff working in the emergency department were significantly more likely than staff in general wards to wear surgical masks (93.0% v 1.8%, p<0.001); however, there were no significant differences in hand-washing or wearing gloves.
	Wong et al. (2004)[45]: Staff working in high-infection districts were significantly more likely to wear gowns (63.0% v 42.0%, p<0.05), wash their hands before going home (82.6% v 63.0%, p<0.05) and use disinfectants to clean surfaces regularly (87.0% v 65.4%, p<0.01); however, they were less likely to quarantine themselves (6.5% v 27.5%, p<0.01) or grant leave to staff until confirmation of diagnosis (41.3% v 71.3%, p<0.01). Staff in public clinics were significantly more likely to wear gowns during consultations than staff in private clinics (81.3% v 45.3%, p<0.05); however, staff in private clinics were more likely to quarantine themselves for a 10-day period after SARS contact than their public sector colleagues (58.1% v 31.3%, p<0.05). Staff in the public sector were more likely to stay away from home.
Contact with confirmed cases	De Perio et al. (2012)[14]: Staff who were present at an aerosol-generating procedure on a patient with pH1N1 (OR 5.20, 95% CI, 1.54-17.55) were more likely to be in the high adherence group.
	Pratt et al. (2009)[18]: Nurses' experience of working directly with confirmed SARS cases was a significant predictor of compliance (p<0.001), with nurses who worked with SARS patients more likely to comply than those who did not. However, this was no longer significant after combining the impact of effort-reward imbalance and burnout.
	Evirgen et al. (2014)[19]: Behaviour was not significantly associated with awareness of suspected or confirmed H1N1 infection in their hospital or their department, or having contact with H1N1 cases.
	Shigayeva et al. (2007)[35]: Recognition of the patient as a SARS case was a significant predictor of consistent adherence to recommended barrier precautions (OR 2.5, 95% CI, 1.5-4.5).

For peer review only - http://bmjopen.bmj.com/site/about/guidelines.xhtml

	Chia et al. (2005)[43]: Staff who regularly came into contact with SARS patients were more likely to wear appropriate respiratory protection compared to those who did not (OR 0.673, 95% CI, 0.587-0.771).
	Wong et al. (2004)[45]: SARS-exposed staff were significantly more likely to insist on patients wearing masks (65.1% v 48.1%, p<0.05) and more likely to ensure their staff wore gloves and masks all the time (33.7% v 11.5%, p<0.01 for gloves, 100% v 92.3%, p<0.01 for masks). However, they were also less likely to quarantine themselves until confirmation of diagnosis (10.8% v 33.3%, p<0.01).
Workload	Pratt et al. (2009)[18]: Hours worked per week were reportedly significantly associated with compliance; however, the authors do not give details about whether more or less hours are associated with greater or lower compliance.
	Chor et al. (2012)[23]: There was a significant decrease in compliance with handwashing before patient contact with the increase in number of weekly patient contacts only in Hong Kong; no significant difference was noted in Singapore or the UK.
	Chau et al. (2008)[30]: Workload was a common barrier to compliance identified by participants.
	Shigayeva et al. (2007)[35]: Work on shifts requiring more frequent patient room entry was associated with lower compliance (OR for 6 or more entries per shift: 0.5, 95% CI, 0.32-0.86). The reported rate of consistent adherence was lower for staff members who reported entering a patient's room more than 5 times per shift: consistent adherence was reported for 84.1% of procedures involving staff members who reported entering the room 1-5 times, compared with 75.5% of procedures involving staff members who reported entering the room 6 or more times (p<0.001).
	Vinck et al. (2011)[36]: The compliance to measures to properly identify suspected cases of respondents who regularly worked overtime was reduced compared with those who did not (p=0.024). Conversely, working overtime was associated with increased compliance with informing patients that they were not supposed to leave their home while ill (p=0.048) and providing patients with the information leaflet (p=0.002). Additionally, compliance with control

For peer review only - http://bmjopen.bmj.com/site/about/guidelines.xhtml

	measures was not influenced by the number of inhabitants within the catchment area of the public health service or by the number of cases for which consultation within the centralised assessment system of each public health service with the Preparedness and Response Unit was carried out (objective workload).
	Moore et al. (2005)[39]: Fatigue was cited as a major cause of failing to follow the proper guidelines, with exhaustion leading to 'sloppy practice'.
	Corley et al. (2010)[41]: Isolation procedures and preventive behaviours created extra workload, frustration and confusion - educating and monitoring external staff and patient's relatives regarding PPE was an extra duty for already overstretched staff and was time-consuming and frustrating.
	Yassi et al. (2005)[46]: The increased time constraints and increased workload were felt to be important barriers to worker adherence to recommendations.
Monitoring of compliance	Hu et al. (2012)[27]: Significantly more nurses than physicians (92.1% v 86.4%, p=0.020) reported being reprimanded by their supervisor for non-compliance. Perceived reprimand by supervisor for non-compliance was a significant predictor of compliance (p=0.035).
	Hsu et al. (2011)[42]: 4.1% of participants believed better policing by infection control staff was the most important strategy for improving hand hygiene, and 3.3% thought that improving public awareness (patients monitoring doctors) was the most important.
	DiGiovanni et al. (2004)[47]: Public health authorities' monitoring of compliance played an important role in establishing the credibility of quarantine for 75% of physicians, 81% of nurses, and 85% of other healthcare workers: however, 58% of physicians, 37% of nurses and 40% of other staff rated the monitoring of their own compliance as bad.

For peer review only - http://bmjopen.bmj.com/site/about/guidelines.xhtml

Patient encounters	De Perio et al. (2012)[14]: 55% of those who reported not using the recommended PPE (n=40) stated it was because they did not know the patient had H1N1 or an influenza-like illness; 20% stated they did not think PPE was needed for the particular activity they were doing; 18% stated it was because the only entered the patients' room for a brief time; 13% stated it was because they did not touch the patient and 10% stated it was because they did not come within six feet of the patient.
	Shigayeva et al. (2007)[35]: Lower rates of consistent adherence were found among staff providing care for patients with higher Acute Physiology and Chronic Health Evaluation (APACHE) II scores – i.e. more severe illness (OR for score APACHE II of 20 or greater: 0.4, 95% CI, 0.28-0.68). Reported rates of consistent adherence were higher when healthcare workers were themselves performing procedures (adjusted OR: 1.7, 95% CI, 1.4-2.2) or assisting with them (adjusted OR: 1.5, 95% CI, 1.1-2.1), compared with procedures that they observed (81% v 79.1% v 74.2%, p<0.001).
Personal protective behaviour-related factors	
Availability of resources	De Perio et al. (2012)[14]: 18% of those who reported not using the recommended PPE (n=40) stated it was because their facility had run out of PPE, while 10% reported that their facility did not provide PPE.
	Hu et al. (2012)[27]: 63.2% reported that appropriate PPE was readily available. Availability of PPE in the ICU was significantly associated with compliance.
	Mitchell et al. (2012)[33]: Staff were significantly more compliant with PPE use when eyewear and gloves were readily available at the point of care; however, PPE availability did not increase compliance with the use of N95 respirators, surgical masks, or gowns.
	Moore et al. (2005)[39]: Participants reported supply problems with face shields and goggles.

	Tan et al. (2006)[40]: Many participants reported that they had not been properly mask-fitted due to lack of proper testing equipment in their clinic. Many were not provided with gowns. Several participants reported lack of goggles and visual shields in their clinics.
	Corley et al. (2010)[41]: Participants believed there were not enough PPE supplies and that guidelines changed depending on supplies running out – for example, surgical masks and plastic aprons became acceptable due to the lack of other PPE. One staff member reported wearing an incorrectly sized mask as their correct size was not in stock.
	Edeghere et al. (2015)[48]: Only 20% of participants experienced barriers in applying infection control measures; the main reported barrier was the occasional unavailability of recommended PPE, most often eye protection.
	Khalid et al. (2016)[49]: 60% perceived inadequate protective measures available to them and cited this as a stressor.
	Locatelli et al. (2012)[50]: Participants reported that stocks of N95 masks ran low so they started using surgical masks, which made it difficult to enforce the use of N95s.
	Rebmann & Wagner (2009)[51]: Many participants reported a lack of resources. Less than half of those whose facility had requested supplies from external agencies actually received them, whereas three participants whose facilities had not requested supplies did receive them. Many reported that supplies were back-ordered and they could not receive them in a timely manner. Theft of supplies was also an issue, with masks and respirators presumably being used or taken by visitors. 26.5% said their facility ran out of respiratory protection. Many reported staff in their facilities were wearing the wrong sizes as they were unable to get the correct sizes.
	Rowlands (2007)[52]: Participants reported that there were not enough masks for staff.
	Van Dijk et al. (2015)[53]: 73.5% believed they had sufficient personal protective materials; 26.5% believed they were insufficient.
	Hsu et al. (2006)[62]: 44% expressed dissatisfaction with shortage of protective equipment such as N95 masks and gloves.

For peer review only - http://bmjopen.bmj.com/site/about/guidelines.xhtml

Perceived difficulty of protective behaviour	Van Dijk et al. (2015)[53]: 35.0% believed recommended personal protective measures were feasible.
	Kang et al. (2018)[54]: Participants reported difficulties with the complexity of using several PPE items together.
Logistical issues	Rebmann & Wagner (2009)[51]: Participants reported logistical issues with needing to fit-test large numbers of staff in a short time.
	Kang et al. (2018)[54]: Limited hospital layouts disrupted space management for PPE use.
	Wong et al. (2012)[55]: Barriers to PPE use included inadequate shower facilities for staff, the small size of the changing room, and the physical layout of the two-bed isolation room which was poor for infectious case management.
Perceived effectiveness	Yap et al. (2010)[4]: There was a significant correlation between attitudes (an 'attitude' score was based on responses to questions on attitudes towards the use of PPE, vaccination, Tamiflu, help-seeking and risk perception) ($r=0.24$, $p<0.01$); there was no such correlation for cases or close contacts who answered the same questions.
	Hu et al. (2012)[27]: Positive attitude towards PPE use (i.e. belief that it provided adequate protection) significantly predicted compliance ($p=0.001$).
	Mitchell et al. (2012)[33]: Staff who felt better protected against influenza when they wore an N95 respirator were significantly more compliant with N95 respirator use than those who did not ($p<0.001$ for aerosol-generating medical procedures and $p=0.04$ for patient care not involving these procedures).
	Parker & Goldman (2006)[34]: Staff considering the combination of all infection control measures to be highly effective in protecting patients from SARS reported increased use of a mask when examining patients compared with staff considering all measures less effective ($p=0.05$). However, handwashing, glove use, gown use, eye protection use, and wearing a mask at all times in the emergency department did not significantly differ between the two groups.

For peer review only - http://bmjopen.bmj.com/site/about/guidelines.xhtml

	Yassi et al. (2005)[46]: Beliefs in the effectiveness of infection control guidelines, as modified by past experiences, were identified as having important influence on worker adherence to procedures.
	Kang et al. (2018)[54]: Participants expressed doubts about the quality and effectiveness of PPE.
Perceived importance	Vinck et al. (2011)[36]: Compliance with the recommendation to consult the Preparedness and Response Unit for centralised assessment of patients was fairly low, with the main reasons for non-compliance being finding it unnecessary (38.3%) or time-consuming (22.7%).
	Hsu et al. (2011)[42]: 20.1% believed that failure to recognise the importance of hand hygiene prevented compliance
Incentives	Jeong et al. (2011)[22]: Perceived benefits of the preventive behaviours significantly predicted higher compliance (OR 3.18, CI 2.15-4.68).
	Hsu et al. (2011)[42]: 19.9% of participants believed that providing positive incentives for hand hygiene was the single most important factor in improving compliance.
	Kang et al. (2018)[54]: Participants reported a lack of incentives to comply with preventive behaviours.
Convenience	De Perio et al. (2012)[14]: 30% of those who reported not using the recommended PPE (n=40) stated it was because the PPE was not available near their patients' room; 10% felt too busy to wear PPE; and 10% stated it was inconvenient to use.
	Hu et al. (2012)[27]: Half of participants reported that PPE use was inconvenient.
	Moore et al. (2005)[39]: Some participants reported that PPE was time-consuming due to having to constantly gown, de-gown, and re-gown.
	Tan et al. (2006)[40]: Participants reported that gowns were inconvenient during toilet breaks.

For peer review only - http://bmjopen.bmj.com/site/about/guidelines.xhtml

	Corley et al. (2010)[41]: Application and removal of PPE was reported to be time-consuming.
	Hsu et al. (2011)[42]: 60.4% believed lack of time prevented hand hygiene compliance.
	Kang et al. (2018)[54]: PPE use was inconvenient in the presence of hospital visitors.
	Lam & Hung (2013)[56]: Participants reported that gowning and de-gowning was very time-consuming.
	Nickell et al. (2004)[57]: 41.7% of participants felt precautionary measures affected their ability to do their job.
Comfort	Mitchell et al. (2012)[33]: Staff who reported always or often feeling comfortable wearing protective eyewear were significantly more likely to wear it (p<0.001). Similarly, staff who reported always or often feeling comfortable wearing an N95 respirator were significantly more compliant with N95 use. Less than 19% of staff reported always or often feeling short of breath, claustrophobic or dizzy when wearing protective eyewear or N95 respirators; no significant differences in compliance were observed between these staff and staff who reported never or rarely feeling short of breath, claustrophobic or dizzy.
	Moore et al. (2005)[39]: Some participants reported that PPE was uncomfortable.
	Tan et al. (2006)[40]: Participants reported physical discomfort from prolonged use of N95 masks, including difficulty breathing, headache, and development of allergic facial rash; they reported moments of complacency where they removed masks intermittently in order to allow normal breathing.
	Corley et al. (2010)[41]: Wearing PPE for extended periods was difficult as it was uncomfortable and could cause dehydration and injuries such as skin peeling.
	Yassi et al. (2005)[46]: Participants identified the need to address the increased amount of worker fatigue that existed when staff worked with full PPE, noting that the discomfort associated with wearing PPE was an important barriers to worker adherence to recommendations.

For peer review only - http://bmjopen.bmj.com/site/about/guidelines.xhtml

	Khalid et al. (2016)[49]: Participants reported that having to wear PPE was stressful.
	Rowlands (2007)[52]: Participants reported that masks were warm and uncomfortable, often leading them to develop headaches or skin rashes, and made it hard to breathe.
	Kang et al. (2018)[54]: Many participants reported having ill-fitted PPE.
	Lam & Hung (2013)[56]: Participants reported that PPE was uncomfortable.
	Nickell et al. (2004)[57]: 85.4% of participants found masks bothersome; of these, 92.9% stated they caused physical discomfort and 13.0% said they gave them a sense of isolation.
	Kang et al. (2018)[58]: Participants reported finding it stressful that their masks did not fit properly and emphasised the discomfort of PPE: they found it hard to breathe or see clearly while wearing it, which made their work harder, and it made them sweaty and dizzy when they wore it for too long.
	Khoo et al. (2005)[59]: Participant opinions on 3M powered air-purifying respirator: 2.3% found it uncomfortable, 11.6% reported moderate discomfort, 32.6% found it tolerable, 30.2% reported slight discomfort, and 23.3% found it very comfortable. 2.3% reported unacceptable visual impairment. 2.3% felt it was suffocating, 4.6% found breathing uncomfortable, 7.0% reported it was comfortable but moderately hard to breathe, 60.5% found it comfortable with minimal difficulty breathing, and 25.6% reported no breathing discomfort. Compared to Stryker powered air-purifying respirator: 4.5% found it uncomfortable, 4.5% reported moderate discomfort, 13.7% found it tolerable, 31.8% reported slight discomfort, and 45.5% found it very comfortable. 4.5% reported unacceptable visual impairment. 0% felt suffocated.
	Straus et al. (2004)[60]: Participants reported that masks hurt, made them sweat and gave them headaches.

For peer review only - http://bmjopen.bmj.com/site/about/guidelines.xhtml

Impact on patient care	Hu et al. (2012)[27]: 21.2% believed that PPE use would interfere with patient care (no significant difference between nurses and physicians). The perception that PPE use would interfere with patient care was significantly associated with poor compliance (p=0.002).
	Tan et al. (2006)[40]: Staff reported that masks muffled their speech and they had to raise their voices to communicate with patients. They also reported that patient reactions to PPE ranged from amusement to apprehension and anxiety; some saw their doctor wearing PPE and assumed they had been exposed to SARS and could infect them and their families.
	Corley et al. (2010)[41]: Wearing PPE was reported to make communication difficult.
	Rowlands (2007)[52]: Participants reported that masks impacted the patient counselling process as they compromised their communication skills and made their speech muffled. They also believed that psychiatric patients with paranoia were sensitive to masks.
	Lam & Hung (2013)[56]: Participants reported that PPE affected their communication with patients, making them have to speak very loudly in order to be heard.
	Nickell et al. (2004)[57]: 85.4% of participants found masks bothersome; of these, 47.0% stated that they caused difficulties communicating.
	Khoo et al. (2005)[59]: Some difficulties regarding communication. Opinions on the 3M powered air-purifying respirator: 0% felt they had to shout but 25.6% felt they had to raise their voice significantly, 37.2% had to raise voice moderately, 32.6% raised voice normally, and 4.6% could speak normally. 2.3% said it caused unacceptable hearing impairment, 11.6% significant, 27.9% moderate, 41.9% mild and 16.3% hearing impairment. Compared to the Stryker powered air-purifying respirator: 0% had to shout, 27.3% raised voice significantly, 31.8% raised voice moderately, 27.3% raised voice mildly, and 13.7% could speak normally. 0% reported unacceptable hearing impairment. About two-thirds of

For peer review only

	respondents agreed (22%) or strongly agreed (42%) that they looked frightening to their patients whenever they used the PAPR.
	Straus et al. (2004)[60]: Participants reported that wearing protective equipment made assessing and communicating with patients a challenge, as they could not establish any of the usual non-verbal cues with patients. They were concerned that patients may have been seen less frequently than usual because of the time required to get into protective clothing.
	Tolomiczenko et al. (2005)[61]: Nurses, more than other occupational groups, reported that infection control measures had an adverse impact on patient care because they were less 'visible' to their patients when gowned, masked and gloved.
Guidance	
Issues with availability or understanding	Alsahafi & Cheng (2016)[28]: Almost two thirds of participants were unaware of guidelines or protocols for the care of infected patients. A high proportion believed that compliance was negatively affected by the lack of policies and procedures (87.3% of nurses, 85% of doctors, 89.4% of other healthcare staff).
	Chau et al. (2008)[30]: Issues with communication and understanding were cited as barriers to compliance. Participants expressed mixed views on the guidelines; some described them as easy to follow and facilitating increased alertness, while others found them difficult to follow and too long (and suggested they be given summaries instead, to get a basic idea of the principles). Other suggestions included tailored information; more programmes in Chinese with a more interactive approach to learning; making courses more practical; and educating the public and patients too.
	Moore et al. (2005)[39]: Participants reported a lack of consistency with safety instructions and frequently changing directives. New levels of enforcement by regulatory agencies interfered with rational infection control practice and infection control policies developed elsewhere had little perceived relevance to their own workplace. Basic infection control policies and procedures were frequently perceived as not well-developed. A major problem was perceived to be

For peer review only - http://bmjopen.bmj.com/site/about/guidelines.xhtml

	issues with communicating the guidelines: participants reported it was difficult to disseminate information simultaneously to all staff; often new information was sent by email, which was not checked before going to work.
	Tan et al. (2006)[40]: Some participants reported not wearing gowns or gloves due to inadequate dissemination of information/instructions from health authorities. However, a SARS seminar and hotline were organised which were perceived to be an effective channel of communication to address concerns and queries on PPE.
	Corley et al. (2010)[41]: Participants perceived a lack of firm recommendations/guidelines regarding what specific PPE was required, which created confusion and made them feel unprotected. They also remarked that current guidelines changed too frequently to keep up. Participants also perceived a lack of clear guidelines and conflicting advice about what to do when a patient is deemed non-infectious.
	Yassi et al. (2005)[46]: To improve adherence to infection control guidelines, participants felt that better enforcement of infection control guidelines was needed but did not want such enforcement to rely on nurses "policing" other professionals.
	Locatelli et al. (2012)[50]: Participants reported many barriers to effective communication about infection control, including information overload, often contradictory information from different sources, and difficulty integrating information from various sources and working out what was a priority. 10 participants reported not having a pre-defined communication plan, or discovered flaws with their plan. Some reported that everyone had to develop their own guidelines, with no clear guidance or resources on how to do so provided. This resulted in confusion about the use of PPE.
	Rebmann & Wagner (2009)[51]: Participants reported a lack of guidance for alternate care sites and non-acute care settings such as physician offices, ambulatory care clinics, long-term care and nursing homes, as well as a general lack of infection prevention information and guidance related to occupational health issues. Rapidly-changing practice

For peer review only

	recommendations were often contradictory which made it difficult to maintain staff compliance and trust, and to stay abreast of the latest recommendations. There was a perceived lack of consistency between sources, with CDC, state health departments, and city health departments all saying different things. Participants reported gaps in communication between departments, or between hospitals and physician offices. Many did not know which type of mask or respirator to use when caring for H1N1 patients, and reported conflicting guidelines related to isolation, masks, and respirator use. 34.3% reported their facility had changed PPE use guidelines and 34.3% reported isolation guidelines had been changed midway through the H1N1 response.
	Van Dijk et al. (2015)[53]: 71.8% believed information provision was well-timed, 65.1% believed information provision was complete, 59.9% believed infection prevention policy changes were well-communicated, 59.2% believed information was explicitly formulated, and 68.5% felt they were clear on when to take protection measures.
	Kang et al. (2018)[54]: Participants described confusion from non-standardized and constantly evolving protocols, making it difficult to develop appropriate PPE protocols and training.
	Wong et al. (2012)[55]: Participants reported difficulties with guidelines as they changed so often.
	Lam & Hung (2013)[56]: Half the participants were satisfied that departmental communication about guidelines was effective, but others had problems with excessive guidelines and rapidly-changing regulations.
	Kang et al. (2018)[58]: Participants cited frequently changing guidelines as a stressor.
	Tolomiczenko et al. (2005)[61]: Emergency room personnel and intensive care unit personnel, more than other groups, reported poor communication about SARS and false ideas about safety precautions, adding that they were not kept up-to-date about what to wear and that personnel at the hospital entrance had no idea what the rules of the day were regarding what should be worn and when.

For peer review only - http://bmjopen.bmj.com/site/about/guidelines.xhtml

	Hsu et al. (2006)[62]: 37% participants reported poor communication between agencies and 30% reported a lack of standard operating procedures.
	Nhan et al. (2012)[63]: Participants reported that communication of guidelines was a barrier to compliance: guidelines were inconsistent between different pandemic management levels and advisory committees, often lacked clarity, were from an overwhelming number of different sources, frequently changed, and were slow to be communicated. Guidelines were also perceived as too rigid to accommodate particular regional or local situations.
	Rambaldini et al. (2005)[64]: Participants reported variability in the information made available at different hospitals, the perceived inconsistency of interhospital and interdepartmental interpretation of public health directives, and the lack of communication between institutions. Some reported frustration at receiving information filtered down from other sources, rather than first-hand. Residents at other hospitals, however, reported receiving regular updates from program directors, chief medical residents and infection control specialists in a variety of forms including websites, email and direct communication via personal conversations or group meetings. The most important information was deemed to be changes to infection control protocols, the status of SARS at their hospital and within the community, and the clinical condition of affected health care colleagues.
	Robertson et al. (2004)[65]: Participants reported a lack of clear guidelines about how to minimise infection at home and in quarantine and uncertainty about effective risk control, leading them to fear contaminating their family members. Unclear guidelines meant that staff developed their own thoughts about which behaviours were necessary and which were not.
Distress and risk perception	

For peer review only

Distress	Chia et al. (2005)[43]: Staff with IES scores of 20 or greater were more likely to wear more effective respiratory protection than those with scores of 19 or less.
	Wong et al. (2004)[45]: Staff who were highly anxious/frightened of dealing with SARS were significantly more likely to insist on patients wearing masks (68.1% v 48.5%, $p<0.05$), to ensure their staff wore gloves at work (32.4% v 17.9%, $p<0.05$), and to wash their hands before entering their home (79.4% v 61.2%, $p<0.05$).
	DiGiovanni et al. (2004)[47]: 5% reported such high stress they were tempted to break quarantine.
Risk perception	Kim & Choi (2016)[20]: Risk perception was significantly positively correlated with preventive behaviours ($p<0.01$).
	Taghrir et al. (2020)[21]: There was a significant negative correlation between preventive behaviours and fear (fear of becoming infected and thinking they are more likely to become infected than others) – as preventive behaviours increased, risk perception declined.
	Jeong et al. (2011)[22]: Perceived susceptibility to the pandemic significantly predicted preventive behaviours.
	Chor et al. (2012)[23]: In Hong Kong, compliance with infection control practices (wearing gloves, changing gloves and washing hands before each patient contact) was associated with the perceived seriousness of the likely impact of pandemic H1N1 on their life; however, compliance with these practices did not correlate with the perceived disease severity among staff in Singapore or the UK.
	Parker & Goldman (2006)[34]: Considering SARS to be a high, rather than low, public health threat was significantly associated with increased compliance with hand-washing, wearing a mask at all times in the emergency department, and wearing gloves when examining patients ($p<0.05$).
	Moore et al. (2005)[39]: Some participants reported that nobody ‘cut corners’ as fear of infection was very real.

For peer review only - http://bmjopen.bmj.com/site/about/guidelines.xhtml

	DiGiovanni et al. (2004)[47]: Staff who directly cared for SARS patients feared becoming infected and passing along infection to their families, so they not only complied with quarantine but also restricted social contact more strictly and for longer than required, with many sending their families away or living alone in their basements; 94% reported the most important reason for complying was to reduce the risk of transmission to others.
	Rebmann & Wagner (2009)[51]: Participants believed that healthcare workers' perceptions of H1N1 severity led to poor compliance with PPE; for example, one participant reported that staff chose not to wear PPE with the idea that they wanted to get mild flu so they wouldn't get a more virulent form of the disease in the autumn.
	Goulia et al. (2010)[66]: Degree of worry about H1N1 was significantly associated with restricting social contacts outside of work because the work environment was considered dangerous (p<0.0005).
Attitudes and behaviours of others	
Non-compliance of others	Hu et al. (2012)[27]: 21% reported their colleagues often forgot to use PPE during patient care.
	Chau et al. (2008)[30]: A common barrier to compliance identified by participants was poor preventive practices by colleagues, patients and visitors.
	Moore et al. (2005)[39]: Basic infection control policies and procedures were frequently perceived as not being enforced; participants identified various deficiencies including tracking who received training, lack of consistent policies for quarantining people, reuse of masks, and lack of consistency in deciding which patients require negative pressure rooms.
	Hsu et al. (2011)[42]: 56.1% of participants believed that senior clinicians leading by example was the most important strategy for improving hand hygiene compliance, and 10.1% believed that peer example (i.e. senior staff not practicing

For peer review only - http://bmjopen.bmj.com/site/about/guidelines.xhtml

	hand hygiene) was a reason for poor compliance. 2.4% of participants believed support and emphasis of guidelines by top administrative staff was the most important strategy for improving hand hygiene compliance.
	Yassi et al. (2005)[46]: The peer environment, especially the compliance of other occupational groups (including physicians), and the feedback from peers were identified as factors that could exert positive or negative influence on individual worker actions.
	DiGiovanni et al. (2004)[47]: As hospitals began to experience critical staff shortages, many modified the recommended quarantine guidelines to allow their staff to return.
	Locatelli et al. (2012)[50]: Participants reported resistance from administration staff about putting up signs with precautions, because they thought it might cause panic.
Attitudes of family members	Moore et al. (2005)[39]: Participants reported that a facilitator of compliance was the attitude of family members, who wanted them to comply with recommended measures as they were scared of getting sick and were often angry or upset that their healthcare worker family member was continuing to work.
	Yassi et al. (2005)[46]: Attitudes of family members, in particular the fear that family members expressed toward contracting SARS, influenced workers' behaviour.
Other potential predictors of compliance	
Complacency	Tan et al. (2006)[40]: Participants reported not wearing gowns due to complacency.
Commitment	Alsahafi & Cheng (2016)[28]: 96.2% of nurses, 93.6% of physicians and 98.4% of other healthcare staff believed that healthcare workers' lack of commitment to policies and procedures reduced compliance.

For peer review only - http://bmjopen.bmj.com/site/about/guidelines.xhtml

	Moore et al. (2005)[39]: Participants reported a lack of commitment to occupational health and safety by workers and management.
Previous experience with pandemic	Moore et al. (2005)[39]: Some participants reported that past exposures to disease led to decreased compliance when experience demonstrated that protective behaviours are not needed 100% of the time.
Passing of time	Rebmann & Wagner (2009)[51]: Participants reported that compliance lowered with time, as it was hard to keep vigilant in the middle of outbreak.
Forgetting	Hu et al. (2012)[27]: Approximately 21% reported they forgot to change PPE between patients. Forgetting to use or change PPE was significantly associated with poor compliance ($p<0.001$).
Death of a doctor	Tan et al. (2006)[40]: Compliance improved after the death of a doctor friend.
Effort-reward imbalance	Pratt et al. (2009)[18]: Effort-reward imbalance ratio was significantly predictive of decreased compliance ($p<0.05$).
Consulting with experts	Manabe et al. (2012)[15]: Consulting with experts with experience of caring for H5N1 patients did not significantly predict compliance with recommended behaviours ($p=0.776$).
Lack of staff	Moore et al. (2005)[39]: Participants felt there were not enough infection control practitioners, or they were not visible enough.
	Corley et al. (2010)[41]: Participants reported that not having enough staff (or enough experienced staff working at once) was a barrier to compliance.
PPE fit-testing procedures	Moore et al. (2005)[39]: Participants reported the fit-testing process for PPE was not standardised and some felt it was unnecessary.
Professionalism	Yassi et al. (2005)[46]: Professionalism was identified as having an important influence on adherence to procedures.

For peer review only - http://bmjopen.bmj.com/site/about/guidelines.xhtml

Use of public transport	Evirgen et al. (2014)[19]: Behaviour was not significantly associated with using public transportation to get to work.
Loss of income	DiGiovanni et al. (2004)[47]: Fear of loss of income was an obstacle to complying with quarantine, particularly among those unconvinced quarantine was necessary – 60% of doctors, 76% of nurses and 70% of other healthcare workers said they would need fairly detailed information about when, how, and how much compensation they would receive as encouragement to comply with quarantine.
Overcrowding	Alsahafi & Cheng (2016)[28]: Overcrowding in the emergency department was cited as a reason for poor compliance (97.2% of nurses, 96.6% of physicians, 93.2% of other healthcare staff)

For peer review only - http://bmjopen.bmj.com/site/about/guidelines.xhtml

Appendix I. Search strategy

Search 1: Compliance-related terms

compliance OR comply OR adhere* OR attitude* OR behaviour* OR behavior*

Search 2: Infection control-related terms

hygien* OR handwashing OR hand-washing OR infection control OR mask* OR PPE OR protective equipment OR social distanc*

Search 3: Healthcare worker-related terms

healthcare worker* OR healthcare staff OR healthcare personnel OR "medical personnel" OR "hospital personnel" OR frontline OR medical staff OR medical worker* OR doctor* OR dentist* OR midwife* OR midwives OR surgeon* OR nurse* OR gp* OR general practitioner* OR physician* OR health care worker* OR health care staff OR hospital employee* OR medical employee* OR healthcare employee* OR health care employee* OR hospital worker* OR hospital staff

Search 4: Emerging infectious disease-related terms

SARS OR severe acute respiratory syndrome OR MERS OR middle east respiratory syndrome OR H1N1 OR swine flu OR H5N1 OR avian influenza OR bird flu OR pandemic* OR coronavirus OR COVID-19 OR n-COV OR SARS-COV-2

Searches 1, 2, 3 and 4 were combined with AND.

For peer review only

For peer review only - http://bmjopen.bmj.com/site/about/guidelines.xhtml

Appendix II. Flow diagram of screening process

PRISMA 2009 Flow Diagram

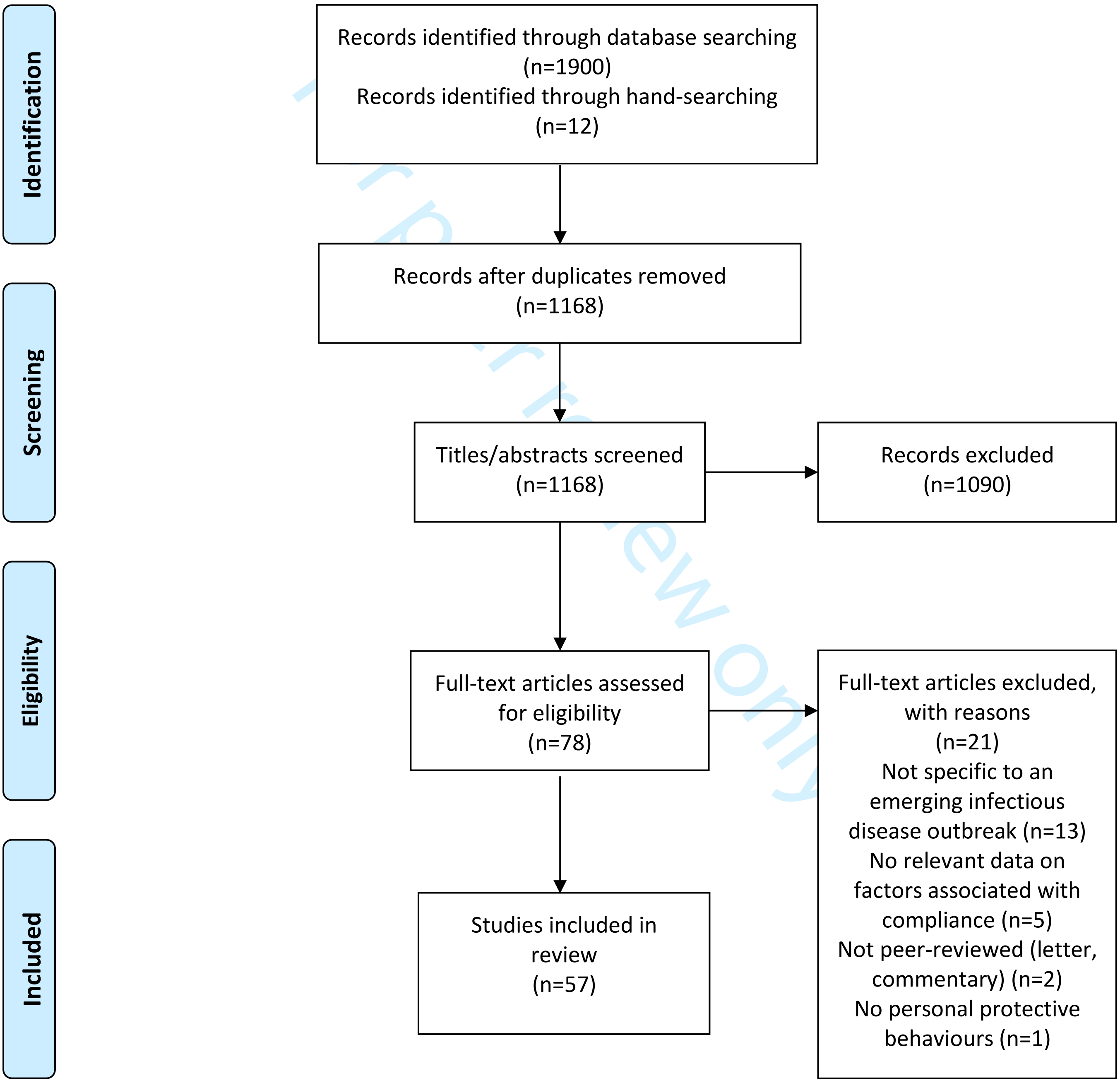

PRISMA 2009 Checklist

Section/topic	#	Checklist item	Reported on page #
**TITLE**			
Title	1	Identify the report as a systematic review, meta-analysis, or both.	1
**ABSTRACT**			
Structured summary	2	Provide a structured summary including, as applicable: background; objectives; data sources; study eligibility criteria, participants, and interventions; study appraisal and synthesis methods; results; limitations; conclusions and implications of key findings; systematic review registration number.	2
**INTRODUCTION**			
Rationale	3	Describe the rationale for the review in the context of what is already known.	3
Objectives	4	Provide an explicit statement of questions being addressed with reference to participants, interventions, comparisons, outcomes, and study design (PICOS).	3
**METHODS**			
Protocol and registration	5	Indicate if a review protocol exists, if and where it can be accessed (e.g., Web address), and, if available, provide registration information including registration number.	n/a
Eligibility criteria	6	Specify study characteristics (e.g., PICOS, length of follow-up) and report characteristics (e.g., years considered, language, publication status) used as criteria for eligibility, giving rationale.	4
Information sources	7	Describe all information sources (e.g., databases with dates of coverage, contact with study authors to identify additional studies) in the search and date last searched.	4
Search	8	Present full electronic search strategy for at least one database, including any limits used, such that it could be repeated.	Supplementary material
Study selection	9	State the process for selecting studies (i.e., screening, eligibility, included in systematic review, and, if applicable, included in the meta-analysis).	4
Data collection process	10	Describe method of data extraction from reports (e.g., piloted forms, independently, in duplicate) and any processes for obtaining and confirming data from investigators.	4
Data items	11	List and define all variables for which data were sought (e.g., PICOS, funding sources) and any assumptions and simplifications made.	4
Risk of bias in individual studies	12	Describe methods used for assessing risk of bias of individual studies (including specification of whether this was done at the study or outcome level), and how this information is to be used in any data synthesis.	n/a – rapid review so not all steps of a systematic review were

For peer review only - http://bmjopen.bmj.com/site/about/guidelines.xhtml

PRISMA 2009 Checklist

			followed, as recommended by WHO (2017) guidelines
Summary measures	13	State the principal summary measures (e.g., risk ratio, difference in means).	n/a
Synthesis of results	14	Describe the methods of handling data and combining results of studies, if done, including measures of consistency (e.g., I^2) for each meta-analysis.	4

Section/topic	#	Checklist item	Reported on page #
Risk of bias across studies	15	Specify any assessment of risk of bias that may affect the cumulative evidence (e.g., publication bias, selective reporting within studies).	n/a
Additional analyses	16	Describe methods of additional analyses (e.g., sensitivity or subgroup analyses, meta-regression), if done, indicating which were pre-specified.	n/a
**RESULTS**			
Study selection	17	Give numbers of studies screened, assessed for eligibility, and included in the review, with reasons for exclusions at each stage, ideally with a flow diagram.	5 / supplementary material
Study characteristics	18	For each study, present characteristics for which data were extracted (e.g., study size, PICOS, follow-up period) and provide the citations.	Supplementary material
Risk of bias within studies	19	Present data on risk of bias of each study and, if available, any outcome level assessment (see item 12).	n/a
Results of individual studies	20	For all outcomes considered (benefits or harms), present, for each study: (a) simple summary data for each intervention group (b) effect estimates and confidence intervals, ideally with a forest plot.	n/a
Synthesis of results	21	Present results of each meta-analysis done, including confidence intervals and measures of consistency.	n/a
Risk of bias across studies	22	Present results of any assessment of risk of bias across studies (see Item 15).	n/a
Additional analysis	23	Give results of additional analyses, if done (e.g., sensitivity or subgroup analyses, meta-regression [see Item 16]).	n/a
**DISCUSSION**			
Summary of evidence	24	Summarize the main findings including the strength of evidence for each main outcome; consider their relevance to key groups (e.g., healthcare providers, users, and policy makers).	11-12

For peer review only - http://bmjopen.bmj.com/site/about/guidelines.xhtml

PRISMA 2009 Checklist

Limitations	25	Discuss limitations at study and outcome level (e.g., risk of bias), and at review-level (e.g., incomplete retrieval of identified research, reporting bias).	13-14
Conclusions	26	Provide a general interpretation of the results in the context of other evidence, and implications for future research.	14
**FUNDING**			
Funding	27	Describe sources of funding for the systematic review and other support (e.g., supply of data); role of funders for the systematic review.	Title page (submitted as supplementary material for editor)

From: Moher D, Liberati A, Tetzlaff J, Altman DG, The PRISMA Group (2009). Preferred Reporting Items for Systematic Reviews and Meta-Analyses: The PRISMA Statement. PLoS Med 6(7): e1000097. doi:10.1371/journal.pmed1000097

For more information, visit: **www.prisma-statement.org**.

For peer review only

For peer review only - http://bmjopen.bmj.com/site/about/guidelines.xhtml
